# Homochiral versus Racemic 2D Covalent Organic Frameworks

**DOI:** 10.1021/jacs.5c01004

**Published:** 2025-05-15

**Authors:** José del Refugio Monroy, Tejas Deshpande, Joël Schlecht, Clara Douglas, Robbie Stirling, Niklas Grabicki, Glen J. Smales, Zdravko Kochovski, Filippo Giovanni Fabozzi, Stefan Hecht, Sascha Feldmann, Oliver Dumele

**Affiliations:** † Department of Chemistry & Center for the Science of Materials Berlin, 9373 Humboldt-Universität zu Berlin, Brook-Taylor-Strasse 2, Berlin 12489, Germany; ‡ Institute of Organic Chemistry, 9174 Albert-Ludwigs-Universität Freiburg, Albertstrasse 21, Freiburg 79104, Germany; § Institute of Chemical Sciences and Engineering, 27218 École Polytechnique Fédérale de Lausanne, Rue de l’Industrie 17, Sion 1951, Switzerland; ∥ 42220 Bundesanstalt für Materialforschung und -prüfung, Unter den Eichen 87, Berlin 12205, Germany; ⊥ Institute of Electrochemical Energy Storage, Helmholtz-Zentrum Berlin für Materialien und Energie, Hahn-Meitner-Platz 1, Berlin 14109, Germany; # Freiburg Materials Research Center, Albert-Ludwigs-Universität Freiburg, Stefan-Meier-Strasse 21, Freiburg 79104, Germany; ∇ Freiburg Center for Interactive Materials and Bioinspired Technologies, Albert-Ludwigs-Universität Freiburg, Georges-Köhler-Allee 105, Freiburg 79110, Germany

## Abstract

The synthesis of homochiral two-dimensional covalent organic frameworks (2D COFs) from chiral π-conjugated building blocks is challenging, as chiral units often lead to misaligned stacking interactions. In this work, we introduce helical chirality into 2D COFs using configurationally stable enantiopure and racemic [5]­helicenes as linkers in the backbone of 2D **[5]­HeliCOFs** as powders and films. Through condensation with 1,3,5-triformylbenzene (TFB) or 1,3,5-triformylphloroglucinol (TFP), our approach enables the efficient formation of a set of homochiral and racemic 2D **[5]­HeliCOFs**. The resulting carbon-based crystalline and porous frameworks exhibit distinct structural features and different properties between homochiral and racemic counterparts. Propagation of helical chirality into the backbone of the crystalline frameworks leads to the observation of advanced chiroptical properties in the far-red visible spectrum, along with a less compact structure compared with the racemic frameworks. Homogeneous thin films of **[5]­HeliCOFs** disclosed photoluminescent properties arising from the controlled growth of highly ordered π-conjugated lattices. The present study offers insight into general chiral framework formation and extends the Liebisch–Wallach rule to 2D COFs.

## Graphical abstract

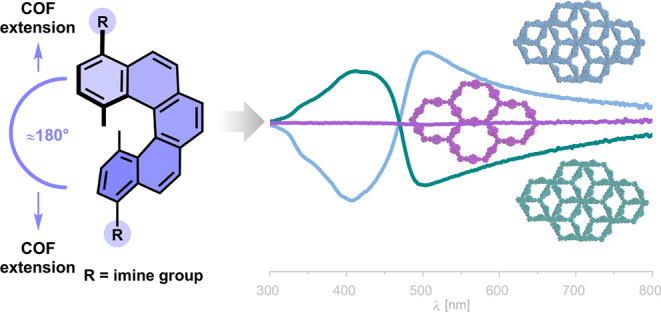

## Introduction

Homochiral carbon-based architectures with complex structural features can establish unique connections between molecular and global chirality.^1–3^ The amplified chiroptical properties of such all-carbon structures usually are linked to their dimensionality – being either discrete molecules,^4,5^ macromolecules,^6,7^ or supramolecular assemblies.^8,9^ For example, organic homochiral polymers frequently exhibit amplified chiroptical properties compared to their unichiral building blocks.^10,11^ Hence, homochiral structures, derived from precisely designed enantiopure organic units, offer an attractive opportunity to be explored in fundamental research and functional materials.

Conceptually, reticular chemistry allows the incorporation of chiral building blocks across highly periodic frameworks, integrating their initial chiral information into novel salient functionalities of infinite chiral structures.^12^ In particular, covalent organic frameworks (COFs) are an interesting example of highly ordered and porous solids with fine-tunable structures.^13^ Until now, introducing enantiopure building blocks into two-dimensional (2D) COFs has remained a synthetic challenge, as the intrinsic nonplanarity of the chiral units could cause deviations in the stacking of crystalline lattices.^14–16^ For instance, the first example of 2D chiral COFs was achieved by postsynthetic side-chain modification from planar building blocks to avoid the presumed steric mismatch in the backbone of the 2D lattices (Figure 1a).^17^ Subsequently, various approaches have been developed to incorporate or induce chirality into 2D COFs, including chiral-preorganized building blocks,^18–22^ metal-template synthesis,^23–26^ enrichment with enantiopure biomolecules,^27–29^ and using enantiopure modulators.^30–32^ Such advanced 2D homochiral COFs are mainly studied in chiral separation technology,^33,34^ enantioselective catalysis,^35–37^ light-emissive materials,^38–40^ nanoarchitectonics,^41–43^ and fundamental studies^44^ – such as the formation of metal-helical rods.^45^

**Figure 1:**
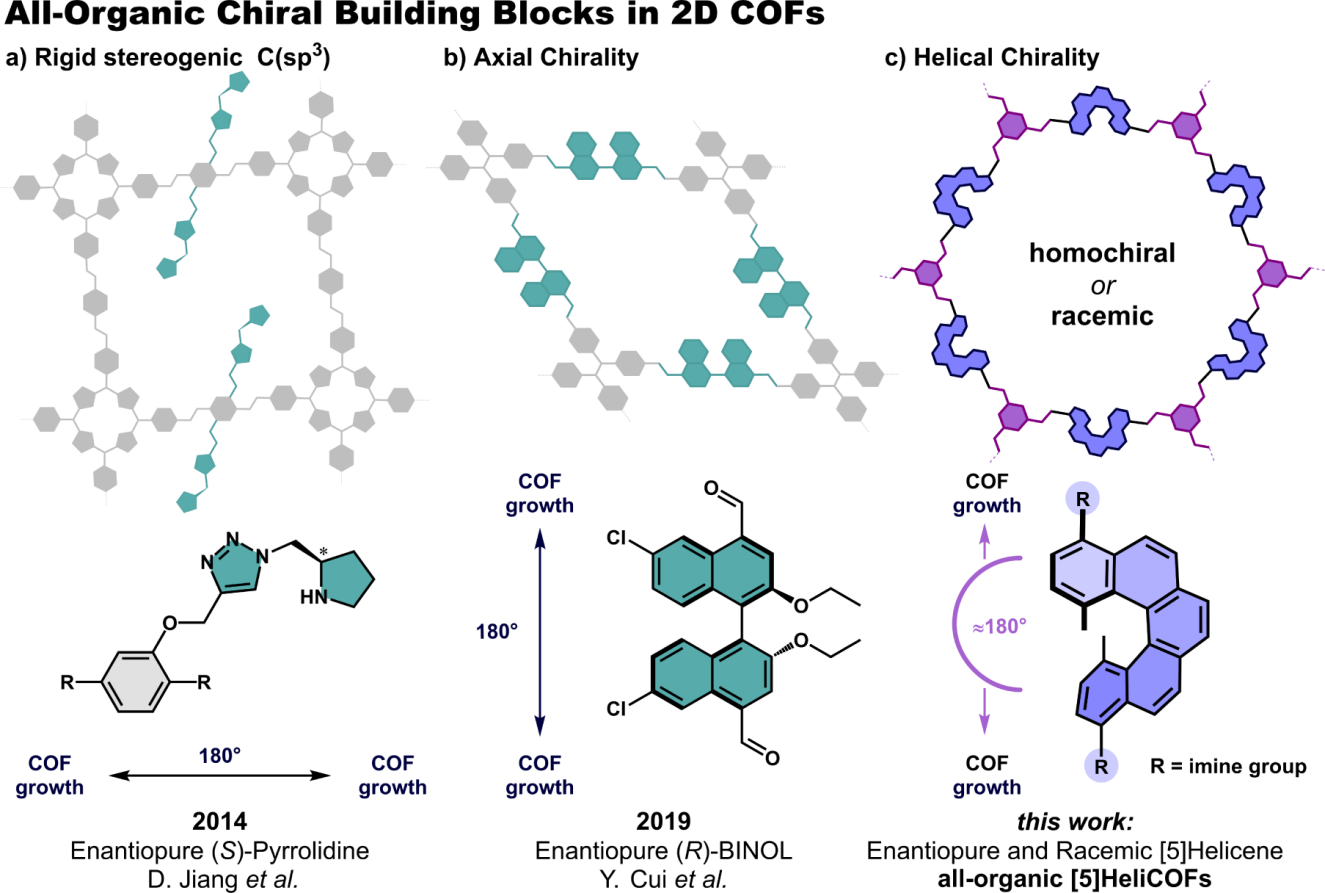
Representative examples of previously reported carbon-based chiral building blocks for the synthesis of 2D chiral COFs; (a) homochiral 2D COFs bearing the C­(sp^3^) stereocenter in the side chain;^17^ (b) incorporation of axial chirality in the backbone of 2D homochiral COFs from (*R*)-BINOL based building blocks;^52^ (c) in this work, incorporation of helical chirality into the backbone of 2D COFs with configurationally stable [5]­helicenes as *pseudo*-C_2_ linear linkers for the synthesis of all-organic 2D **[5]­HeliCOFs**.

Currently, nonplanar building blocks have been integrated into the backbone of 2D COFs,^46^ leading to the development of “twisted” architectures, which no longer rely on planar units.^47–51^ For example, axial chirality has been incorporated into homochiral 2D COFs with a precisely designed π-conjugated (*R*)-BINOL derivative as a *pseudo*-*C*_2_ linker (Figure 1b).^52^ However, such advanced homochiral 2D COFs are rarely synthesized using enantiopure π-conjugated building blocks due to their challenging multistep synthesis.^53–56^

Despite the growing interest in homochiral 2D COFs, reports on two opposite homochiral lattices versus their racemic analogues are rare.^38,39,45^ Systematic studies on these fundamental structural differences could help to derive general trends regarding the influence of the absolute configuration of the building blocks on the formation of homochiral 2D frameworks.^57^ Inherently, a high degree of crystallinity is an essential property of COFs.^58^ In analogy to the Liebisch–Wallach rule,^59,60^ which states that racemic single crystals are more densely packed and usually preferred over their homochiral counterparts,^61,62^ racemic building blocks should lead to 2D COFs with higher density than their homochiral versions. Following this rule, racemic 2D COFs should also possess a higher degree of crystallinity than their homochiral analogues due to preferred stacking interactions based on the complementary geometries from their racemic units. However, the structural analogy of the Liebisch–Wallach rule to the growth of homochiral versus racemic lattices has not been experimentally studied in all-organic 2D framework materials.^63^ As a result of this fundamental comparison,^64–67^ it would be possible to discover general structural tendencies that eventually lead to rationally designed homochiral (or racemic) 2D COF architectures with unique topologies and large chiroptical activity, revealing further potential applications of these novel materials.

In this context, [*n*]­helicenes^68^ have led to intensive investigation of their photophysical and electronic properties, drawing attention to their incorporation into advanced unichiral architectures,^69–72^ homochiral polymers,^73–75^ and noncovalent assemblies.^76^ Hence, incorporating enantiopure [*n*]­helicenes into the backbone of 2D COFs could reveal structural differences in homochiral versus racemic 2D frameworks. Racemic [*n*]­helicenes have been used in 2D COFs – however, their chiroptical properties have not been addressed since only racemic solids have been reported.^77,78^ Hence, helical chirality remains unexplored in homochiral 2D COFs. Currently, there is no systematic report on the structural preferences derived from homochiral versus racemic 2D frameworks using chiral π-conjugated units.^57^

In this work, we introduce carbon-based helical chirality into the backbone of imine-linked homochiral (and racemic) 2D COFs by using enantiopure (or racemic) configurationally stable [5]­helicene derivatives as *pseudo*-*C*_2_ linkers for the synthesis of all-organic 2D **[5]­HeliCOFs** (Figure 1c). We evaluated systematically the influence of their absolute structural configuration under identical experimental conditions on the formation of homochiral and racemic 2D **[5]­HeliCOFs** that displayed remarkable crystalline, porous, and extended chiroptical properties.

## Results and Discussion

### Design and Synthesis of [5]­Helicene Building Blocks

We identified that 4,11-substituted [5]­helicenes could be tested as *pseudo*-*C*_2_ linkers for the synthesis of homochiral (or racemic) 2D COFs (Figure 1c). This substitution pattern offers a nearly 180° angle required for a formal *C*_2_ linker to be incorporated into the backbone of extended 2D lattices, while also exhibiting a nonplanar geometry, enabling the incorporation of carbon-based helical chirality into the backbone of the homochiral 2D frameworks. Commonly, enantiopure [5]­helicene racemizes in solution at 25 °C.^79^ To avoid its epimerization, methyl substituents were introduced at the 1,14-positions of the [5]­helicene core,^80^ following the synthetic approach developed by Juríček and coworkers.^81^ This precise modification allows enantiopure [5]­helicene building blocks to retain their initial absolute configuration during the standard 2D COF synthesis (ca. 120 °C).

At first, the racemic [5]­helicene core, (±)-4,11-dibromo-1,14-dimethyl[5]­helicene ((±)-**1**) was synthesized on an enlarged scale following a photocyclodehydrogenation reaction (Section S2).^82^ The bromine atoms in the 4- and 11-positions of (±)-**1** allow the incorporation of diverse substituents for the synthesis of 2D **[5]­HeliCOFs**. For instance, *N*-aryl benzophenone imines have proven to be effective moieties for the synthesis of highly crystalline imine-linked 2D COFs.^83,84^ In addition, these protected building blocks are more stable to oxidation in air than their free amine versions.^85,86^ A brief theoretical analysis suggests that directly incorporating imines in the 4- and 11-positions of (±)-**1** would lead to steric hindrance between the H_α_ of the [5]­helicene core and the adjacent imine during COF crystallization (Figure S131). Thus, we aimed for the incorporation of *N*-aryl benzophenone imine groups with an additional phenyl ring to extend the racemic [5]­helicene core (±)-**1** and reduce potential steric limitations during 2D COF growth. This was achieved by Suzuki–Miyaura cross-coupling with boronic ester **2** to obtain (±)-**3** in excellent yield (90%, Scheme 1). After the acidic hydrolysis of the imine groups, unambiguous structural proof of the racemic diamine (±)-**3b** was obtained by single-crystal X-ray analysis, exhibiting a 19.3° deviation from a formal *C*_2_ linker, induced by its helical nature (Figure S125).

**Scheme 1:**
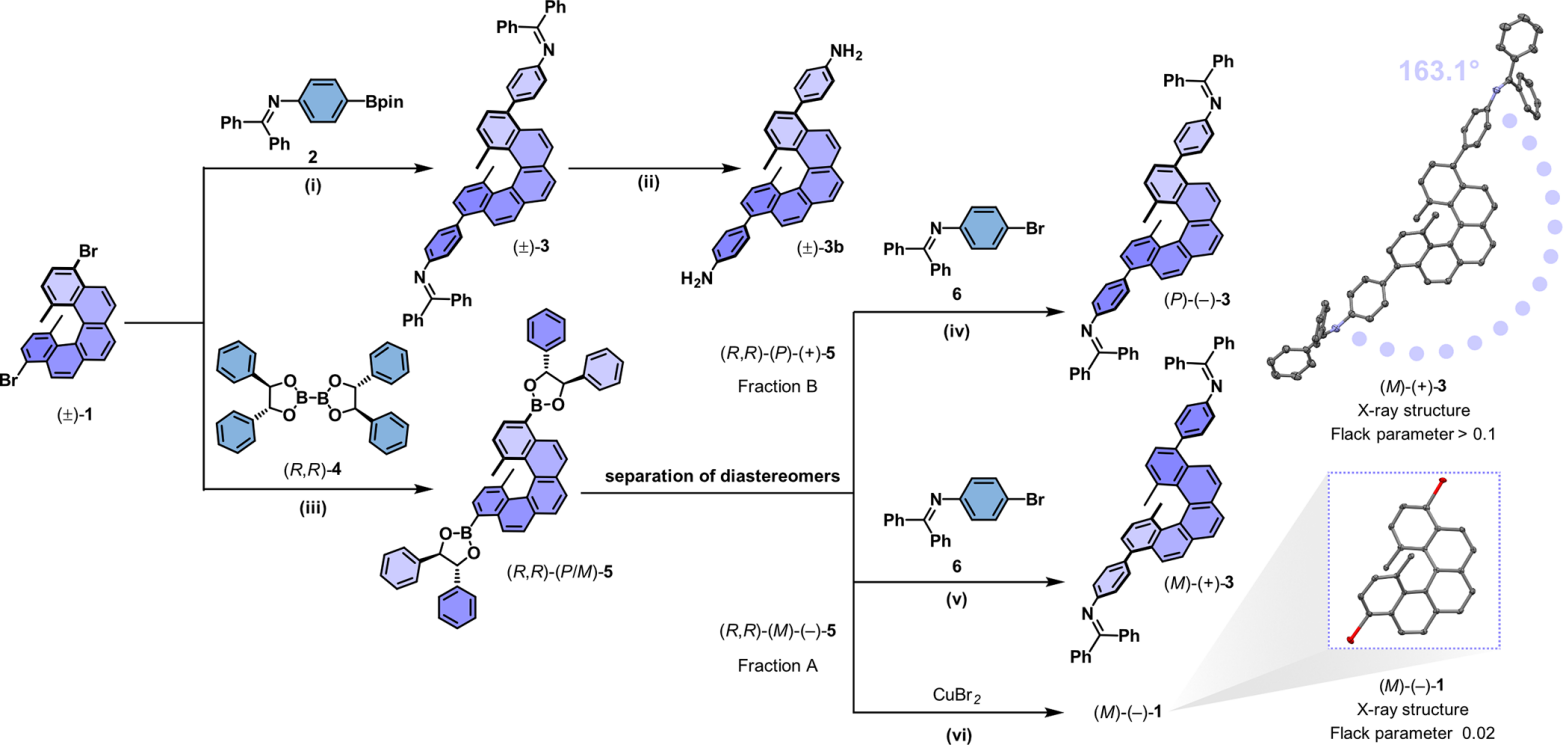
Synthesis of Racemic and Enantiopure [5]­Helicene Building Blocks^a^

a Reagents and conditions: (i) **2**, Pd­(PPh_3_)_4_, Cs_2_CO_3_, DMF/water 4:1, 110 °C, 16 h, 90%; (ii) aq. AcOH (6 M), 1,4-dioxane, 70 °C, 2 h, 80%; (iii) (*R*,*R*)-(+)-**4**, [Pd­(dppf)­Cl_2_·CH_2_Cl_2_], CH_3_COOK, 1,4-dioxane, 80 °C, 45 min, 87%; (iv) (*R*,*R*)-(*P*)-(+)-**5**, **6**, Pd­(PPh_3_)_4_, Cs_2_CO_3_, DMF/water 4:1, 110 °C, 20 h, 68%; (v) (*R*,*R*)-(*M*)-(−)-**5**, **6**, Pd­(PPh_3_)_4_, Cs_2_CO_3_, DMF/water 4:1, 110 °C, 20 h, 75%; (vi) (*R*,*R*)-(*M*)-(−)-**5**, CuBr_2_, methanol/water 4:1, 85 °C, 12 h, 98%; (inset) single-crystal X-ray structure of (*M*)-(+)-**3** or (*P*)-(−)-**3** with a Flack parameter >0.1 (angle measured along atoms N–C_Ph_ −N), and single-crystal X-ray structure of the enantiopure (*M*)-(−)-**1** with a Flack parameter = 0.02 (hydrogen atoms are omitted for clarity).

An alternative synthetic route was established to obtain the enantiopure building blocks (*P*)-(−)-**3** and (*M*)-(+)-**3** (Scheme 1). In this case, the chiral boronate auxiliary (*R*,*R*)-bis­(hydrobenzoinato)­diboron (*R*,*R*)-**4** was coupled to the parent [5]­helicene core (±)-**1** in a Miyaura borylation to obtain the mixture of diastereomers (*R*,*R*)-(*P*/*M*)-**5** in good yield (87%, Scheme 1).^87^ Next, the diastereomeric mixture was resolved using preparative high-performance liquid chromatography with a Pirkle-type chiral stationary phase (Figures S103–S105).^88^ Subsequently, the pure diastereomers (*R*,*R*)-(*P*)-(+)-**5** (*ee* = 99.6%) and (*R*,*R*)-(*M*)-(−)-**5** (*ee* = 99.9%) were subjected to cross-coupling reaction with **6**, readily losing the chiral auxiliary and leading to the enantiopure diimines (*P*)-(−)-**3** and (*M*)-(+)-**3** in good yields (68% and 75%, respectively, Scheme 1). Single crystals suitable for X-ray analysis were obtained for (*M*)-(+)-**3** (Figure S126). However, the absolute configuration was not directly assigned with high accuracy from the measured crystals, due to weak resonant scattering caused by the absence of heavy atoms in (*M*)-(+)-**3**.^89,90^ This X-ray analysis only provided a general structure of the [5]­helicene building block with *N*-benzophenone imines (Scheme 1), exhibiting a 16.9° deviation from a formal linear *C*_2_ linker due to its helical nature. To determine the absolute configuration, the isolated diastereomer (*R*,*R*)-(*M*)-(−)-**5** was subjected to a direct bromodeboronation to obtain the enantiopure dibromo[5]­helicene (*M*)-(−)-**1** in excellent yields (98%, Scheme 1, bottom).^91^ The bromine atoms allowed the direct assignment of the absolute configuration with high accuracy of (*M*)-(−)-**1** from single-crystal X-ray analysis (Figure S127). Thus, [5]­helicene derivatives synthesized from the diastereomer (*R*,*R*)-(*M*)-(−)-**5** hold an (*M*)-configuration. Additionally, the absolute configuration of the [5]­helicene building blocks was validated using a combination of electronic circular dichroism (ECD) spectroscopy, optical rotatory dispersion, and time-dependent density functional theory (TD-DFT) calculations (Figures S109 and S110).^92^

For a comparison of the crystallization of **[5]­HeliCOFs**, we report the synthesis of [5]­helicene diimines without phenyl spacers: (±)-**6**, (*R*,*R*)-(*P*/*M*)-**7**, (*R*,*R*)-(*P*)-(+)-**7**, and (*R*,*R*)-(*M*)-(−)-**7** (Scheme S2). Unambiguous structural proof of these alternative [5]­helicene building blocks was achieved by single-crystal X-ray analysis (Figures S128–S130), and the diastereomer separation is detailed in the Figures S106–S108. Hence, our strategy enables rapid access to enantiomerically pure and racemic [5]­helicenes in sufficient quantities for a comparative study of the synthesis of homochiral and racemic 2D **[5]­HeliCOFs**.

### Synthesis of Racemic [5]­HeliCOFs

Given its direct synthetic accessibility, the formation of racemic **[5]­HeliCOFs** was initially studied by using the *pseudo*-linear linker (±)-**3**. We chose 1,3,5-triformylbenzene (TFB) and 1,3,5-triformylphloroglucinol (TFP) as nodes (Scheme 2). In brief, the *rac*-**[5]­Heli-TFB** COF was synthesized under acidic solvothermal conditions with 21 equiv of aq. AcOH (6 M) using 1:1 *o*-DCB/*n*-BuOH solvent mixture at 120 °C for 72 h, with a yield of 69% (for experimental screening of reaction conditions, see Table S1). The formation of a highly crystalline solid was confirmed by powder X-ray diffraction (PXRD) of the *rac*-**[5]­Heli-TFB** COF (Figure 2a), where narrow and intense reflexes at low 2θ angles can be observed. Here, the *rac*-**[5]­Heli-TFB** COF exhibits an intense reflex at 2θ = 2.4°, assigned to the (100) facet, and lower intense reflexes at 4.7° and 7.2°, which correspond to the (200) and (120) planes, in agreement with the simulated diffraction pattern of a hexagonal AA-inclined stacking mode (Figure S89).^93^ The Pawley refinement provided good agreement with the hexagonal proposed model, yielding refined lattice parameters *a* = *b* = 45.7 Å and *c* = 7.4 Å, with *R*_wp_ = 2.2 and *R*_p_ = 4.2, and *P*1 as the space group (see expansion in Figure S92). Simulating nonplanar lattices with standard crystallographic methods is particularly challenging due to potential disorder scenarios.^94^ In the proposed hexagonal AA-inclined model, the (*P*)- and (*M*)-[5]­helicene cores are arranged on top of each other, alternating their *fjord* regions over the racemic 2D lattices (Figure S89). This alternating pair-packing of complementary enantiomers has been observed in (±)-[*5*]­helicene single crystals^95,96^ and previously reported racemic [*n*]­helicene-based COFs (*n* > 6),^77,78^ suggesting that the racemic *pseudo*-linear linker (±)-**3** has been successfully incorporated into the extended crystalline frameworks. Furthermore, the racemic periodic lattices of the *rac*-**[5]­Heli-TFB** COF were directly visualized using low-dose cryogenic high-resolution transmission electron microscopy (HR-TEM) (Figure 2b), where perfect hexagonal domains can be observed along the [001] plane. In addition, the corresponding fast Fourier transform (FFT) pattern of the HR-TEM image supports a hexagonal symmetry with a *d*-spacing of 4.08 nm (Figure 2b). Thus, the direct visualization of honeycomb-type 2D lattices is in good agreement with the experimental PXRD diffractogram and supports the proposed highly symmetric hexagonal structural model with an AA-inclined stacking mode. Subsequently, the porosity of the *rac*-**[5]­Heli-TFB** COF was investigated using isothermal N_2_-sorption measurements at 77 K (Figure S44). The adsorption data show two inflection points below *p*/*p*_0_ = 0.2, associated with mesoporous materials according to a type IV isotherm.^97^ Also, the surface area was calculated using the Brunauer–Emmett–Teller (BET) method, showing a porosity value of 504 m^2^ g^–1^.^98^ Furthermore, the pore size distribution has been estimated using nonlinear density functional theory (NL-DFT), with a maximum of around 35.7 Å (Figure S55). Next, the morphology of the *rac*-**[5]­Heli-TFB** COF was observed in the FE-SEM micrographs (Figure S70), showing spherical-shaped particles with diverse diameters ranging from ca. 1.8 to 8.8 μm. Furthermore, its thermal stability was confirmed up to 350 °C by thermogravimetric (TGA) analysis (Figure S79). Additionally, the Fourier-transform infrared (FT-IR) spectrum shows the C=N stretch band at 1595 cm^–1^ and the absence of the C=O stretch band of TFB at 1695 cm^–1^, indicating the formation of imine linkages (Figure S83), supported by the ^13^C cross-polarization magic-angle spinning nuclear magnetic resonance (CP-MAS NMR) spectrum, showing a signal at 156.6 ppm (Figure S86). Our findings indicate that the *rac*-**[5]­Heli-TFB** COF is a crystalline organic solid with a 2D honeycomb architecture and mesoporous structure.

**Scheme 2:**
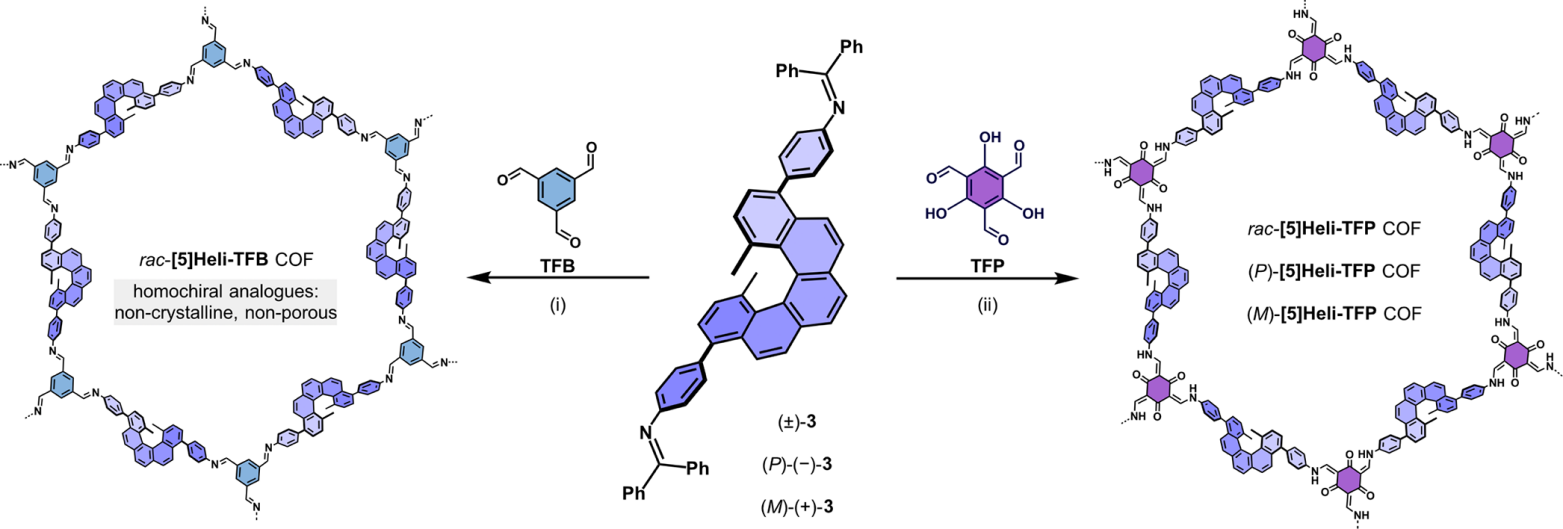
Acidic Solvothermal Synthesis of Racemic and Homochiral 2D **[5]­HeliCOFs** Based on 1,3,5-Triformylbenzene (TFB) and 1,3,5-Triformylphloroglucinol (TFP) as Nodes and Testing (±)-**3**, (*P*)-(−)-**3** and (*M*)-(+)-**3** as *Pseudo*-*C*_2_ Linkers[Fn sch2-fn2]

a Reagents and experimental conditions: (i) TFB, aq. AcOH (6 M, 21 equiv), *o*-DCB/*n*-BuOH 1:1, 120 °C, 72 h, 69% *rac*-**[5]­Heli-TFB** COF (homochiral analogues were not obtained under identical experimental conditions); (ii) TFP, aq. AcOH (6 M, 40 equiv), aniline (0.7 M, 1.5 equiv), *o*-DCB/*n*-BuOH 4:1, 120 °C, 72 h, 96% *rac*-**[5]­Heli-TFP** COF, 96% (*P*)-**[5]­Heli-TFP** COF, and 90% (*M*)-**[5]­Heli-TFP** COF.

**Figure 2:**
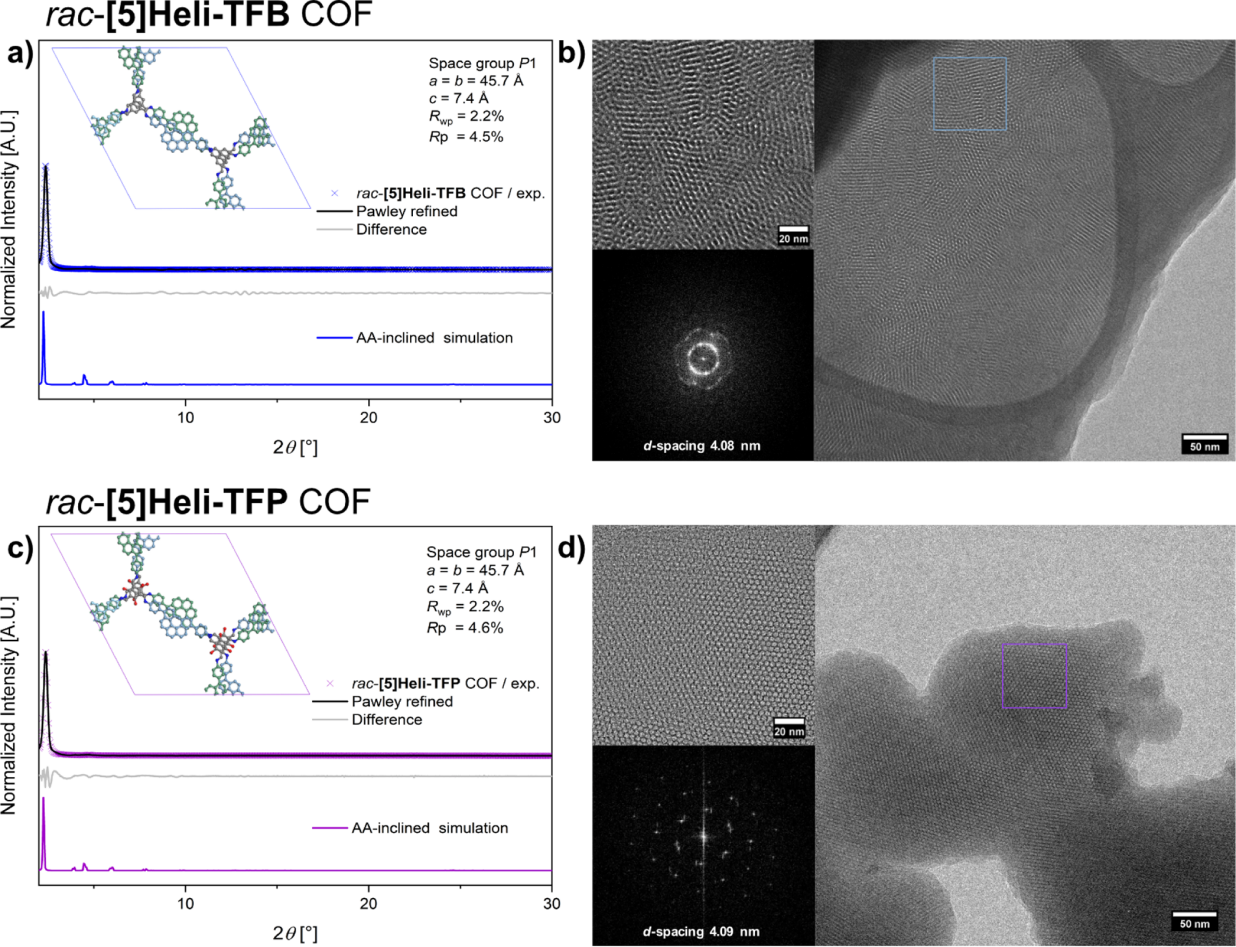
Characterization of *rac*-**[5]­Heli-TFB** COF and *rac*-**[5]­Heli-TFP** COF; (a) experimental (blue crosses), simulated PXRD patterns (blue line), Pawley refinement (black line) using AA-inclined stacking model, difference between Pawley refinement and AA-inclined simulation (gray) of *rac*-**[5]­Heli-TFB** COF; (b) low-dose cryo-HR-TEM of *rac*-**[5]­Heli-TFB** COF, inset: expansion of the blue square region showing hexagonal features along the [100] direction, and FFT of the region indicated; (c) experimental (purple crosses), simulated PXRD patterns (purple line), Pawley refinement using AA-stacking model (black line), difference between Pawley refinement and AA-inclined simulation (gray) of *rac*-**[5]­Heli-TFP** COF; (d) low-dose cryo-HR-TEM of *rac*-**[5]­Heli-TFP** COF, inset: expansion of the purple square region showing hexagonal features along the [100] direction, and FFT of the region indicated.

With the complete analysis of *rac*-**[5]­Heli-TFB** COF and optimized reaction conditions in hand, we aimed to test the enantiopure building blocks (*P*)-(−)-**3** and (*M*)-(+)-**3** as *pseudo*-*C*_2_ linkers, with TFB as a node to explore the formation of homochiral (*P*)- or (*M*)-**[5]­Heli-TFB** COFs, using experimental conditions identical to those for the racemic analogue. Despite rigorous repetitions, our attempts to obtain the homochiral version of *rac*-**[5]­Heli-TFB** COFs remained unsuccessful, yielding amorphous and nonporous solids (Figure S34). Possibly, the enantiopure [5]­helicenes induce too large deviations of the lattices, preventing the formation of crystalline packing motifs necessary to grow homochiral 2D lattices. These unexpected results highlight the complexity of the synthesis of homochiral 2D COFs using carbon-based π-conjugated building blocks.

Hence, we turned to TFP as a node for the synthesis of *rac*-**[5]­Heli-TFP** COF, testing (±)-**3** as a linker (Scheme 2). By using TFP, we anticipated that β-ketoamine linkages could stabilize the homochiral crystallization of **[5]­HeliCOFs** based on enantiopure *pseudo*-*C*_2_ linkers.^99,100^ Additionally, a modulation strategy based on the addition of aniline was applied.^101^ In this case, the *rac*-**[5]­Heli-TFP** COF was obtained under acidic solvothermal conditions, with 40 equiv. AcOH (6 M), 1.5 equiv of aniline, and *o*-DCB/*n*-BuOH 4:1 as the solvent at 120 °C for 72 h, achieving a high yield (96%, for the synthetic screening conditions see Table S2). Notably, the experimental PXRD pattern of *rac*-**[5]­Heli-TFP** COFs exhibits narrow reflexes at low 2θ angles of 2.5°, attributed to the (100) facet, and less intense reflexes at 4.2° and 8.5°, corresponding to the (200) and (120) planes, respectively (as shown in Figure 2c). The simulated diffraction pattern of the hexagonal AA-inclined stacking model agrees with the experimental PXRD diffractogram, and Pawley refinement provided good agreement with our proposed hexagonal AA-inclined stacking model (refined lattice parameters: *a* = *b* = 45.7 Å, and *c* = 7.4 Å, with *R*_wp_ = 2.2 and *R*_p_ = 4.6, and *P*1 as the space group, Figure S96).^93^ Similarly to the *rac*-**[5]­Heli-TFB** COF, we propose a symmetric hexagonal structural model in which (*P*)- and (*M*)-[5]­helicene are alternately stacked on top of each other over the 2D lattices (Figure 4a). In addition, low-dose HR-TEM of *rac*-**[5]­Heli-TFP** COF reveals honeycomb-type lattices (Figure 2d), with a *d*-spacing of 4.09 nm and FFT pattern showing a hexagonal phase projected along the (100) axis. These representative images are consistent with the PXRD analysis, indicating the presence of structural hexagonal AA-inclined 2D crystalline lattices for *rac*-**[5]­Heli-TFP** COF. Furthermore, N_2_-sorption measurements at 77 K of *rac*-**[5]­Heli-TFP** COF show two inflection points below *p*/*p*_0_ = 0.2 (Figure 3b), associated with mesoporous solids according to a type IV isotherm,^97^ with a significant BET surface area of 782 m^2^ g^–1^.^98^ The pore size distribution was estimated using NL-DFT, with a maximum of around 35.3 Å (Figure S56). Also, the thermal stability was confirmed to be up to 350 °C by TGA analysis (Figure S79). The FT-IR spectrum shows the characteristic C=O stretch band at 1582 cm^–1^ and C–N stretch band at 1285 cm^–1^, indicating the presence of ketoenamine linkages (Figure S84), in agreement with the CP-MAS NMR spectrum showing a peak at 185.2 ppm corresponding to the ketone carbon (Figure S87). Our results indicate that the *rac*-**[5]­Heli-TFP** COF is a crystalline and porous solid with hexagonal lattices.

**Figure 3:**
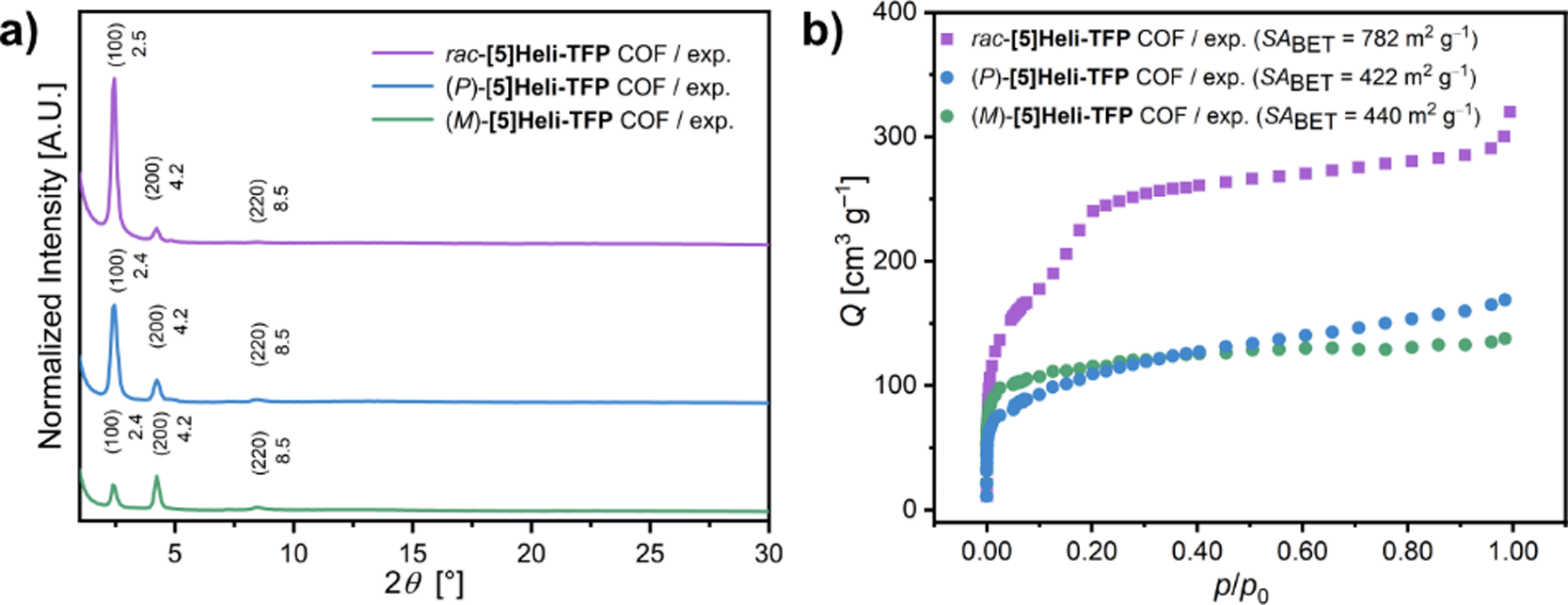
Properties of homochiral and racemic **[5]­Heli-TFP** COFs; (a) experimental PXRD diffractograms of *rac*-**[5]­Heli-TFP** COF (purple line), (*P*)-**[5]­Heli-TFP** COF (blue line), and (*M*)-**[5]­Heli-TFP** COF (green line); (b) N_2_ adsorption isotherms at 77 K of *rac*-**[5]­Heli-TFP** COF (purple squares), (*P*)-**[5]­Heli-TFP** COF (blue dots) and (*M*)-**[5]­Heli-TFP** COF (green dots).

In order to evaluate the potential steric limitations of β-ketoenamine linkages and *ortho*-fused aromatic rings, the [5]­helicene diimines without phenyl spacers (±)-**6** and (*R*,*R*)-(*P*/*M*)-**7** were tested as *pseudo*-*C*_2_ linkers with TFP for the targeted synthesis of *rac*-**[5]­Heli-TFP** COF-2 (Scheme S3). Despite multiple attempts (Tables S4 and S5), PXRD analysis of the resulting materials revealed noncrystalline morphologies (Figures S40 and S41). Similarly, testing the pure diastereomers (*R*,*R*)-(*P*)-(+)-**7** and (*R*,*R*)-(*M*)-(−)-**7** with TFP yielded amorphous materials (Figure S43). These results demonstrate that the intrinsic helical structure of [5]­helicene diimines excluding phenyl rings in the (4,11)-positions introduces geometric limitations that avoid the proper stacking of the [5]­helicenylene layers (Figure S131), leading to randomly oriented structures. This highlights the significant role of the phenyl spacer along with β-ketoenamine linkages.

### Synthesis of Homochiral 2D [5]­HeliCOFs

The enantiopure building blocks (*P*)-(−)-**3** and (*M*)-(+)-**3** were tested as *pseudo*-*C*_2_ linkers in combination with TFP as a node, using identical experimental conditions previously optimized for *rac*-**[5]­Heli-TFB** COF to obtain the homochiral analogues (*P*)-**[5]­Heli-TFP** COF and (*M*)-**[5]­Heli-TFP** COF (Scheme 2). Interestingly, the experimental PXRD diffractograms of *rac*-**[5]­Heli-TFP** COF, and homochiral (*P*)- and (*M*)-**[5]­Heli-TFP** COF display similar angular positions of Bragg reflexes but their relative intensities are distinct from each other. In particular, the PXRD patterns of the homochiral samples display an intense reflex at 2θ = 4.2°, corresponding to the (2 0 0) facet (Figure 3a). In contrast, the racemic sample shows a significantly more intense reflex at 2θ = 2.5°, attributed to the (1 0 0) facet (Figure 3a). These differences suggest a higher structural offset between the crystalline lattices in the homochiral analogues.^102,103^ Consequently, we propose that homochiral (*P*)- and (*M*)-**[5]­HeliTFP** COFs predominantly adopt an asymmetric hexagonal structure, characterized by an equal statistical distribution of AA-inclined:AB-staggered (AA_i_:AB)^stat^ stacking modes (Figure 4d).^104^ These multilayer statistical representations with interlayer shifts have been proposed for isoenergetic ensembles in 2D COFs.^105–108^ In this case, the multilayer model (AA_inc_:AB)^stat^ shows the best agreement with the experimental PXRD diffractograms for both homochiral (*P*)- and (*M*)-**[5]­Heli-TFP** COFs (Figures S97–S102). In contrast, other hexagonal single-stacking modes exhibited a prominent main reflex at 2θ = 2.4°, which did not match the experimental PXRD patterns (Tables S12 and S13).

**Figure 4:**
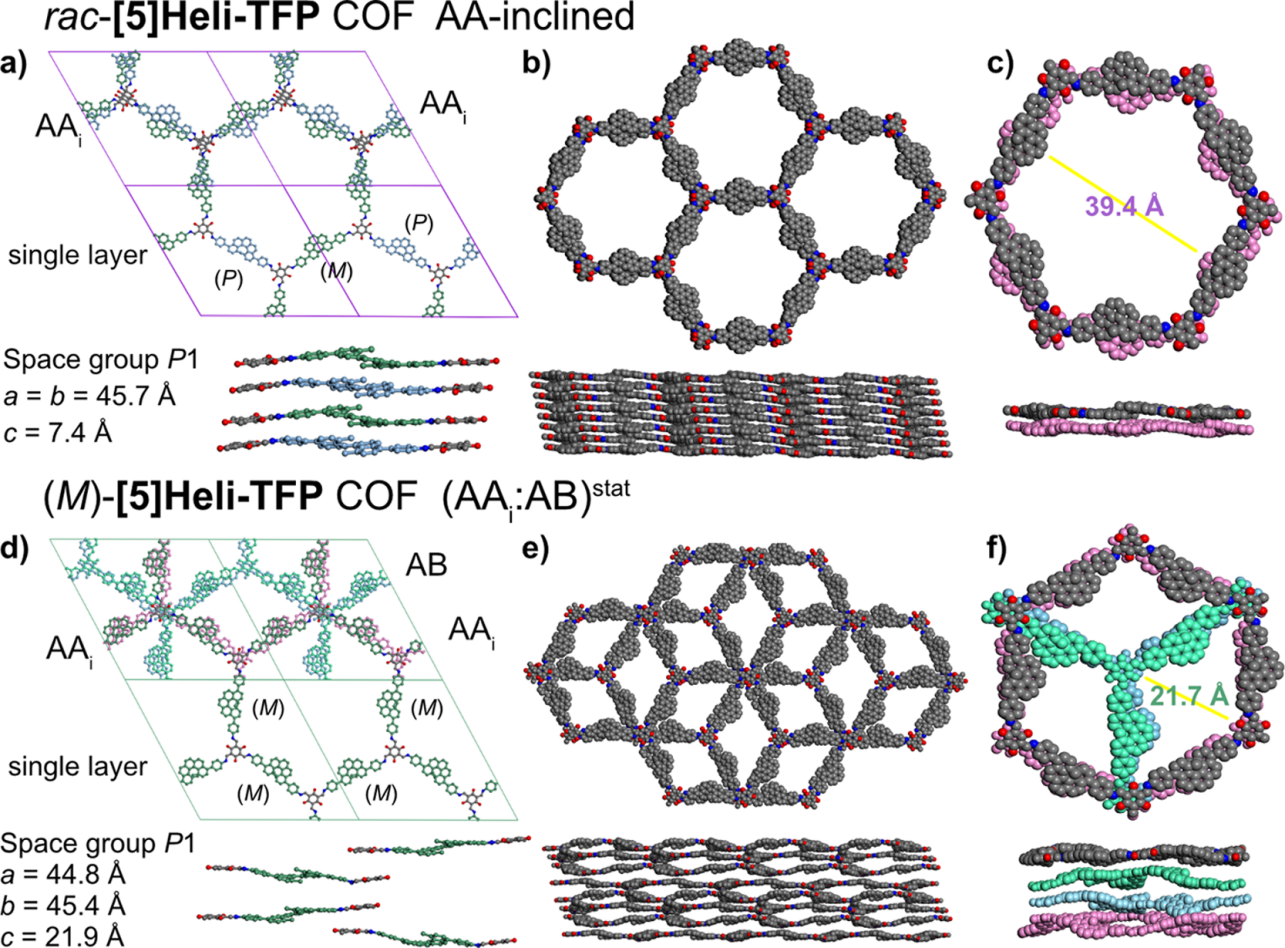
Proposed structural representations of racemic and homochiral **[5]­Heli-TFP** COFs; (a) Pawley-refined structural model of *rac*-**[5]­Heli-TFP** COF with AA-inclined stacking mode (top: complete unit cells, bottom: single layer); (b) top and side views on the corresponding refined structure; (c) example of single hexagonal pore as double-layer extracted from the refined model. (d) Pawley refinement asymmetric structural model of (*M*)-**[5]­Heli-TFP** COF with multilayer statistical representation (AA_i_:AB)^stat^, illustrating the asymmetric stacking of (*M*)-[5]­helicene linkers across the repetitive layers (top: complete unit, bottom: single layer); (e) top and side views of the corresponding statistical model (AA_i_:AB)^stat^; (f) example of single hexagonal pore extracted from the structural model; the homochiral (*P*)-**[5]­Heli-TFP** COF can be also described with statistical structural model (AA_i_:AB)^stat^ (see Figure S101).

In our proposed statistical model (AA_i_:AB)^stat^, the ideal columnar packing of enantiopure [5]­helicene units with their helical axes parallel^95,96^ may generate local dipole moments perpendicular to the *c*-axis, disturbing the ideal AA_i_ stacking mode. Consequently, adjacent homochiral layers avoid their repulsion by adopting a structurally alternating AB-staggered stacking mode, with enantiopure [5]­helicene cores irregularly shifted on top of each other (Figure 4d). Our suggested multilayer representation (AA_i_:AB)^stat^ decreases the proximity of enantiopure [5]­helicene cores and minimizes steric hindrance between the homochiral layers, leading to a reduced packing density (Figure 4e,f). Homochiral **[5]­Heli-TFP** COFs form less compact asymmetric lattices with a lower degree of crystallinity than their racemic counterparts, in agreement with the Liebisch–Wallach rule.^59^

The distinct hexagonal structural models for homochiral and racemic **[5]­Heli-TFP** COFs should also lead to differences in the physical properties of the solids. For instance, entirely different types of inherent porosity are expected from crystalline lattices with predominantly symmetric hexagonal AA stacking mode than more distorted lattices with AB stacking mode.^109–111^ Interestingly, the N_2_ sorption measurements from the homochiral (*P*)- and (*M*)-**[5]­Heli-TFP** COFs exhibited a single step below *p*/*p*_0_ = 0.1, associated with microporous materials according to a type I isotherm (Figure 3b).^97^ Specifically, the (*P*)-**[5]­Heli-TFP** COF has a porosity value of SA_BET_ = 422 m^2^ g^–1^ (Figure S46)^98^ and a pore size distribution estimated at around 17.4 Å (Figure S57). Nearly identically, the (*M*)-**[5]­Heli-TFP** COF shows a porosity value of SA_BET_ = 440 m^2^ g^–1^ (Figure S47),^98^ with a pore size distribution estimated at around 16.1 Å (Figure S58). In contrast, the *rac*-**[5]­Heli-TFP** COF shows significantly higher N_2_ uptake and an isotherm feature with two inflection points (Figure 3b). The first inflection point corresponds to the initial N_2_ filling of the pores (below *p*/*p*_0_ = 0.1), while the second point (around *p*/*p*_0_ = 0.19) reflects N_2_ condensation into its larger pores (pore size distribution of 35.3 nm). These porosity differences between racemic and homochiral samples are in agreement with their proposed stacking models, where highly symmetric hexagonal lattices with an AA-inclined stacking mode generally have greater porosity values compared to less-ordered lattices with an AB stacking mode.^109–111^ Such significant porosity variations have not been reported in homochiral and racemic 2D COFs and are considered a direct consequence of introducing helical chirality into the backbone of all-organic, highly ordered 2D frameworks.

Subsequently, a comparison of the morphology and features of the homochiral and racemic **[5]­Heli-TFP** COFs was performed. In this case, low-dose cryo-HR-TEM of exfoliated homochiral samples revealed regular lattice fringes of around 2.4 nm spacing (most likely viewed across the pore channels; Figure 5, top). Using Bragg’s equation,^112^ the visualized lattice fringes can be associated with their relatively intense reflex in PXRD at 2θ = 4.2°, confirming the crystalline nature of homochiral 2D **[5]­Heli-TFP** COFs. Furthermore, the physical features of homochiral and racemic **[5]­Heli-TFP** COFs were compared by FE-SEM micrographs (Figure 5, bottom). The homochiral samples showed homogeneous particles similar to each other (average lengths of 0.9 and 0.7 μm, respectively), with a pointed star-shape and spike-like surface. In contrast, the *rac*-**[5]­Heli-TFP** COF displays mesh-type conglomerates with variable elongations and rounded features with a soft-like surface (average rod diameter = 2.7 μm). In contrast to these observations, the elemental analysis of both homochiral and racemic **[5]­Heli-TFP** COFs reveals an almost identical chemical composition (Table S10). Further exposure to base, acid, and organic solvents showed no changes in the corresponding PXRD patterns (Figures S132 and S133). Therefore, our results suggest that the structural and physical properties of homochiral and racemic **[5]­Heli-TFP** COFs are mainly directed by the absolute configuration of their respective enantiopure or racemic [5]­helicene-based building blocks.

**Figure 5:**
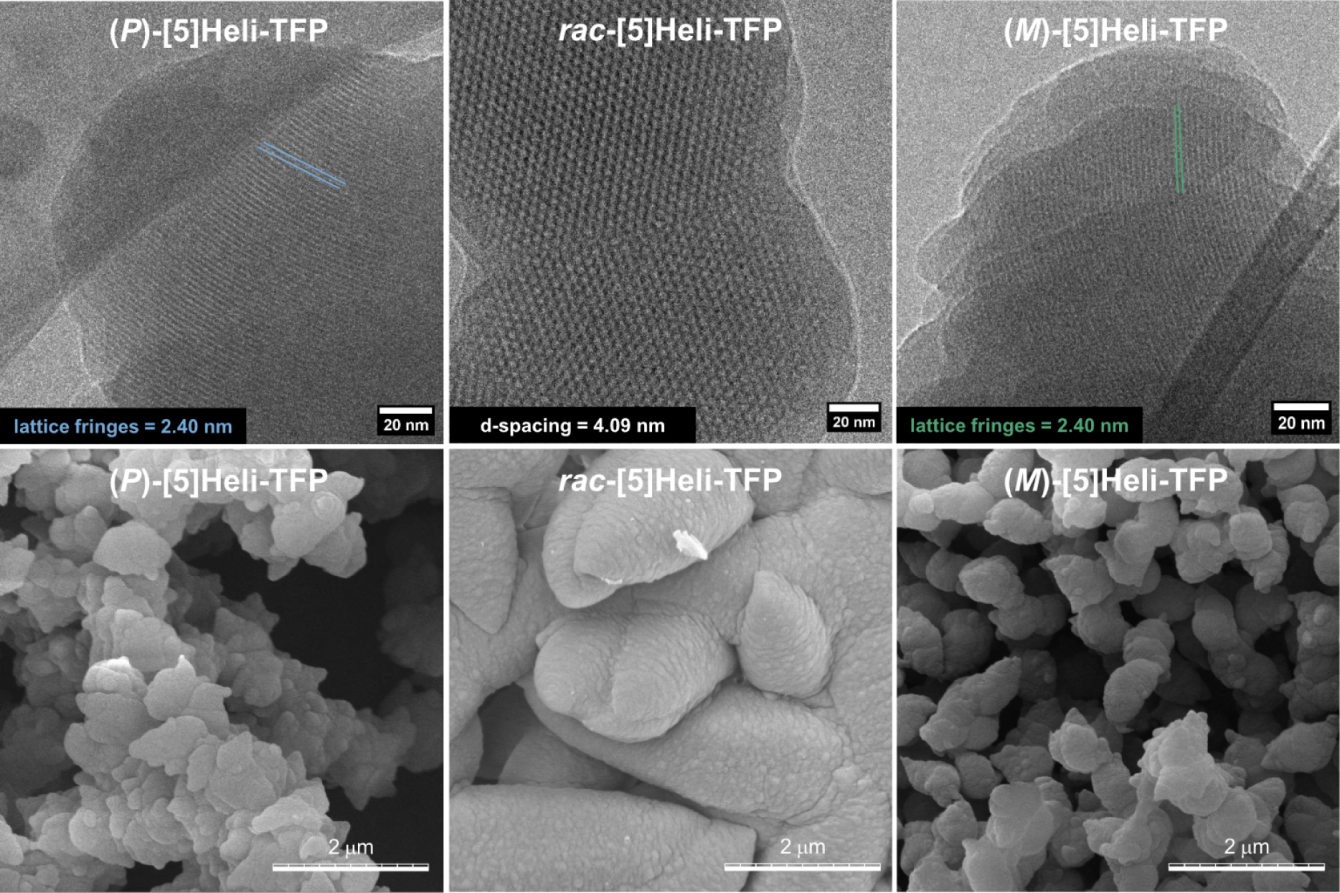
Comparison of the morphology from the homochiral and racemic **[5]­Heli-TFP** COF powders; (top) low-dose cryo-HR-TEM images at 77 K of (*P*)-**[5]­Heli-TFP** COF, *rac*-**[5]­Heli-TFP** COF, and (*M*)-**[5]­Heli-TFP** COF; (bottom) corresponding FE-SEM micrographs of **[5]­Heli-TFP** COF samples at 298 K, showing different features and size particles between each other.

To confirm the structural differences between homochiral and racemic **[5]­Heli-TFP** COFs, their chiroptical properties were analyzed. As the main initial comparison, the ECD spectra of the enantiopure building blocks (*P*)-(−)-**3** and (*M*)-(+)-**3** exhibited mirror-imaged Cotton effects in the UV–vis range (250–450 nm, Figure 6, dashed lines). Interestingly, the averaged ECD spectra for the homochiral (*P*)- and (*M*)-**[5]­Heli-TFP** COFs showed an intense first Cotton effect in the visible region from 470 nm up to ca. 800 nm (Figure 6, top). Specifically, the (*P*)-**[5]­Heli-TFP** COF displayed a first positive Cotton effect at 504 nm and a second negative Cotton effect at 406 nm. Satisfyingly, the homochiral analogue (*M*)-**[5]­Heli-TFP** COF showed the opposite signed ECD pattern with a first negative Cotton effect at 502 nm and a second intense positive Cotton effect at 411 nm. In this case, the observed ECD bands below 470 nm can be associated with the inherent helical chirality from the corresponding enantiopure [5]­helicene *pseudo*-*C*_2_ linkers, supported by TD-DFT simulations of a small (*P*)-**[5]­Heli-TFP** COF fragment (Figure S117).^113^ In contrast, the broad ECD signals exceeding 800 nm arise from the extended helical arrangement in the homochiral 2D lattices, as a result of delocalized exciton formation over the asymmetric conjugated lattices.^114,115^ As expected, the *rac*-[**5]­Heli-TFP** COF remains chiroptically silent, confirming its racemic composition. For further comparison, the ECD spectra of the noncrystalline (*P*)- and (*M*)-**[5]­Heli-TFB** polymeric networks exhibit low degree of optical ellipticity (Figure 6, bottom), with a Cotton effect resembling that observed for the corresponding enantiopure building blocks (*P*)-(−)-**3** and (*M*)-(+)-**3**, only limited to the UV–vis region (<450 nm). These results confirm that incorporating enantiopure helical building blocks into amorphous solids induces only local chirality. In contrast, the crystalline and homochiral **[5]­HeliCOFs** exhibited broad ECD spectral features, reaching the far-red UV–vis spectrum, with high dissymmetry factor (*g*_abs_) values for (*M*)-**[5]­Heli-TFP** COFs of 3.5 × 10^–2^ at λ = 504 nm and for (*P*)-**[5]­Heli-TFP** COFs 9.2 × 10^–3^ at λ = 507 nm, ranking among the highest *g*_abs_ values in the visible region of the spectrum for 2D homochiral COFs (Table S149). Highlighting the importance of helical chirality with new opportunities for 2D chiral architectures in all-organic materials.

**Figure 6:**
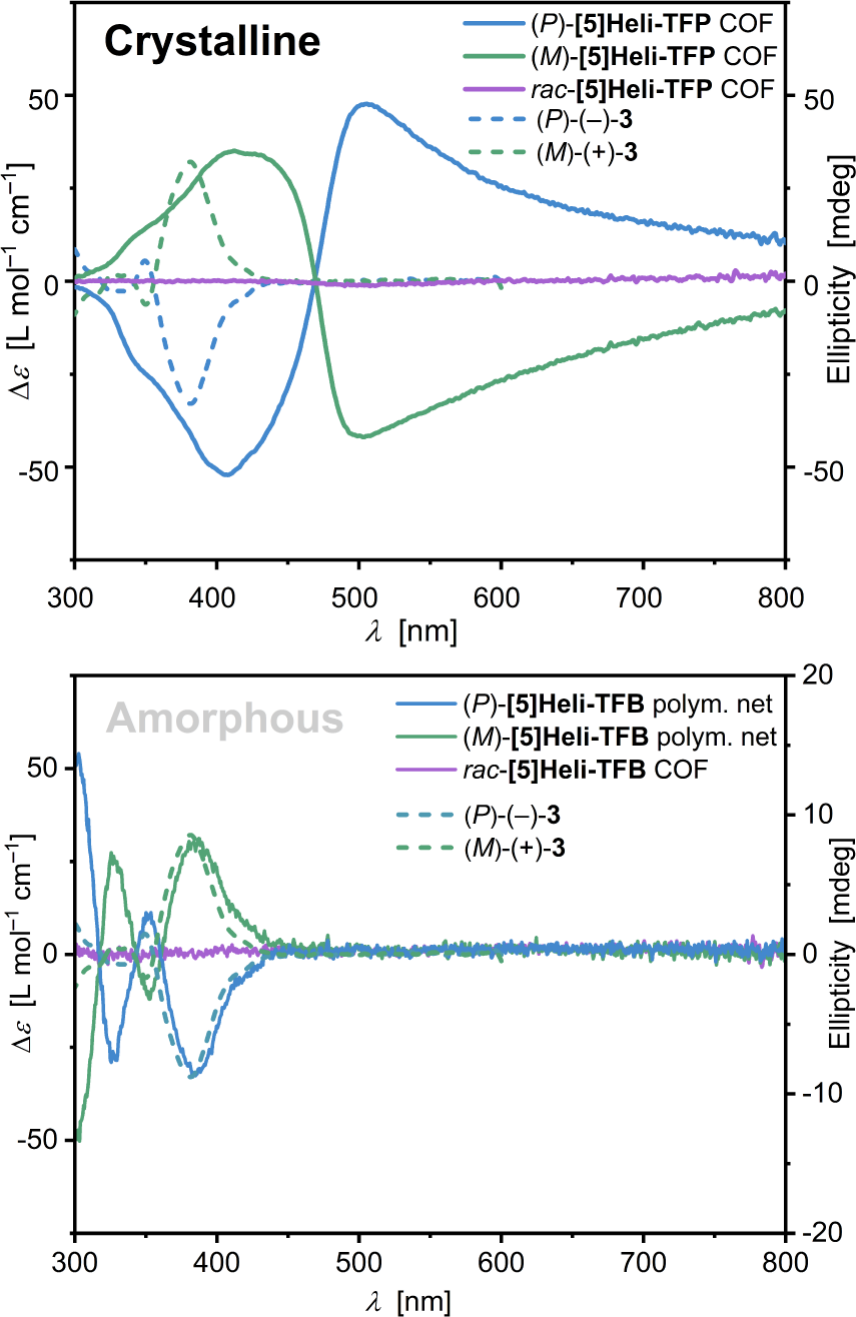
Chiroptical properties of homochiral and racemic **[5]­Heli-TFP** COFs and **[5]­Heli-TFB** polymeric networks; (top) ECD spectra of building blocks (*P*)-(−)-**3** and (*M*)-(+)-**3** in CH_2_Cl_2_ (*c* = 1.1 × 10^–6^ M at 298 K, dashed traces – Δ*ε* ordinate); average ECD spectra of *rac*-**[5]­Heli-TFP** COF, (*P*)- and (*M*)-**[5]­Heli-TFP** COFs (*c ≈* 0.08 g L^– 1^) as DMSO dispersions, averaged over different rotation angles at 298 K (solid traces – ellipticity ordinate; see also Figures S113–115); (bottom), average ECD spectra of *rac*-**[5]­Heli-TFB** COF, (*P*)-**[5]­Heli-TFB** polymeric net, and (*M*)-**[5]­Heli-TFP** polymeric net (*c ≈* 0.16 mg mL^–1^) as DMSO dispersions, averaged over different rotation angles at 298 K (solid traces – ellipticity ordinate).

Moreover, homogeneous thin films of **[5]­Heli-TFP** COFs on plasma-activated quartz substrates could be readily fabricated by using an optimized diluted concentration of building blocks (Figure S36 and Table S3). These COF films show homogeneous topographical aggregation over the substrate (Figure 7a), with an average thickness of 240 nm for the homochiral sample and 282 nm for the racemic film, allowing high control over the crystallization process.^116^ Such homogeneous films of **[5]­Heli-TFP** COFs are essential to integrate their outstanding chiroptical properties into future advanced chiral devices.^57,117^ Using the *in situ* deposition, both homochiral and racemic films exhibited crystalline reflexes in PXRD at low 2θ angles (Figure S39). Compared to their bulk powder counterparts, the films exhibited Bragg reflexes shifted by 0.3° at higher 2θ positions due to COF-substrate interactions. Additionally, the porosity of the films was measured with N_2_ sorption at 77 K (Figure S39), where the homochiral and racemic films exhibited different BET surface area values and distinct isothermal features with each other. The observed differences in crystallinity and porosity between homochiral and racemic films are consistent with their powder version and confirm the direct solvothermal deposition of highly ordered and porous **[5]­Heli-TFP** COF films. In general, the physical appearance of homochiral and racemic **[5]­Heli-TFP** COF films differs from each other (Figure 7a, inset), supporting their distinct structural features as in the bulk powders.

**Figure 7:**
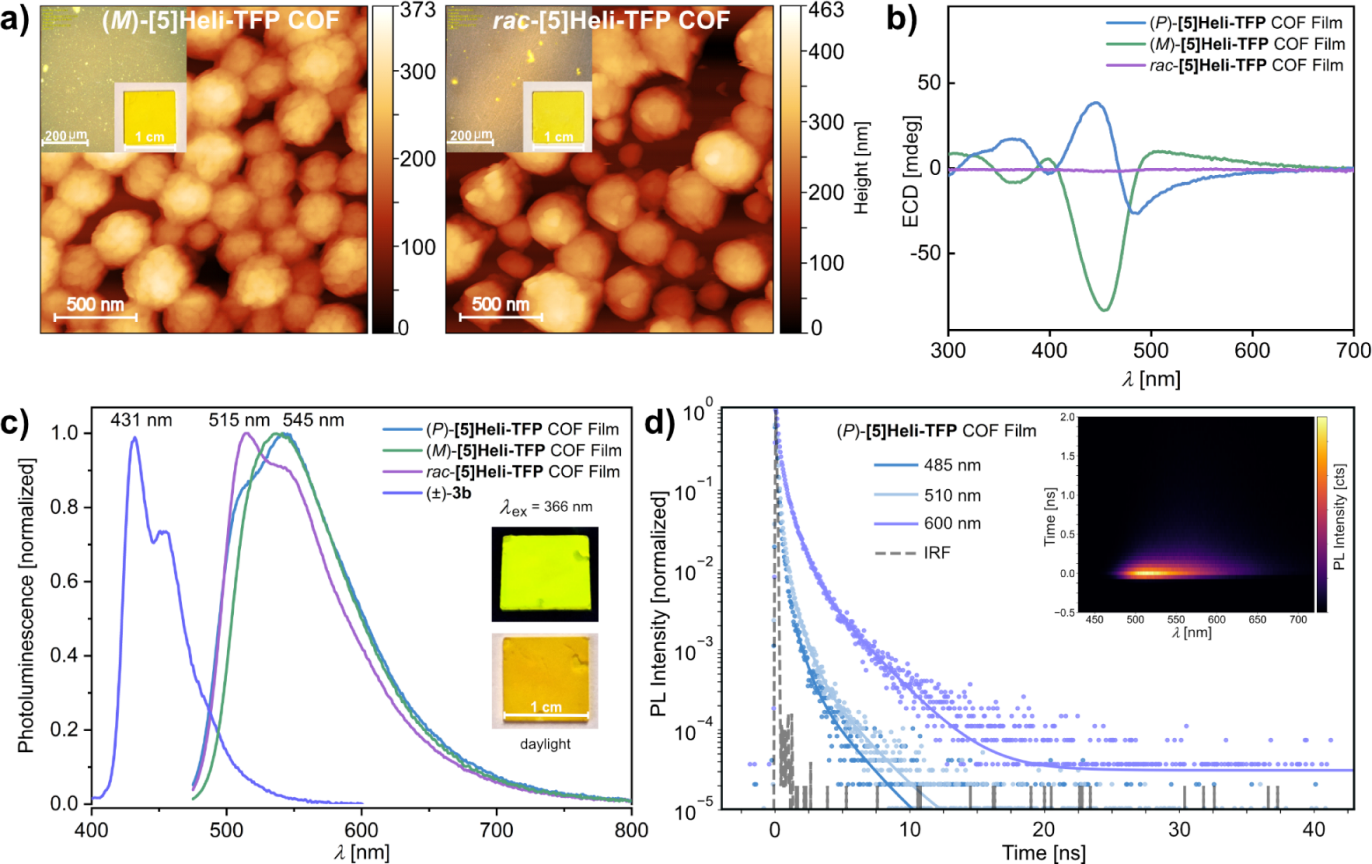
Characterization of racemic and homochiral **[5]­Heli-TPF COF** films over quartz substrates; (a) AFM images of **[5]­Heli-TFP** COF films using 1.2 mM of building blocks (inset = optical microscope images and photographs of the complete films); (b) ECD spectra of **[5]­Heli-TFP** COF films averaged over different rotation angles at 298 K (see also Figures S122–S124); (c) steady-state photoluminescence (PL) spectra of **[5]­Heli-TFP** COF films (λ_ex_ = 460 nm) and building block (±)-**3b** (λ_ex_ = 380 nm) at 298 K; (d) transient PL traces of (*P*)-**[5]­Heli-TFP** COF film at 298 K (λ_ex_ = 343 nm). . ^,^

In parallel, the averaged ECD spectra of both homochiral **[5]­Heli-TFP** COF films reveal extended opposite-signed Cotton effects in the range of 300–700 nm (Figure 7b). The ECD response remains largely unchanged upon rotation and flipping of the samples (Figures S122–S124), confirming that linear dichroism or linear birefringence does not significantly contribute to the observed ECD signals.^117,118^ The observed multiple ellipticity from 300 to 400 nm can be associated with the intrinsic helical chirality from the enantiopure [5]­helicene building blocks.^113^ Meanwhile complex ECD features at >400 nm are related to the exciton coupling from the π-conjugated 2D frameworks.^114,115^ These chiroptical features exhibit greater complexity compared with the previously observed bisignate Cotton effect of the COF powders. The observed extended ellipticity is not perfectly symmetric, as is common for chiral π-conjugated polymeric films due to their complex deposition process.^117^

The photophysical emission properties of powders of **[5]­Heli-TFP COFs** are significantly different from those of their monomeric building blocks (Figure S134). However, studying the photoluminescence (PL) of suspended powders was challenging due to their aggregation in organic solvents (Figure S137). In contrast, the homogeneous **[5]­Heli-TFP** COF films on quartz substrates disclose a symmetric emissive property of these π-conjugated solids, with the emission centered around the green-yellow region (500–550 nm), highlighting the advantages of studying these materials as films (Figure 7c). Specifically, the homochiral (*P*)-**[5]­Heli-TFP** COF film exhibits a Stokes-shifted emission (λ_em,max_ = 545 nm), red-shifted by 30 nm (0.13 eV) compared to the *rac*-**[5]­Heli-TFP** COF film (λ_em,max_ = 515 nm). This distinct PL spectrum between homochiral and racemic films supports their nonidentical structural packing. No detectable CPL was observed from the homochiral film (Figure S144), as may be expected given that the emission does not originate predominantly from the (most twisted) helicene structural subunit, while PL quantum yields (Φ_f_) for the [5]­helicene are also relatively low, yielding *rac*-**[5]­Heli-TFP** COF Φ_f_ = 0.6 ± 0.1%, (*P*)-**[5]­Heli-TFP** COF Φ_f_ = 3.4 ± 0.4%, and (*M*)-**[5]­Heli-TFP** COF Φ_f_ = 2.6 ± 0.4%.^119^ Time-resolved PL studies confirm a <1 ns effective PL lifetime decaying with multiexponential kinetics (Figure 7d). This behavior suggests energy funneling within the framework – resulting in faster decay at higher energy positions observed and nonradiative losses (explaining the observed low PLQY). Future synthetic modifications to the [5]­helicene core are planned to improve the emissivity of these 2D COFs.

## Conclusion

In summary, we synthesized both homochiral and racemic 2D COFs from enantiopure and racemic [5]­helicene derivatives by introducing helical chirality into the backbone of all-organic homochiral 2D COFs. Our findings confirm that the absolute stereochemistry directs the structural characteristics and properties of crystallized carbon-based lattices between homochiral and racemic 2D COFs. Specifically, racemic [5]­helicene linkers promote the formation of highly crystalline and porous 2D frameworks with a predominant AA-inclined stacking mode, attributed to the locally dense packing of racemic motifs, such as *rac*-**[5]­Heli-TFB** COF and *rac*-**[5]­Heli-TFP** COF. In contrast, enantiopure [5]­helicene building blocks yield an asymmetric hexagonal structure, mainly represented by a multilayer model (AA_i_:AB)^stat^ with a lower degree of crystallinity and reduced accessible porosity compared with their racemic counterparts. These results are reproducible as powders and films. Our observations mark the expansion of the Liebisch–Wallach rule to all-organic 2D frameworks, demonstrating that the absolute stereochemical composition of carbon-based building blocks can significantly influence the stacking interaction of crystalline and porous 2D lattices. As powder, the homochiral (*P*)- and (*M*)-**[5]­Heli-TFP** COFs exhibited extended Cotton effects, reaching beyond 800 nm, attributed to the propagation of helical chirality from [5]­helicene into the backbone of the 2D frameworks.

Future work will aim at improving the chiral light emission properties of such homochiral **[5]­HeliCOFs** using highly emissive building blocks and connectivity strategies to enhance the global twisting of the network structure beyond the incorporation of only local chiral units. We expect a broad applicability of our findings for the design of crystalline and porous 2D homochiral organic powders and films, with future applications ranging from spin-optoelectronics to sensing and enantioselective catalysis in confined spaces.

## Supplementary Material



## Notes

Detailed information on experimental methods, materials used, synthetic characterization, calculation data, and atomic coordinates of the frameworks (PDF)

## ¶

Department of Chemistry, University of Cologne, Greinstr. 4, 50939 Köln, Germany, odumele@uni-koeln.de
