# Supplementary material for: Homochiral versus Racemic 2D Covalent Organic Frameworks
Source: J Am Chem Soc. 2025 May 15;147(21):17750–63. doi: 10.1021/jacs.5c01004 (PMC12123627; doi:10.1021/jacs.5c01004)
Supplement: Supplementary file 1 [file ja5c01004_si_001.pdf]

Supporting information for:

Homochiral versus Racemic 2D Covalent Organic Frameworks

José del Refugio Monroy,[1,2] Tejas Deshpande,[3] Joël Schlecht,[2] Clara Douglas,[2] Robbie Stirling,[1] Niklas Grabicki,[1] Glen J. Smales,[4] Zdravko Kochovski,[5] Filippo Giovanni Fabozzi,[1] Stefan Hecht,[1] Sascha Feldmann,[3] and Oliver Dumele[2,6,7]*

1) Department of Chemistry & Center for the Science of Materials Berlin, Humboldt-Universität zu Berlin, Brook-Taylor-Strasse 2, 12489 Berlin, Germany.

2) Institute of Organic Chemistry, Albert-Ludwigs-Universität Freiburg, Albertstrasse 21, 79104 Freiburg, Germany.

3) Institute of Chemical Sciences and Engineering, École Polytechnique Fédérale de Lausanne, Rue de l'Industrie 17, 1951 Sion, Switzerland.

4) Bundesanstalt für Materialforschung und -prüfung, Unter den Eichen 87, 12205 Berlin, Germany.

5) Institute of Electrochemical Energy Storage, Helmholtz-Zentrum Berlin für Materialien und Energie, Hahn-Meitner-Platz 1, 14109 Berlin, Germany.

6) Freiburg Materials Research Center, Albert-Ludwigs-Universität Freiburg, Stefan-Meier-Strasse 21, 79104 Freiburg, Germany.

7) Freiburg Center for Interactive Materials and Bioinspired Technologies, Albert-Ludwigs-Universität Freiburg, Georges-Köhler-Allee 105, 79110 Freiburg, Germany.

***Corresponding Author**

E-mail: oliver.dumele@oc.uni-freiburg.de

Current address:
Department of Chemistry, University of Cologne, Greinstr. 4, 50939 Köln, Germany
odumele@uni-koeln.de

Table of Contents

S1. General Experimental Details 3
S2. Synthetic Procedures 11
S3. Selected NMR spectra. 23
S4. Synthesis of COFs and POPs 39
S5. Nitrogen adsorption isotherms 50
S6. Pore Size Distribution 61
S7. High-Resolution Transmission Electron Microscopy 63
S8. Scanning Electron Microscopy 75
S9. Atomic Force Microscopy 79
S10. Elemental Analysis 80
S11. Thermal Gravimetric Analysis 81
S12. UV-Vis Spectroscopy 82
S13. FT-IR Spectroscopy 84
S14. CP-MAS NMR Analysis 86
S15. PXRD and Structure Modelling of COF materials 88
    S15.1 Racemic **[5]Heli-TFB** and **[5]Heli-TFP COF**s 88
    S15.2 Homochiral HeliCOFs 94
S16. Diastereomer Separation 102
S17. Electronic Circular Dichroism spectroscopy 106
S18. Crystallographic Data 120
S19. DFT calculations 131
S20. Stability of **[5]Heli-TPF COF**s 132
S21. Photoluminescence spectroscopy 133
S22. Cartesian coordinates 141
S23. References 148

S1. General Experimental Details

Chemical reagents and solvents. Unless otherwise specified, the reagents were purchased from different suppliers (abcr, BLDpharm, Fluorochem, Sigma-Aldrich, and TCI), and used as received, without further treatment. Solvents were dried using a Solvent Purification System from PURESOLV, stored over molecular sieves 3–4 Å, and degassed prior to use by 3 freeze–pump–thaw cycles. The starting materials 1,4-bis(2-bromo-5-methylstyryl)benzene and 4-bromo-*N*-(diphenylmethylidene)aniline were obtained according previously reported procedures.[1,2]

General Reactions. Unless otherwise noted, reactions were performed in flame-dried glassware fitted with rubber septa under a positive pressure of argon. Air- and moisture-sensitive liquids were transferred via syringe or stainless-steel cannula through rubber septa. Solids were added under inert gas counter flow or were dissolved in appropriate solvents. Unless otherwise specified, reaction temperatures above 23 °C were conducted in a heated oil bath. The reactions were magnetically stirred, and monitored by analytical thin-layer chromatography (TLC), UPLC-MS traces or ^{1}H NMR spectra. The reported yields (%) are for compounds that were dried under vacuum (10^{-2} mbar) before analytical characterization.

Thin-layer chromatography (TLC). The experiments were conducted using on aluminum plates precoated with silica gel (SiO_2, 0.25 mm, 60 Å pore size) purchased from Merck & co, impregnated with a fluorescent indicator (F254 nm). TLC plates were visualized by exposure to ultraviolet light (UV) at 254 or 366 nm.

Automated-Medium Pressure Column Chromatography (MPLC). Unless otherwise noted, the products were purified using a CombiFlashRf 300 system from Teledyne ISCO, with 200 mL min^{-1} max flow, and 200 psi max, equipped with integrated ELSD and (200–800) nm UV-Vis variable wavelength detector.

Nuclear Magnetic resonance (NMR). The experiments were recorded using a Bruker Avance II 300 (300 MHz for ^{1}H and 75 MHz for ^{13}C), Bruker Avance 400 (400 MHz for ^{1}H and 101 MHz for ^{13}C), and a Bruker Avance III 500 (500 MHz for ^{1}H and 126 MHz for ^{13}C) at 298 K. Proton chemical shifts are expressed in parts per million (ppm, δ scale, assigned proton atom), and referenced to residual proton in the NMR solvent ($CDCl_3$: δ_H = 7.26 ppm, CD_2Cl_2: δ_H = 5.32 ppm). Carbon chemical shifts are expressed

in parts per million (ppm, δ scale, assigned carbon atom) and are referenced to the carbon resonance of the NMR solvent ($CDCl_3$: δ_C = 77.16 ppm, CD_2Cl_2: δ_C = 54.00 ppm).[3] ^1H NMR spectroscopic data are reported as follows: Chemical shift in ppm (multiplicity, coupling constants *J* (Hz), integration intensity, assigned proton). The resonance multiplicities are abbreviated with s (singlet), d (doublet), t (triplet), q (quartet), quint. (quintet), sept. (septet), m (multiplet), and br. (broad). In the case of combined multiplicities, the multiplicity with the larger coupling constant is stated first. The chemical shift of all signals, as well for centrosymmetric multiplets, is reported as the center of the resonance range. In addition, to ^1H and ^{13}C NMR measurements, 2D NMR techniques such as homonuclear correlation spectroscopy (COSY), heteronuclear single quantum coherence (HSQC) and heteronuclear multiple bond coherence (HMBC) were used to assist signal assignment. Coupling constants *J* are reported in Hz. All raw fid files were processed and the spectra analyzed using the software MestReNova 12.0.2 from Mestrelab Research S. L.

Ultra-high-performance liquid-chromatography coupled to Mass Spectrometry (UPLC–MS). The experiments were recorded using a Waters UPLC Acquity with a Waters Alliance System (gradient mixture acetonitrile/water). The Waters systems consisted of a Waters Separations Module 2695, Waters Diode Array Detector 996, and Waters Mass Detector ZQ 2000.

High-resolution mass spectrometry. The experiments were performed on a MALDI-TOF Autoflex Max (Bruker Daltonik, Bremen), 355 nm laser, 4000 shots accumulated on 2 different spots, reflector modus, 20 mg mL^{-1} in $CHCl_3$ with 1 mg potassium trifluoroacetate. For MALDI measurements, the matrix was *trans*-2-[3-(4-*tert*-butylphenyl)-2-methyl-2-propenylidene] (DCBT). The masses are reported in *m*/*z* units as the molecule ion $[M]^+$.

Single-crystal X-ray crystallography. The data were collected with a BRUKER D8 VENTURE area detector with Mo-K_α radiation (λ = 0.71073 Å) or Cu-K_α(λ = 1.54178 Å). The data were corrected with a multi-scan absorption corrections in SADABS[4]. The resulting structures were solved by intrinsic phasing method (SHELXT-2013)[5], and refined using full matrix least square procedures based on F2 with all measured reflections (SHELXL-2014)[6] in the graphical user interface (SHELXLe)[7] with anisotropic temperature factors for all non-hydrogen atoms. All hydrogen atoms were

added geometrically and refined by using a riding model. Finally, the calculation of the voids in the structure was achieved with the Platon Program.[8]

Fourier-transform infrared spectroscopy (FT-IR). The experiments were collected on an Agilent technologies Cary 630 FT-IR spectrometer equipped with a diamond ATR attachment. The spectra were measured between 640–4000 cm^{-1}. The absorption bands are represented are reported in wavenumbers (cm^{-1}). The relative intensity of the absorption bands is described as (*vs* = very strong, *s* = strong, *m* = medium, *w* = weak, *br* = broad). The spectra are displayed with a baseline correction.

Electron circular dichroism (ECD). The spectra were recorded in a 1.00 mm path length cuvette using a JASCO J-1500 spectrometer. Wavelengths are reported in nanometers (nm). The spectra were measured between 250–600 nm, data points were collected in 0.1 nm interval steps.

Specific Rotation was measured on a A. Krüss Optronic P8000-T at the given temperature T at the Na D line (λ = 598 nm) in a 10 cm (l = 1; equivalent to 1 dm) path length cell with solutions at ca. 1.33 mM. The given units of concentration "c" is in [g/100 mL]. The specific rotation is calculated according to $[\alpha]_\lambda^T = \frac{100 \times \alpha}{l \times c}$, whereas α is the measured optical rotation value. The reported values derive from at least six measurements of the same solution (or two duplicated solution).

Small-/wide-angle X-ray scattering (SAXS/WAXS) (from now referred as PXRD). The experiments were performed using the MOUSE (Methodology Optimization for Ultrafine Structure Exploration) at BAM.[9] X-rays were obtained from a microfocus X-ray tube, followed by multilayer optics to parallelize, and monochromatize the X-ray beams to wavelengths of Cu Kα (λ = 0.154 Å). Scattered radiation was detected on an *in-vacuum* Eiger 1M detector (Dectris, Switzerland), which was placed at multiple distances between 55–2307 mm from the COF material. The obtained data has been processed using the DAWN software package in a standardized complete 2D correction pipeline with uncertainty propagation.[10] The experimental diffractogram patterns were fitted using Pawley refinement to computational COF models using Accelrys' Materials Studio program package. The structural models were optimized using the Forcite module with an 'ultra-fine' optimization with Universal Force Field and 'Smart' algorithm.[11]

Powder X-ray diffraction (PXRD). The experiments were performed using the benchtop Miniflex 600 diffractometer, X-rays were obtained from a Cu *K*α line focused radiation (λ = 0.15416 nm, 40 kV, 15 mA, 600 W), using a low-background silicon sample holder. The data were collected in a range of 2.0° < 2θ < 50°. and using a 0.01° 2θ step at a rate of 2.0° per min. The experimental diffractogram patterns were fitted using Pawley refinement to computational COF models using Accelrys' Materials Studio program package. The structural models were optimized using the Forcite module with an 'ultra-fine' optimization with Universal Force Field and 'Smart' algorithm.[11]

Nitrogen adsorption/desorption isotherms. The measurements were recorded using a Microtrac Belsorp Max Surface Area and Porosity Analyzer at 77 K. The materials were transferred to dried, and tared analysis tubes equipped with filler rods, and capped with a seal frit. Before analysis, samples were degassed at 313 K for 1 h, and at 393 K for 10 h. After cooling at 298 K, the tubes were weighted again to determine the mass of the activated samples (10–30 mg). Nitrogen gas (N_2, 99.999% purity) from Praxair was used for all sorption measurements. The adsorption isotherm was measured from $p/p_0 = 10^{-6}$ to $p/p_0 = 0.995$. The desorption isotherm was measured from p/p_0 = 0.995 to p/p_0 = 0.050. The Brunauer–Emmett–Teller surface areas (SA_{BET}) were calculated using the software BETSI (minimum number of points: 7; minimum R_2: 0.998; Rouquerol criteria 1–4: checked; small Error: 20).[12] The pore size distribution (PSD) were evaluated based on the adsorption isotherm, using data points down to 10^{-6} p/p_0, with a 2D non-local density functional theory (2D-NLDFT) analysis by the 2019 SAIEUS program from Micromeritics.

Solid-state ^{13}C cross-polarization magic angle spinning nuclear magnetic resonance (CP-MAS). The spectra were recorded using a Bruker AVANCE spectrometer 400 (101 MHz for ^{13}C), and a 4 mm rotor head under magic angle spinning conditions, with a rotation frequency of 10 kHz (4 mm rotor). The contact time was set to 1.0 ms, with 2700 scans (ns) and a recycle delay of 5 s. The experiments were performed at 298 K. The ^{13}C chemical shift values (δ) are expressed in parts per million in with respect to adamantane as a secondary standard (δ_C = 29.5 ppm).

Scanning electron microscopy (SEM). The micrographs were taken by using a benchtop microscope Phenon Pharos G2 from Thermo Scientific, using an acceleration voltage of 15 kV, and a secondary electron detector. Prior to

measurement, the COF materials were placed on carbon conductive tape and coated with a 20–30 nm gold layer. The micrographs show representative areas of the materials, and the morphology was consistent throughout all material.

Low-dose, High-Resolution Transmission Electron Microscopy (HR-TEM). The images were recorded with a low dose acquisition scheme (< 1 e $Å^{-2}$) by using SerialEM5 on JEM-2100 (JEOL GmbH, Eching, Germany) operated at 200 kV and equipped with a 4 k × 4 k CMOS digital camera (TVIPS TemCam-F416) at 90 K. The COF materials were sonicated in ethanol for 10 min at 298 K, afterwards, an aliquot dispersion (approximately 4.0 µL) was applied to Lacey carbon-coated copper TEM grids (200 mesh, Science Services), and allowed to air-dry. The TEM grids were loaded into a cryogenic transfer holder (Gatan 914, Gatan, Munich, Germany), followed to cool down at 77 K with liquid nitrogen. HR-TEM images were acquired at a magnification of 100,000× or 500,000× corresponding to a pixel size of 0.1198 nm or 0.0229 nm at the specimen level.

Atomic force microscopy (AFM). Film characterizations were performed using an AFM Multimode 8 (Bruker Corporation), software Nanoscope 9.4 with SCANASYST-AIR-HR probes: f_0 = 130 kHz; k = 0.4 N/m; T = 0.5 µm. Measurements were carried out by using ScanAsyst mode (Bruker Corporation). AFM images were flattened by using a second order plane correction and the height was measured by using the tool for cross section in the open-source software Gwyddion.[13] Optical microscopy images were taken on an Olympus LEXT OLS4100 laser scanning microscope. The open-source software Fiji was used for the generation of the figures.[14]

Thermogravimetric analysis (TGA). The measurements were carried out using a PerkinElmer Thermogravimetric Analyzer Pyris 1 TGA, from a temperature range of 303–1073 K. Initially, holding the temperature at 303 K for 10 min, and increasing the temperature at 60 K min^{-1}, and finally holding a constant temperature at 1073 K for 10 min.

Supercritical point drying ($scCO_2$ drying). The experiments were performed using a Samdri-PVT-3B equipment. The COF materials were placed into commercial teabags (the tea bags were bought from English Tea Store, ETS Drawstring Tea Filters) and stored in methanol for 24 h, without letting it get dried, the staple-sealed teabags were paced into the dryer chamber, and the chamber was full-filled with absolute ethanol. Then, the closed drying chamber was filled with liquid carbon dioxide

(CO_2, 99.999% purity) from Praxair, the absolute ethanol was completely flushed with liquid CO_2. After all ethanol was removed, the system was closed, and the pressure was allowed to rise to 58.6–62.0 bar, by increasing the temperature of the system to 304 K. The chamber was held above the critical point of CO_2 (T_c = 304 K, p_c = 73.8 bar). The pressure was constantly held above the critical pressure 73.8–103.4 bar by venting the system. After 10 min, the system was carefully opened, and the pressure was released slowly. The dried tea bags were stored under an argon atmosphere.

Fluorescence spectroscopy. The photoluminescence experiments were performed using an Edinburgh Instruments FLS 980 spectrometer using a Xe lamp at 298 K, and the emission was recorded from (400–800) nm, with an excitation bandwidth of 1 nm.

Steady-state absorption. A Shimadzu UV-3600 Plus spectrophotometer was used to collect the steady-state absorbance spectra of samples, which uses a photomultiplier tube. The final data shown is corrected for by measuring the same cuvette with the solvent only.

Steady-state and time-resolved photoluminescence (PL) spectra were recorded by a gated intensified CCD camera (Andor Star DH740 CCI-010) connected to a grating spectrometer (Andor SR303i). The pulsed output from a mode-locked Ti:sapphire optical amplifier (Spectra-Physics Solstice, 1.55 eV photon energy, 80 fs pulse width, 1 kHz repetition rate) was used to produce 400 nm excitation via second harmonic generation in a β-barium borate crystal. The iCCD gate (width 2 ns) was electronically stepped in 2 ns increments, relative to the pump pulse, to enable ns-temporal resolution of the PL decay. Faster (~100 ps resolved) kinetics were recorded using time–correlated single-photon counting (TCSPC), employing a Picoquant system at 405 nm excitation. The instrument response function (IRF) was determined using a solution of silica (glass) colloidal spheres to detect the laser scatter under otherwise identical excitation and detection conditions. This IRF was then used for the fitting of the fast time traces through convolution of the IRF with the fitting function to accurately describe the measured data using the model described below.

Steady-state PL spectra of polymer and monomer solutions in a 1 cm path length cuvette were measured using an Edinburgh Instruments FLS1000 spectrometer, using a Xe lamp at 298 K as excitation source (λ_{ex}= 300 nm, 320 nm for polymer, monomer

solutions respectively), in a 90° geometry between excitation and emission collection. The photoluminescence of the sample was collected by a photomultiplier tube (PMT - 900). Lifetimes of the polymer and monomer solutions in a 1 cm path length cuvette were collected by time-correlated single photon counting (TCSPC) at their respective peak emission wavelengths with the Edinburgh Instruments FLS1000 spectrometer, using an EPL-375 pulsed laser diode as excitation source (Fluence: 0.18 uJ/cm^2 (Power = 0.150 mW, rep rate = 50 MHz, spot size = ~ 3.25 mm diameter, excitation wavelength = 375 nm. Time-resolved PL measurements on the COF films were also performed by time-correlated single photon counting (TCSPC), with fluence: ~ 1.2 uJ/cm^2 (Power = 0.160 mW, rep rate = 50kHz, spot size= ~ 0.85 mm diameter). The 343 nm excitation source were generated from a 1030 nm seed laser (PHAROS, Light Conversion, Yb:KGW lasing medium, 400 µJ pulse energy, 150 fs duration, 50 kHz repetition rate) using a harmonic generation unit equipped with beta-barium borate and lithium triborate nonlinear crystals (HIRO, Light Conversion). Upon photoexcitation, fluorescence from the sample was collected in transmission mode (180°) and focused onto a photomultiplier tube (Becker & Hickl HPM-100-07) positioned behind the exit slit of a monochromator, where photon counting occurred using the single photon counting module provided by Light Conversion.

Steady-state CPL spectra of polymer films and monomer solutions (1 mm path length cuvette) were measured using the setup described by *Kitzmann et al.*[35] capable of differentiating linear polarization from intrinsic circularly polarized luminescence. The most important measurement parameters and instruments are summarized here: The sample was measured at 298 K in a 90° geometry, with a Thorlabs SOLIS LED as excitation source (365 nm, CW, unpolarized, Power = 3W upon spot with radius ~ 1.1mm: 80 W/cm^2). The emission was directed through a photo-elastic modulator (PEM, Hinds Instruments PEM200 II/FS42, f = 42 kHz), an ultra-broadband wire-grid polarizer (Thorlabs WP25M-UB, 250–4000 nm, extinction ratios: >1% for 300–4000 nm, >0.1% for 600–4000 nm, >0.01% for 2250–4000 nm) for thin samples and a Glan-Thompson polarizing prism. The emission was then directed onto the slit of a monochromator (Princeton Instruments, Acton SP-2155), and was collected by a photomultiplier tube (PMT, Hamamatsu H10723-20), which is operated at 5 V via a power supply (Agilent E2520A).

Photoluminescence quantum efficiency (PLQE) data was collected using the method described by *de Mello et al*.[15] Briefly, samples were positioned in an integrating sphere and excited at 400 nm, while the PL was collected with an Andor Shamrock spectrometer and Andor iDus CCD array. A corrected value is then determined by collecting the light from the sphere without a sample, without hitting the sample and with hitting the sample, respectively. Stated values were determined on triplicate samples which were each measured thrice, hence reporting the average of nine measurements for each composition. Transient absorption (TA) spectroscopy TA is a form of pump-probe spectroscopy which measures the spectrally resolved variation in absorption by a sample under photoexcitation by a pump source. By varying the pump-probe time delay, the carrier recombination kinetics of the sample can be investigated. The third harmonic of a pulsed Nd:YVO4 laser (Picolo-AOT MoPa) was used as the pump beam (~1 ns pulse width, 500 Hz repetition rate, 355 nm) for the ns regime measurements. The probe spectrum was generated using a white light quasi-continuum generated through pumping a CaF2 window with the 800 nm fundamental of a Ti:Sapphire amplifier (Spectra-Physics Solstice). A delay generator was used to electronically vary the pump-probe delay. For the short time fs-regime, the pump beam was the second harmonic (400 nm) generated by the 800 nm fundamental passing through a β-barium borate crystal. The transmitted probe and reference pulses were recorded with an NMOS linear image sensor (Hamamatsu S8381-1024Q) and processed by a customized PCI interface from Entwicklungsbüro Stresing.

The PLQE of the polymer solutions was measured using the Edinburgh Instruments FLS1000 spectrometer, additionally equipped with an integrating sphere, using a Xe lamp at 298 K as excitation source (λ_{ex}= 300 nm) and collected by a photomultiplier tube (PMT-900), analogous to the steady-state photoluminescence measurements detailed above. Both the sample and the blank solvent were measured twice to better estimate the statistical variance and measurement uncertainty. The measurement range was expanded compared to the conventional photoluminescence measurements to capture the emission (scatter) of sample and blank at the excitation wavelength.

S2. Synthetic Procedures

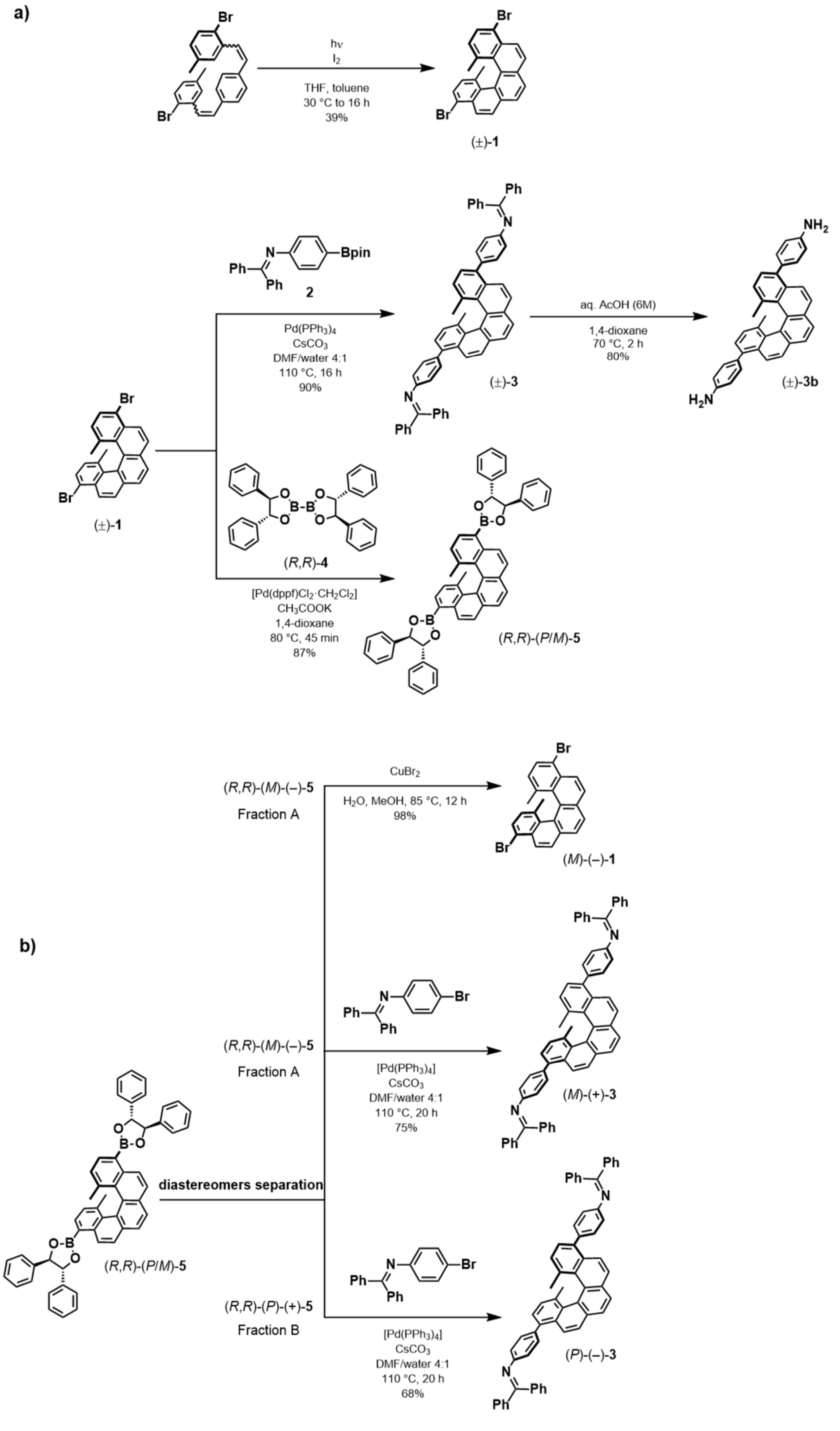

Scheme S1. General synthetic route for racemic and enantiopure building blocks for the synthesis of [5]HeliCOFs; (a) synthesis of racemic building blocks; (b) synthesis of enantiopure building blocks.

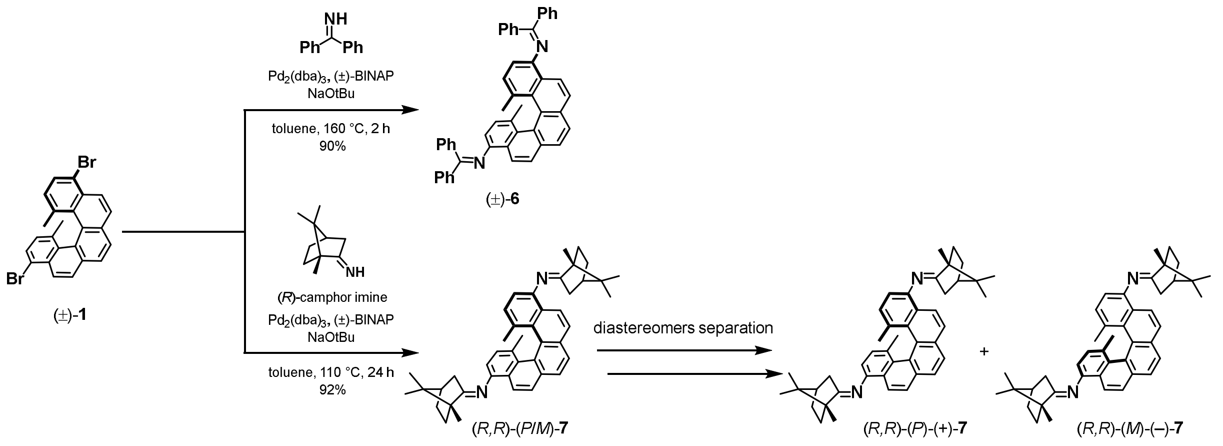

Scheme S2. General synthetic routes for racemic and enantiopure building blocks for the synthesis of [5]Heli-TFP POPs; (top) synthesis of racemic building block; (bottom) synthesis and separation of diastereomers.

(±)-7,14-Dibromo-10,11-dimethyldibenzo[*c*,*g*]phenanthrene (±)-1[1]

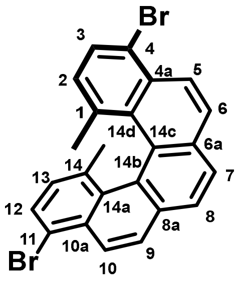

Based on a published procedure,[1] (±)-**1** (810 mg, 4.44 mmol, 39%, Lit.: 73%[1]) was synthesized from 1,4-bis(2-bromo-5-methylstyryl)benzene[1] (2.08 g, 4.44 mmol, racemic mixture ca. 15:9:1 (*cis*,*cis*)/(*cis*,*trans*)/(*trans*,*trans*)). The spectral data are in line with the literature.[1]

***R**$_f$* = 0.57 (UV, SiO_2, cyclohexane); **^{1}H NMR** (500 MHz, $CDCl_3$, 25 °C) δ = 8.40 (d, *J* = 8.7 Hz, 2H, H–C(5,10)), 8.08 (s, 2H, H–C(7,8)), 8.00 (d, *J* = 8.7 Hz, 2H, H–C(6,9)), 7.78 (d, *J* = 7.8 Hz, 2H, H–C(3,12)), 6.95 (d, *J* = 7.8 Hz, 2H, H–C(2,13)), 0.89 ppm (s, 6H, H_3C–C(1,14)); **^{13}C NMR** (126 MHz, $CDCl_3$, 25 °C): δ = 134.7 (C(1,14)), 133.9 (C(14a,14d)), 131.5 (C(6a,8a)), 131.4 (C(4a,10a)), 130.2 (C(3,12)), 129.3 (C(2,13)), 127.1 (C(6,9)), 127.0 (C(7,8)), 126.6 (C(5.10)), 126.2 (C(14b,14c)), 119.9 (C(4,11)), 22.4 ppm (CH_3); **HR-ESI(+)-MS**: *m*/*z* (%) = found: 463.37 (26, $[M + H]^+$ calc. for $C_{24}H_{16}{}^{79}Br_2{}^+$, 463.25); found: 465.46 (50, $[M + H]^+$, calc. for $C_{24}H_{16}{}^{79}Br^{81}Br^+$, 465.25), found: 467.38 (24, $[M + H]^+$, calc. for $C_{24}H_{16}{}^{81}Br_2{}^+$, 467.25).

N-(4-(4,4,5,5-Tetramethyl-1,3,2-dioxaborolan-2-yl)phenyl)-1,1-diphenylmethanimine (2)

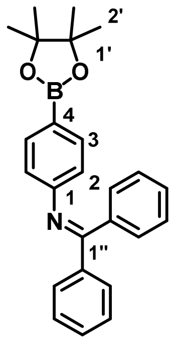

A solution of 4-bromo-*N*-(diphenylmethylidene)aniline[2] (1.002 g, 2.8 mmol, 1.0 equiv.) in 1,4-dioxane (28 mL) was treated with bis(pinacolato)diboron (830.9 mg, 3.207 mol, 1.1 equiv.), and potassium acetate (876.0 mg, 8.5 mmol, 3 equiv.). The reaction mixture was purged with Argon for 30 min at 25 °C. Pd(dppf)Cl_2 (105 mg, 141 µmol, 0.05 equiv.) was added and heated for 6 h at 85 °C. The resulting mixture was allowed to cool down to 25 °C, the solvent was removed in vacuo, and the crude product was purified via MPLC (cyclohexane/EtOAc (+0.5% Et_3N) 100:0→98:2) to obtain **2** (713 mg, 66%) as a yellow solid.

***R*f** = 0.41 (UV; SiO_2, cyclohexane/EtOAc 98:2 (+0.5% Et_3N)); **^{1}H NMR** (500 MHz, CD_2Cl_2, 25 °C): δ = 7.74 (d, *J* = 7.9 Hz, 2H, H–benzophenone), 7.54 (d, *J* = 7.3 Hz, 2H, H–C(3)), 7.49 (t, *J* = 7.4 Hz, 1H, H–C(H–benzophenone)), 7.42 (t, *J* = 7.6 Hz, 2H, H–benzophenone), 7.33–7.23 (m, 3H, H–benzophenone), 7.16–7.10 (m, 2H, H–benzophenone), 6.70 (d, *J* = 7.7 Hz, 2H, H–C(2)), 1.30 ppm (s, 12H, H_3C–C(2’)), rotamers at the NMR time scale cause a more complex spectrum due to the rotation of the benzophenone imine residue at the C–N bond; **^{13}C NMR** (126 MHz, CD_2Cl_2): δ = 168.5 (C(1’’)), 154.8(C(1)), 139.9 (C–benzophenone), 136.7 (C–benzophenone), 135.6 (C(2)), 131.4 (C–benzophenone), 129.8 (C–benzophenone), 129.8 (C–benzophenone), 129.1 (C–benzophenone), 128.7 (C–benzophenone), 128.4 (C–benzophenone), 120.4 (C(3)), 84.1 (C(1’)), 25.2 ppm (C(2’)). Note: one carbon corresponding at C(4) is hidden by the noise; **MS:** ESI(+)-MS: *m*/*z*: 384.31 (calc. for $C_{25}H_{27}BNO_2^+$ [*M* + H]$^+$: 384.21).

(±)-*N,N'*-((10,11-Dimethyldibenzo[*c,g*]phenanthrene-7,14-diyl)bis(4,1-phenylene))bis(1,1-diphenylmethanimine) (±)-3

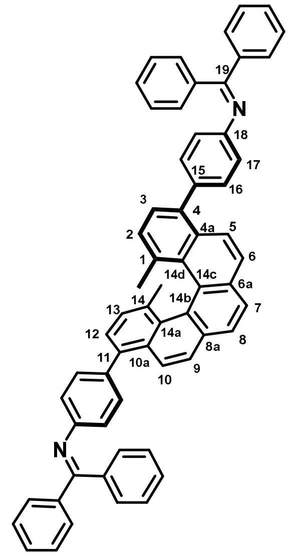

A suspension of [5]helicene (±)-**1**[1] (180 mg, 0.38 mmol, 1.0 equiv.) and boronic ester **2** (343 mg, 0.80 mmol, 2.1 equiv.) in DMF (31 mL) was treated with a solution of caesium carbonate (0.15 M, 7.68 mL, 1.15 mmol, 3.0 equiv.) at 25 °C. The pale-yellow suspension was degassed using a stream of argon for 20 min. $Pd(PPh_3)_4$ (46 mg, 38.4 μmol, 0.09 equiv.) was added to the reaction mixture, obtaining an intense yellow suspension. Under intense stirring, the reaction mixture was immersed in a preheated oil bath at 110 °C for 16 h. The dark-brown suspension was allowed to cool to 25 °C, the mixture was filtered through a pad of silica, and washed with CH_2Cl_2/Et_3N 99:1. The organic solvents were evaporated (for removing the DMF residual, high vacuum (10^{-2} mbar) was applied). The remaining pale-yellow solid was purified by flash-column chromatography (SiO_2, EtOAc/cyclohexane + 0.5% Et_3N, 100:0→95:5) to provide the bis-benzophenone imine (±)-**3** (282 mg, 90%) as a crystalline yellow solid.

***R*$_f$** = 0.45 (UV; SiO_2, EtOAc/cyclohexane 95:5 [+0.5% Et_3N]); **^{1}H NMR** (500 MHz, CD_2Cl_2, 25 °C, assignments based on ^{1}H,^{1}H-COSY, ^{1}H,^{13}C-HSQC and ^{1}H,^{13}C-HMBC NMR spectra): δ = 8.02 (s, 2H, H–C(7,8)), 7.88 (d, *J* = 8.7 Hz, 2H, H–C(5,10)), 7.83–7.77 (m, 6H, H–C(6,9), H–C(benzophenone imine)), 7.56–7.40 (m, 8H, H–C(2,13), H–C(benzophenone imine)), 7.40–7.31 (m, 10H, H–C(17), H–C(benzophenone imine)), 7.29–7.20 (m, 4H, H–C(benzophenone imine)), 7.08 (dd, *J* = 7.4, 0.9 Hz, 2H, H–C(C3,12)), 6.91–6.84 (m, 4H, H–C(16)), 0.95 ppm (s, 6H, H3C–C(1,14)), rotamers at the NMR time scale cause a more complex spectrum due to the rotation of the benzophenone imine residue at the C–N bond; **^{13}C NMR** (126 MHz, $CDCl_3$, assignments based on ^{1}H,^{1}H-COSY, ^{1}H,^{13}C-HSQC and ^{1}H, ^{13}C-HMBC NMR spectra): δ= 168.7 (C(19)), 151.1 (C(18)), 140.0 (C(14b,14c) 137.7 (C(4,11)), 136.8 (C(4a,10a), 136.3 (C(15)), 134.5 (C(1,14)), 133.0 (C(14a,14d)), 131.5 (C(17)), 131.4 (C(6a,8a)), 131.2 (C(benzophenone imine)), 131.2 (C(benzophenone imine)), 130.0

(C(benzophenone imine)), 129.7 (C(benzophenone imine)), 129.1 (C(benzophenone imine)), 128.6 (C(benzophenone imine)), 128.5 (C(3,12)), 128.4(C(benzophenone imine)), 127.1 (C(benzophenone imine)), 126.9 (C(2,13)), 126.4 (C(7,8)), 125.9 (C(5,10)), 125.6 (C(6,9)), 121.2 (C(16)), 22.4 ppm (CH_3); **HR-ESI-MS**: *m*/*z* (%): 817.3593 (100, calc. for $C_{62}H_{45}N_2^+$ [*M* + H]$^+$: 817.3577).

(±)-4,4'-(10,11-Dimethyldibenzo[*c*,*g*]phenanthrene-7,14-diyl)dianiline (±)-3b

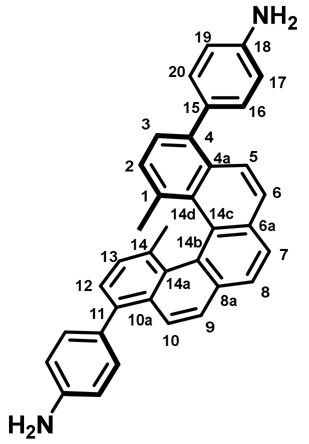

A solution of (±)-**3** (15 mg, 0.03 mmol, 1.0 equiv.) in 1,4-dioxane (2 mL) was treated with aqueous acetic acid (6 M, 31 µL, 0.30 mmol, 10.0 equiv.) at 25 °C. Under intense stirring, the reaction mixture was immersed in a preheated oil bath at 70 °C for 2 h. The solution was cooled to 25 °C and diluted with CH_2Cl_2 (10 mL), the organic phase was washed with sat. $NaHCO_3$ (2×5 mL), water (2×5 mL), and brine (2×5 mL). The organic phase was dried over Mg_2SO_4, filtered, and the solvent was removed under reduced pressure to afford a yellow solid. Crystallization from CH_2Cl_2/toluene 10:1 (80 mL, 25 °C) by slow evaporation over 48 h afforded (±)**-3b** (7 mg, 80%) as yellow crystals. Unambiguous structural proof was obtained by a single-crystal X-ray structure (Figure S125).

^{1}H NMR (500 MHz, CD_2Cl_2, 25 °C, assignments based on ^{1}H,^{1}H-COSY, ^{1}H,^{13}C-HSQC and ^{1}H,^{13}C-HMBC NMR spectra): δ = 8.07–8.01 (m, 4H, H–C(7,8), H–C(6,9)), 7.89 (d, *J* = 8.7 Hz, 2H, H–C(5,10)), 7.45 (d, *J* = 7.3 Hz, 2H, H–C(2,13)), 7.41–7.36 (m, 4H, H–C(C17)), 7.10 (dd, *J* = 7.4, 0.9 Hz, 2H, H–C(C3,12)), 6.89–6.83 (m, 4H, H–C(C16)), 3.89 (br. s, 4H, H–N), 0.99 ppm (s, 6H, H_3C–C(1,14)); **^{13}C NMR** (126 MHz, $CDCl_3$, assignments based on ^{1}H,^{1}H-COSY, ^{1}H,^{13}C-HSQC and ^{1}H,^{13}C-HMBC NMR spectra): δ = 146.5 (C(18)), 138.1 (C(4)), 134.1, 133.1, 132.0 (C(19), 131.4 (17), 128.5 (C(3,12)), 127.0 (C(2,13)), 126.9, 126.3 (C7,C8), 126.2 (C(6,9)) 125.4 (C(5,10)), 115.1 (C(16,20)), 22.4 (CH_3); **HR-ESI-MS**: *m*/*z* (%) calc. for $C_{62}H_{45}N_2^+$ [*M* + H]$^+$: 489.2331; found: 489.2205.

(*R*,*R*)-(*P*/*M*)-2,2'-(10,11-Dimethyldibenzo[*c,g*]phenanthrene-7,14-diyl)bis(4,5-diphenyl-1,3,2-dioxaboralane) (*R*,*R*)-(*P*/*M*)-5 and pure diastereomers (*R*,*R*)-(*P*)-(+)-5 and (*R*,*R*)-(*M*)-(–)-5

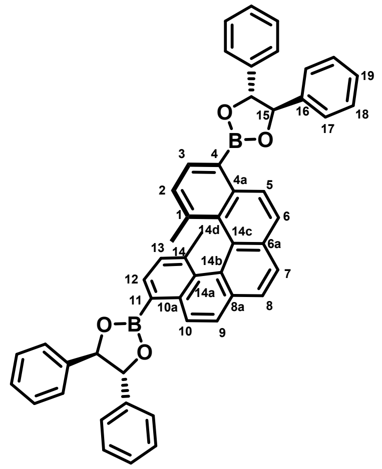

An oven dried pressure tube was charged with (±)-**1**[1] (320 mg, 0.69 mmol, 1 equiv.), (*R*,*R*)-(+)-**4** (923 mg, 2.07 mmol, 3 equiv.),[16] and potassium acetate (406 mg, 4.14 mmol, 6 equiv.). Dry 1,4-dioxane (35 mL) was added and the mixture was degassed using a stream of argon for 20 min. [Pd(dppf)Cl_2·CH_2Cl_2] (56 mg, 0.07 mmol, 0.1 equiv.) was added to the dispersion and heated to 80 °C for 45 min. The mixture was concentrated under reduced pressure and purified using column chromatography (SiO_2-enriched with boric acid,[17] cyclohexane/acetone 8:2, ran at fast operating speed to prevent excessive decomposition). Drying under vacuum afforded (*R*,*R*)-(*P*/*M*)-**5** (153.3 mg, 0.168 mmol, 87%) as a pale-yellow solid. Preparative HPLC using a Regis Whelk-O1 (*S*,*S*) solid phase and *n*-hexane/*i*-PrOH as a mobile phase (see Section S16) gave diastereomerically pure (*R*,*R*)-(*M*)-(–)-**5** (retention time t_R = 32.0 min, fraction A) and (*R*,*R*)-(*P*)-(+)-**5** (t_R = 36.5 min, fraction B) as pale-yellow solids.

(*R*,*R*)-(*P*/*M*)-**5** (diastereomer mixture):

***R*$_f$** = 0.7 (UV; SiO_2, cyclohexane/acetone 8:2); **^{1}H NMR** (500 MHz, $CDCl_3$, 25 °C, assignments based on ^{1}H,^{1}H-COSY, ^{1}H,^{13}C-HSQC, and ^{1}H,^{13}C-HMBC NMR spectra): δ = 9.04 (dd, *J* = 8.7, 3.7 Hz, 2H, H–C(5,10)), 8.31 (dd, *J* = 7.1, 3.9 Hz, 2H, H–C(3,13)), 7.96 (dd, *J* = 8.8, 3.0 Hz, 2H, H–C(6,9)), 7.52–7.40 (m, 20H, H–C(16–19)), 7.18 (d, *J* = 7.2 Hz, 2H, H–C(2,13)), 5.51 (s, 4H, H–C(15)), 1.03 and 1.02 ppm (s, *J* = 3.6 Hz, 6H, H_3C–C(1,14) of each diastereomer); **^{13}C NMR** (126 MHz, $CDCl_3$, 25 °C, assignments based on ^{1}H,^{1}H-COSY, ^{1}H, ^{13}C-HSQC, and ^{1}H,^{13}C-HMBC NMR spectra): δ = 140.6 (C(16–19)), 139.5 (C(14a,14d)), 137.6 (C(14b,14c)), 136.2 (C(4a,10a)), 136.0 (C(3,13)), 132.4 (C(1,14)), 130.9 (C(6a,8a)), 129.9 (C(16–19)), 128.6 (C(16–19)), 128.0 (C(2,13)), 127.6 (C(5,10)), 126.6 (C(6,9)), 126.3 (C(7,8)), 126.2 (C(16–19)), 87.1 (C(15)), 23.1 ppm (CH_3), signal for C(4,11), bound to the boron was hidden by the noise due to quadrupolar coupling; **HR-MALDI-MS:** *m*/*z* (%): 749.3133 (50, [*M* + H]$^+$ calc. for

$C_{52}H_{40}{}^{10}BBO_4{}^+$, 749.3144), 750.3090 (100, [*M*]$^+$ calc. for $C_{52}H_{40}B_2O_4{}^+$, 750.3107), 751.3146 (75, [*M* + H]$^+$ calc. for $C_{52}H_{41}B_2O_4{}^+$, 751.3185).

(*R*,*R*)-(*P*/*M*)-**5**:

α_D^{21} = –(208±1)° (*c* 1.014, CH_2Cl_2).

(*R*,*R*)-(*P*)-(+)-**5**:

α_D^{21} = +(17±2)° (*c* 0.171, CH_2Cl_2).

(*R*,*R*)-(*M*)-(–)-**5**:

α_D^{21} = –(501±10)° (*c* 0.100, CH_2Cl_2).

((*P*)-(–)-*N,N'*-((10,11-Dimethyldibenzo[*c,g*]phenanthrene-7,14-diyl)bis(4,1-phenylene))bis(1,1-diphenylmethanimine) (*P*)-(–)-3

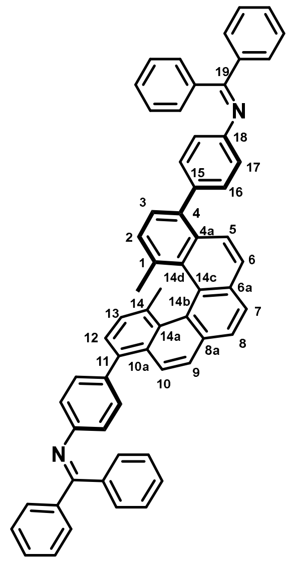

A suspension of (*R*,*R*)-(*P*)-(+)-**5** (51.5 mg, 0.07 mmol, 1.0 equiv. fraction B) and **6**[2] (79 mg, 0.24 mmol, 3.0 equiv.) in DMF (6.2 mL) was treated with aqueous caesium carbonate (0.25 M, 1.6 mL, 0.39 mmol, 5.0 equiv.) at 25 °C. The pale-yellow suspension was degassed using a stream of argon for 20 min. $Pd(PPh_3)_4$ (9 mg, 7.8 µmol, 0.1 equiv.) was added to the reaction mixture, obtaining an intense yellow suspension. Under intense stirring, the reaction mixture was immersed in a preheated oil bath at 110 °C for 20 hours. The dark-brown suspension was allowed to cool to 25 °C, the mixture was filtered through silica, and washed with CH_2Cl_2/Et_3N 99:1. The organic solvents were evaporated (up to 10^{-2} mbar) and the pale yellow solid was purified by flash-column chromatography (SiO_2, cyclohexane/EtOAc [+0.5% Et_3N] 100:0→95:5) to provide (*P*)-(–)-**3** (43 mg, 68%) as a crystalline yellow solid.

***R*$_f$** = 0.45 (UV, SiO_2, cyclohexane/EtOAc 95:5 [+0.5% Et_3N]); **^{1}H NMR** (500 MHz, CD_2Cl_2, 25 °C, assignments based on ^{1}H,^{1}H-COSY, ^{1}H,^{13}C-HSQC, and ^{1}H,^{13}C-HMBC NMR spectra): δ = 8.04 (s, 2H, H–C(7,8)), 7.89 (d, *J* = 8.7 Hz, 2H, H–C(5,10)), 7.84–7.79 (m, 6H, H–C(6,9), H–C(benzophenone imine)), 7.55–7.50 (m, 2H, H–C(benzophenone imine)), 7.48–7.42 (m, 6H, H–C(2,13), H–C(benzophenone imine)), 7.41–7.37 (m, 10H, H–C(C17), H–C(benzophenone imine)), 7.30–7.23 (m, 4H, H–C(benzophenone imine)), 7.09 (dd, *J* = 7.4, 0.9 Hz, 2H, H–C(C3,12)), 6.92–6.86 (m, 4H, H–C(C16)), 0.95 ppm (s, 6H, H3C–C(1,14)), rotamers at the NMR time scale cause a more complex spectrum due to the rotation of the benzophenone imine residue at the C–N bond. **HR-ESI-MS**: *m*/*z* (%): 817.3553 (100, calc. for $C_{62}H_{45}N_2^+$ [*M* + H]$^+$: 817.3577); α_D^{19} = –(250±3)° (*c* 0.071, CH_2Cl_2).

(*M*)-(+)-*N*,*N'*-((10,11-Dimethyldibenzo[c,g]phenanthrene-7,14-diyl)bis(4,1-phenylene))bis(1,1-diphenylmethanimine) (*M*)-(+)-3

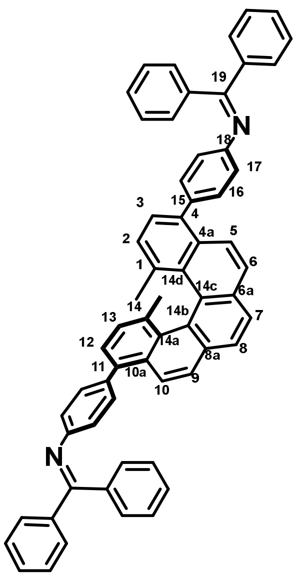

A suspension of (*R*,*R*)-(*M*)-(–)-**5** (60.1 mg, 0.08 mmol, fraction A, 1.0 equiv.) and **6** (69 mg, 0.20 mmol, 3.0 equiv.) in DMF (4.0 mL) was treated with aqueous caesium carbonate (0.25 M, 1.4 mL, 0.34 mmol, 5.0 equiv.) at 25 °C. The pale-yellow suspension was degassed using a stream of argon for 20 min. $Pd(PPh_3)_4$ (8 mg, 6.8 µmol, 0.1 equiv.) was added to the reaction mixture, obtaining an intense yellow suspension. Under intense stirring, the reaction mixture was immersed in a preheated oil bath at 110 °C for 20 hours. The dark-brown suspension was allowed to cool to 25 °C, the mixture was filtered through silica, and washed with CH_2Cl_2/Et_3N 99:1. The organic solvents were evaporated (up to 10^{-2} mbar) and the pale yellow solid was purified by flash-column chromatography (SiO_2, cyclohexane/EtOAc [+0.5% Et_3N] 100:0→95:5) to provide (*M*)-(+)-**3** (42 mg, 75%) as a crystalline yellow solid.

***R*f** = 0.45 (UV, SiO_2, cyclohexane/EtOAc 95:5 [+0.5% Et_3N]); **^{1}H NMR** (500 MHz, CD_2Cl_2, 25 °C) (assignments based on ^{1}H,^{1}H-COSY, ^{1}H,^{13}C-HSQC and ^{1}H,^{13}C-HMBC NMR spectra) : δ = 8.03 (s, 2H, H–C(7,8)), 7.89 (d, *J* = 8.7 Hz, 2H, H–C(5,10)), 7.84–7.80 (m, 6H, H–C(6,9), H–C(H–benzophenone imine)), 7.55– 7.50 (m, 2H, H–C(H–benzophenone imine)), 7.48 – 7.41 (m, 6H, H–C(2,13), H–C(H–benzophenone imine)), 7.41–7.36 (m, 10H, H–C(C17)), H–C(H–benzophenone imine)), 7.29 – 7.24 (m, 4H, H–C(H–benzophenone imine)), 7.09 (dd, *J* = 7.4, 0.9 Hz, 2H, H–C(C3,12)), 6.92 – 6.87 (m, 4H, H–C(C16)), 0.96 (s, 6H, H3C–C(1,14)); rotamers at the NMR time scale cause a more complex spectrum due to the rotation of the benzophenone imine residue at the C–N bond; **HR-ESI-MS**: *m*/*z* (%): 817.3540 (100, calc. for $C_{62}H_{45}N_2^+$ [*M* + H]$^+$: 817.3577); α_D^{19} = +(256±2)° (*c* 0.148, CH_2Cl_2). Unambiguous structural proof was obtained by a single-crystal X-ray structure (Figure S126).

(*M*)-(–)-7,14-dibromo-10,11-dimethyldibenzo[*c*,*g*]phenanthrene (*M*)-(–)-1[1]

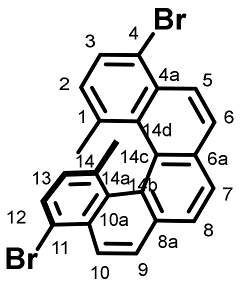

A solution of the isolated fraction A of the chiral HPLC-separated (*R*,*R*)-(*M*)-(–)-**5** (8 mg, 10.6 μmol, 1.0 equiv.) in methanol (2.7 mL) was intensely stirred at 85 °C for 10 min, resulting in a clear solution, which was then cooled to 25 °C. This solution was treated with $CuBr_2$ (24 mg, 0.11 mmol, 10 equiv.), and water (450 μL) was added. Next, the reaction mixture was intensely stirred at 85 °C for 12 h.[18] The resulting blue-green suspension was cooled to 25 °C and diluted with CH_2Cl_2 (2 mL). The organic phase was washed with water (2×5 mL) and brine (2 mL). The organic phase was dried over anhydrous Mg_2SO_4, filtered, and the solvents were removed under reduced pressure to afford a yellow solid. The remaining pale-yellow solid was purified via column chromatography (SiO_2, EtOAc/cyclohexane, 100:0→98:2), affording (*M*)-(–)-**3** (4.8 mg, 10.0 μmol, 98%) as a crystalline yellow solid. Unambiguous structural proof of the absolute configuration of (*M*)-(–)-**3** was confirmed by a single-crystal X-ray analysis. Crystals were grown from a saturated solution in $CHCl_3$ and vapor diffusion of MeCN at 10 °C for72 h (Figure S78). The following ^{1}H NMR spectral data are in line with the literature of the racemic (±)-**1**.[1]

^{1}H NMR (500 MHz, $CDCl_3$, 25 °C) δ = 8.40 (d, *J* = 8.8 Hz, 2H, H–C(5,10)), 8.08 (s, 2H, H–C(7,8)), 8.00 (d, *J* = 8.8 Hz, 2H, H–C(6,9)), 7.78 (d, *J* = 7.8 Hz, 2H, H–C(3,12)), 6.95 (d, *J* = 7.8 Hz, 2H, H–C(2,13)), 0.89 ppm (s, 6H, H_3C–C(1,14)). Due to the low amount of the sample

^{13}C NMR spectrum was not possible to be recorded with an adequate signal-to-noise ratio. Unambiguous structural prove is provided by single-crystal X-ray diffraction (Figure S127).

***N,N'*-(10,11-Dimethyldibenzo[c,g]phenanthrene-7,14-diyl)bis(1,1-diphenylmethanimine) ((±)-6)**

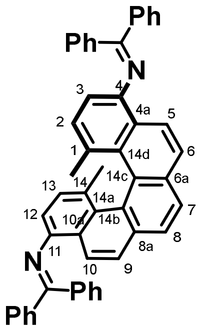

In a pressure tube, *rac*-BINAP (43.9 mg, 0.69 mmol) and $Pd_2(dba)_3$ (17.7 mg, 0.23 mmol) were suspended in toluene (5 mL). The mixture was degassed under a stream of argon for 20 min and heated at 120 °C for 45 min. After cooling to 25 °C and under a flow of argon, (±)-1[1] (107 mg, 0.23 mmol), benzophenone imine (119 mg, 1.00 mmol), NaOtBu (69 mg, 0.69 mmol), and toluene (12 mL) were added to the reaction mixture. The mixture was subsequently heated at 160 °C for 1 h. The mixture was cooled at 25 °C and MeOH (1 mL) was added. The resulting mixture was filtered through a silica plug and rinsed with CH_2Cl_2/Et_3N 99:1. The filtrate was concentrated under reduced pressure, purified by flash-column chromatography (SiO_2, cyclohexane/EtOAc 9:1 (with 1% Et_3N to prevent degradation), and dried under vacuum, affording (±)-**6** (130 mg, 0.23 mmol, 85%) as a pale-yellow solid. Unambiguous structural proof and absolute configuration is provided by single-crystal X-ray diffraction of the enantiopure sample (Figure S128).

***R*f** = 0.57 (UV, SiO_2, cyclohexane/EtOAc 95:5 [+0.5% Et_3N]); **m.p.** 260–263 °C; **^{1}H NMR** (500 MHz, CD_2Cl_2, 25 °C): δ = 8.24 (d, *J* = 8.6 Hz, 2H, H–C(5,10)), 8.02 (s, 2H, H–C(7,8)), 7.95–7.92 (m, 4H, $H_{benzophenone}$), 7.90 (d, *J* = 8.6 Hz, 2H, H–C(6,9)), 7.58–7.53 (m, 2H, $H_{benzophenone}$), 7.53–7.47 (m, 4H, $H_{benzophenone}$), 7.29–7.19 (m, 10H, $H_{benzophenone}$), 6.80–6.74 (m, 2H, H–C(3,12)), 6.53 (d, *J* = 7.7 Hz, 2H, H–C(2,13)), 0.70 ppm (s, 6H, 19); **^{13}C NMR** (126 MHz, CD_2Cl_2, 25 °C): δ = 168.2 (C=N), 146.3 ($C_{benzophenone}$), 139.6 ($C_{benzophenone}$), 137.0 (C(1,14)), 132.9 (C(14a,14d)), 131.8 (C(6a,8a)), 131.2 ($C_{benzophenone}$), 130.5 ($C_{benzophenone}$), 129.8 ($C_{benzophenone}$), 129.3 ($C_{benzophenone}$), 128.9 (C(3,12)), 128.8 (C(2,13)), 128.7($C_{benzophenone}$), 128.34 (($C_{benzophenone}$), 126.7, 126.51 (C(7,8)), 125.6 ((C(6,9)), 123.5 (C(5.10)), 115.9 (C(2,13)), 22.4 ppm (CH_3); **HR-ESI-MS**: *m*/*z*: 665.2962 ([*M* + H]$^+$, calc. for $C_{50}H_{37}N_2^+$: 665.2952).

(1*R*,1'*R*,2*E*,2'*E*,4*R*,4'*R*)-*N*,*N*'-(10,11-Dimethyldibenzo[c,g]phenanthrene-7,14-diyl)bis(1,7,7-trimethylbicyclo[2.2.1]heptan-2-imine) ((*R*,*R*)-(*P*/*M*)-7)) and pure diastereomers (*R*,*R*)-(*P*)-(+)-7 and (*R*,*R*)-(*M*)-(–)-7)

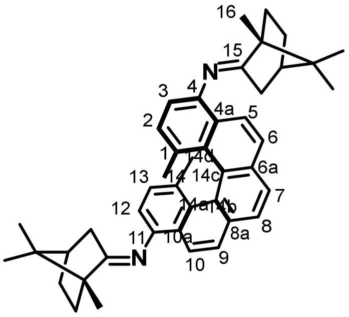

A pressure tube was charged with *rac*-BINAP (79 mg, 0.12 mmol), $Pd_2(dba)_3$ (39 mg, 0.03 mmol), and toluene (5 mL). The mixture was degassed using a stream of argon for 20 min (the solution turned red), and it was heated for 45 min at 120 °C. The mixture was allowed to cool at 25 °C. Under a flow of argon, (±)-**1**[1] (190 mg, 0.40 mmol), (*R*)-camphor imine[19] (248 mg, 1.60 mmol), and NaOtBu (236 mg, 2.48 mmol) were added, and toluene (15 mL) was used to wash the walls. The reaction mixture was heated at 110 °C for 24 h, then it was cooled to 25 °C, and MeOH (2 mL) was added. The resulting mixture was filtered through a silica plug, and flushed with CH_2Cl_2/Et_3N 99:1. The filtrate was concentrated under reduced pressure and purified by flash-column chromatography (SiO_2, cyclohexane/EtOAc 98:2 (1% Et_3N) to prevent degradation). Drying under vacuum afforded (*R*,*R*)-(*P*/*M*)-**7** (223 mg, 0.40 mmol, 92%) as a crystalline-yellow solid.

Preparative HPLC using a Regis Whelk-O1 (*S*,*S*) solid phase and *n*-hexane/*i*-PrOH as a mobile phase (see Section S15) gave diastereomerically pure (*R*,*R*)-(*M*)-(–)-**7** (retention time *t*R = 18.0 min, fraction A) and (*R*,*R*)-(*P*)-(+)-**7** (*t*R = 20.5 min, fraction B) as pale-yellow solids.

(*R*,*R*)-(*P*/*M*)-**7** (diastereomer mixture):

***R*f** = 0.60 (UV, SiO_2, cyclohexane/EtOAc 95:5 [+0.5% Et_3N]); **m.p.** 240–245 °C; **^{1}H NMR** (500 MHz, CD_2Cl_2, 25 °C): δ = 8.01 (s, 2H, H–C(7,8)), 7.97 (dd, *J* = 8.6, 6.0 Hz, 2H, H–C(5,10)), 7.88–7.83 (m, 2H, H–C(6,9)), 7.02 (d, *J* = 7.6 Hz, 2H, H–C(2,13)), 6.80 (dd, *J* = 7.6, 2.7 Hz, 2H, H–C(3,12)), 2.29 (ddd, *J* = 17.7, 4.1, 2.2 Hz, 1H, camphor), 2.10 (ddd, *J* = 17.9, 4.0, 2.4 Hz, 1H, camphor), 2.00–1.89 (m, 6H, camphor), 1.84 (d, *J* = 17.7 Hz, 1H, camphor), 1.77–1.64 (m, 4H, camphor), 1.39–1.31 (m, 1H, camphor), 1.28 (d, *J* = 3.8 Hz, 6H, camphor), 1.05 (d, *J* = 2.5 Hz, 6H), 1.01 (s, 3H), 0.95 (s, 3H), 0.91 ppm (d, *J* = 6.3 Hz, 6H, camphor); **^{13}C NMR** (126 MHz, CD_2Cl_2, 25 °C): δ = 186.5 (d, *J* = 25.0 Hz, C(15)), 146.7 (d, *J* = 11.5 Hz, C(4,11)), 133.1 (d, *J* = 9.8 Hz), 131.7 (d, *J* = 3.5 Hz, (C(1,14)), 130.1 (d, *J* = 4.8 Hz), 129.2 (d, *J* = 3.1 Hz, C(3,12)), 126.8 (d, *J* = 6.2 Hz, (C(5,10)), 126.4 (C(8,7)), 125.2 (t, *J* = 4.1 Hz), 122.5 (d, *J*

= 7.3 Hz, C(6,9)), 115.2, 54.9, 47.8 (d, *J* = 11.4 Hz), 44.4, 36.8 (d, *J* = 12.5 Hz), 32.8 (d, *J* = 15.8 Hz), 27.8 (d, *J* = 9.9 Hz), 22.1 (d, *J* = 12.6 Hz, CH_3–C(1,14)), 20.0 (CH_3–camphor), 19.2 (CH_3–camphor), 11.6 ppm (d, *J* = 5.3 Hz, CH_3–camphor). The observed split of the signals in ^{13}C NMR spectrum is related to the diastereotopic carbons present in the mixture, since each diastereomer can have slightly different ^{13}C chemical shift for each carbon; **HR-ESI-MS**: *m*/*z*: 605.3900 (calc. for $C_{44}H_{49}N_2^+$: 605.3891). Unambiguous structural proof is provided by single-crystal X-ray diffraction of the enantiopure sample (Figure S129).

(*R*,*R*)-(*P*)-(+)-**7**:

α_D^{21} = +(286±1)° (*c* 0.95, CH_2Cl_2). Unambiguous structural proof and absolute configuration is provided by single-crystal X-ray diffraction of the enantiopure sample (Figure S130).

(*R*,*R*)-(*M*)-(–)-**7**:

α_D^{21} = –(203±1)° (*c* 0.96, CH_2Cl_2).

(1*R*,4*R*)-1,7,7-Trimethylbicyclo[2.2.1]heptan-2-imine)[19] ((*R*)-camphor imine)

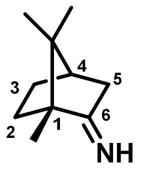

The procedure was adapted from the reported racemic approach.[19] An oven-dried pressure tube was charged with (*R*)-camphor (2.00 g, 12.3 mmol), Sc(OTF)$_3$ (653 mg, 1.3 mmol), hexamethyldisilazane (5.4 mL), and chlorobenzene (5.4 mL) to obtain a suspension. The mixture was heated at 90 °C for 24 h. After cooling at 25 °C, MeOH (2 mL) was added, and the mixture was stirred for 1 h. The mixture was filtered through a plug of silica, flushed with diethyl ether, and treated with HCl (10.7 mL of a 2 M solution in diethyl ether). The resulting suspension was stirred at 25 °C for 1 h. The mixture was concentrated under reduced pressure, and the colorless solid was rinsed with cold diethyl ether to afford (*R*)-camphor imine (1.5 g, 12.9 mmol, 77%, Lit: 68%)[19] as a colorless solid. The characterization of (*R*)-camphor imine is in line with the literature.[19]

B.p. 153–154 °C (10^{-2} mbar, sublimation); **^{1}H NMR** (500 MHz, $CDCl_3$, 25 °C): δ = 12.96 (br s, 1H), 3.09–2.95 (m, 1H), 2.69–2.54 (m, 1H), 2.17 (q, *J* = 3.0 Hz, 1H), 2.02–1.89 (m, 2H), 1.53–1.36 (m, 2H), 1.35 (s, 3H, CH_3), 1.00 (s, 3H, CH_3), 0.86 ppm (s, 3H, CH_3); **^{13}C NMR** (126 MHz, CD_2Cl_2, 25 °C): δ = 209.7 (C=N), 58.1, 50.2, 43.5, 38.8, 32.0, 26.2, 19.6, 18.6, 9.4 ppm; **HR-ESI-MS**: *m*/*z*: 152.1433 ([*M* + H]$^+$, calc. for $C_{10}H_{18}N^+$: 152.1434).

S3. Selected NMR spectra.

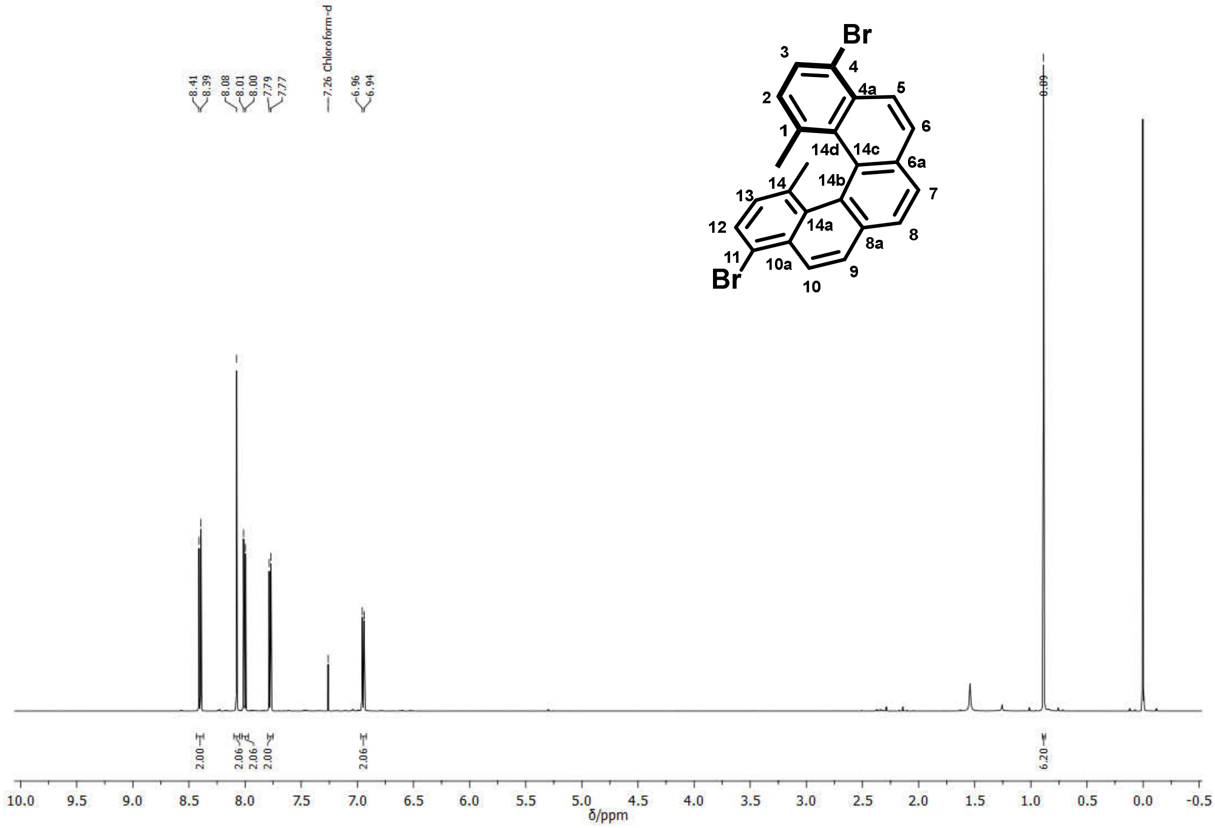

Figure S1. ^{1}H NMR (500 MHz, 298 K) of (±)-**1** in $CDCl_3$.

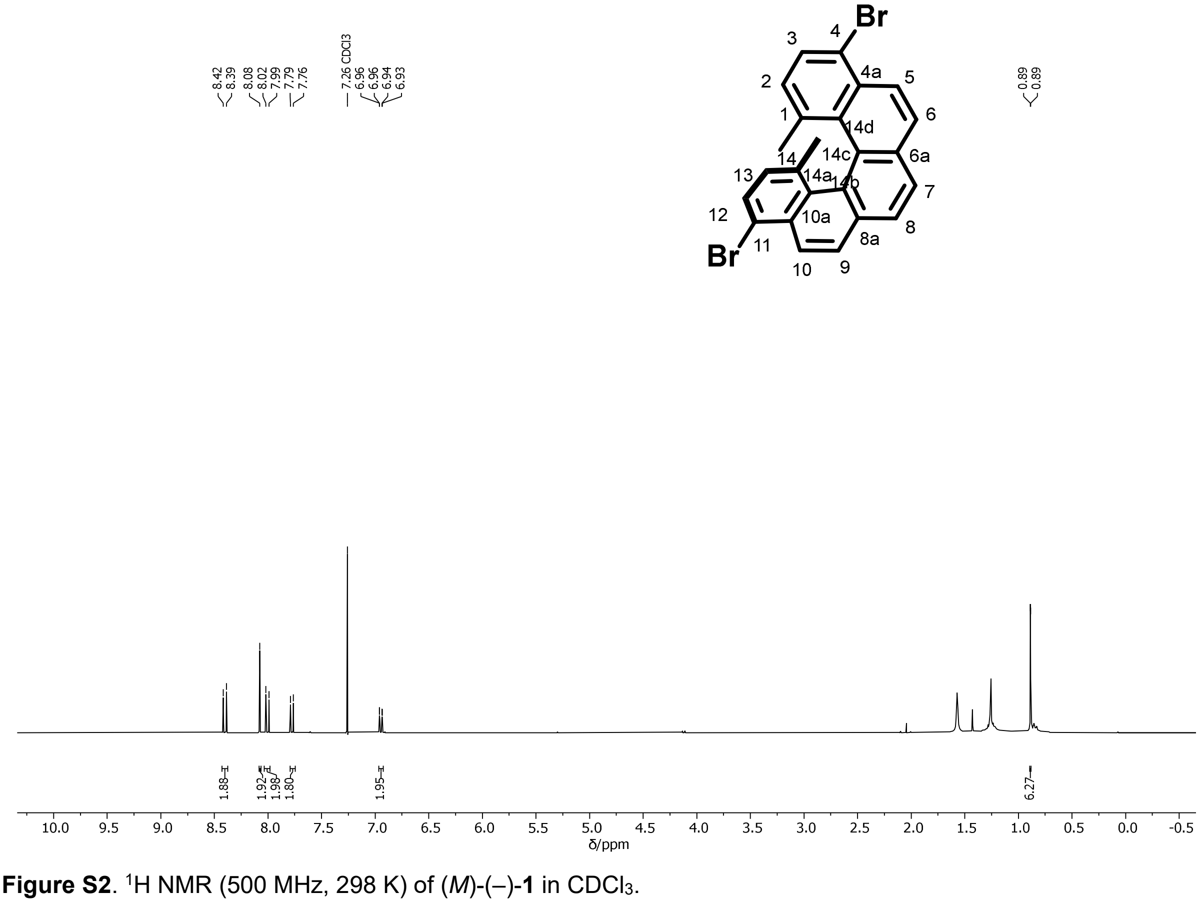

Figure S2. ^{1}H NMR (500 MHz, 298 K) of (*M*)-(–)-**1** in $CDCl_3$.

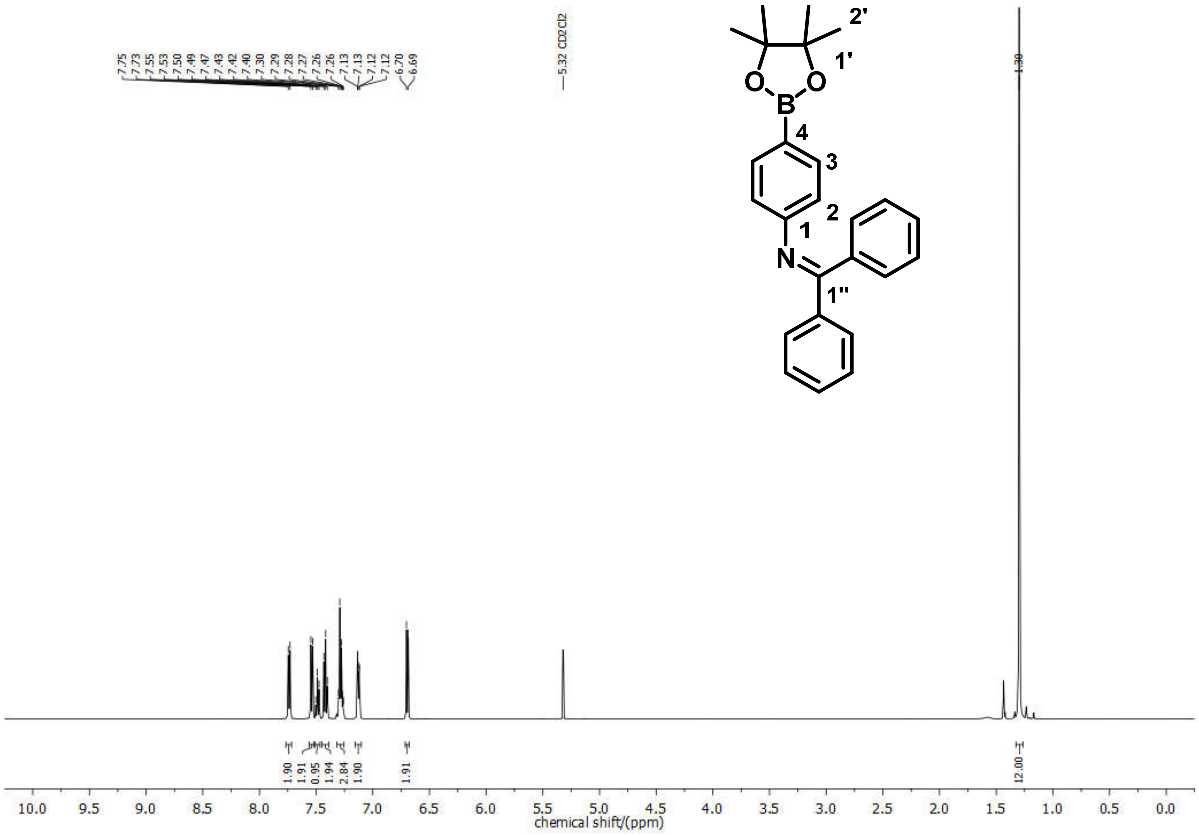

Figure S3. ^{1}H NMR (500 MHz, 298 K) spectrum of **2** in CD_2Cl_2.

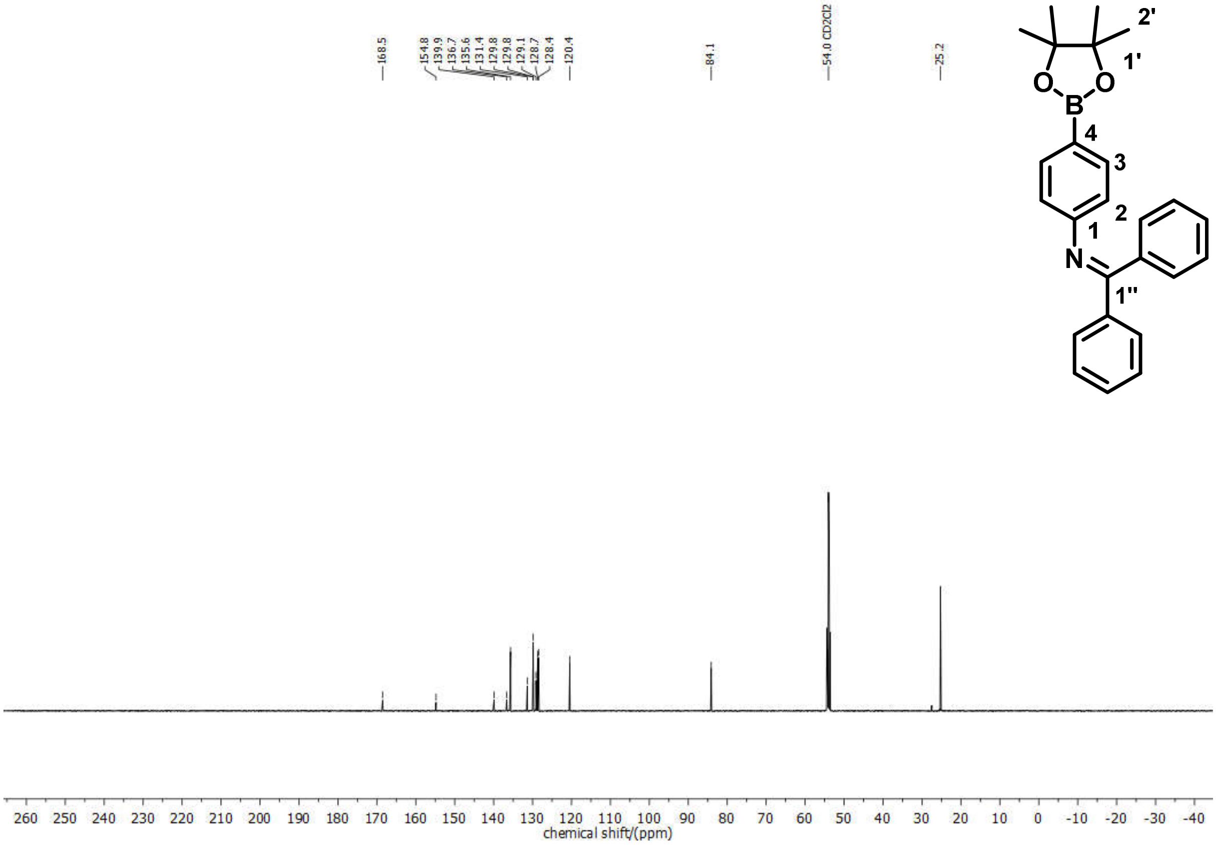

Figure S4. ^{13}C NMR (126 MHz, 298 K) spectrum of **2** in CD_2Cl_2.

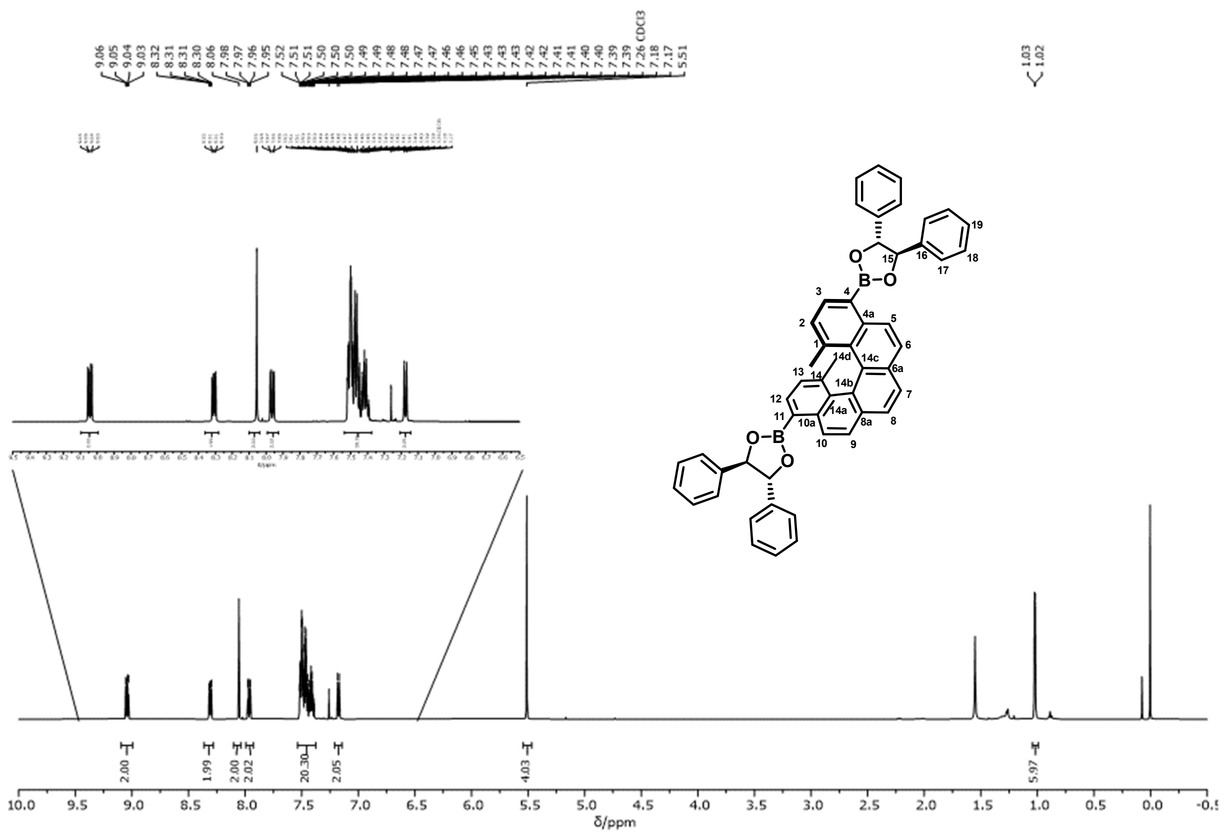

Figure S5. ^{1}H NMR (500 MHz, 298 K) of (*R*,*R*)-(*P*/*M*)-**5** in $CDCl_3$.

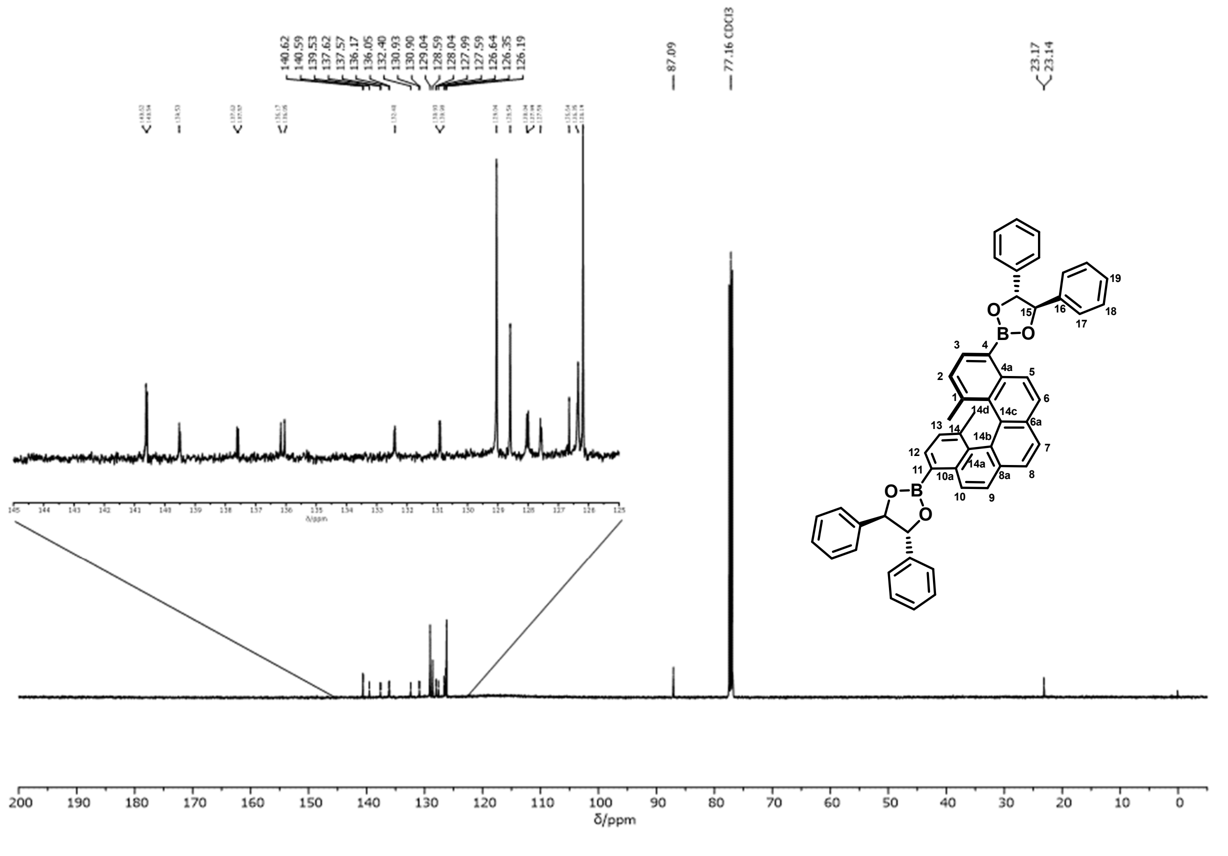

Figure S6. ^{13}C NMR (126 MHz, 298 K) of (*R*,*R*)-(*P*/*M*)-**5** in $CDCl_3$.

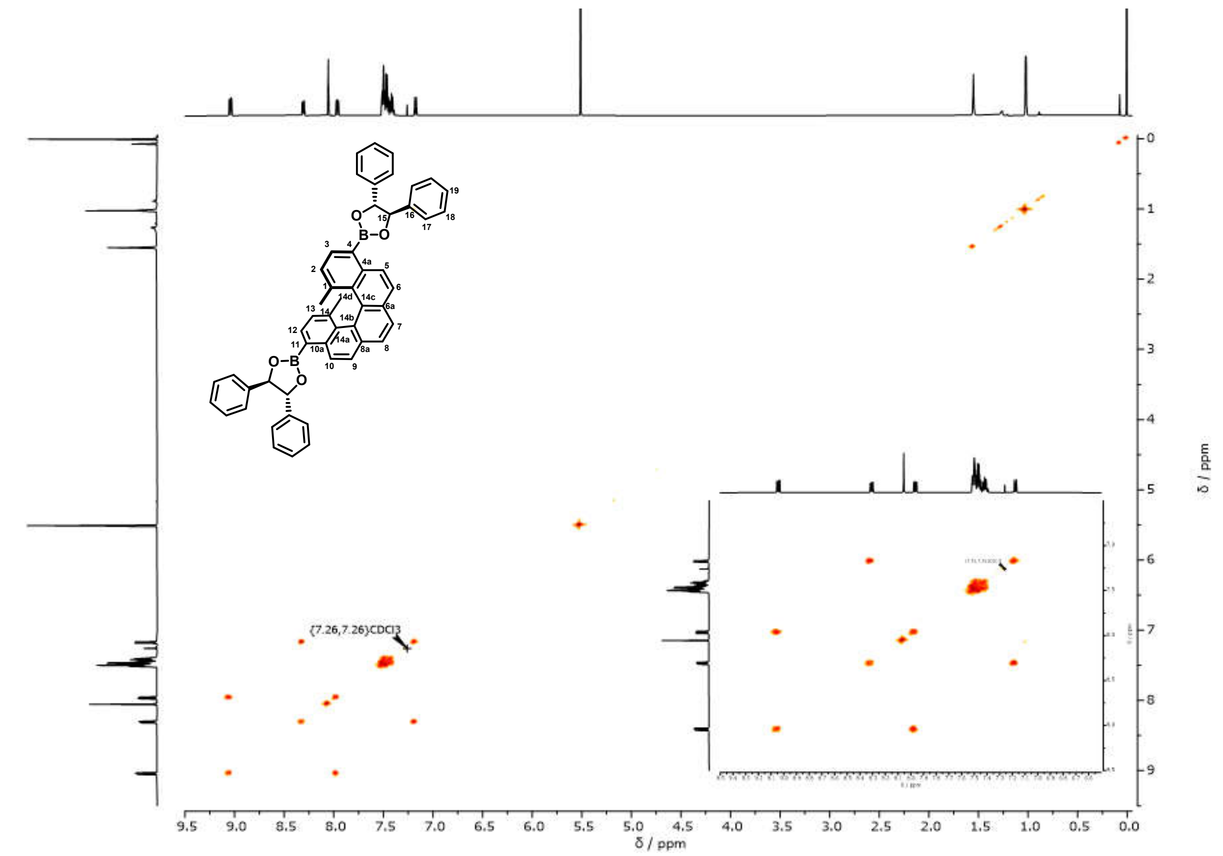

Figure S7. $^{1}H,^{1}H$-COSY NMR (500 MHz, 298 K) of (*R*,*R*)-(*P*/*M*)-**5** in $CDCl_3$.

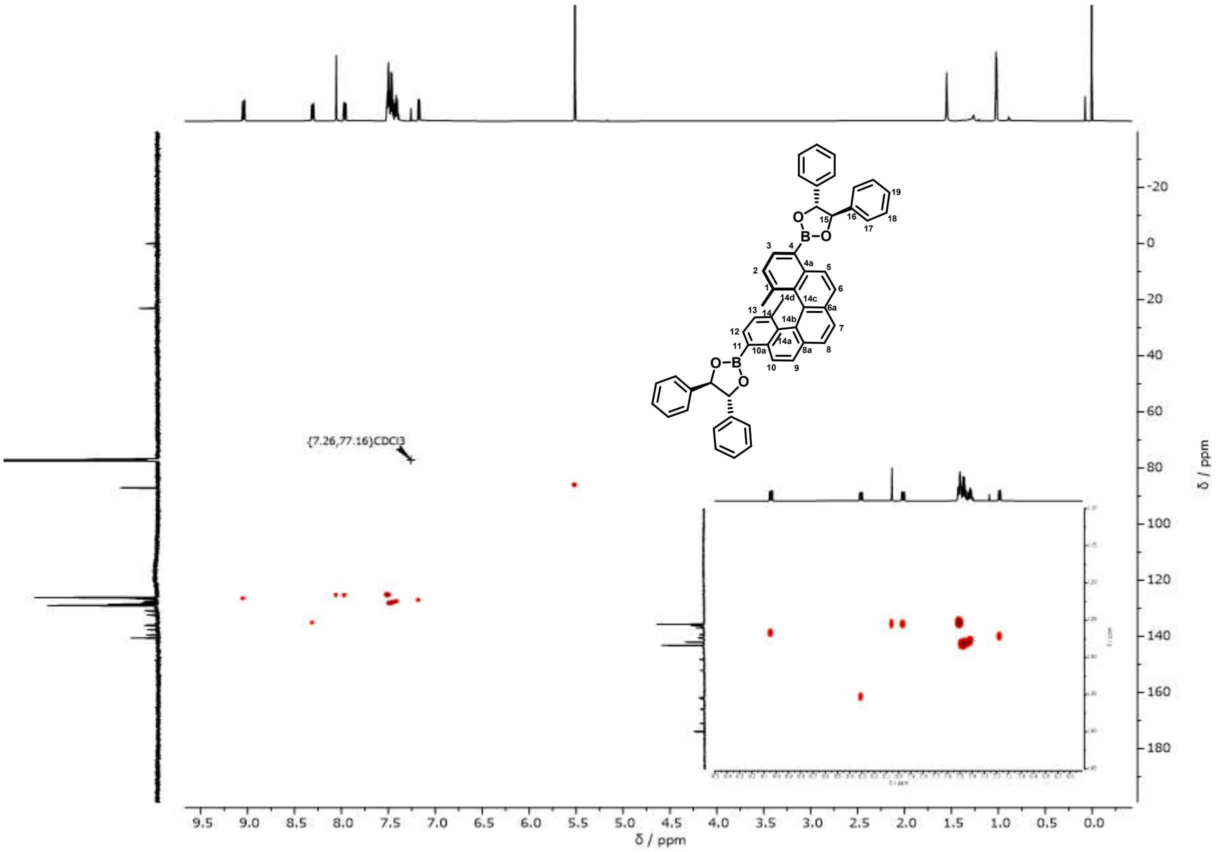

Figure S8. $^{1}H,^{13}C$-HSQC NMR (500, 126 MHz, 298 K) of (*R*,*R*)-(*P*/*M*)-**5** in $CDCl_3$.

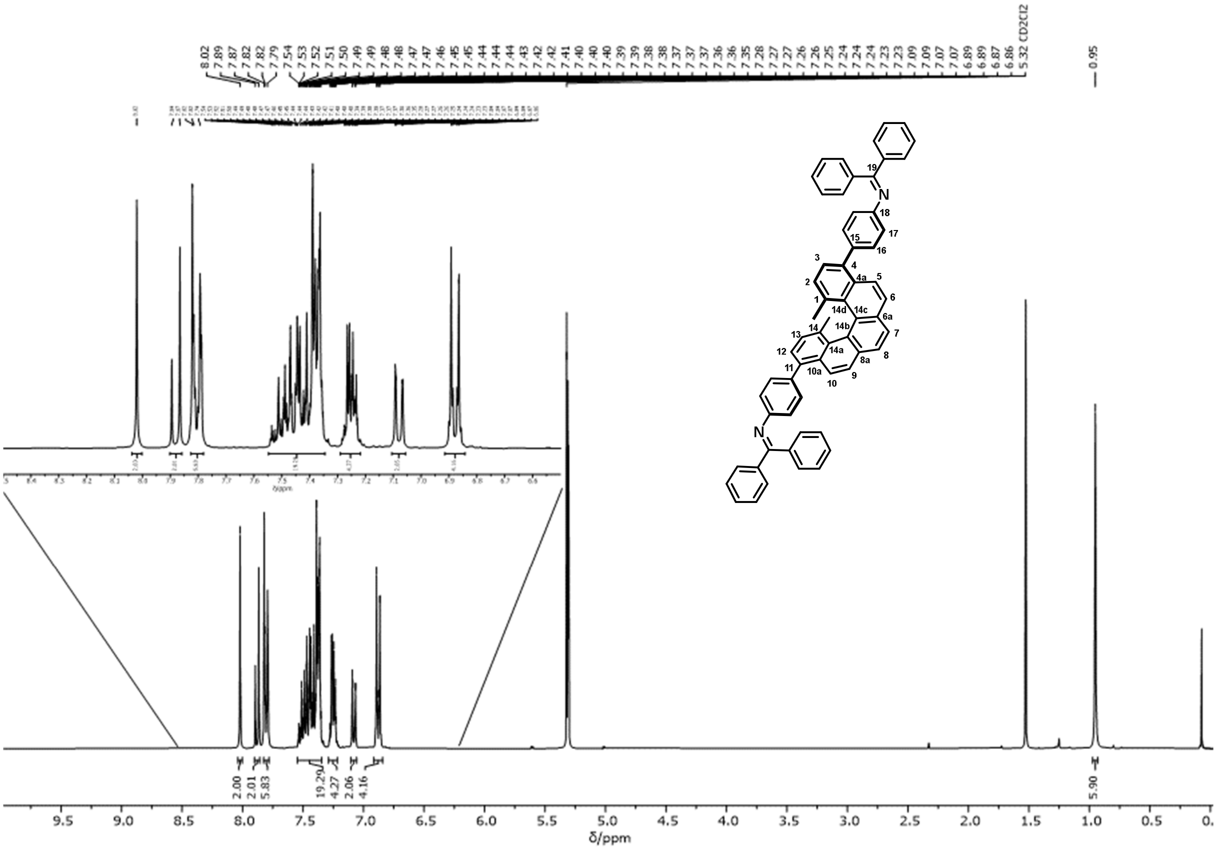

Figure S9. ^{1}H NMR (500 MHz, 298 K) spectrum of (±)-**3** in CD_2Cl_2.

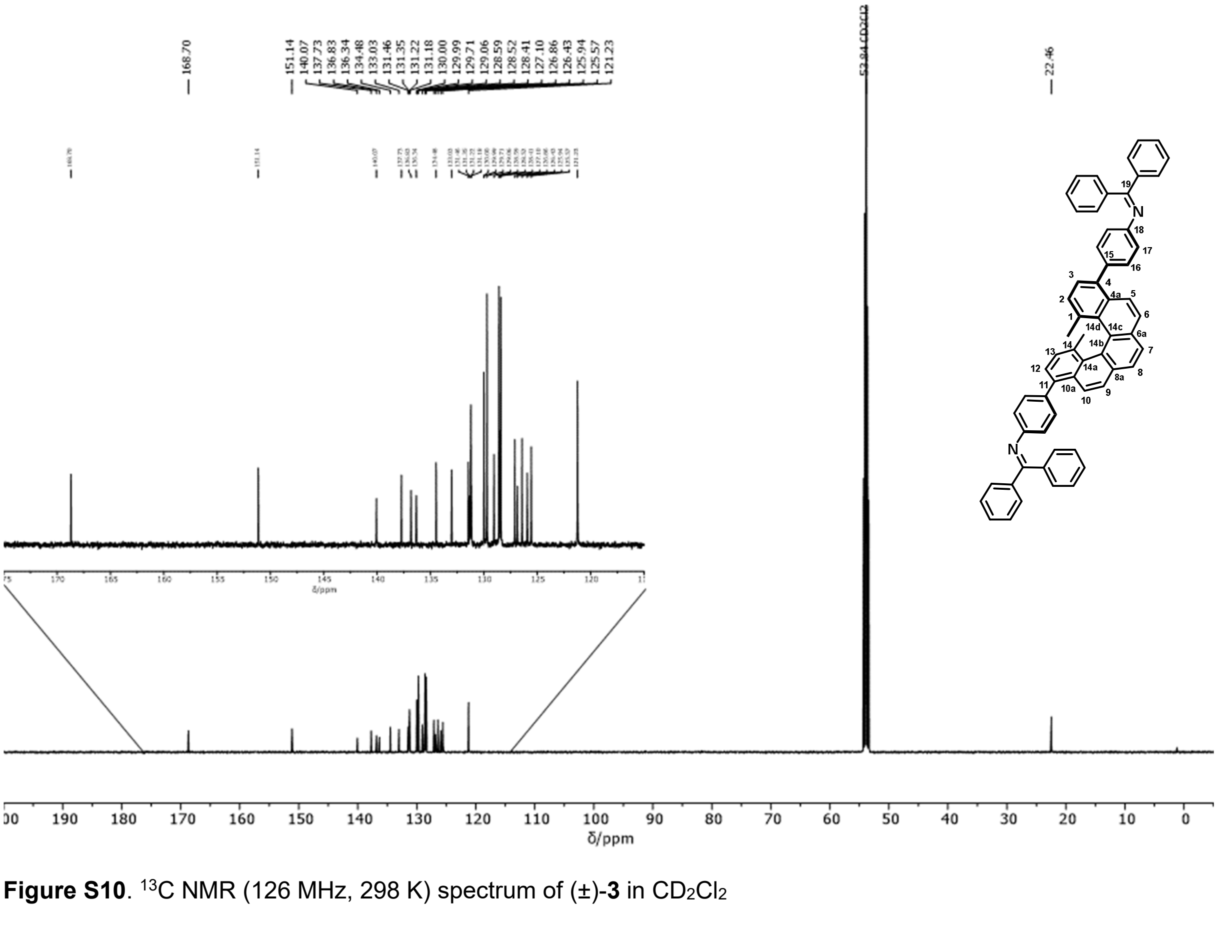

Figure S10. ^{13}C NMR (126 MHz, 298 K) spectrum of (±)-**3** in CD_2Cl_2

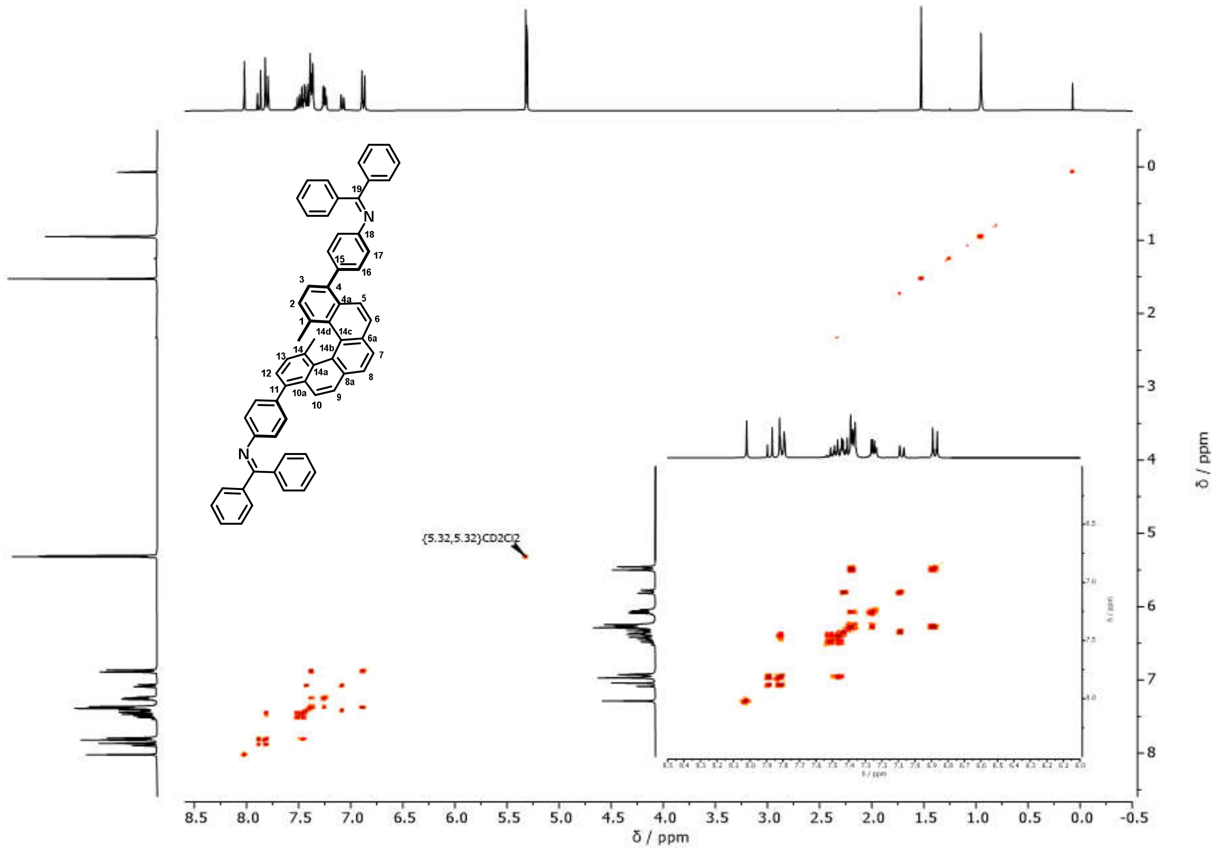

Figure S11. $^{1}H,^{1}H$-COSY NMR (500 MHz, 298 K) spectrum of (±)-**3** in CD_2Cl_2.

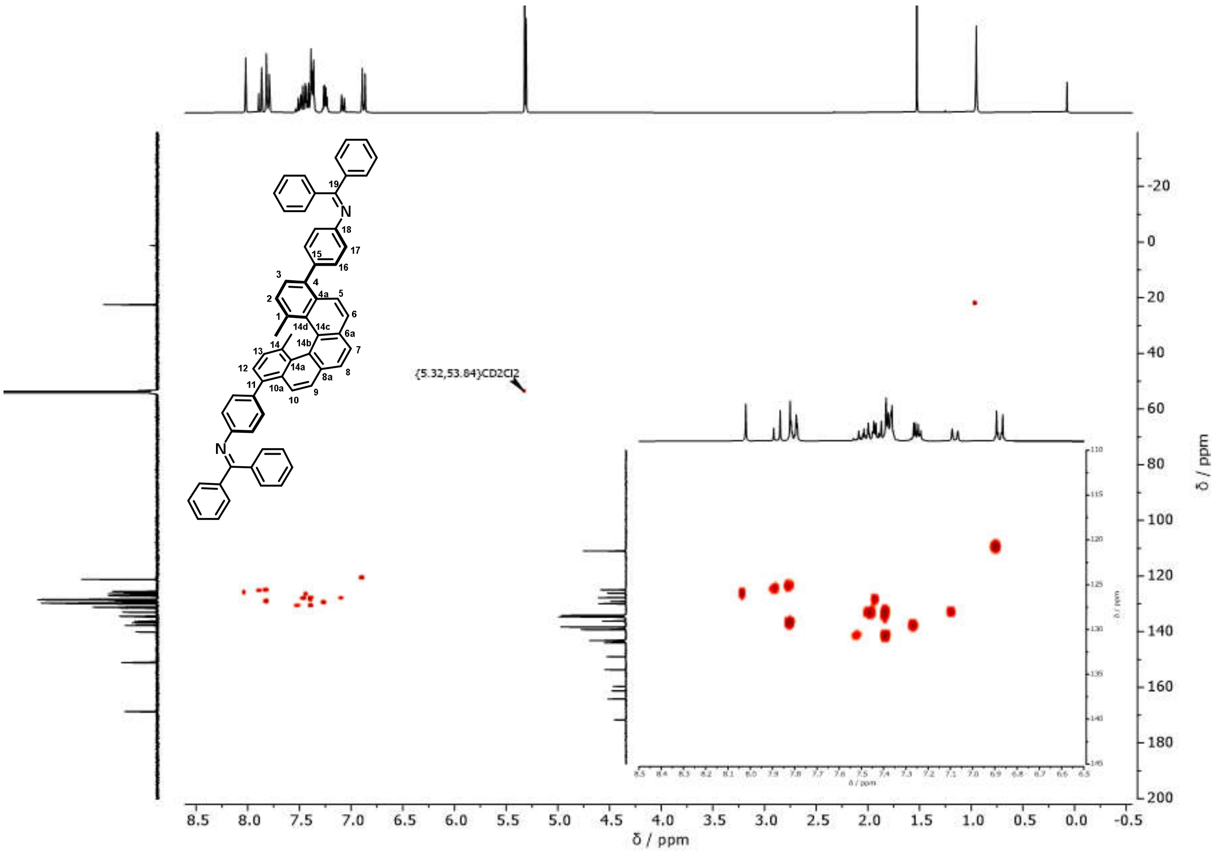

Figure S12. $^{1}H,^{13}C$-HSQC NMR (500, 126 MHz, 298 K) spectrum of (±)-**3** in CD_2Cl_2.

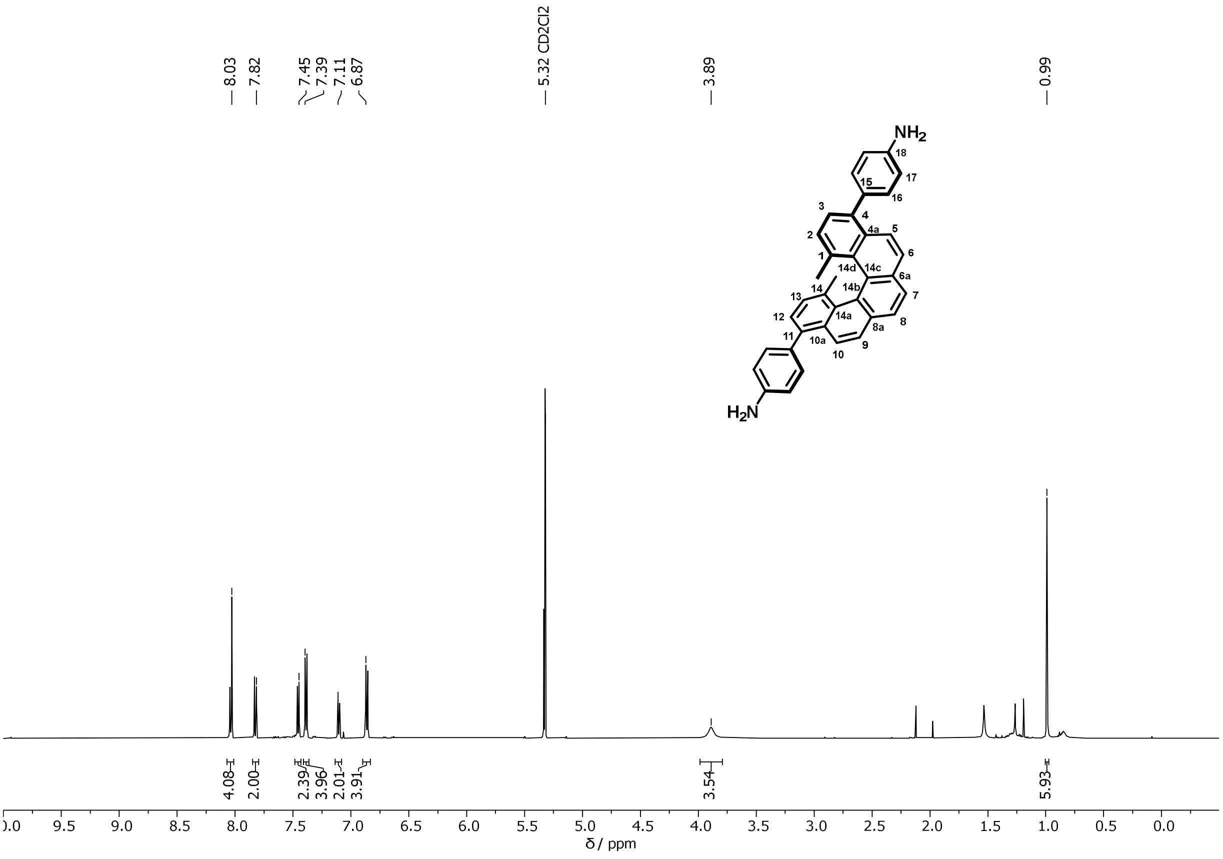

Figure S13. ^{1}H NMR (500 MHz, 298 K) spectrum of (±)-**3b** in CD_2Cl_2.

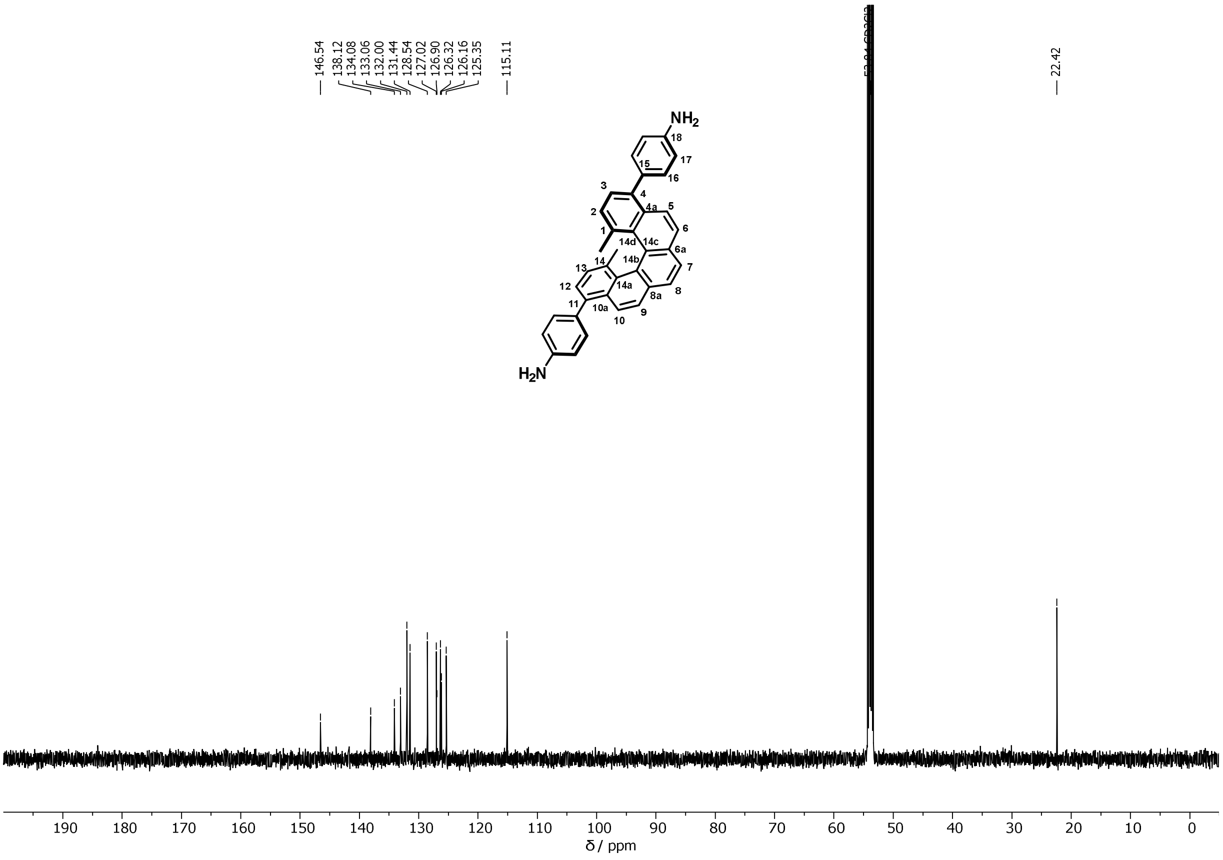

Figure S14. ^{13}C NMR (126 MHz, 298 K) spectrum of (±)-**3b** in CD_2Cl_2.

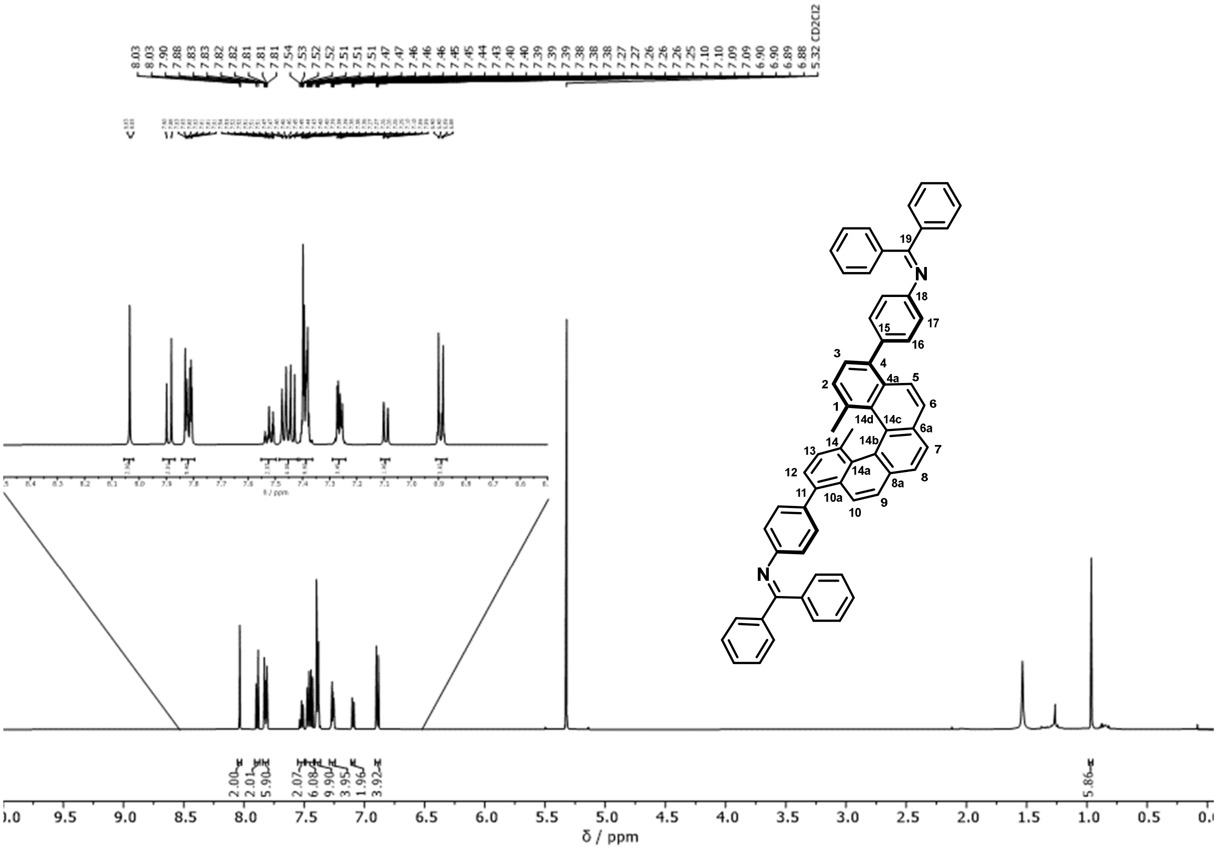

Figure S15. ^{1}H NMR (500 MHz, 298 K) spectrum of (*P*)-(–)-**3** in CD_2Cl_2.

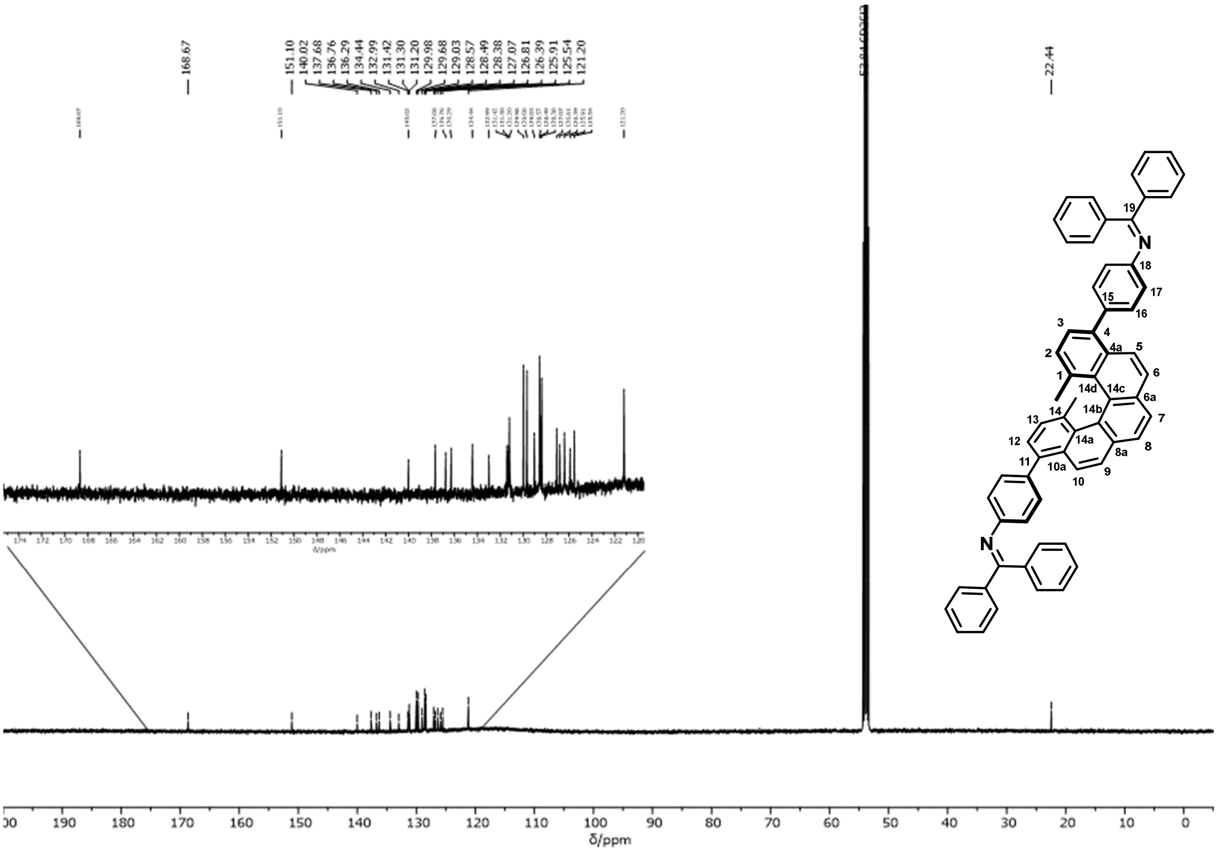

Figure S16. ^{13}C NMR (126 MHz, 298 K) spectrum of (*P*)-(–)-**3** in CD_2Cl_2.

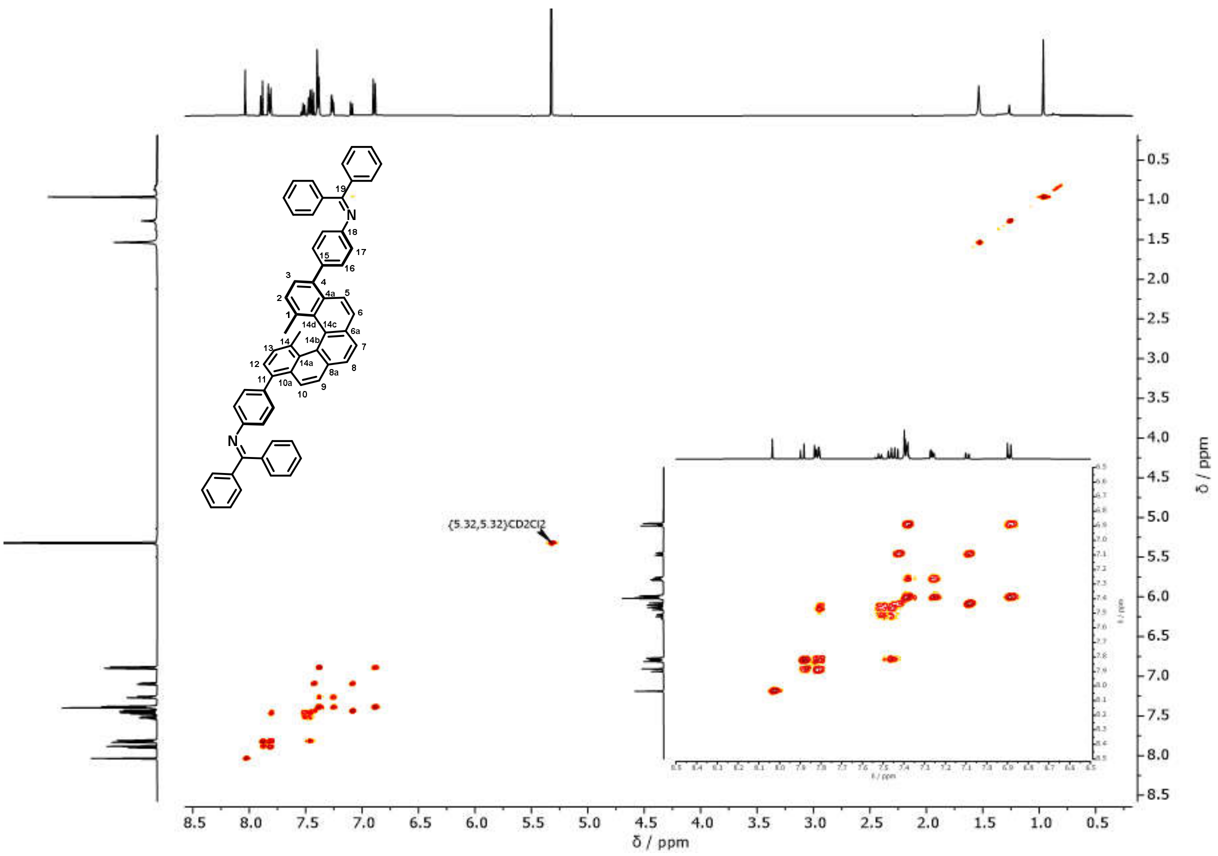

Figure S17. ^{1}H,^{1}H-COSY NMR (500 MHz, 298 K) spectrum of (*P*)-(–)-**3** in CD_2Cl_2.

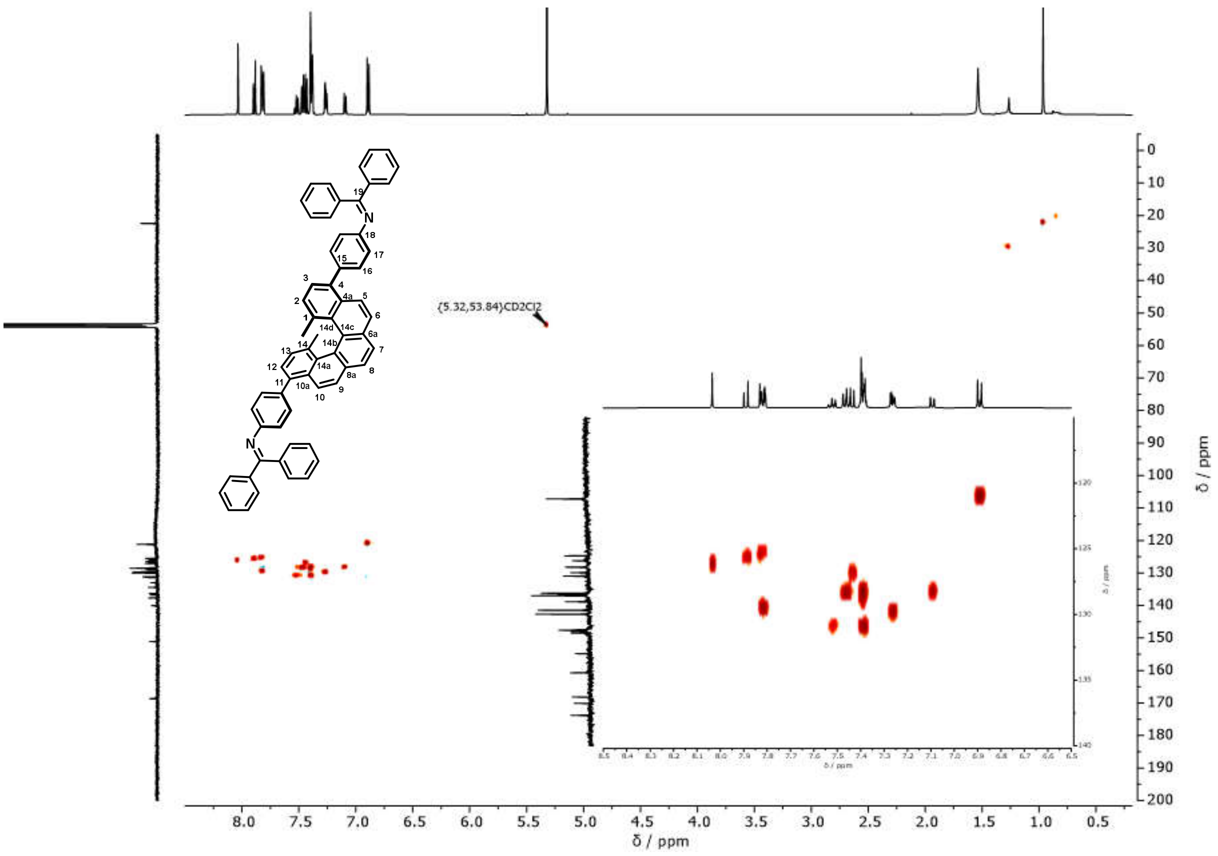

Figure S18. ^{1}H,^{13}C-HSQC NMR (500, 126 MHz, 298 K) spectrum of (*P*)-(–)-**3** in CD_2Cl_2.

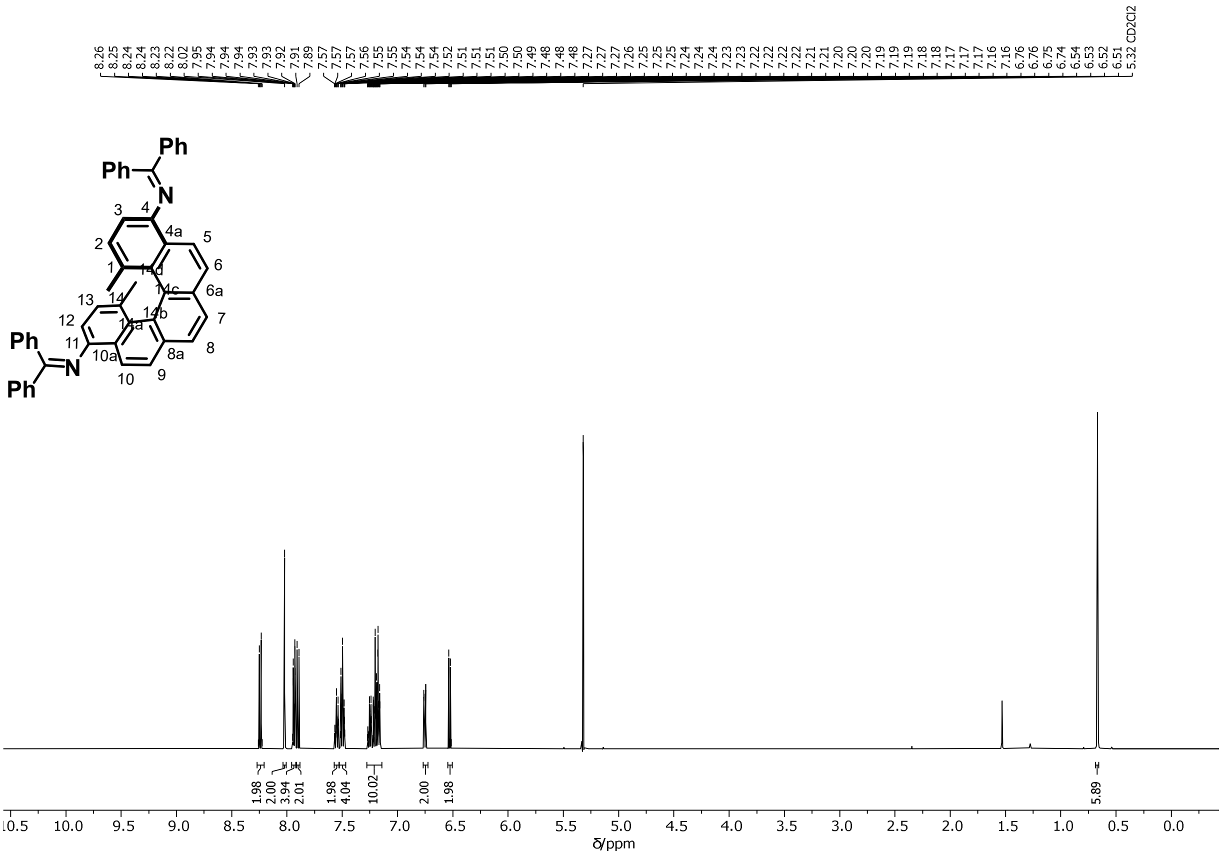

Figure S19. ^1H NMR (500 MHz, 298 K) spectrum of (±)-**6** in CD_2Cl_2.

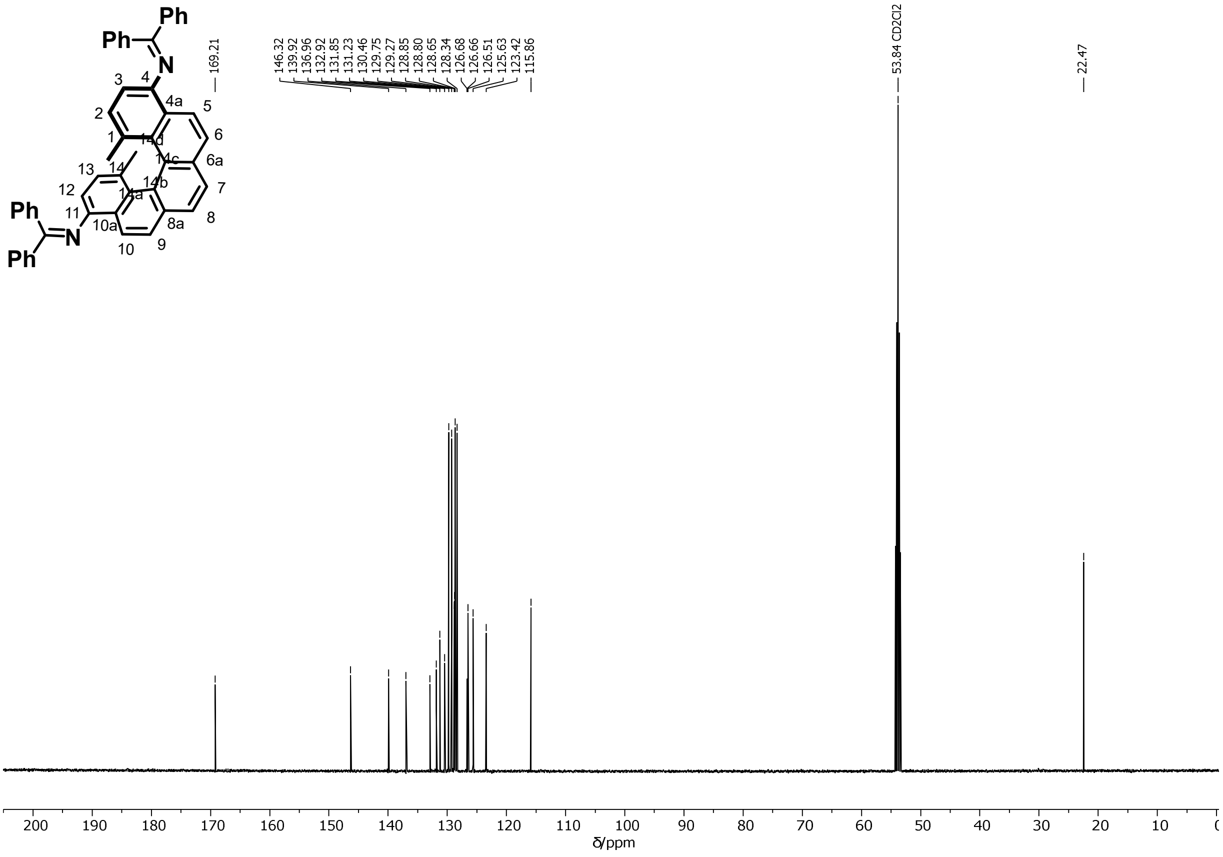

Figure S20. ^{13}C NMR (126 MHz, 298 K) spectrum of (±)-**6** in CD_2Cl_2.

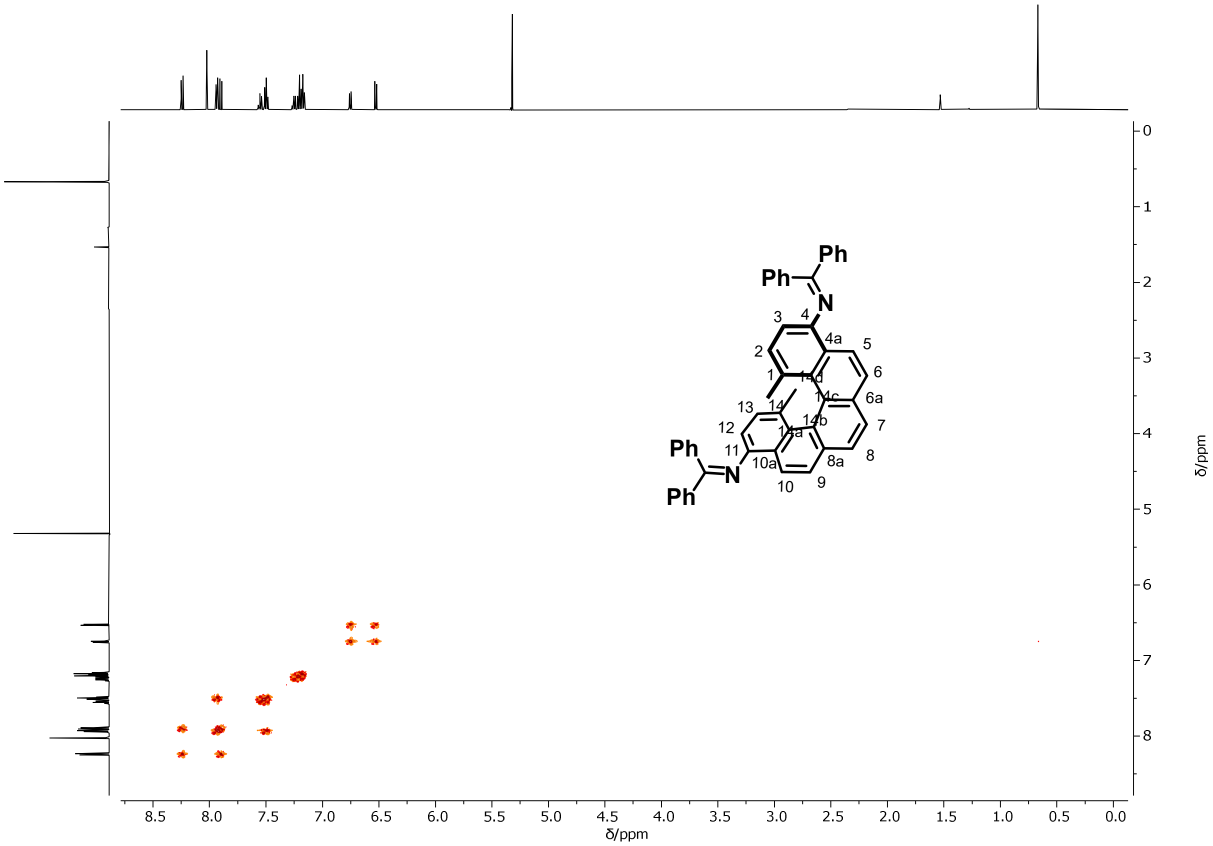

Figure S21. ^{1}H, ^{1}H-COSY NMR (500 MHz, 298 K) spectrum of (±)-**6** in CD_2Cl_2.

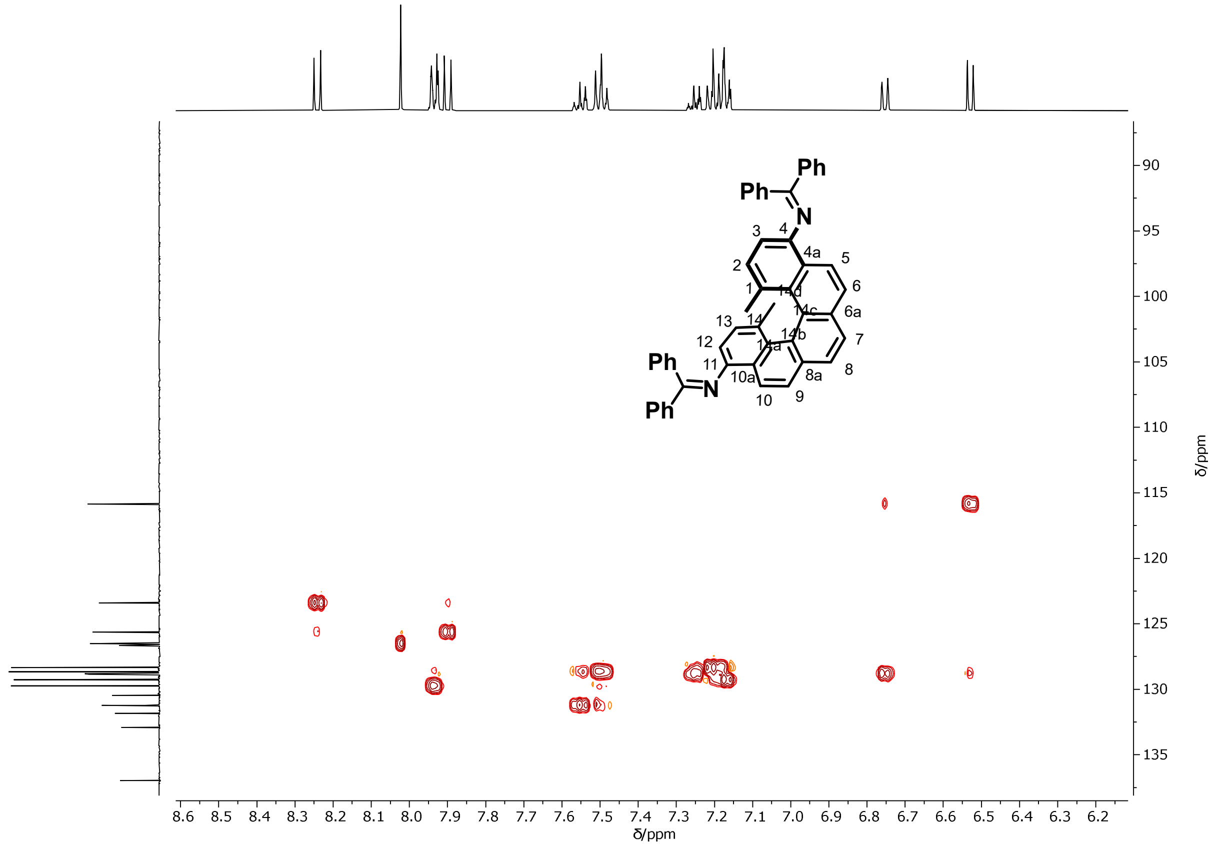

Figure S22. ^{1}H, ^{13}C-HSQC NMR (500 MHz, 126 MHz, 298 K) spectrum of (±)-**6** in CD_2Cl_2.

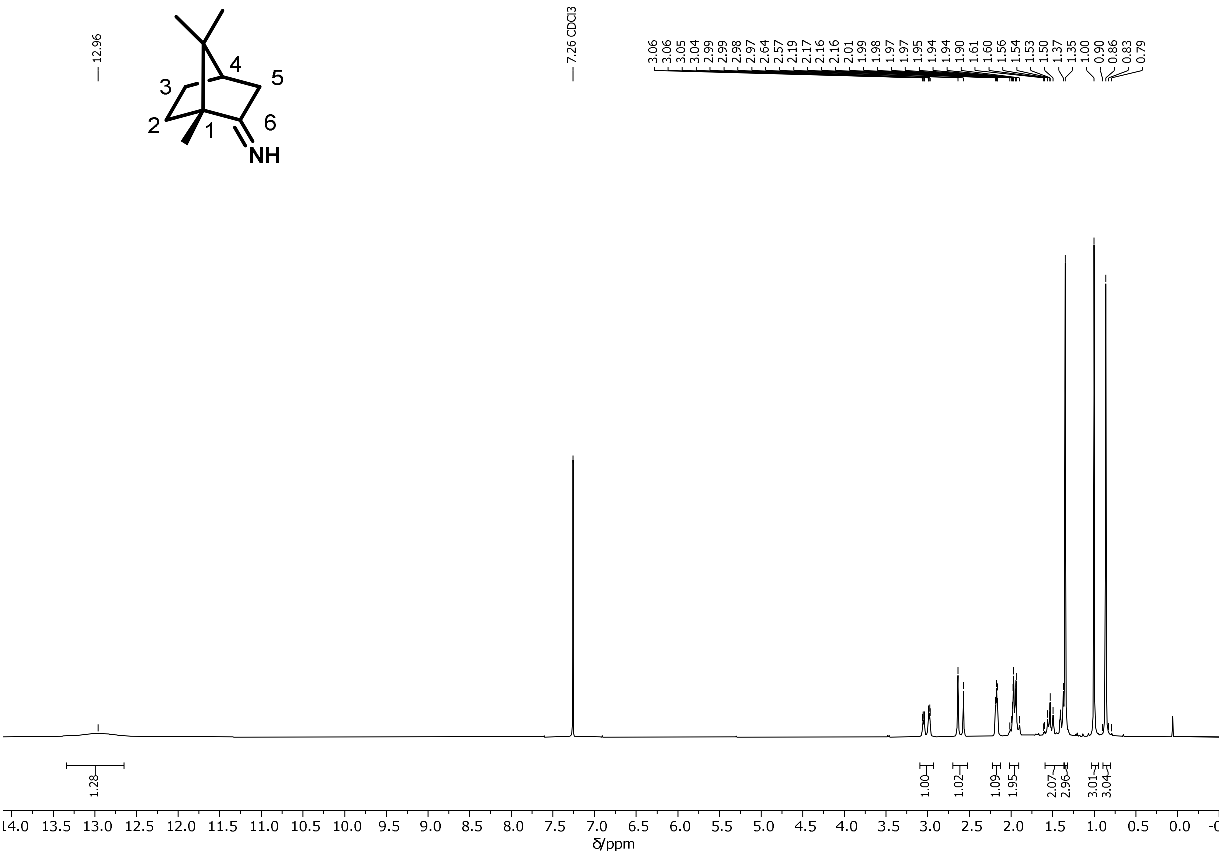

Figure S23. ^{1}H NMR (500 MHz, 298 K) spectrum of (*R*)-camphor imine in $CDCl_3$.

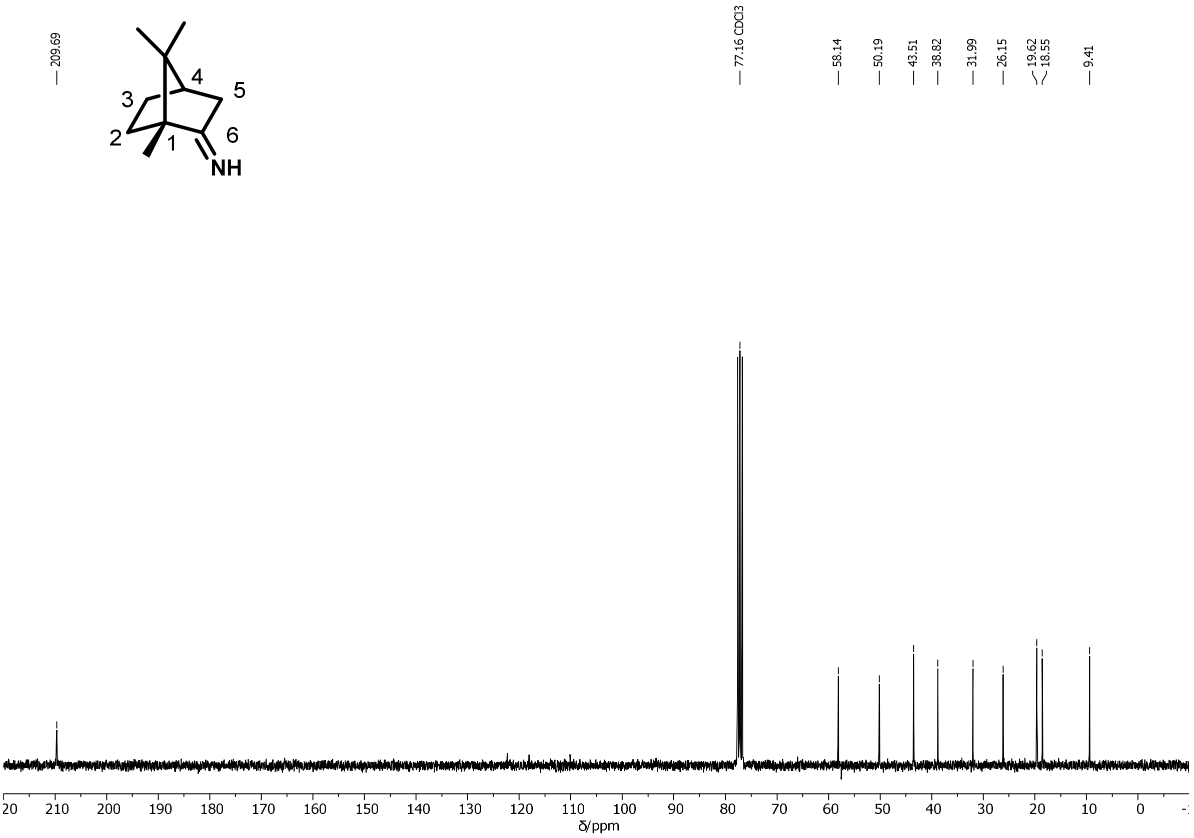

Figure S24. ^{13}C NMR (126 MHz, 298 K) spectrum of (*R*)-camphor imine in $CDCl_3$.

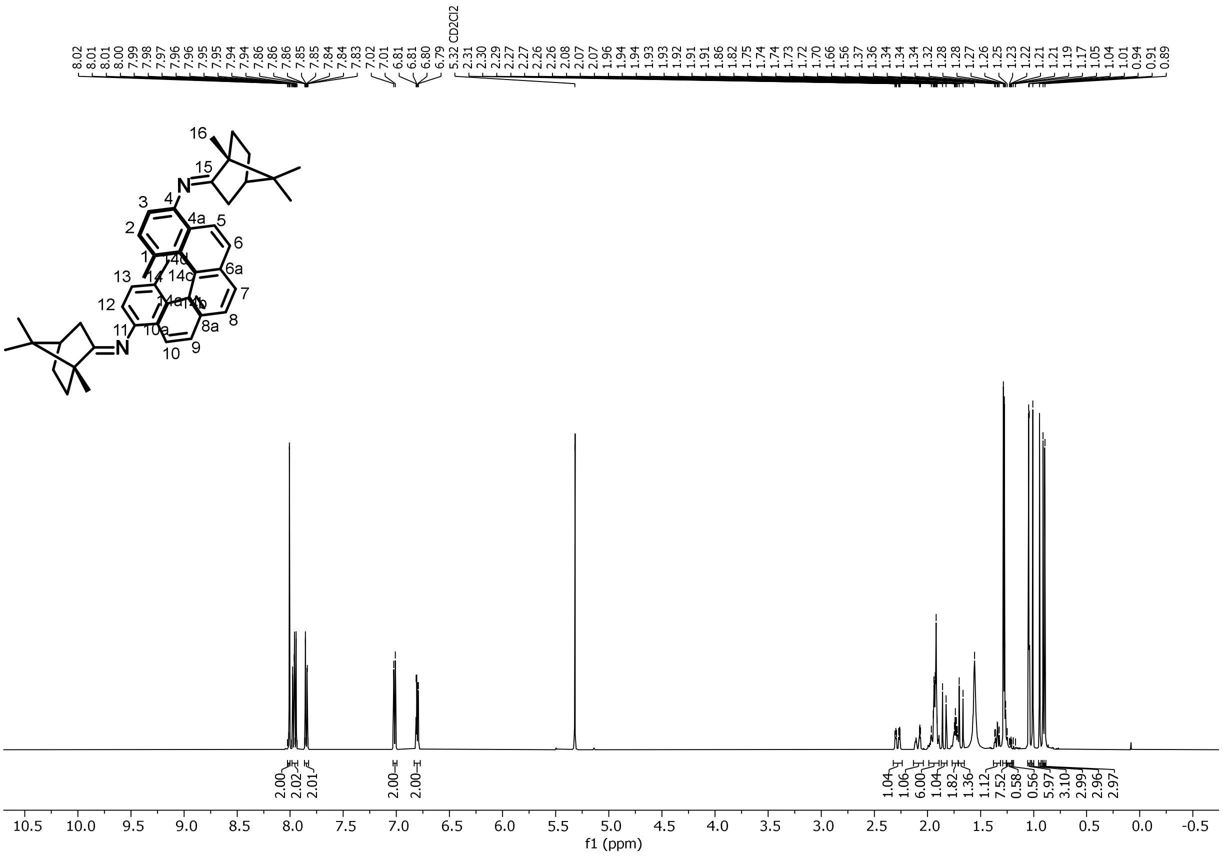

Figure S25. ^{1}H NMR (500 MHz, 298 K) spectrum of (*R*,*R*)-(*P*/*M*)-**7** in CD_2Cl_2.

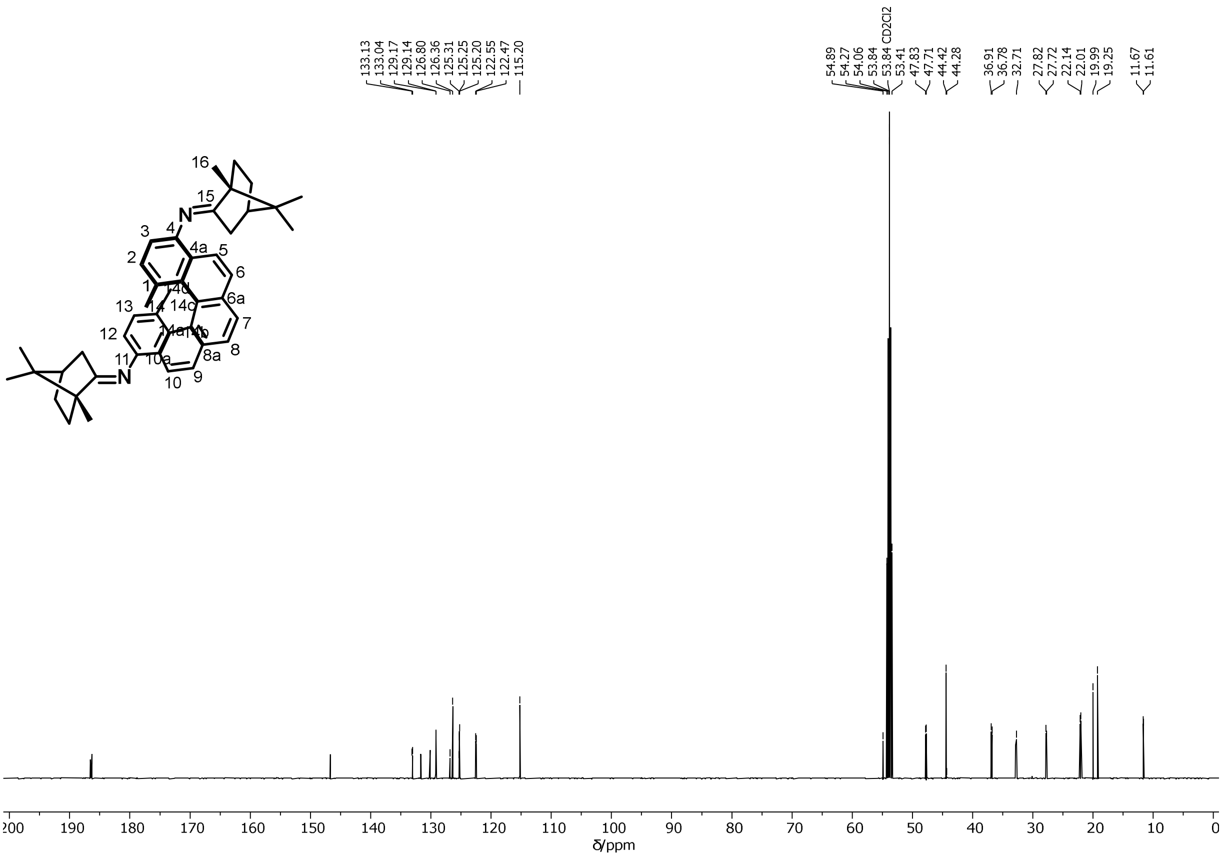

Figure S26. ^{13}C NMR (126 MHz, 298 K) spectrum of (*R*,*R*)-(*P*/*M*)-**7** in CD_2Cl_2.

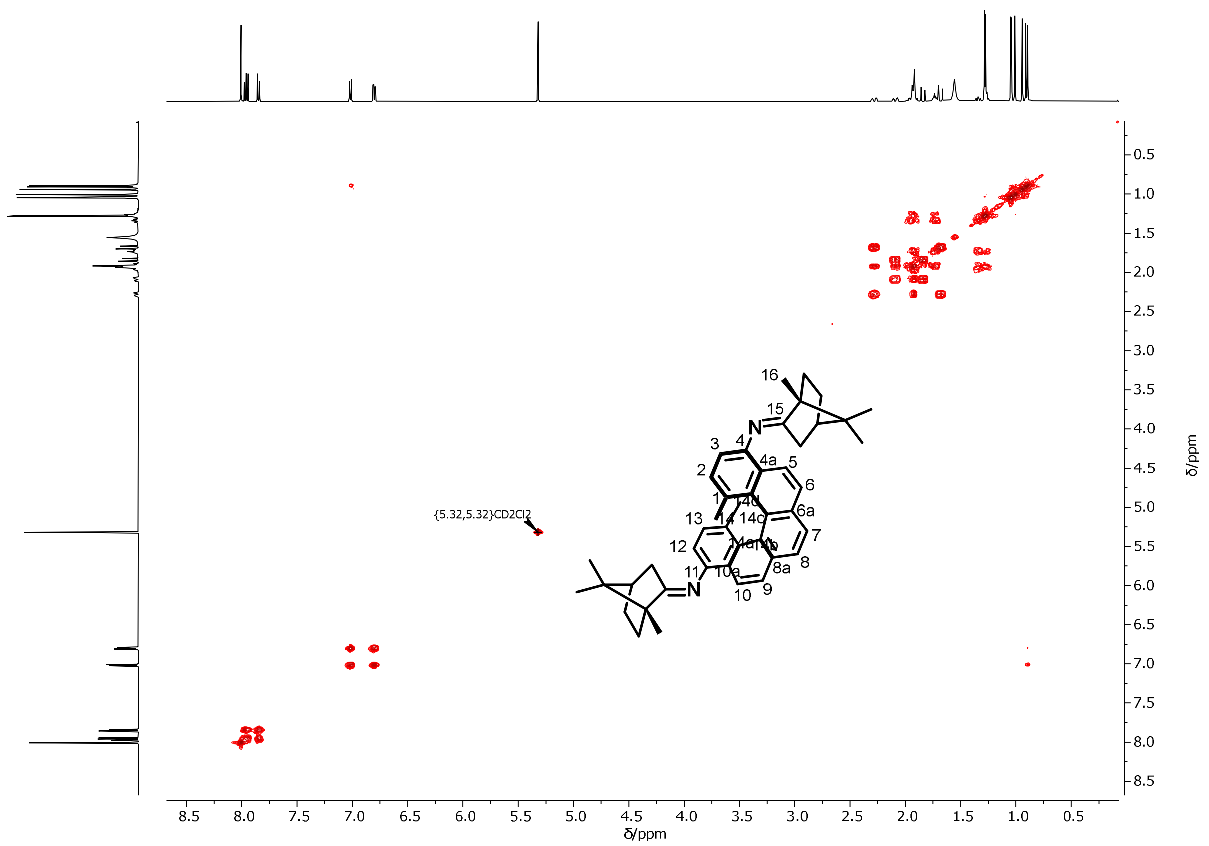

Figure S27. ^{1}H, ^{1}H-COSY NMR (500 MHz, 298 K) spectrum of (*R*,*R*)-(*P*/*M*)-**7** in CD_2Cl_2.

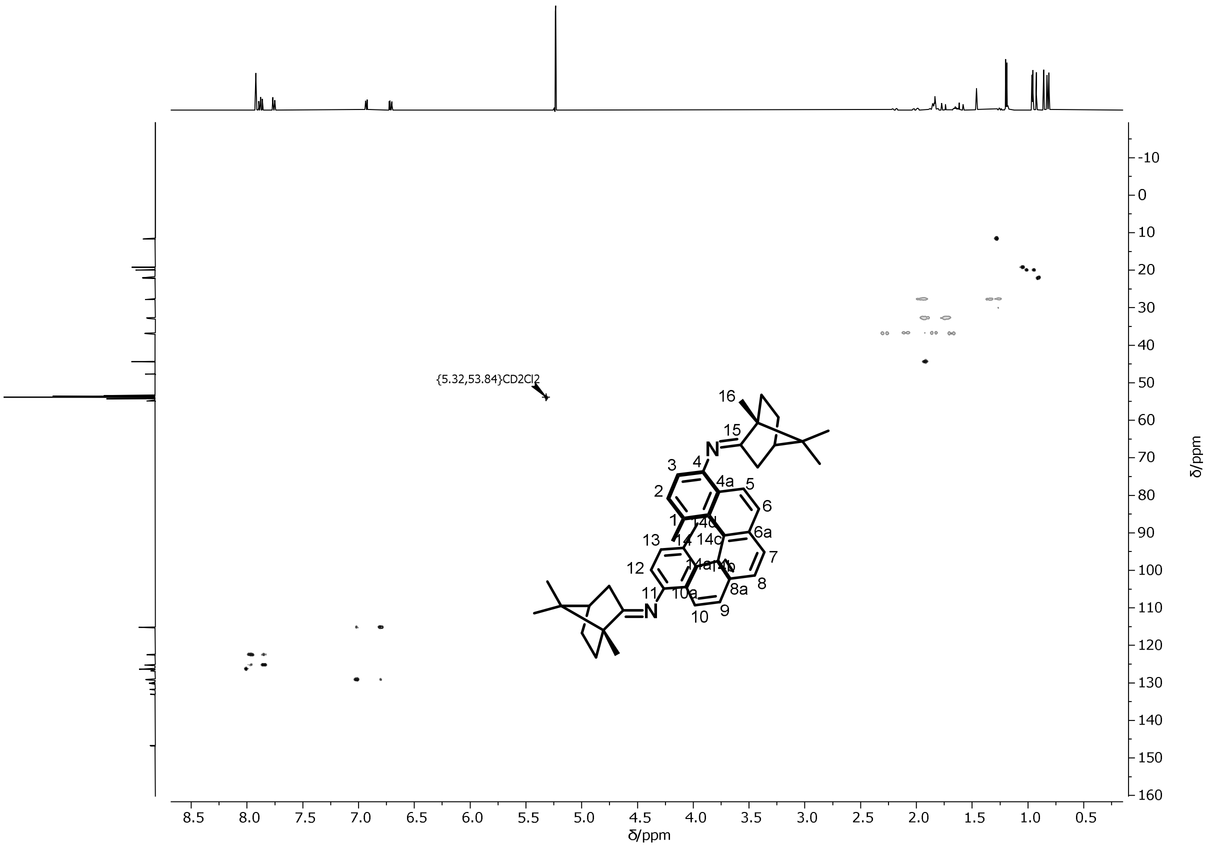

Figure S28. ^{1}H, ^{13}C-HSQC NMR (500 MHz, 126 MHz, 298 K) spectrum of (*R*,*R*)-(*P*/*M*)-**7** in CD_2Cl_2.

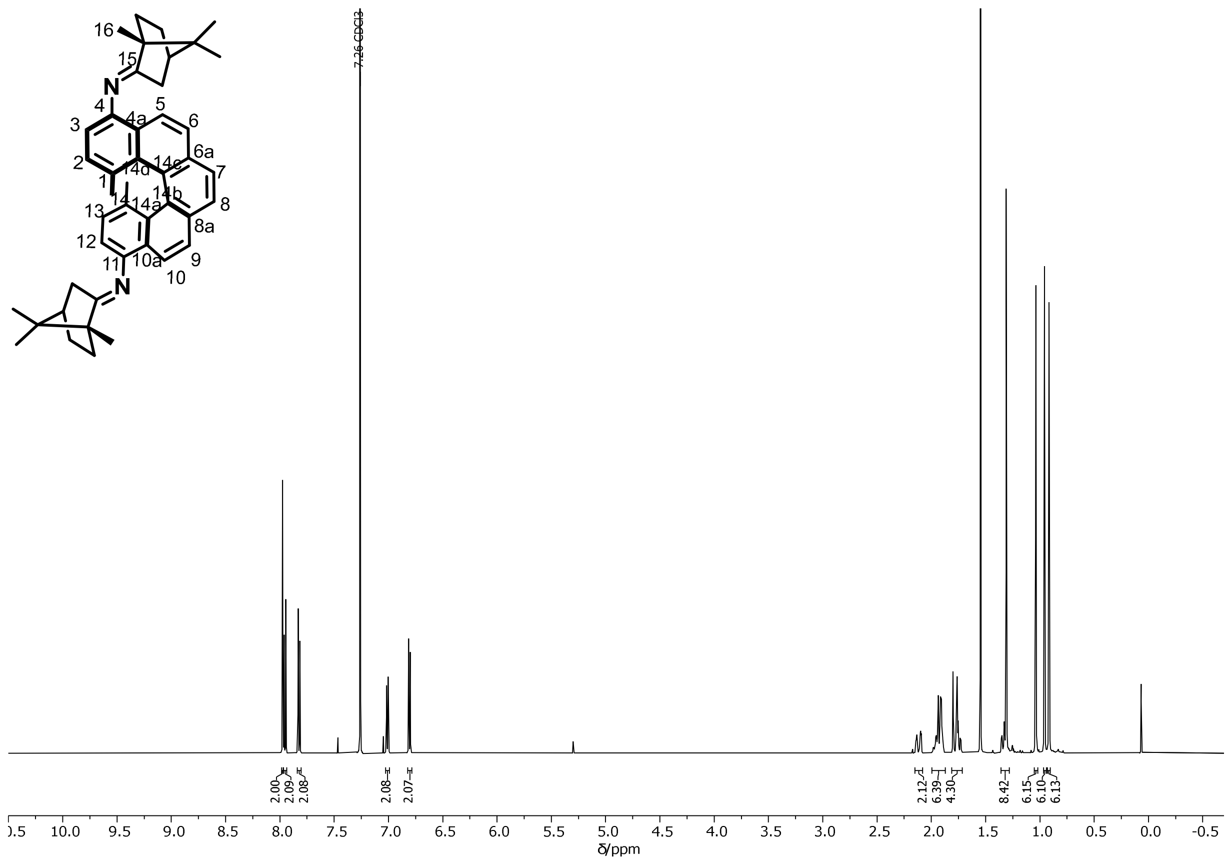

Figure S29. ^{1}H NMR (500 MHz, 298 K) spectrum of (*R*,*R*)-(*P*)-(+)-**7** in $CDCl_3$.

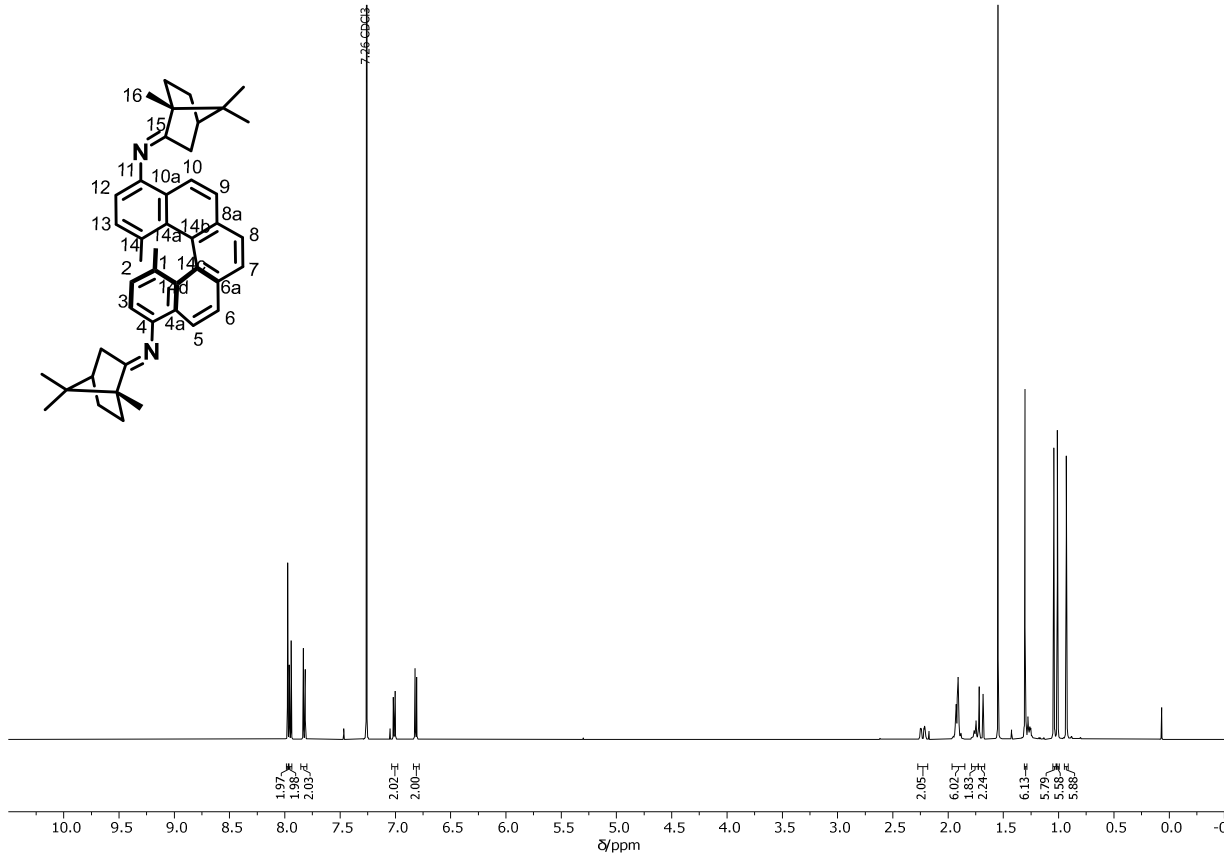

Figure S30. ^{1}H NMR (500 MHz, 298 K) spectrum of (*R*,*R*)-(*M*)-(–)-**7** in $CDCl_3$.

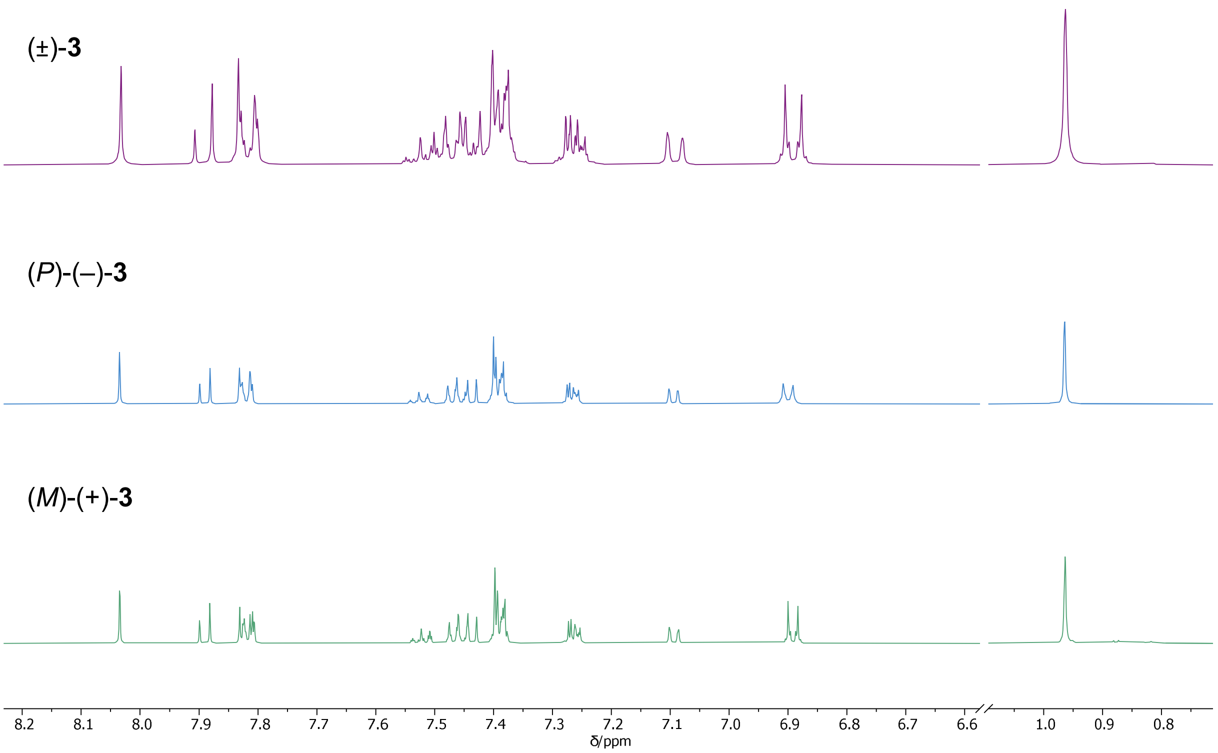

Figure S31. ^{1}H NMR (500 MHz, 298 K) spectra comparison of (±)-**3** (purple), (*P*)-(–)-**3** (blue), and (*M*)-(+)-**3** (green) at 10 mM in CD_2Cl_2. The differences in NMR signals between the racemic mixture and the enantiopure compounds may origin from their specific non-covalent interactions and the varying residual amount of water in the solution.

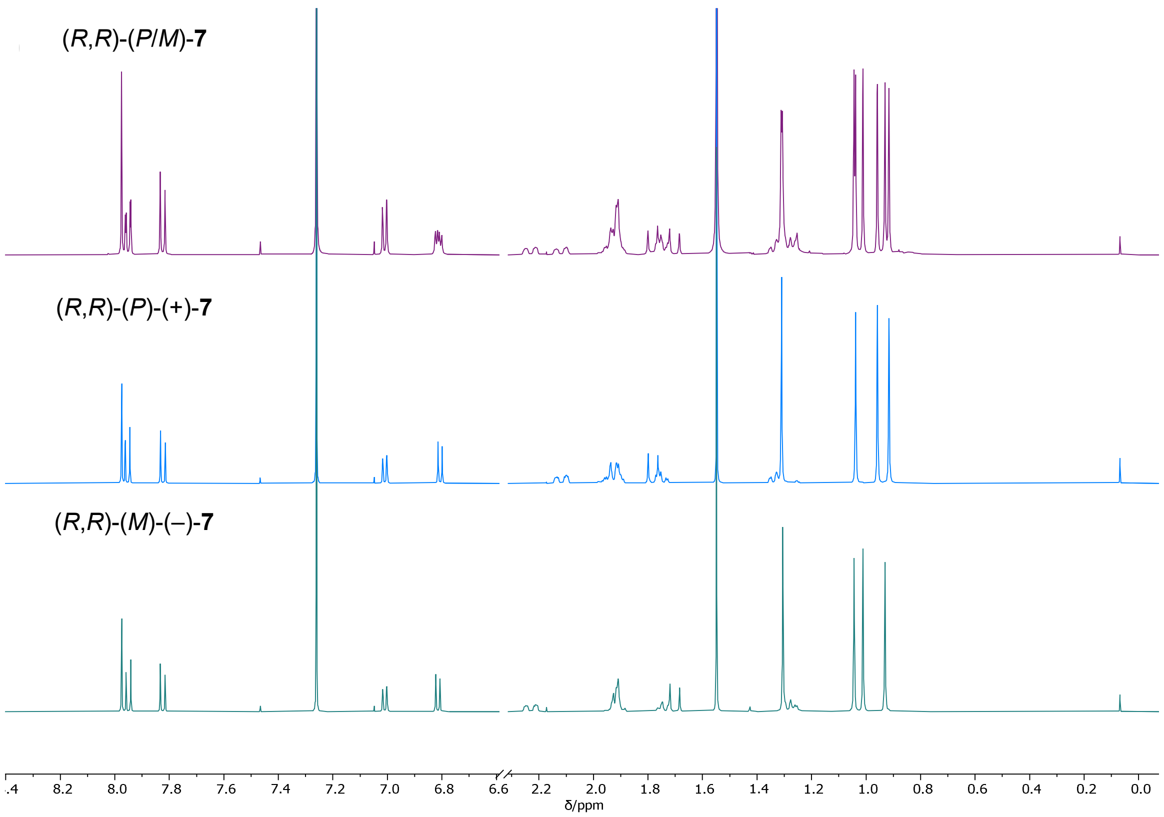

Figure S32. ^{1}H NMR (500 MHz, 298 K) spectra comparison of (*R*,*R*)-(*P*/*M*)-**7** (purple), (*R*,*R*)-(*P*)-(+)-**7** (blue), and (*R*,*R*)-(*M*)-(–)-**7** (green) at 30 mM in $CDCl_3$.

S4. Synthesis of COFs and POPs

rac-[5]Heli-TFB COF

The *pseudo*-linear linker (±)-**3** (23 mg, 27 mmol) and node TFB (3 mg, 18 mmol) were added in a Pyrex tube (30 cm length, inner diameter 0.8 mm), and were sonicated for 5 min. Followed by the addition of *o*-DCB (240 µL) and the yellow suspension was sonicated for 5 min. Then, aqueous acetic acid (64 µL, 6 M) was added, and the suspension was sonicated for 5 min. Next, *n*-BuOH (240 µL) was added, and the mixture was sonicated for 5 min. Then, the mixture was degassed by three freeze–pump–thaw cycles and evacuated to an internal pressure below 0.01 mbar. The tube was flame–sealed with a length of 10 cm, and it was allowed to heat to 25 °C. The tube was immersed in a sand bath and heated at 120 °C for 72 h inside of a pre-heated electric oven without disturbance (natural convection, no fan). The mixture was allowed to cool at 20 °C, and the precipitate was transferred to a funnel equipped with a 45 µm PTFE filter. The resulting solid was washed with hot acetone (3×25 mL), and hot methanol (3×25 mL), while making sure that the solid never ran dry, the sample was transferred to a pre-wetted tea bag, sealed with a staple, and soaked in methanol for 24 h. Subsequently, the sample was dried with $scCO_2$ for 20 min, to yield *rac*-**[5]Heli-TFB** COF as an intense yellow powder (7.8 mg, 69%). Reproducibility was confirmed in 3 repetitions, yielding the same BET surface area (±10%).

The identical experimental conditions were tested with the enantiopure building blocks (*P*)-(–)-**3** and (*M*)-(+)-**3**, yielding only amorphous and non-porous materials in both cases (Figure S34).

Screening experimental conditions for *rac*-**[5]Heli-TFB** COF

In order to achieve high-quality porous and crystalline samples of *rac*-**[5]Heli-TFB** COF, we screened different concentrations, solvent mixtures, acid equivalents, and the presence of modulators. The determination of BET surface area and crystallinity through PXRD served as the main criteria for identifying the optimal reaction conditions. The results are summarized in Table S1.

Table S1. Screening experimental conditions for *rac*-**[5]Heli-TFB** COF.

Entry	Internal code	TFB [mmol L^{-1}]	Solvent mixture (ratio)	AcOH 6M [equiv]	Appearance	Yield	S_{BET} [m^2 g^{-1}]	PXRD [2θ]
1	MJO-229	40	o-DCB/n-BuOH (1:1)	20	powder	52%	366	2.4 4.7
2	MJO-230	40	1,4-dioxane/mesitylene (1:1)	20	flakes	55%	35	amorphous
3	MJO-236	81	1,4-dioxane/mesitylene (1:1)	10	flakes	55%	48	amorphous
4	MJO-237	81	o-DCB/n-BuOH (1:1)	10	solid	66%	137	amorphous
5	MJO-238	81	o-DCB/n-BuOH (1:1)	100	solid	92%	131	2.4, 4.7
6	MJO-255	40	o-DCB/n-BuOH (1:1)	40	flakes	52%	297	2.4, 4.7
7	MJO-257	60	o-DCB/n-BuOH (1:1)	20	powder	69%	504	2.4, 4.7 intense
8	MJO-258	40	o-DCB/n-BuOH (4:1)	20	powder	94%	94	2.4
9	MJO-259	40	o-DCB/n-BuOH (1:1)[a]	20	powder	42%	non-porous	amorphous

[a] 1.5 equiv of aniline (0.7 M in o-DCB).

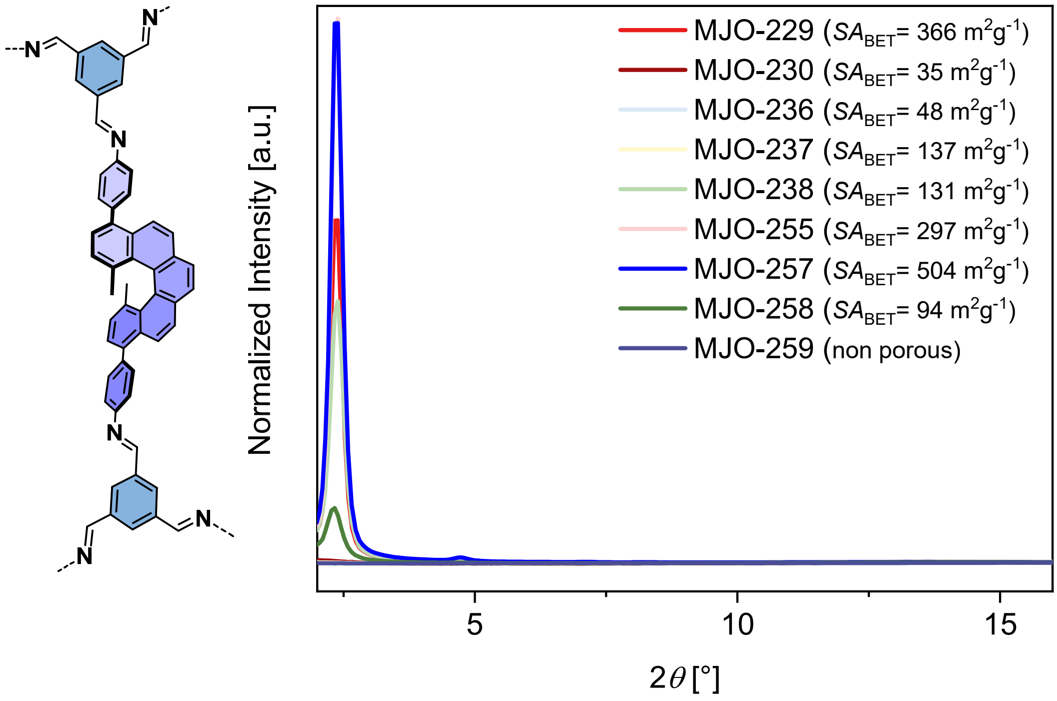

Figure S33. PXRD patterns of screening experimental conditions for *rac*-**[5]Heli-TFB** COF.

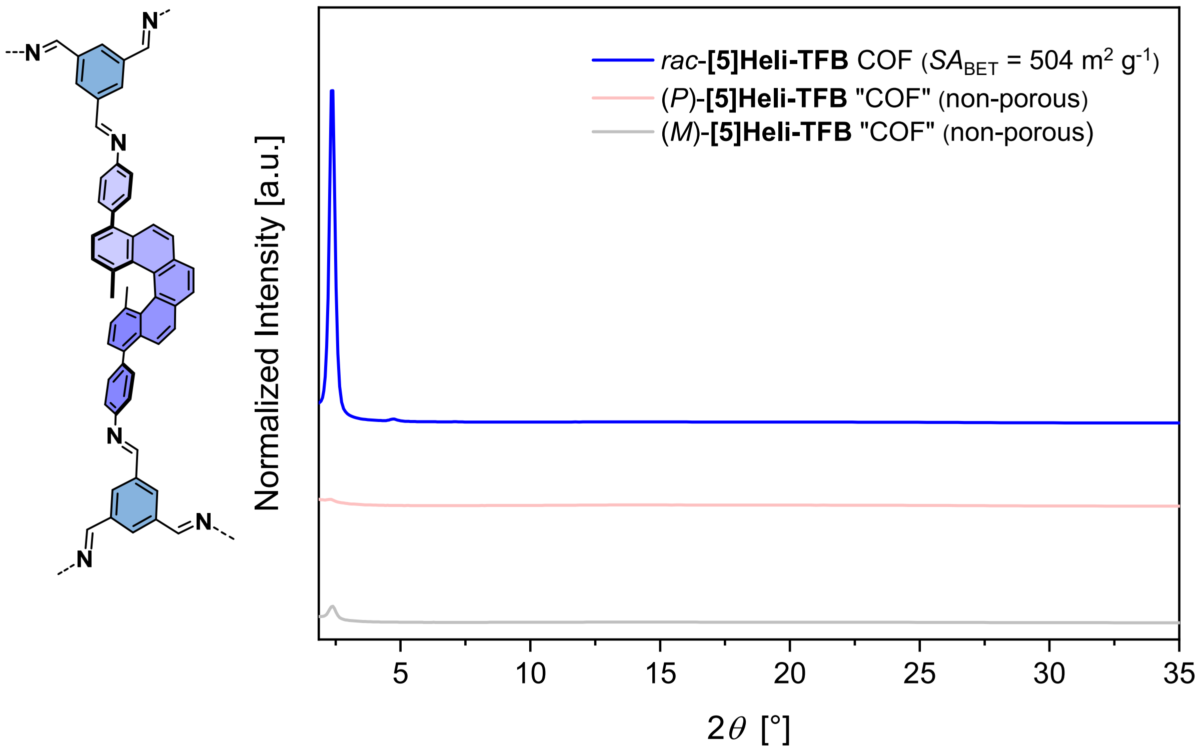

Figure S34. Experimental PXRD patterns of *rac*-**[5]Heli-TFB** COF (blue line), and the attempts for making (*P*)-**[5]Heli-TFB** "COF" (pink line), (*M*)-**[5]Heli-TFB** "COF" (gray line). The resulting materials from enantiopure building blocks (*P*)-(–)-**3** and (*M*)-(+)-**3** are amorphous and cannot be classified as COFs.

rac-[5]Heli-TFP COF, (*P*)-[5]Heli-TFP COF or (*M*)-[5]Heli-TFP COF

Using a Pyrex tube (30 cm length, inner diameter 0.8 mm), the node 1,3,5-triformylphloroglucinol (TFP, 3 mg, 0.014 mmol) and aniline (30 uL, 0.7 M in *o*-DCB) were suspended in *o*-DCB (60 µL), and sonicated for 5 min. Then, aqueous acetic acid (94 µL, 6 M) was added and the suspension was sonicated for 5 min. Then, the linker (±)-**3** (17 mg, 0.021 mmol) was added to reaction mixture, followed by the addition of *o*-DCB (180 µL), and the mixture was sonicated for 5 min. The mixture was degassed by three freeze–pump–thaw cycles and evacuated to an internal pressure below 0.01 mbar. The tube was flame–sealed (after taking it out from a liquid N_2 bath) at a length of 10 cm, and it was allowed to warm to 25 °C. The tube was immersed in a sand bath, and heated at 120 °C for 72 h inside of a pre-heated electric oven (natural convection, no fan) without mechanical disturbance. The mixture was allowed to cool to 20 °C and the precipitate was transferred to a funnel equipped with a 45 µm PTFE filter. The resulting solid was washed with hot acetone (3×25 mL), and hot methanol (3×25 mL), while making sure that the solid never ran dry, the sample was transferred to a pre-wetted (in MeOH) tea bag, sealed with a staple, and soaked in methanol for 24 h. Subsequently, the sample was dried with $scCO_2$ for 20 min, to yield *rac*-**[5]Heli-TFP** COF as intense yellow powder (9 mg, 96%). Reproducibility was confirmed in 3 independent repetitions starting from different synthetic batches of (±)-**3**, yielding a similar BET surface area

(±10%). The same conditions were employed for the enantiopure building blocks (*P*)-(–)-**3** and (*M*)-(+)-**3**, yielding a crystalline and porous material (*P*)-**[5]-Heli-TFP** COF (9 mg, 96%) and (*M*)-**[5]-Heli-TFP** COF (8 mg, 90%).

In order to achieve high-quality porous and crystalline samples of *rac*-**[5]Heli-TFP** COF, we screened different concentrations, solvent mixtures, acid equivalents, acid concentration, and the presence of modulators. The determination of BET surface area and crystallinity through PXRD served as the main criteria for identifying the optimal reaction conditions (Figure S35). The results are summarized in Table S2.

Table S2. Screening experimental conditions for *rac*-**[5]Heli-TFP** COF.

Entry	Internal code	TFP [mmol L^{-1}]	Solvent mixture (ratio)	AcOH 6M [equiv]	Appearance	Yield	S_{BET} [m^2 g^{-1}]	PXRD [2θ]
1	MJO-261	40	o-DCB/n-BuOH (1:1)	40[c]	powder	99%	non-porous	amorphous
2	MJO-262	40	o-DCB/n-BuOH (1:1)	40	powder	99%	non-porous	amorphous
3	MJO-263	40	1,4-dioxane/mesitylene (4:1)[a]	40[c]	flakes	43%	non-porous	2.4
4	MJO-264	40	o-DCB/n-BuOH (1:1)[a]	40	powder	97%	256	2.4, 4.2
5	MJO-270	40	o-DCB/n-BuOH (1:4)[a]	40	powder	99%	non-porous	2.4
6	MJO-271	40	o-DCB/n-BuOH (4:1)[a]	40	powder	99%	782	2.4, 4.2, 4,8, 8.5
7	MJO-272	40	o-DCB/n-BuOH (1:1)[a]	80	flakes	97%	non-porous	2.4, 4.2, 4,8, 8.5
8	MJO-273	40	o-DCB/n-BuOH (1:1)[b]	40	flakes	99%	110	2.4, 4.2, 4,8, 8.5
9	MJO-275	60	o-DCB/n-BuOH (4:1)[a]	40	flakes	97%	712	2.4, 4.2, 4,8, 8.5

[a] 1.5 equiv of aniline (0.7 M in *o*-DCB); [b] 3.0 equiv of aniline (0.7 M in *o*-DCB); [c] 10 M aq. AcOH.

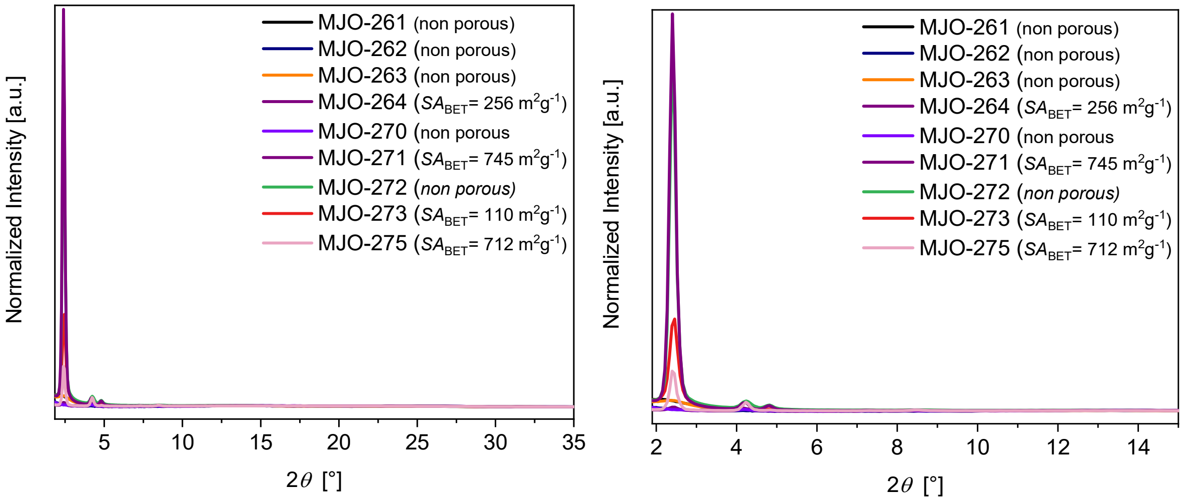

Figure S35. PXRD patterns of screening experimental conditions for *rac*-**[5]Heli-TFP** COF. The right graph is an enlargement of the abscissa from 1.9° to 5°.

In-situ synthesis of *rac*-[5]Heli-TFP COF film on quartz substrate

The linker (±)-**3** (5.8 mg, 7 μmol), aniline (10.1 μL from a solution 0.7 M in *o*-DCB), and node TFP (4.7 μmol, 1.0 mg) were suspended in a 25 mL Schlenk tube and dissolved for 5 min with a mixture of *o*-DCB/*n*-BuOH 4:1 (3.14 mL). Aqueous acetic acid (499 μL, 6 M) was added, and the suspension was sonicated for 5 min. The previously ozone-cleaned quartz substrate was added to the reaction mixture in a horizontal position. The mixture was degassed by three freeze–pump–thaw cycles. The Schlenk tube was evacuated to an internal pressure below 0.01 mbar and heated at 120 °C in a pre-heated oil bath without disturbance for 72 h. The mixture was allowed to cool to 20 °C, and the substrate was soaked in acetone (2×10 mL, 10 min), methanol (2×10 mL, 10 min), *i*-PrOH (2×10 mL, 10 min), and dried in a stream of argon gas for 10 min.

In order to achieve homogeneous and crystalline samples of *rac*-**[5]Heli-TFP** COF films, different reaction mixture concentrations of (±)-**3**, and aq. AcOH (6 M) parts in the solution (%*v*/*v*) were tested. The films were analyzed by laser-microscopy images, crystallinity through PXRD, and photoluminescence emission were the main criteria for identifying optimal reaction conditions (Figure S37–S38). The results are summarized in Table S3.

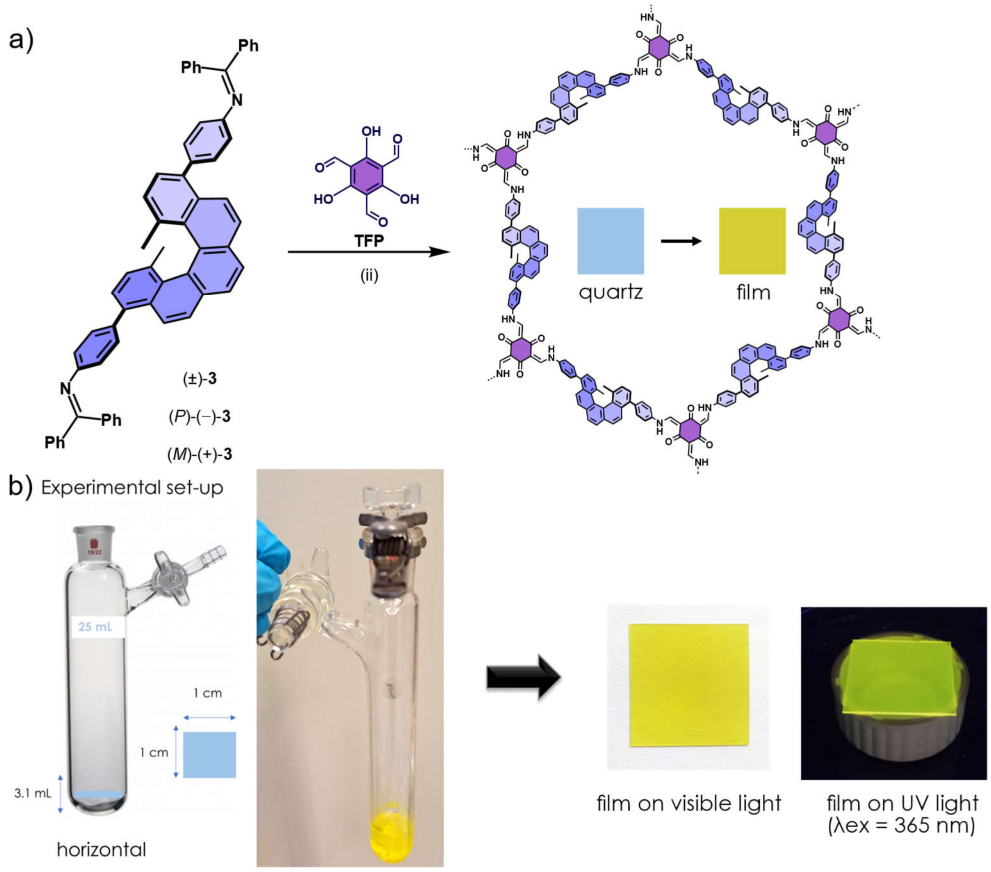

Figure S36. Experimental set up for the in-situ formation of homochiral and racemic **[5]Heli-TFP** COF films over plasma-activated quartz-substrates with different concentrations of (±)-**3**; (a) reaction conditions for the in-situ synthesis of [5]Heli-TFP COF; (b) experimental set-up for the deposition of homogeneous films.

Table S3. Screening experimental conditions for *rac*-**[5]Heli-TFP** COF films

(±)-**3** [mM]	**Equiv.** AcOH [6 M]	**PXRD**	**Solid-state emission**	**Observations**
47*	40 (24% v/v)	active	intense	homogeneous/opaque
23	40 (14% v/v)	---	---	no deposition
23	70 (24% v/v)	active	high	low-homogeneous/opaque
12	162 (24% v/v)	active	medium	medium-homogeneous/opaque
8	247 (24% v/v)	active	medium	medium-homogeneous/transparent
1.2	1270 (24% v/v)	low-active	medium	homogeneous/transparent
0.5	1270 (24% v/v)	non-active	low	non-homogeneous/transparent

*Previously stablished powder-synthesis conditions.

The concentrations of 12 mM and 1.2 mM of (±)-**3** were also tested for the in-situ synthesis of homochiral films by using the enantiopure building blocks (*P*)-(–)-**3** and (*M*)-(+)-**3**, yielding homogenous and crystalline (*P*)-**[5]-Heli-TFP** COF films and (*M*)-**[5]-Heli-TFP** COF films over quartz substrates (Figure S39).

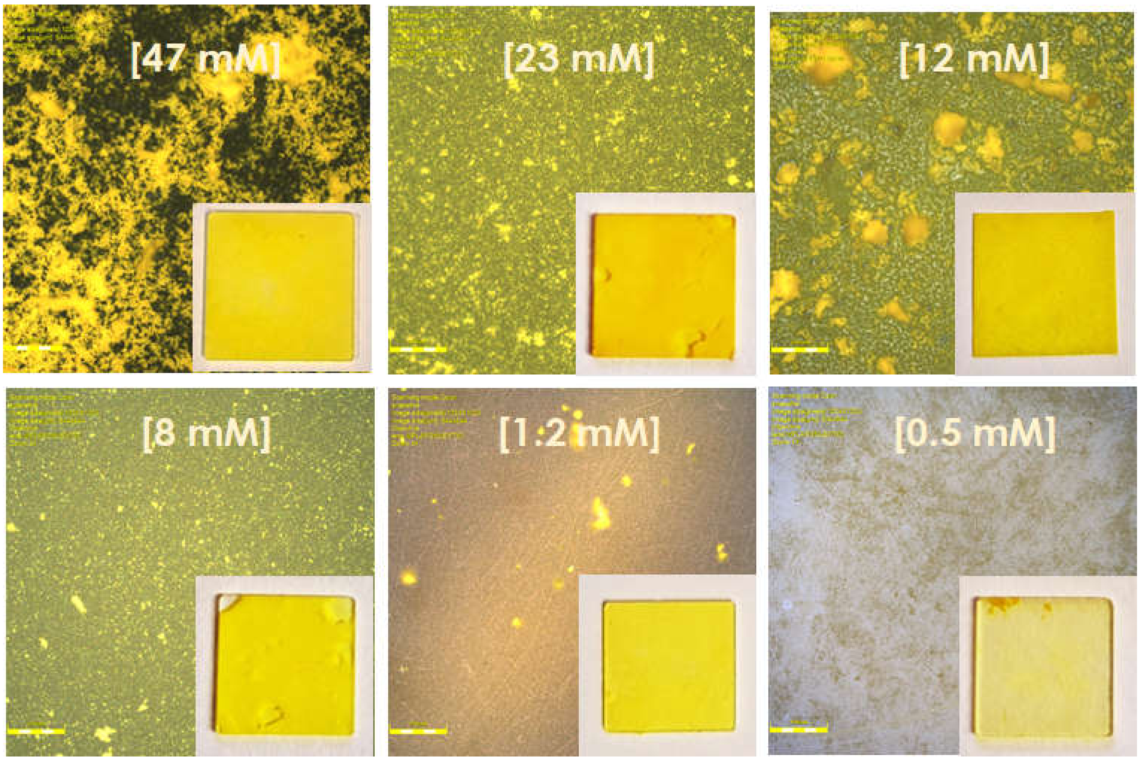

Figure S37. Images of the *rac*-**[5]Heli-TFP** COF films over quartz-substrate with different concentrations of (±)-**3**. Scale identical to main text Figure 7a.

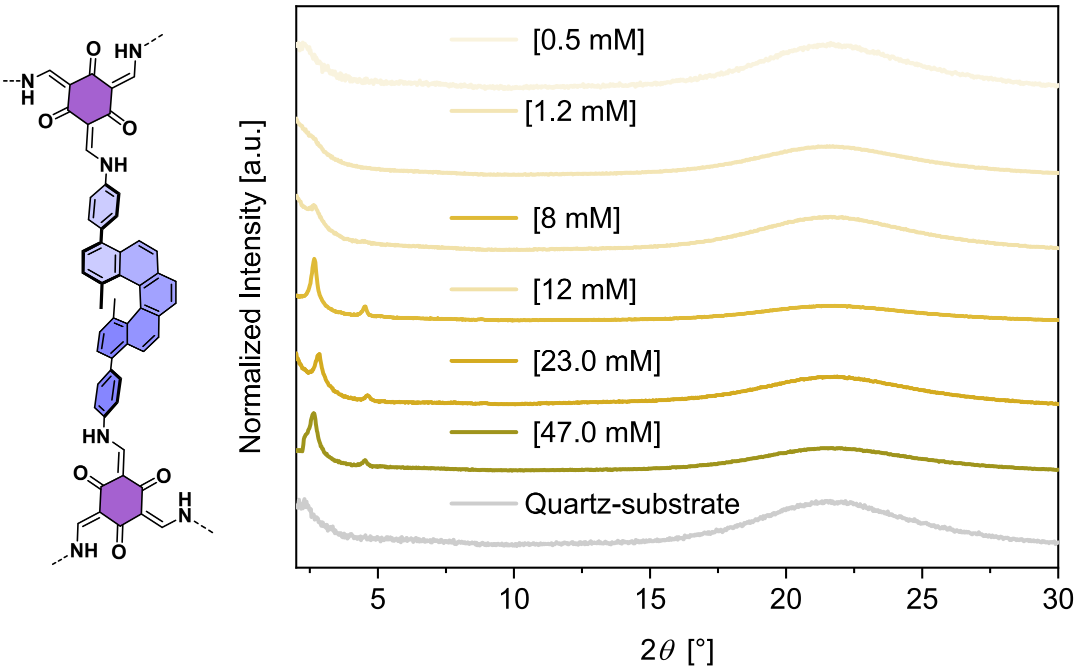

Figure S38. PXRD diffractograms of *rac*-**[5]Heli-TFP** COF films over quartz substrate with a Bragg–Brentano geometry with different concentrations of (±)-**3**.

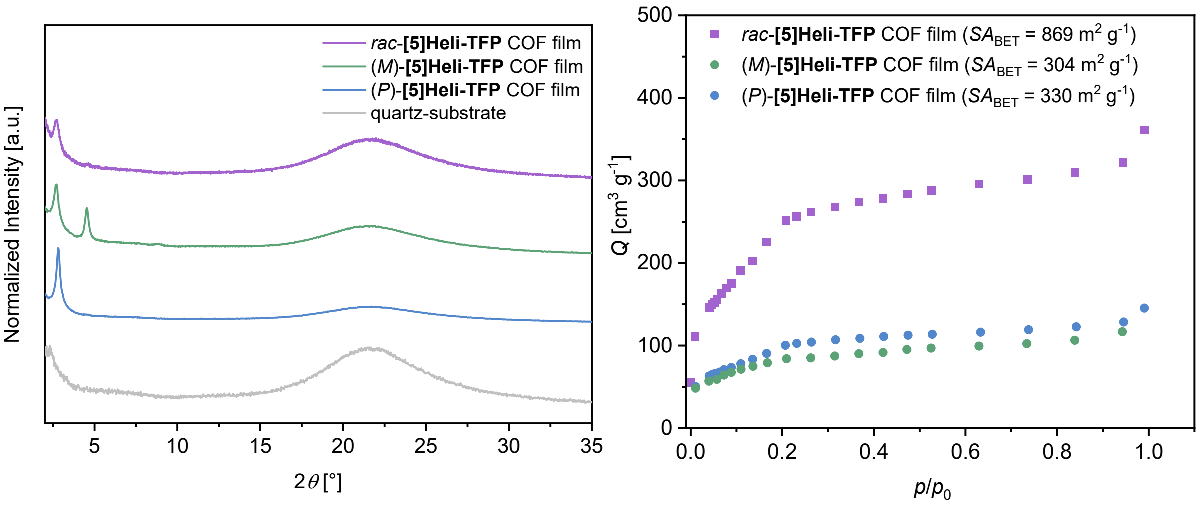

Figure S39. Characterization of homochiral and racemic **[5]Heli-TFP** COF films from the building blocks (*P*)-(−)-**3**, (*M*)-(+)-**3**, and (±)-**3** at 12 mM over quartz substrates; (a) experimental PXRD diffractograms of homochiral and racemic **[5]Heli-TFP** COF films with a Bragg–Brentano geometry; (b) N_2 adsorption isotherms of films at 77 K of *rac*-**[5]Heli-TFP** COF (yellow squares), (*P*)-**[5]Heli-TFP** COF (blue dots) and (*M*)-**[5]Heli-TFP** COF (green dots). The homochiral films were reproduced by duplicate obtaining similar Bragg reflexes and porosity values.

Synthesis of *rac*-**[5]Heli-TFP** POP (amorphous porous polymer) from (±)-**6**

The *pseudo*-linear linker (±)-**6** (87.4 mg, 0.13 mmol) and node TFP (18.6 mg, 0.087 mmol) were added in a Pyrex tube (30 cm length, inner diameter 0.8 mm) and were sonicated for 5 min. Followed by the addition of a mixture of solvents 1,4-dioxane/mesitylene 1:1 (450 µL) and the yellow suspension was sonicated for 5 min. Aqueous acetic acid (84 µL, 10 M) was added and the suspension was sonicated for 5 min. The mixture was degassed by three freeze–pump–thaw cycles and evacuated to an internal pressure below 0.01 mbar. The tube was flame–sealed with a length of 10 cm and allowed to heat to 25 °C. The tube was immersed in a sand bath and heated at 120 °C for 3 days inside a pre-heated electric oven without disturbing (natural convection, no fan). The mixture was allowed to cool at 20 °C and the precipitate was transferred to a funnel equipped with a 45 µm PTFE filter. The resulting solid was washed with hot acetone (3×25 mL) and hot methanol (3×25 mL), while making sure that the solid never ran dry. The sample was transferred to a pre-wetted tea bag, sealed with a staple, and soaked in methanol for 24 h. Subsequently, the sample was dried with $scCO_2$ for 20 min to yield *rac*-**[5]Heli-TFP** POP as a dark red powder (35 mg, 65%). In order to obtain crystalline samples of *rac*-**[5]Heli-TFP** POP, different reaction mixture concentrations, solvent mixtures, acid equivalents, and the presence of modulators were tested (Figure S40). Crystallinity through PXRD served as the main criteria for identifying optimal reaction conditions, unfortunately non-crystalline reflexes were obtained (Table S4).

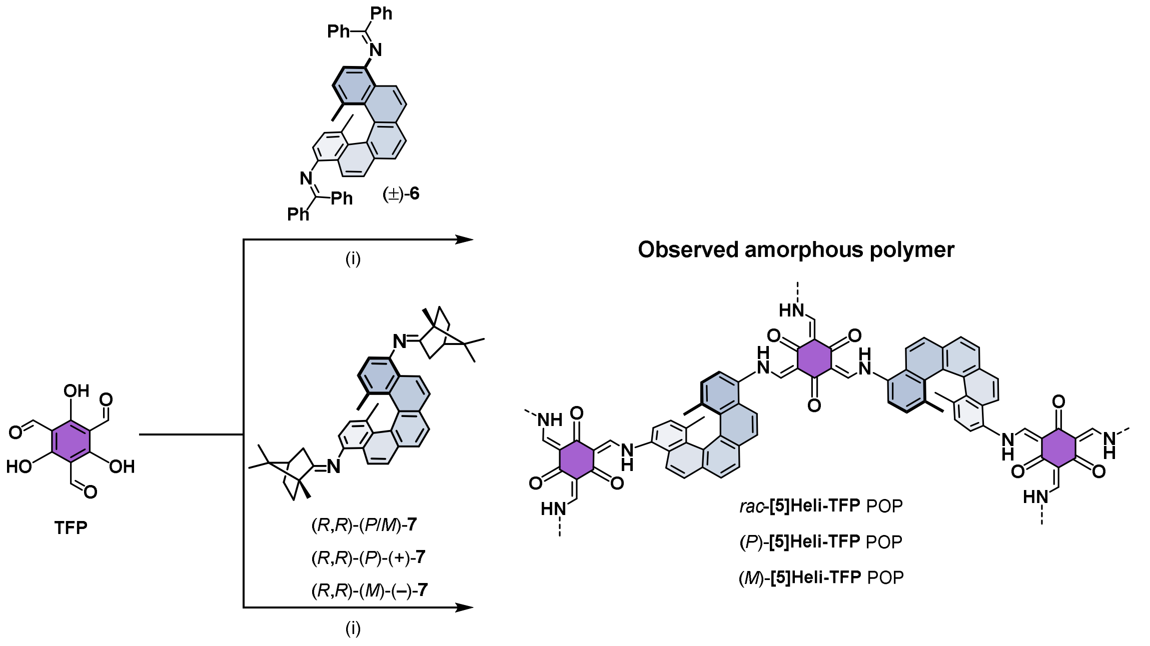

Scheme S3. Target synthesis of racemic and homochiral **[5]Heli-TFP** COF-2, using (±)-**6**, (*R*,*R*)-(*P*/*M*)-**7**, (*R*,*R*)-(*P*)-(+)-**7**, and (*R*,*R*)-(*M*)-(–)-**7** as *pseudo*-C_2 linkers with TFP as node: (i) aq. AcOH (10 M), mesitylene/1,4-dioxane 1:1, 77 mM, 120 °C, 72 h; after the characterization of the products, only amorphous and porous solids were obtained: *rac*-**[5]Heli-TFP** POP-1, (*P*)-**[5]Heli-TFP** POP-1, and (*M*)-**[5]Heli-TFP** POP-1.

Table S4. Screening experimental conditions for *rac*-**[5]Heli-TFP** POP from (±)-**6**.

Entry	Internal code	TFP [mM]	Solvent mixture (ratio)	AcOH (6 M) [equiv]	Appearance	Yield	S_{BET} [$m^2 g^{-1}$]	Crystallinity [2θ]
1	MJO-022	97	1,4-dioxane/mesitylene (1:1)	10	Red flakes	55%	560	Broad reflexes
2	MJO-029	97	4-dioxane/mesitylene (1:1)	10 TFA (6 M)	Red flakes	46%	640	Broad reflexes
3	MJO-101	97	1,4-dioxane/mesitylene (1:1)	10 AcOH (10 M)	Red powder	65%	723	Broad reflexes
4	MJO-211	30	1,4-dioxane/mesitylene (1:1)	21	Red powder	76%	---	Broad reflexes
5	MJO-212	30	o-DCB/n-BuOH (1:1)	21	Red flakes	70%	---	Broad reflexes
6	MJO-243	16	o-DCB/n-BuOH (1:1)	21	Red powder	60%	---	Broad reflexes

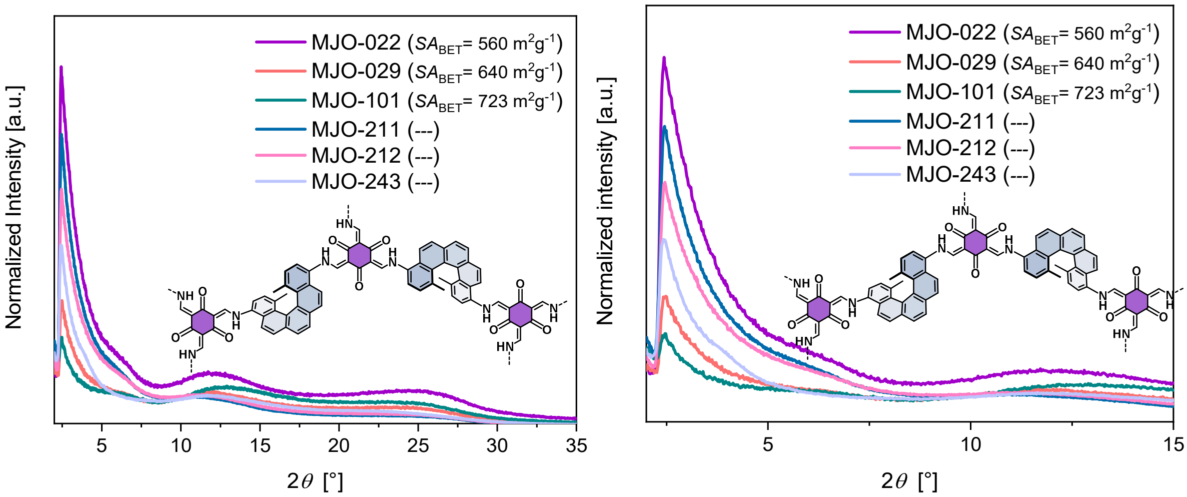

Figure S40. Zoomed-in PXRD patterns of screening experimental conditions for *rac*-**[5]Heli-TFB** POP from (±)-**6**.

Synthesis of *rac*-**[5]Heli-TFP** POP, (*P*)-**[5]Heli-TFP** POP, and (*M*)-**[5]Heli-TFP** POP from (*R*,*R*)-(*P*/*M*)-**7,** (*R*,*R*)-(*P*)-(+)-**7**, and (*R*,*R*)-(*M*)-(–)-**7**.

The *pseudo*-linear linker (*R*,*R*)-(*P*/*M*)-**7** (76.9 mg, 0.126 mmol) and node TFP (18.0 mg, 0.084 mmol), were added in a Pyrex tube (30 cm length, inner diameter 0.8 mm) and were sonicated for 5 min. Followed by the addition of a mixture of solvents 1,4-dioxane/mesitylene 1:1 (548 µL) and the yellow suspension was sonicated for 5 min. Aqueous acetic acid (84 µL, 10 M) was added and the suspension was sonicated for 5 min. The mixture was degassed by three freeze–pump–thaw cycles and evacuated to an internal pressure below 0.01 mbar. The tube was flame–sealed with a length of 10 cm and allowed to heat to 25 °C. The tube was immersed in a sand bath and heated at 120 °C for 3 days inside a pre-heated electric oven without disturbance (natural convection, no fan). The mixture was allowed to cool at 20 °C and the precipitate was transferred to a funnel equipped with a 45 µm PTFE filter. The resulting solid was washed with hot acetone (3×25 mL) and hot methanol (3×25 mL), while making sure that the solid never ran dry. The sample was transferred to a pre-wetted tea bag, sealed with

a staple, and soaked in methanol for 24 h. Subsequently, the sample was dried with $scCO_2$ for 20 min to yield *rac*-**[5]Heli-TFP** POP as a dark red powder (48 mg, 72%, scheme S3). The same conditions were tested with the enantiopure building blocks (*R*,*R*)-(*P*)-(+)-**7** and (*R*,*R*)-(*M*)-(–)-**7**, yielding amorphous and non-porous materials, (45%) and (41%) respectively (Figure S43).

In order to obtain high-quality samples of *rac*-**[5]Heli-TFP** POP, different reaction mixture concentrations, solvent mixtures, acid equivalents, and the presence of modulators were tested (Figure S41). The determination of BET surface area and crystallinity through PXRD served as the main criteria for identifying optimal reaction conditions. The results are summarized in Table S5.

Table S5. Screening conditions for *rac*-**[5]Heli-TFP** POP from (*R*,*R*)-(*P*/*M*)-**7**.

Entry	Internal code	TFB [mM]	Solvent mixture (ratio)	AcOH (6M) [equiv]	Appearance	Yield [%] comments	Crystallinity [2θ]	S_{BET} [$m^2 g^{-1}$]
1	MJO-048	81	1,4-dioxane/mesitylene (1:1)	10	Red powder	50	Amorphous	767
2	MJO-050	81	1,4-dioxane/mesitylene (1:1)	10 TFA (6 M)	Red powder	55	Amorphous	623
3	MJO-054	71	1,4-dioxane/mesitylene (1:1)	20	Red powder	50	Amorphous	652
4	MJO-056	71	1,4-dioxane/mesitylene (1:1)	20 TFA (6 M)	Red powder	42	Amorphous	292
5	MJO-058	88	1,4-dioxane/mesitylene (1:1)	5	Red flakes	65	Amorphous	586
6	MJO-071	81	1,4-dioxane/mesitylene (1:1)	6 AcOH (10 M)	Red powder	70	Amorphous	639
7	MJO-072	71	o-DCB/n-BuOH (1:1)	10 AcOH (10 M)	Red powder	76	Amorphous	772
8	MJO-086	71	o-DCB/n-BuOH (7:3)	10 AcOH (10 M)	Red powder	50	Amorphous	360

* The tube was sealed off and heated at 120 °C for 72 h.

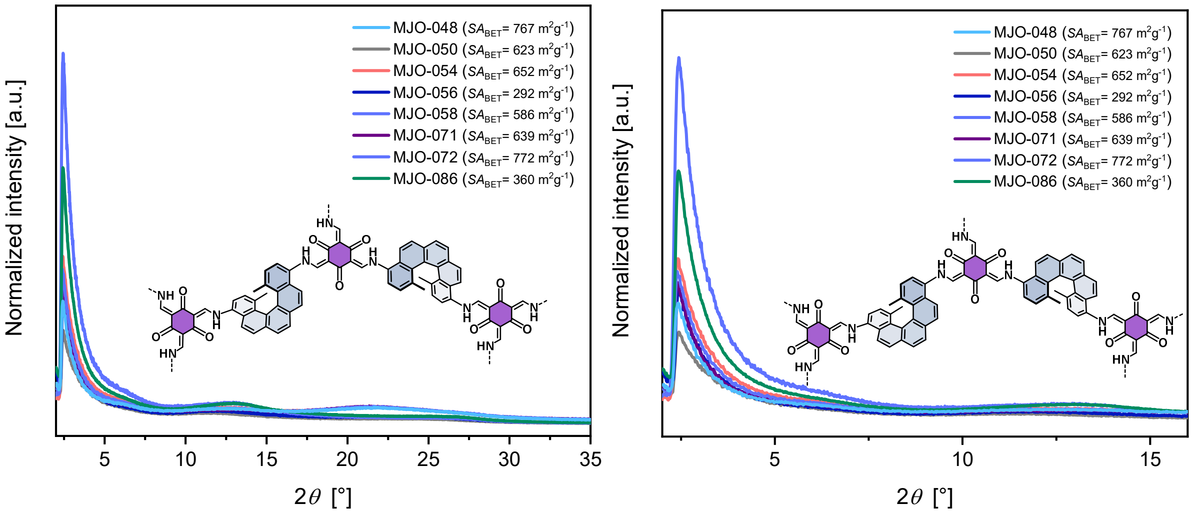

Figure S41. PXRD patterns of screening experimental conditions for *rac*-**[5]Heli-TFP** POP from (*R*,*R*)-(*P*/*M*)-**7**. Data were collected using the Rigaku Miniflex benchtop instrument.

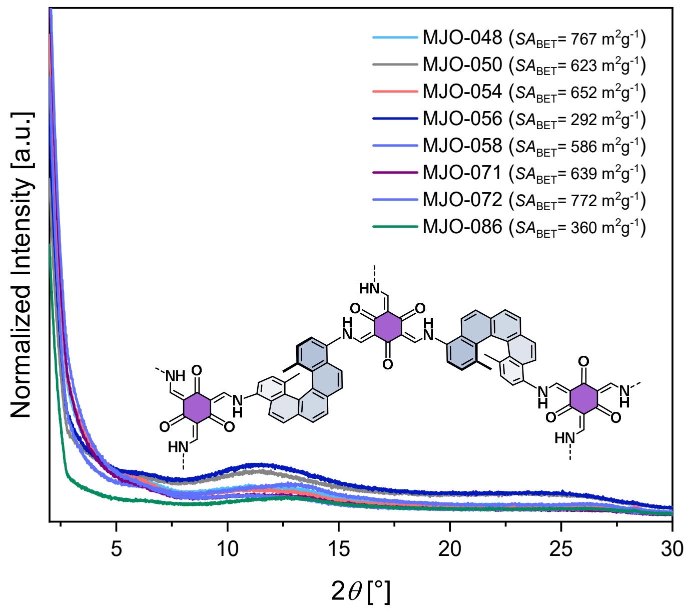

Figure S42. PXRD patterns of selected samples of *rac*-**[5]Heli-TFP** POP from (*R*,*R*)-(*P*/*M*)-**7**; data were collected using the SAXS/WAXS instrument.

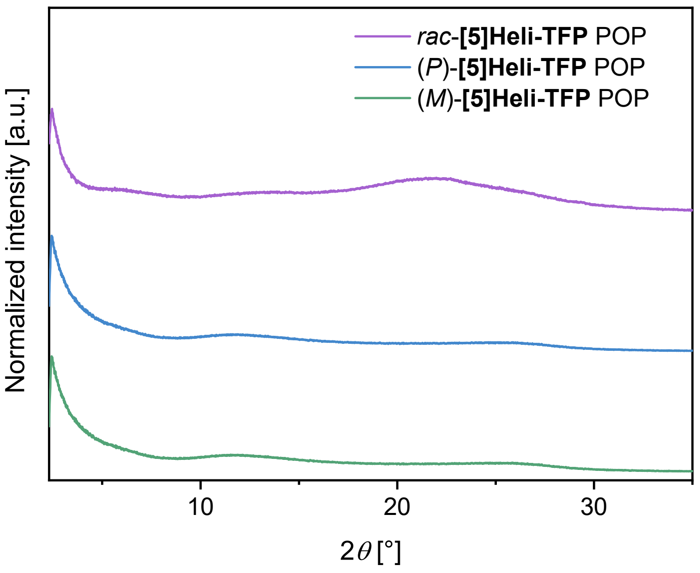

Figure S43. Experimental PXRD patterns of *rac*-**[5]Heli-TFP** POP (purple line), (*P*)-**[5]Heli-TFP** POP (blue line), (*M*)-**[5]Heli-TFP** POP (green line). The resulting materials from the building blocks: (*R*,*R*)-(*P*/*M*)-**7**, (*R*,*R*)-(*P*)-(+)-**7**, and (*R*,*R*)-(*M*)-(–)-**7** showing an amorphous behavior, data were collected using the Rigaku Miniflex instrument.

These results confirm the steric limitations of [5]helicene derivatives without a phenyl-spacer: (±)-**6**, (*R*,*R*)-(±)-**7**, *R*,*R*)-(*M*)-(–)-**7** and (*R*,*R*)-(*P*)-(+)-**7** as a *pseudo*-C_2 linkers, with TFP for the target formation of **[5]Heli-TFP** COF-2 (Scheme S3). PXRD analysis of the resulting materials revealed non-crystalline morphologies, identified by smooth and shallow diffraction patterns. Since they exhibit porosity in N_2 sorption measurement (Figures S51-54), these materials are considered as porous organic polymer (POPs). These results highlight the essential role of the phenyl-spacer in the (4,11)-positions in the central annulated core, along with β-ketoenamine linkages to obtain homochiral and racemic [5]helicene based lattices.

S5. Nitrogen adsorption isotherms

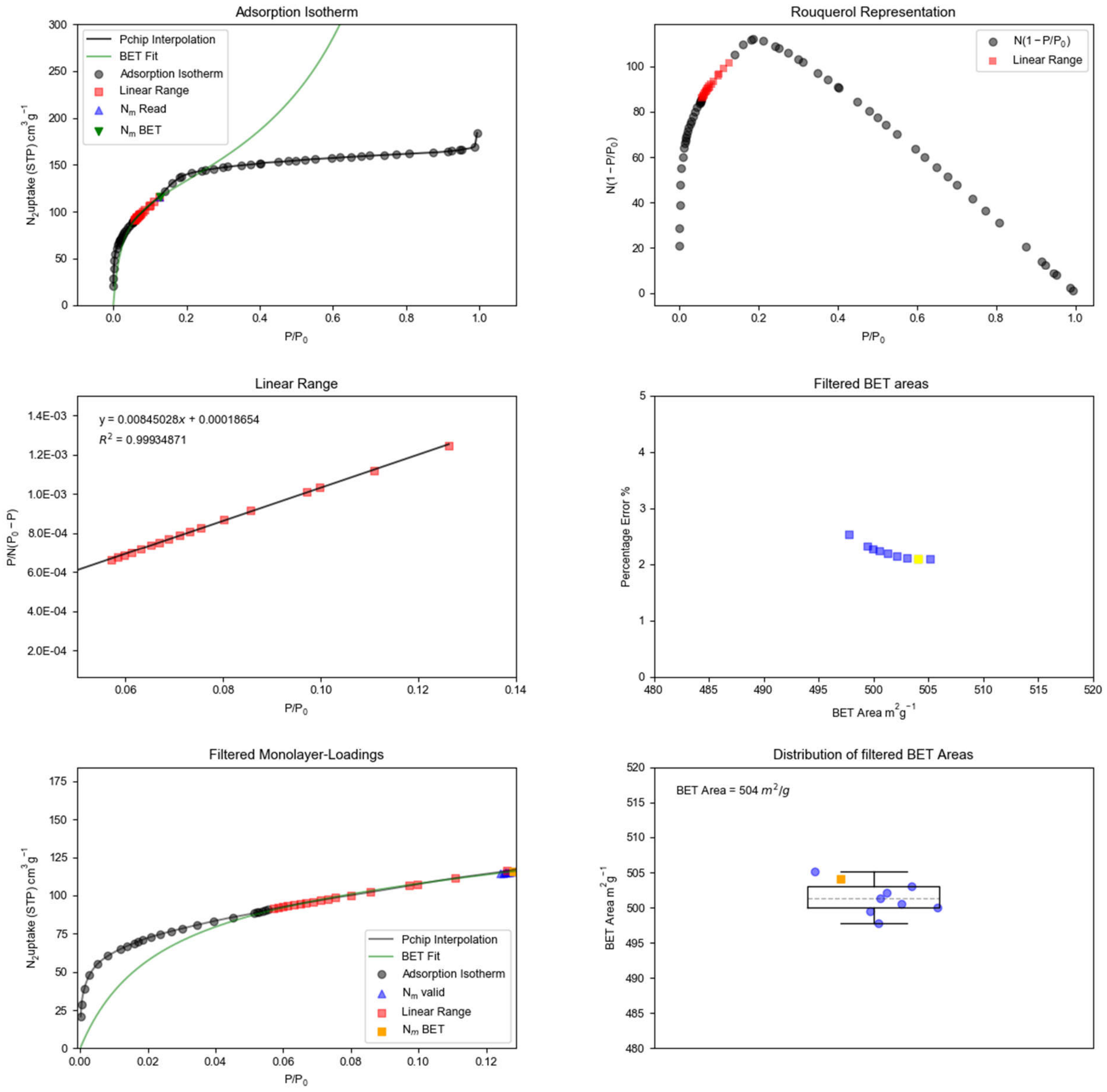

Figure S44. BET analysis of *rac*-**[5]Heli-TFB** COF using the BETSI program.[12]

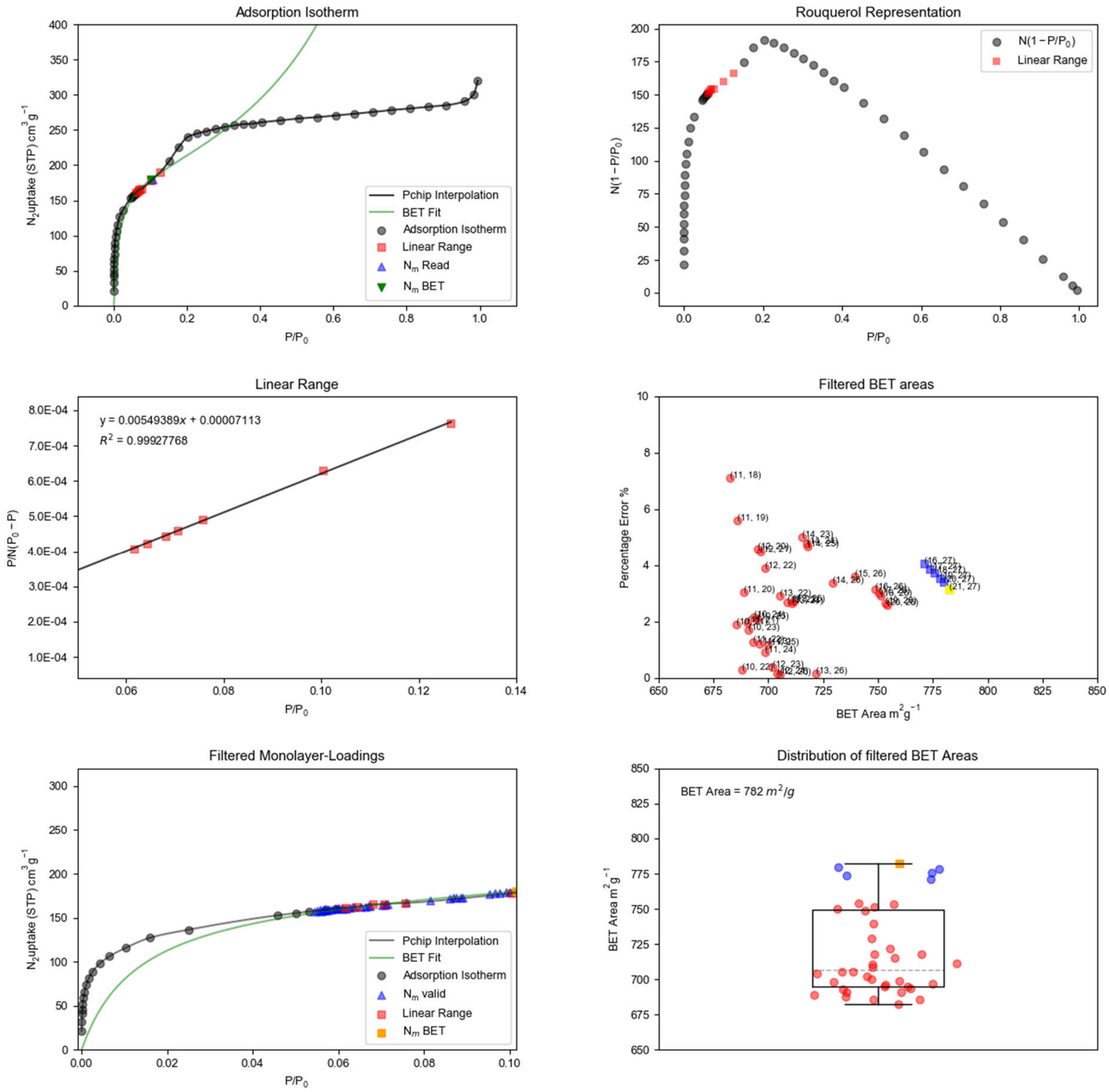

Figure S45. BET analysis of *rac*-**[5]Heli-TFP** COF using the BETSI program.[12]

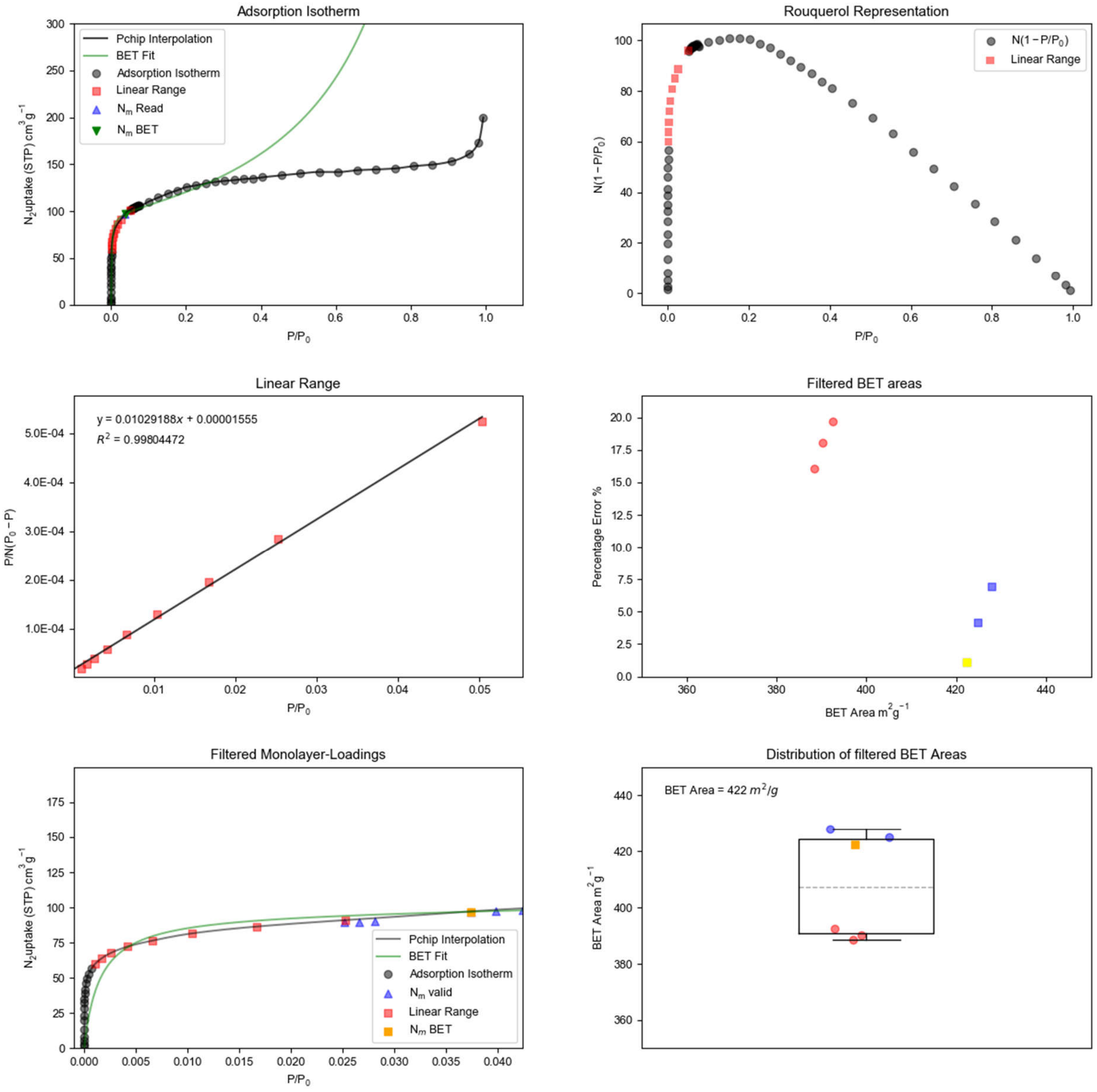

Figure S46. BET analysis of (*P*)-**[5]Heli-TFP** COF using the BETSI program.[12]

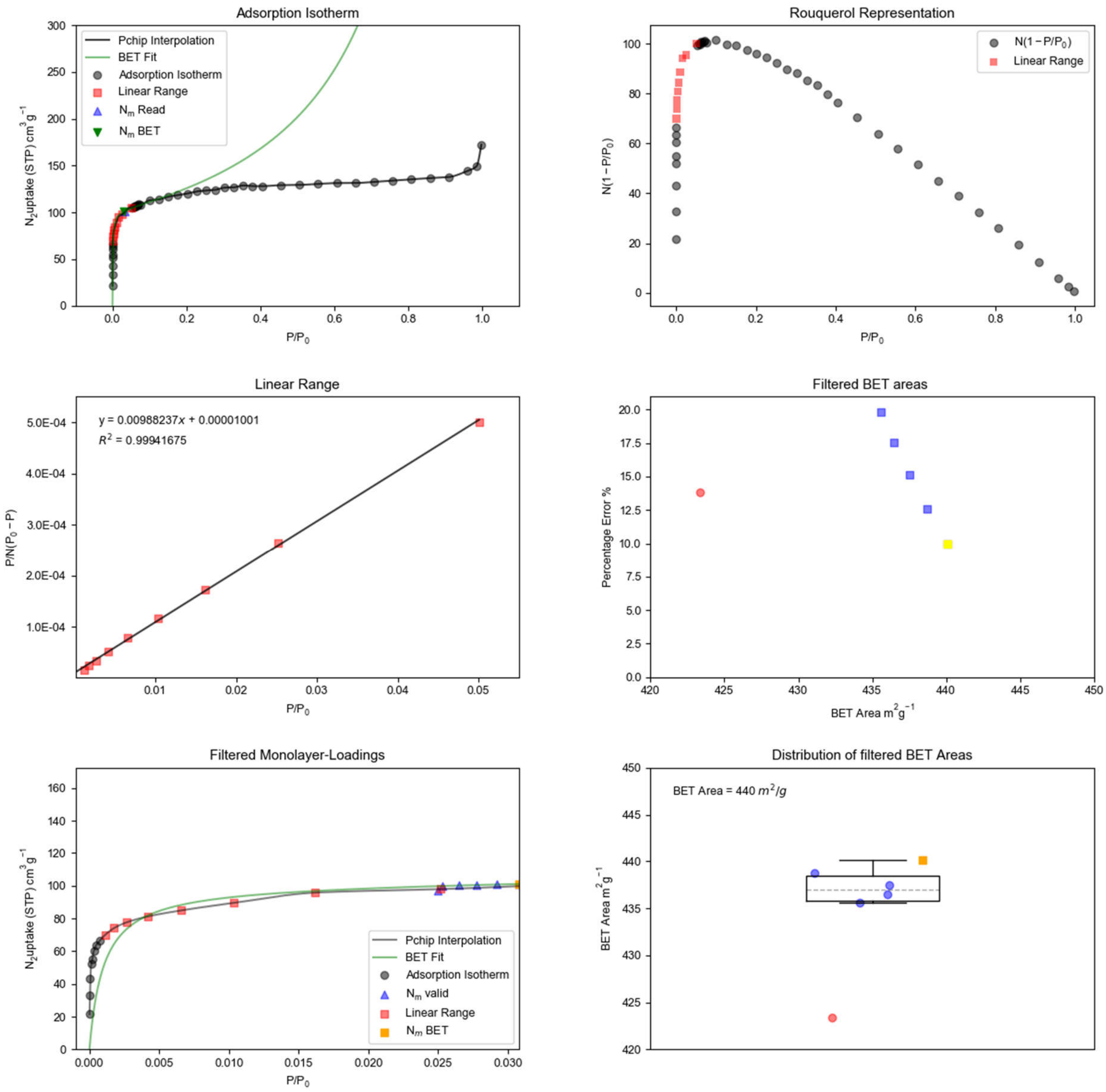

Figure S47. BET analysis of (*M*)-**[5]Heli-TFP** COF using the BETSI program.[12]

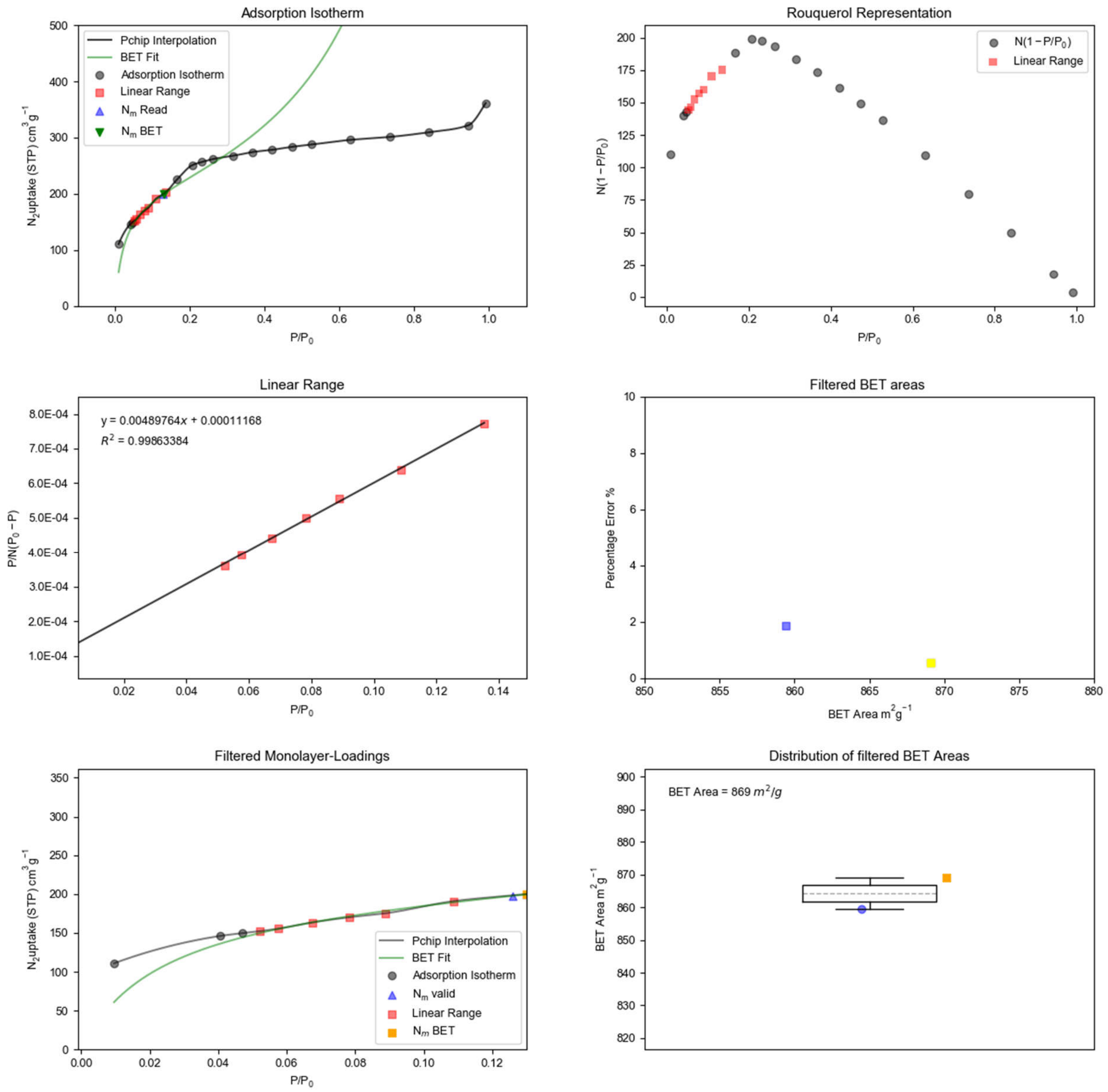

Figure S48. BET analysis of *rac*-**[5]Heli-TFP** COF film with a concentration of 12 mM of (±)-**3** using the BETSI program.[12]

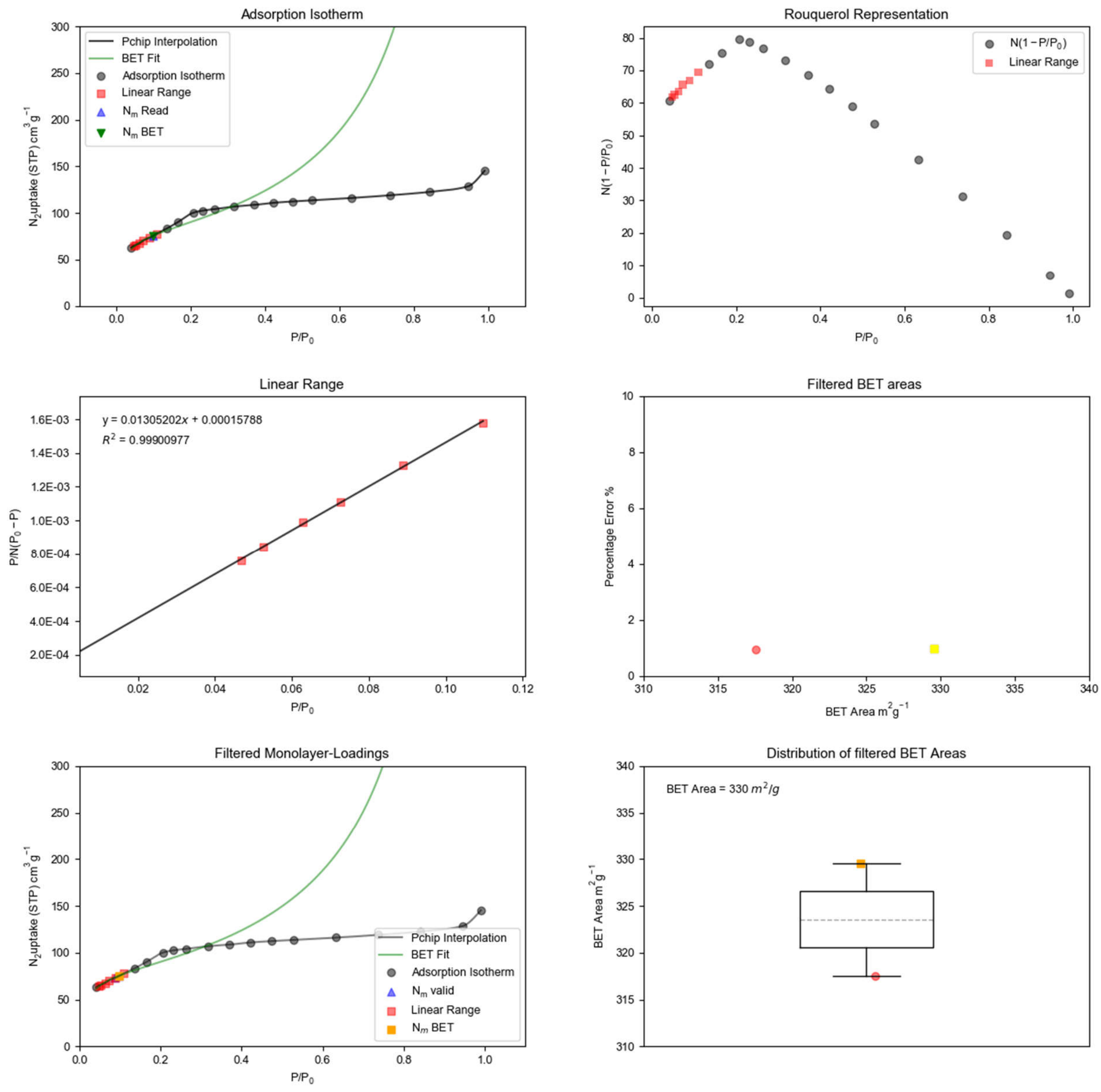

Figure S49. BET analysis of (*P*)-**[5]Heli-TFP** COF film with concentration of 12 mM of (*P*)-(–)-**3** using the BETSI program.[12]

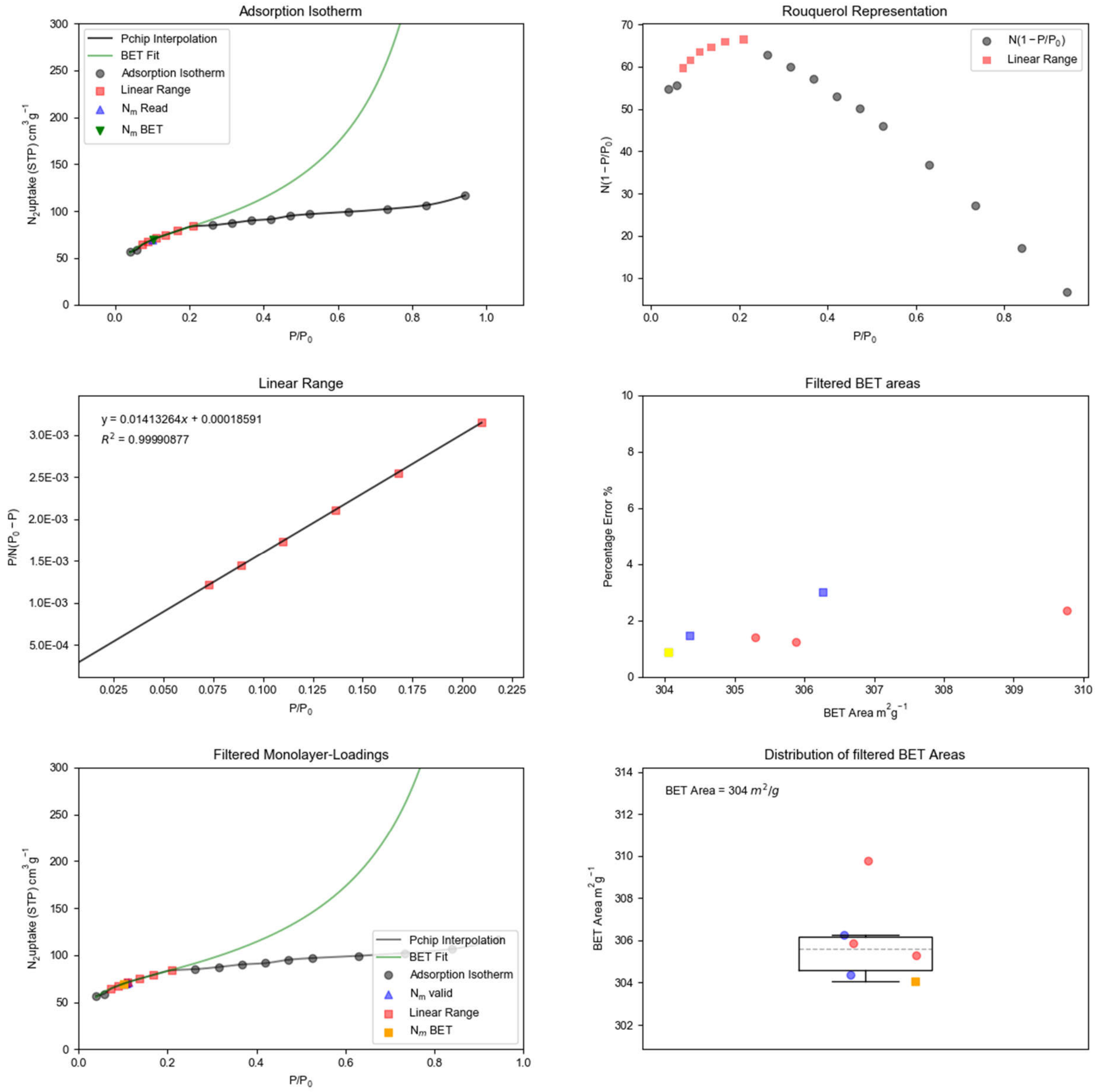

Figure S50. BET analysis of (*M*)-**[5]Heli-TFP** COF film with concentration of 12 mM of (*M*)-(+)-**3** using the BETSI program.[12]

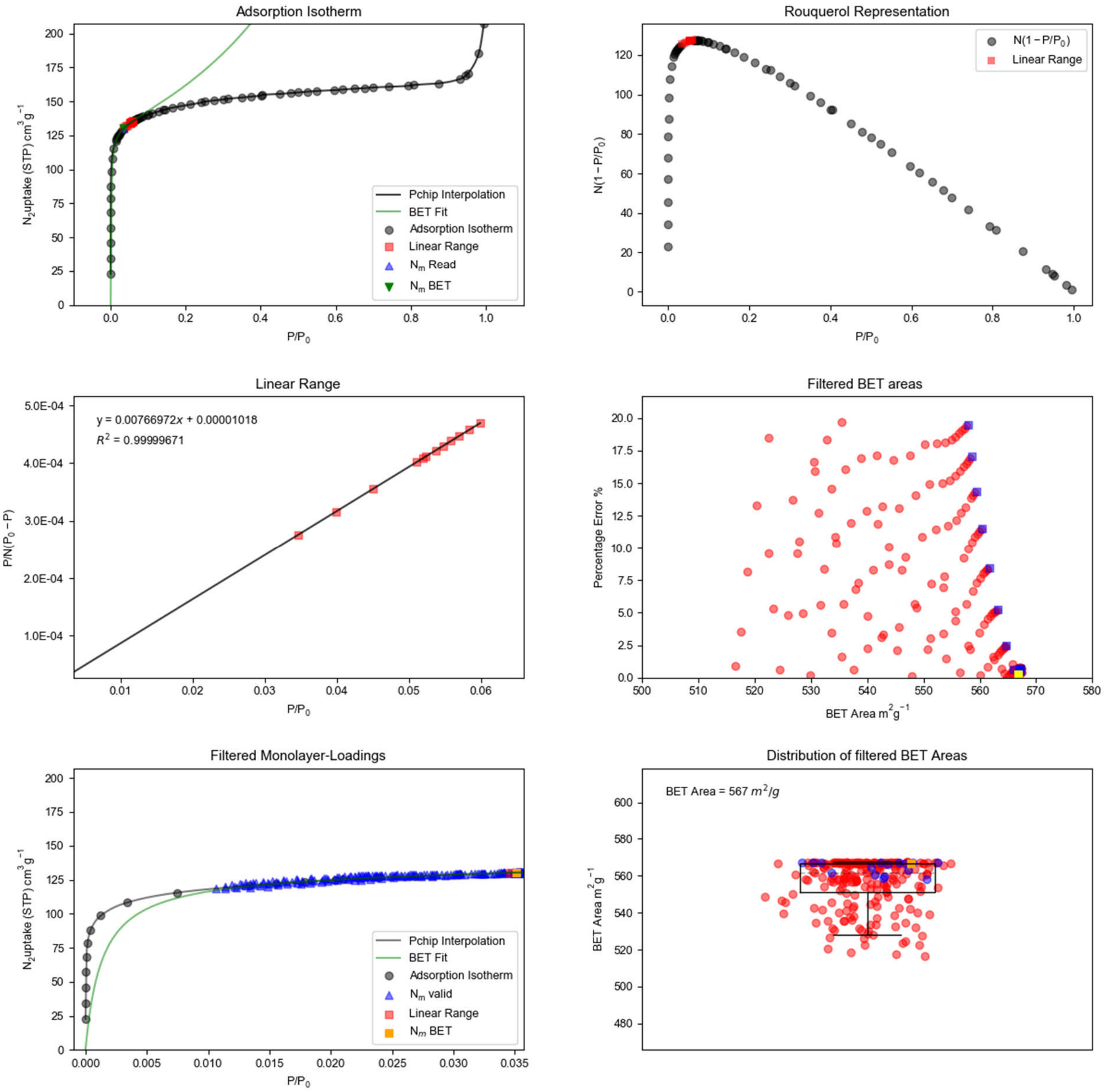

Figure S51. BET analysis of *rac*-**[5]Heli-TFP** POP from (±)-**6** using the BETSI program.[12]

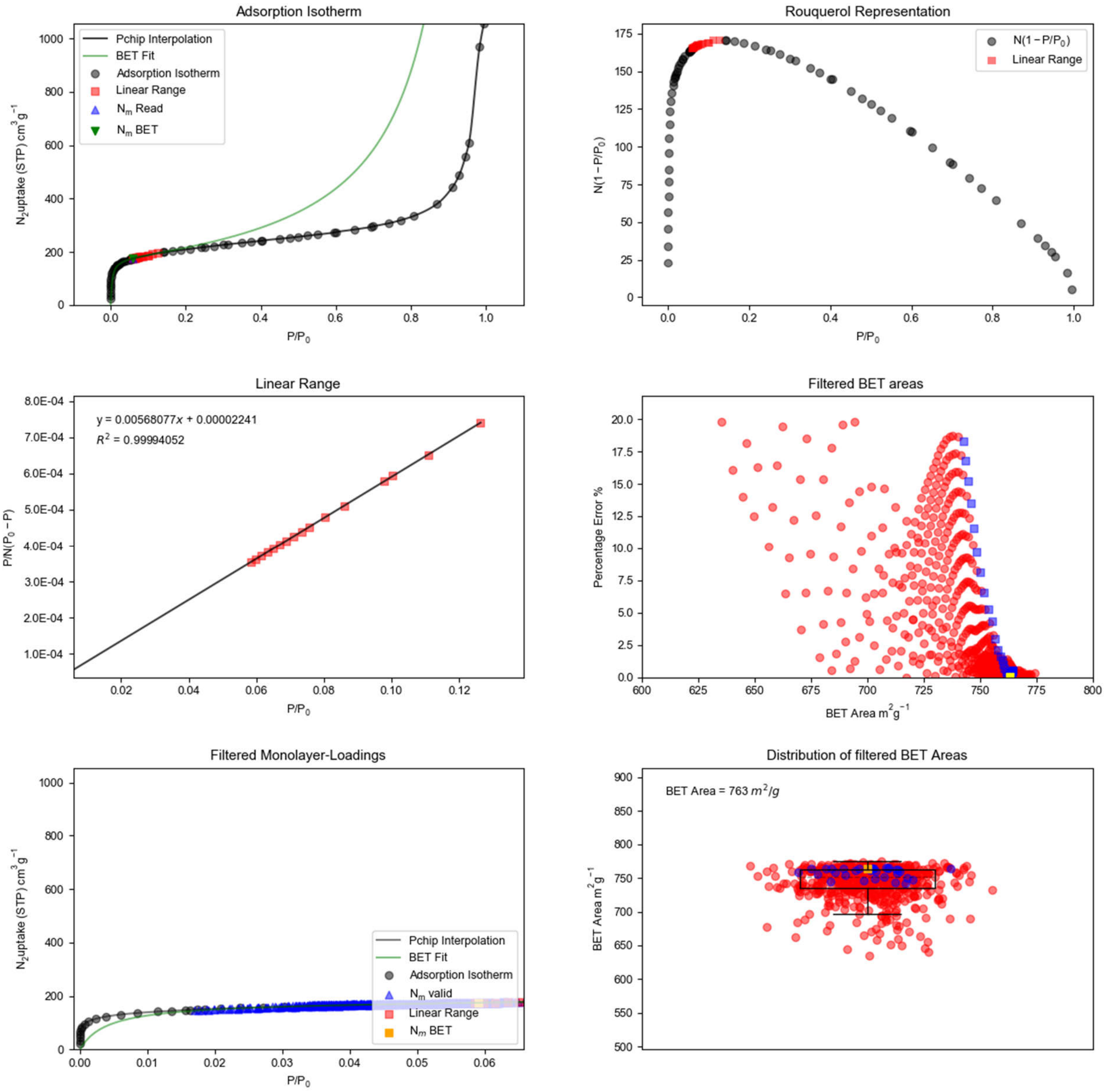

Figure S52. BET analysis of *rac*-**[5]Heli-TFP** POP from (±)-**7** using the BETSI program.[12]

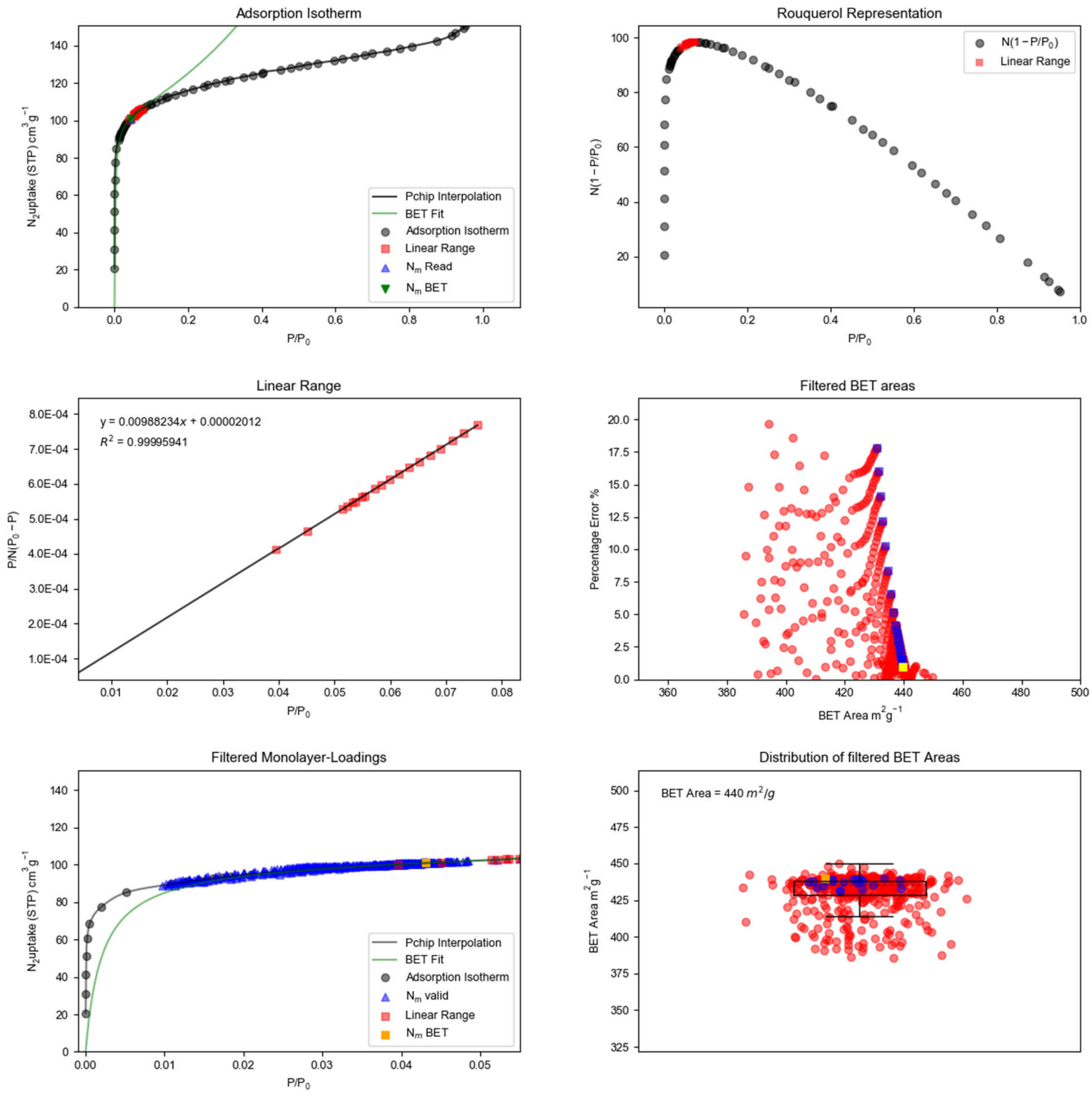

Figure S53. BET analysis of (*M*)-**[5]Heli-TFP** POP from (*R*,*R*)-(*M*)-(–)-**7** using the BETSI program.[12]

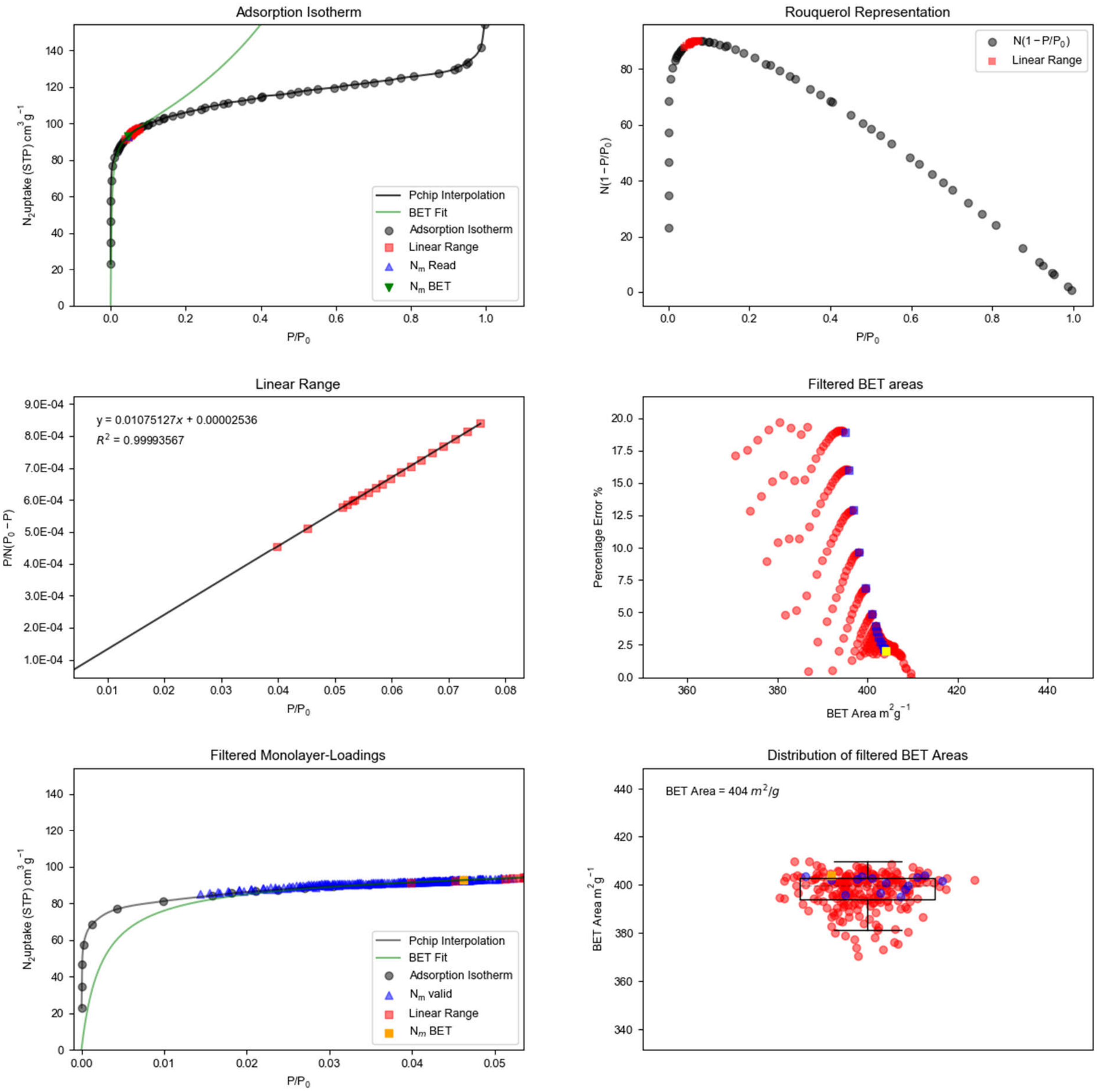

Figure S54. BET analysis of (*P*)-**[5]Heli-TFP** POP from (*R*,*R*)-(*P*)-(+)-**7** using the BETSI program.[12]

S6. Pore Size Distribution

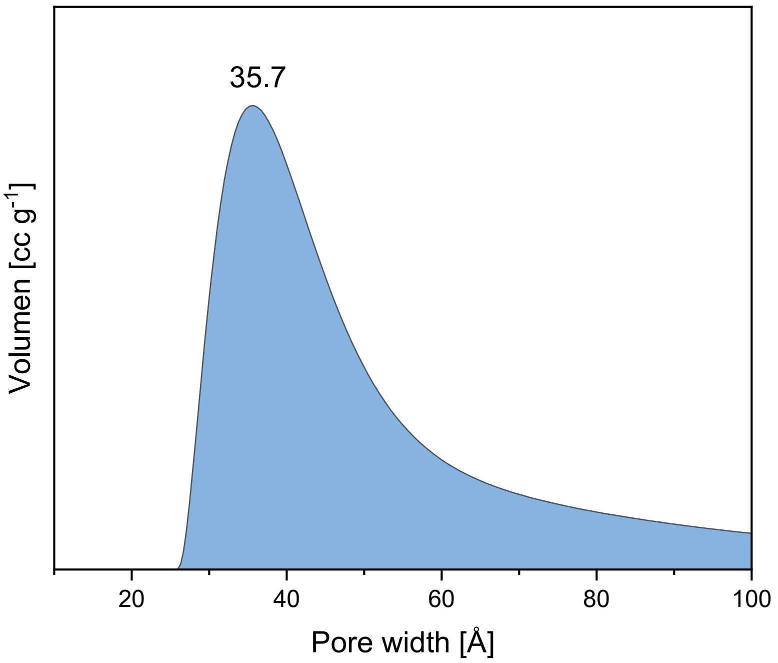

Figure S55. Pore size distribution of *rac*-**[5]Heli-TFB** COF, Carbon N_2, 77 K, cylindrical model.

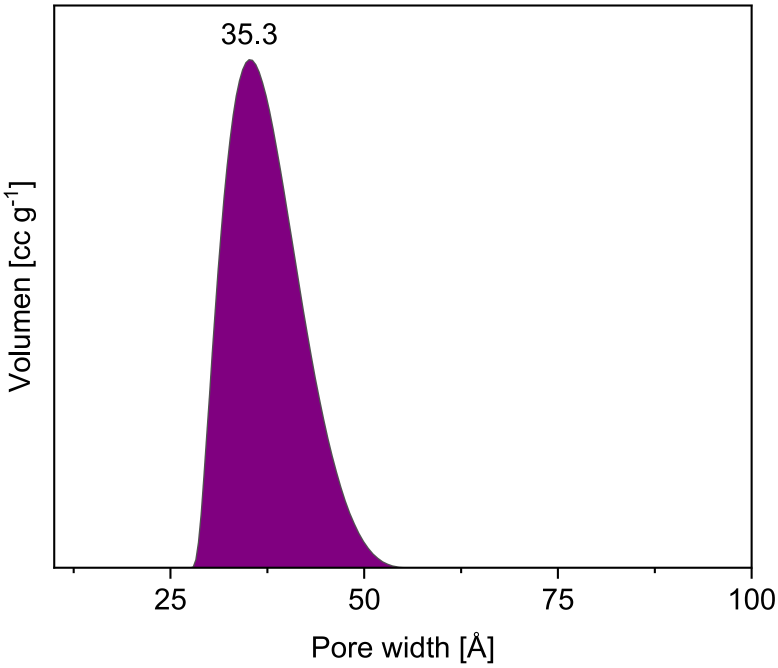

Figure S56. Pore size distribution of *rac*-**[5]Heli-TFP** COF, Carbon N_2, 77 K, cylindrical model.

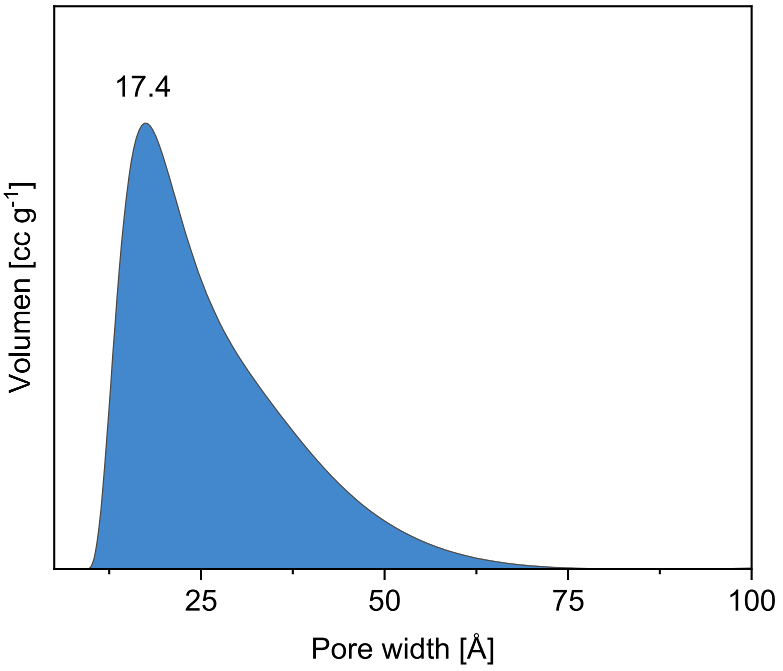

Figure S57. Pore size distribution of (*P*)-**[5]Heli-TFP** COF, Carbon N_2, 77 K, cylindrical model.

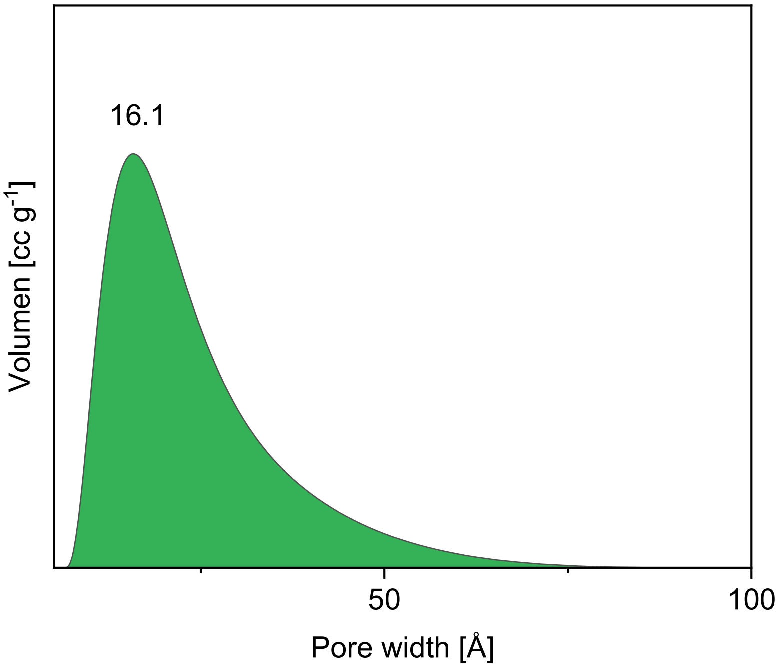

Figure S58. Pore size distribution of (*M*)-**[5]Heli-TFP** COF, Carbon N_2, 77 K, cylindrical model.

S7. High-Resolution Transmission Electron Microscopy

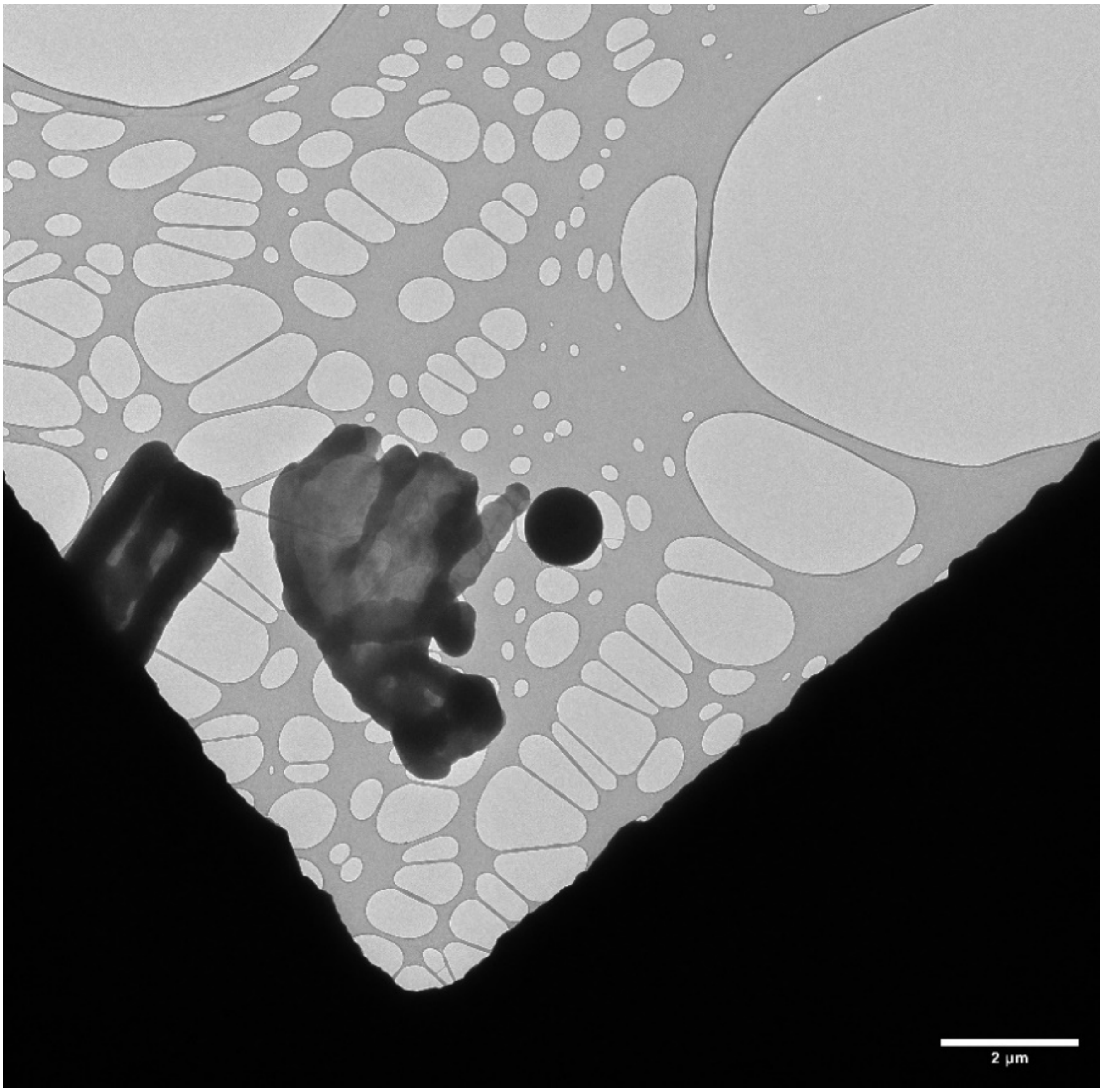

Figure S59. HR-TEM image of *rac*-**[5]Heli-TFB** COF with 3k magnification, accelerating voltage 200 kV at 77 K.

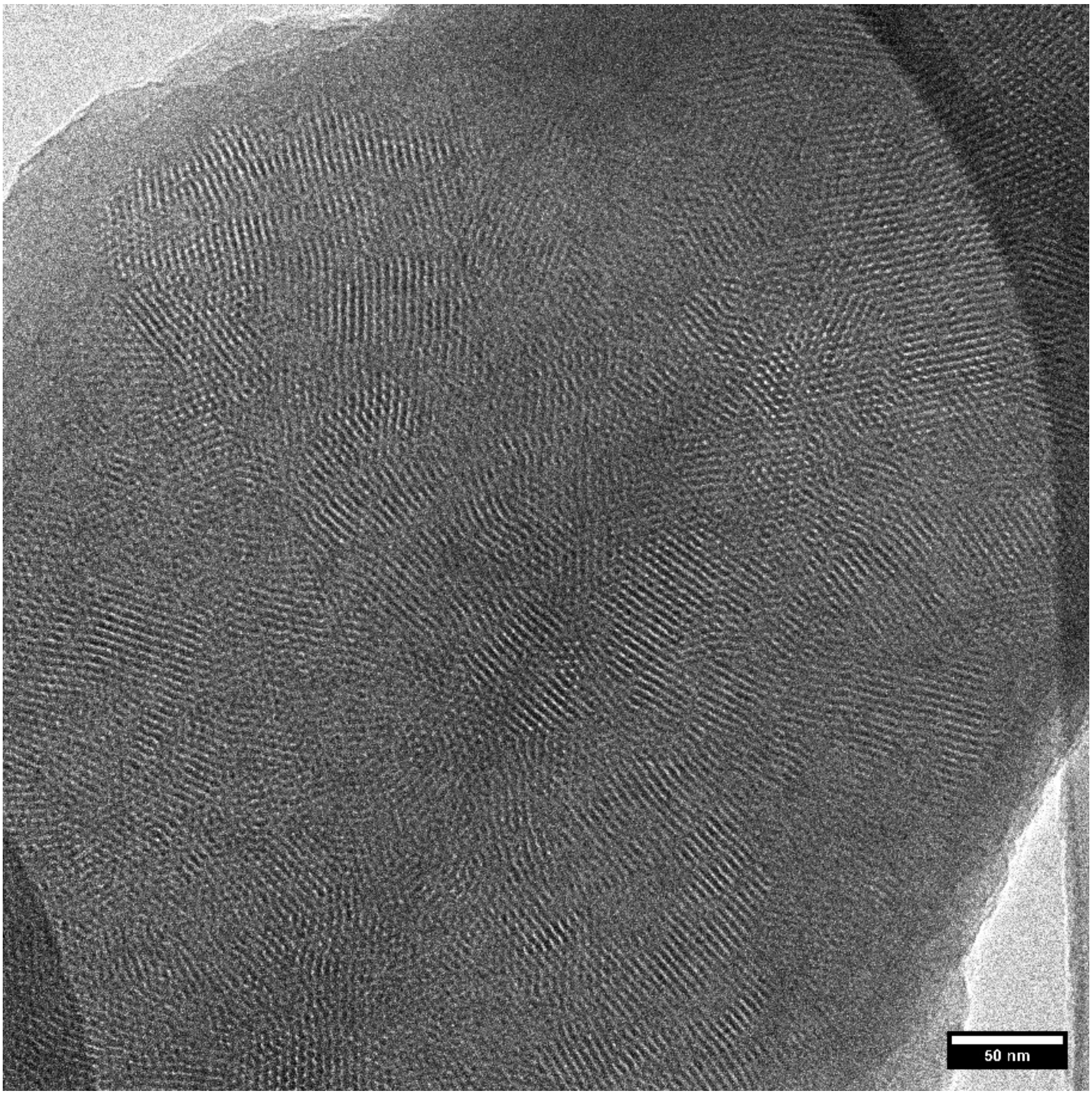

Figure S60. HR-TEM image of *rac*-**[5]Heli-TFB** COF with 100k magnification, accelerating voltage 200 kV at 77 K.

Table S6. Estimated *d*-spacing of *rac*-**[5]Heli-TFB** COF from the HR-TEM figure S60. A distance was measured along continuous hexagonal ring patterns (6, 8 and 10) using the software Fiji, and divided by the number of rings to obtain an average *d*-spacing value in the dimension of "one ring pattern".[14]

Measurement	Distance along 6 rings [nm]	Distance along 8 rings [nm]	Distance along 10 rings [nm]
1	25.15	32.17	39.72
2	24.70	33.26	39.68
3	24.78	32.31	41.09
4	24.80	31.03	40.69
5	24.53	32.31	41.58
6	23.53	33.55	41.07
7	25.26	34.60	41.06
8	23.70	32.78	41.13
9	23.20	32.17	40.91
10	25.39	32.43	40.12
average	24.50	32.17	40.70
d-spacing	4.08	4.08	4.07
Average *d*-spacing from all the measurements (nm)			**4.08**

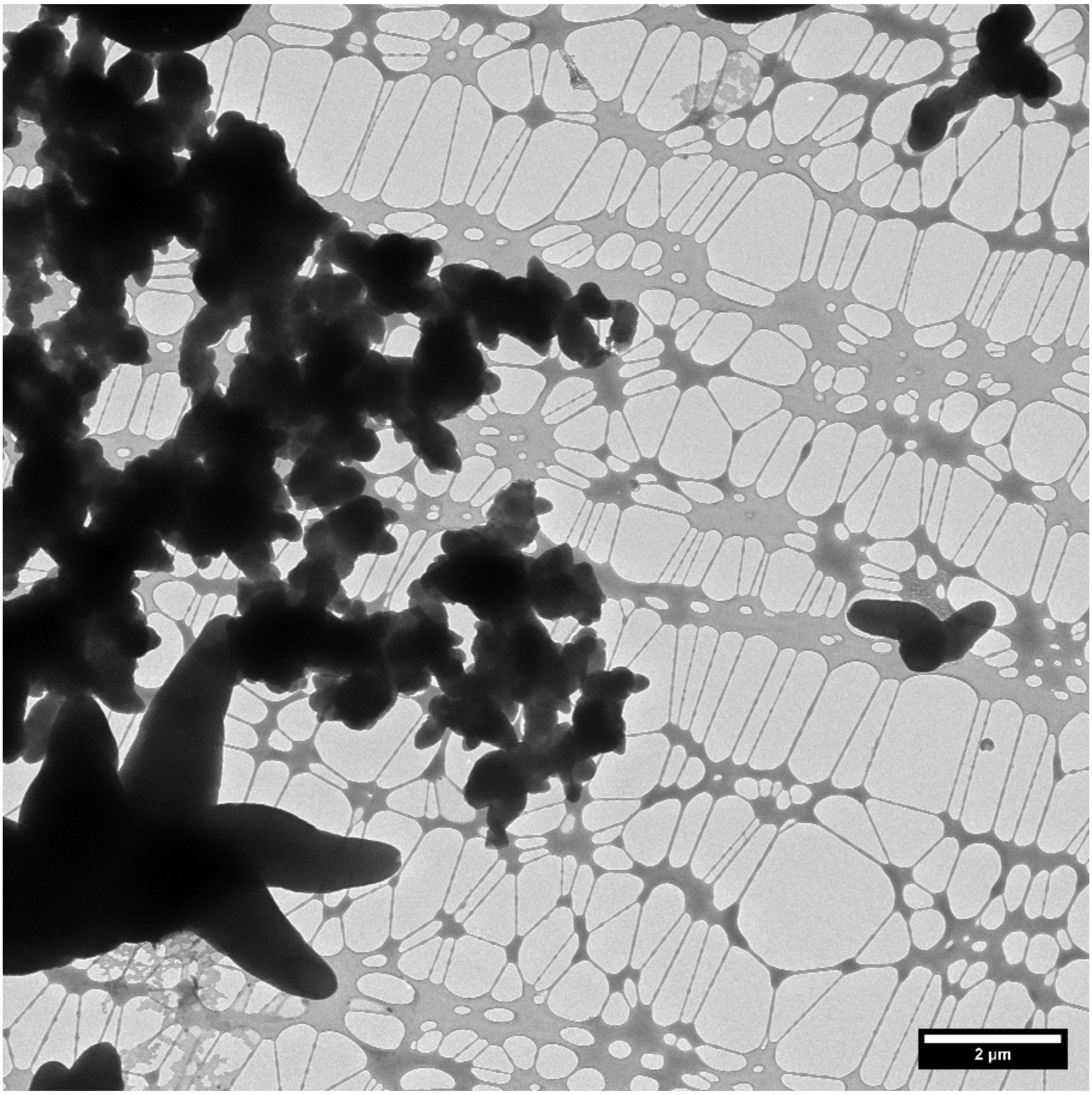

Figure S61. HR-TEM image of *rac*-**[5]Heli-TFP** COF with 3k magnification, accelerating voltage 200 kV at 77 K.

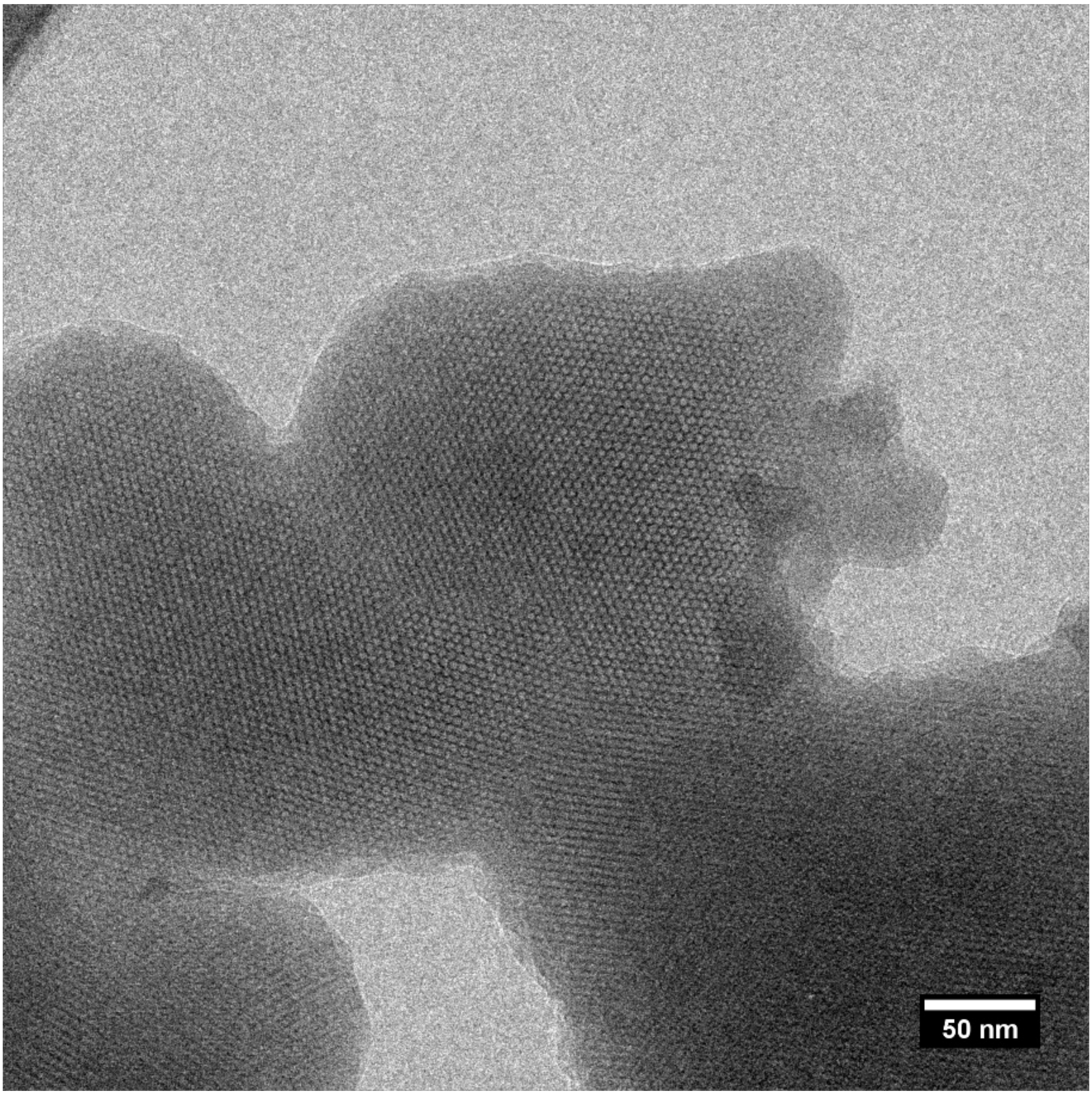

Figure S62. HR-TEM image of *rac*-**[5]Heli-TFP** COF with 100k magnification, accelerating voltage 200 kV at 77 K.

Table S7. Estimated *d*-spacing of *rac*-**[5]Heli-TFP** COF from the HR-TEM figure S62. A distance was measured along continuous hexagonal ring patterns (6, 8 and 10) using the software Fiji and finally divided by the number of rings to obtain an average d-spacing value in the dimension of "one ring pattern".[14]

Measurement	Distance along 6 rings [nm]	Distance along 8 rings [nm]	Distance along 10 rings [nm]
1	24.45	32.61	41.13
2	24.44	32.52	41.19
3	24.46	32.89	41.09
4	24.26	32.82	40.69
5	24.74	33.24	41.03
6	24.17	31.23	41.07
7	24.22	32.28	41.06
8	24.65	33.23	40.69
9	24.54	33.07	41.03
10	24.69	32.96	41.25
average	24.46	32.68	41.02
d-spacing	4.08	4.09	4.10
Average *d*-spacing from all the measurements (nm)			4.09

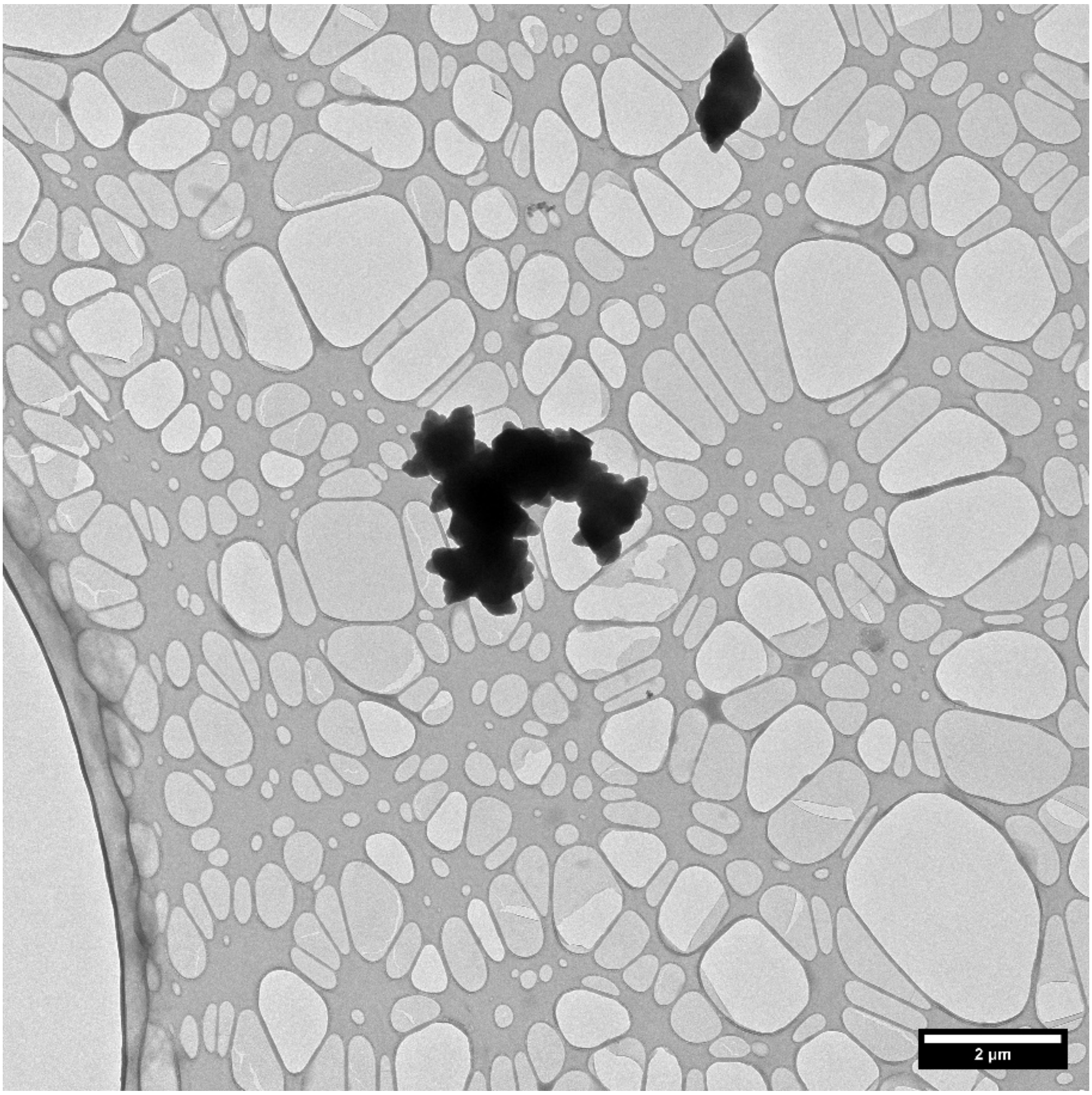

Figure S63. HR-TEM image of (*P*)-**[5]Heli-TFP** COF with 3k magnification, accelerating voltage 200 kV at 77 K.

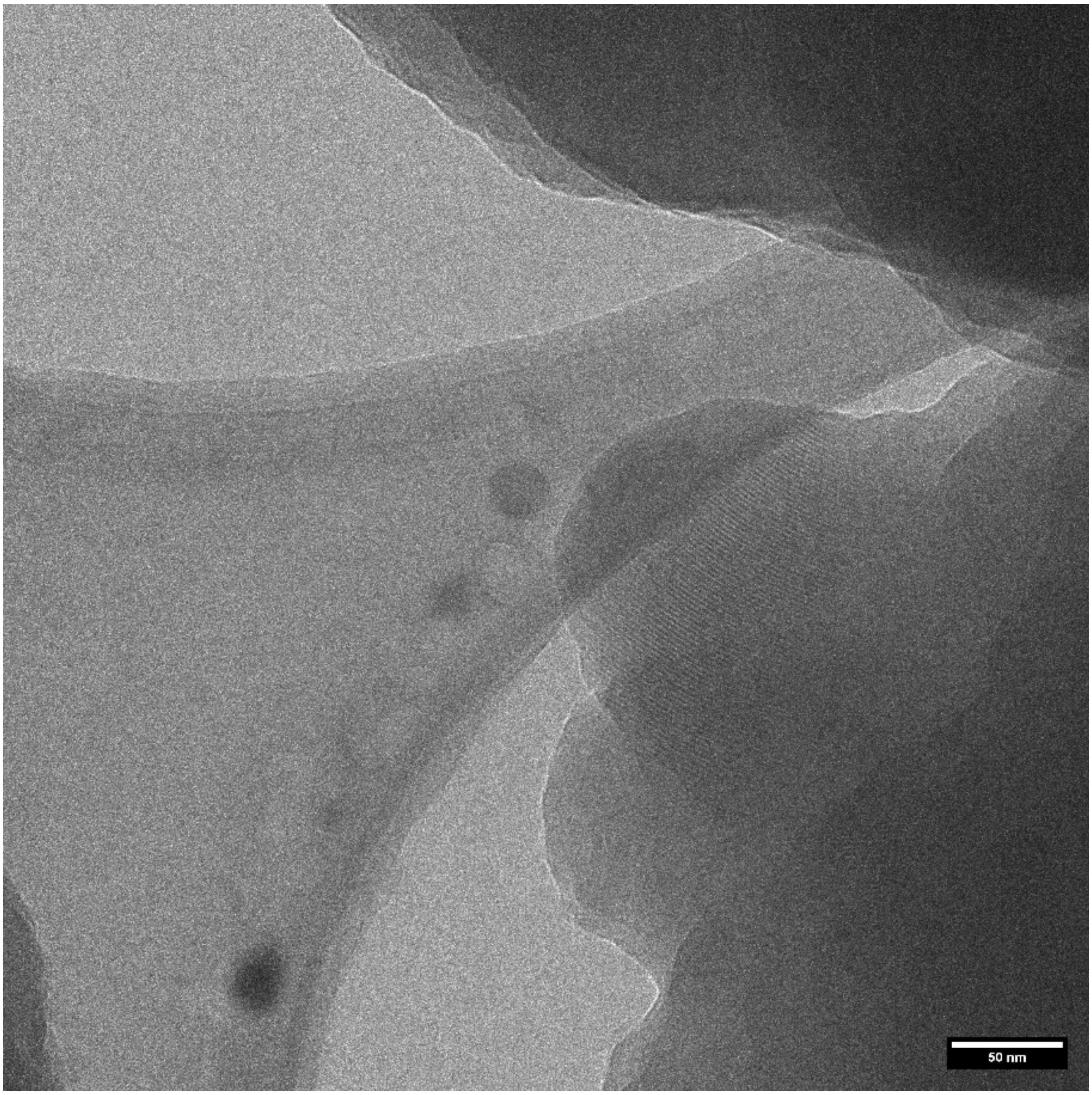

Figure S64. HR-TEM image of (*P*)-**[5]Heli-TFP** COF with 100k magnification, accelerating voltage 200 kV at 77 K.

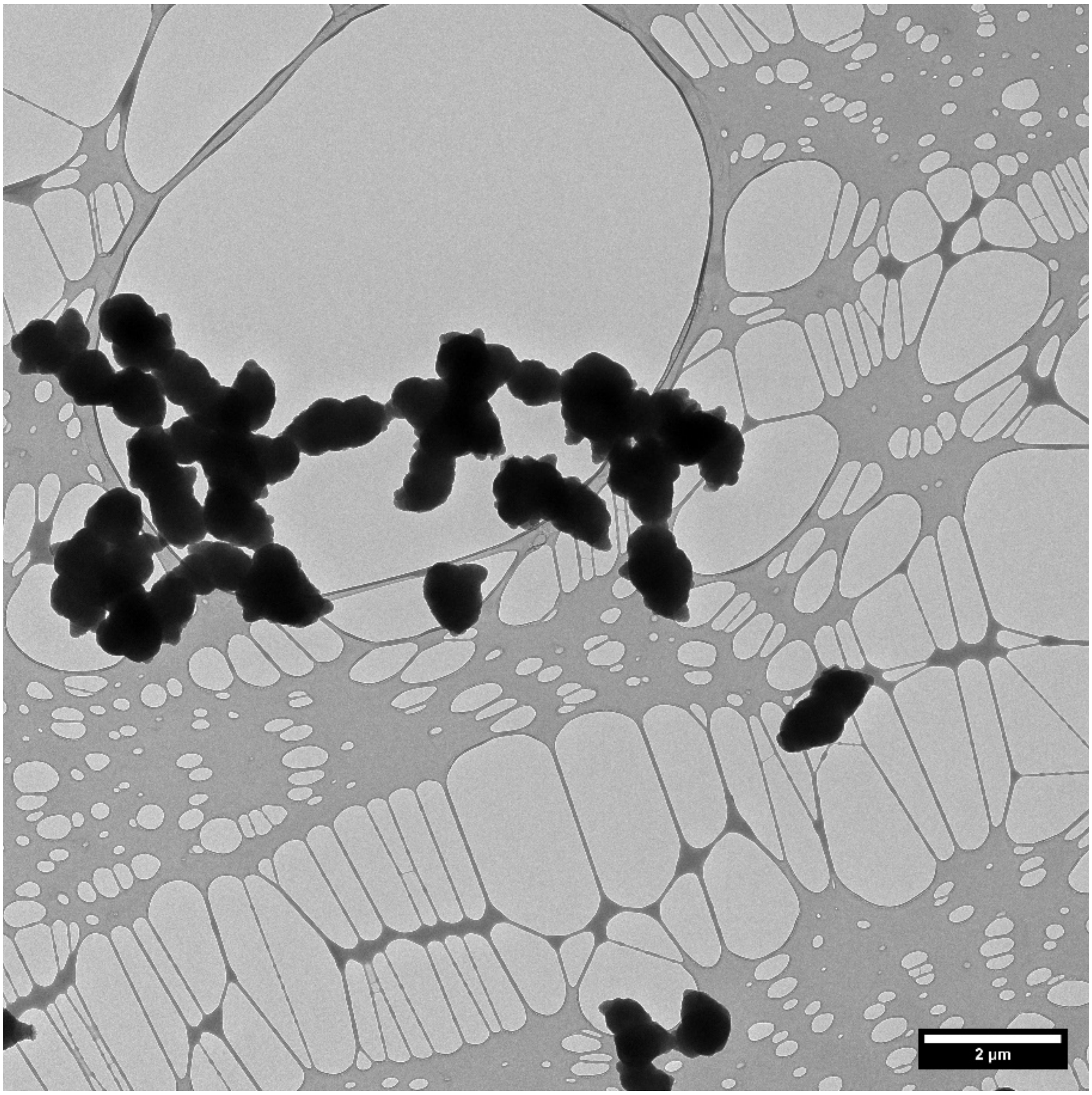

Figure S65. HR-TEM image of (*M*)-**[5]Heli-TFP** COF with 3k magnification, accelerating voltage 200 kV at 77 K.

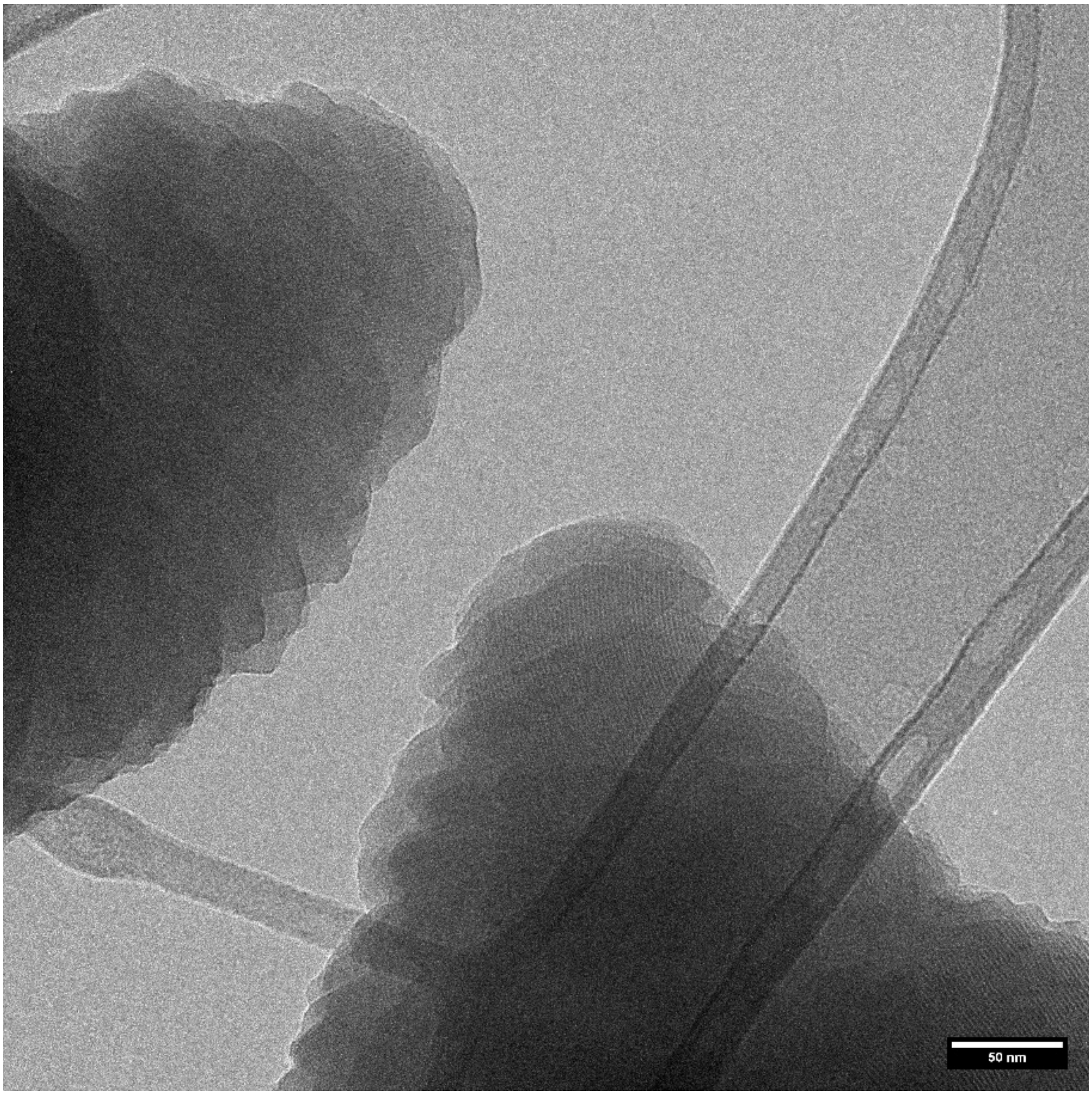

Figure S66. HR-TEM image of (*M*)-**[5]Heli-TFP** COF with 100k magnification, accelerating voltage 200 kV at 77 K.

Table S8. Measurement of the distance of lattice fringes from (*P*)-**[5]Heli-TFP** COF using HR-TEM figure S64 by using the software Fiji.[14]

Measurement	Distance of lattice fringes [nm]
1	2.43
2	2.28
3	2.49
4	2.40
5	2.36
6	2.48
7	2.36
8	2.44
9	2.32
10	2.41
average	2.40

Table S9. Measurement of the distance of lattice fringes from (*M*)-**[5]Heli-TFP** COF using HR-TEM figure S66 by using the software Fiji.[14]

Measurement	Distance of lattice fringes [nm]
1	2.45
2	2.37
3	2.40
4	2.37
5	2.36
6	2.48
7	2.40
8	2.43
9	2.35
10	2.40
average	2.40

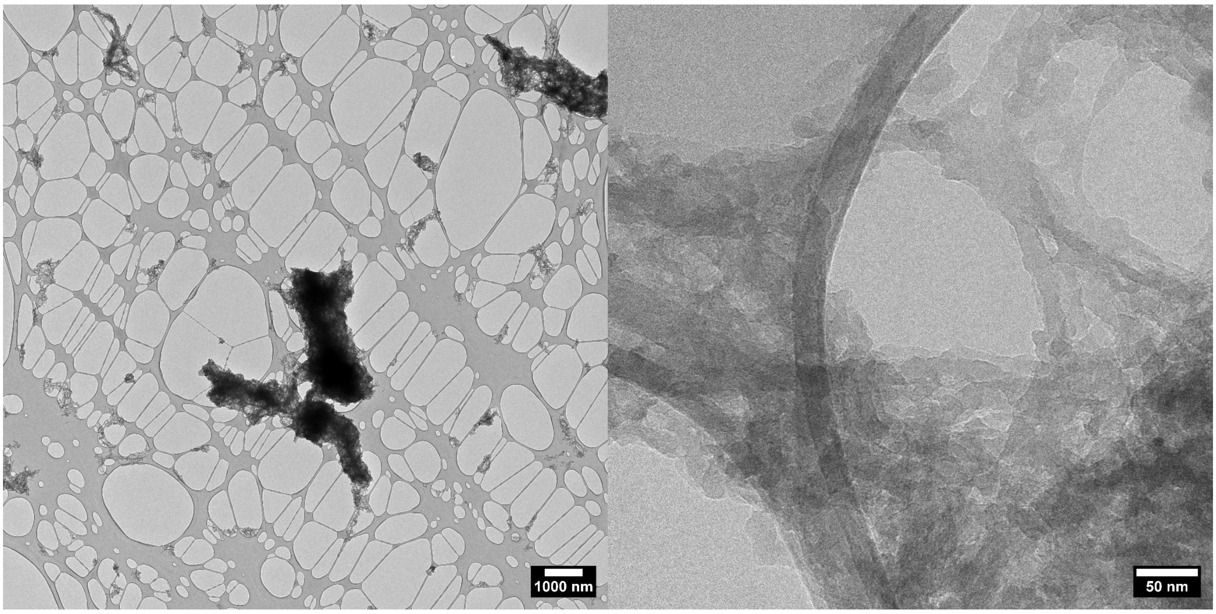

Figure S67. HR-TEM image of *rac*-**[5]Heli-TFP** POP from (*R*,*R*)-(*P*/*M*)-**7** at 77 K.

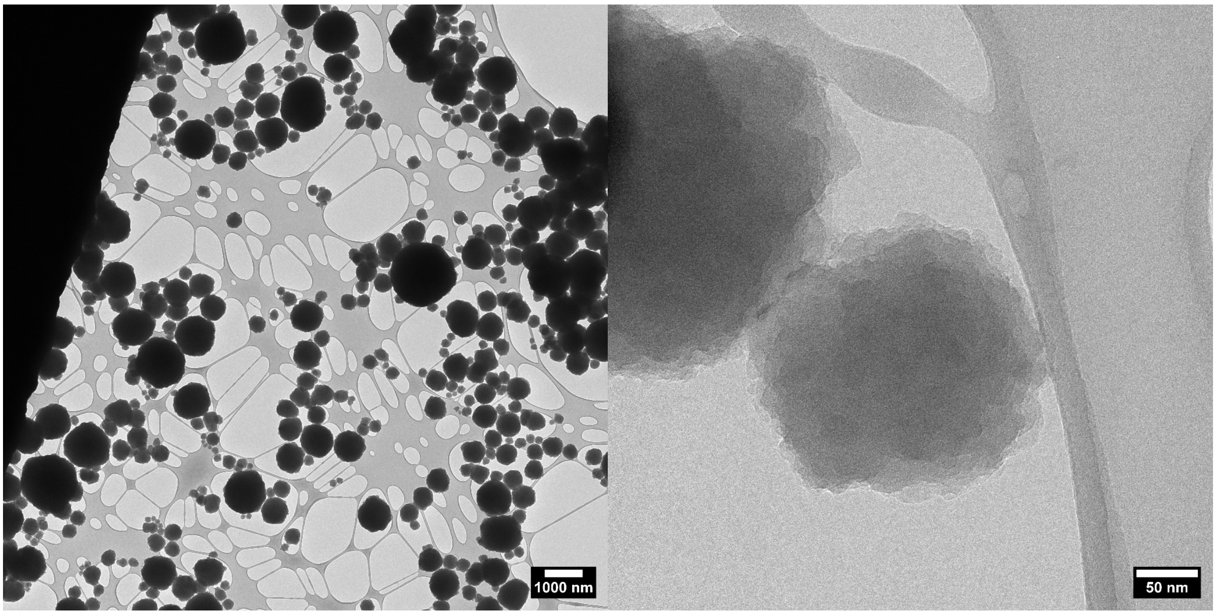

Figure S68. HR-TEM image of (*M*)-**[5]Heli-TFP** POP from (*R*,*R*)-(*M*)-(–)-**7** at 77 K.

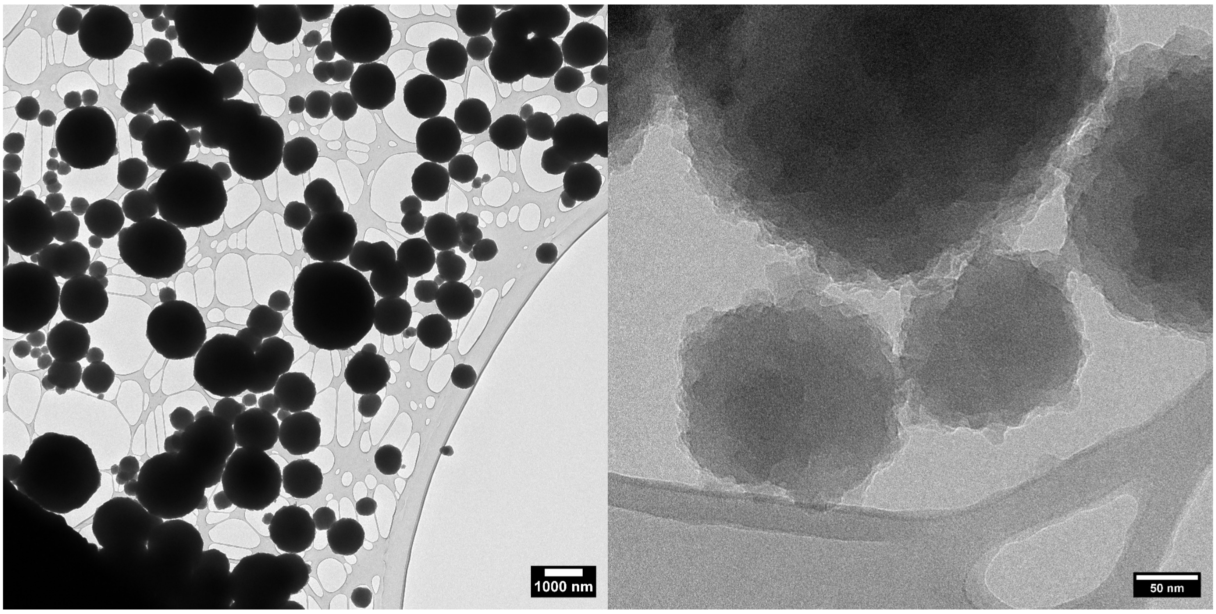

Figure S69. HR-TEM image of (*P*)-**[5]Heli-TFP** POP from (*R*,*R*)-(*P*)-(+)-**7** at 77 K.

S8. Scanning Electron Microscopy

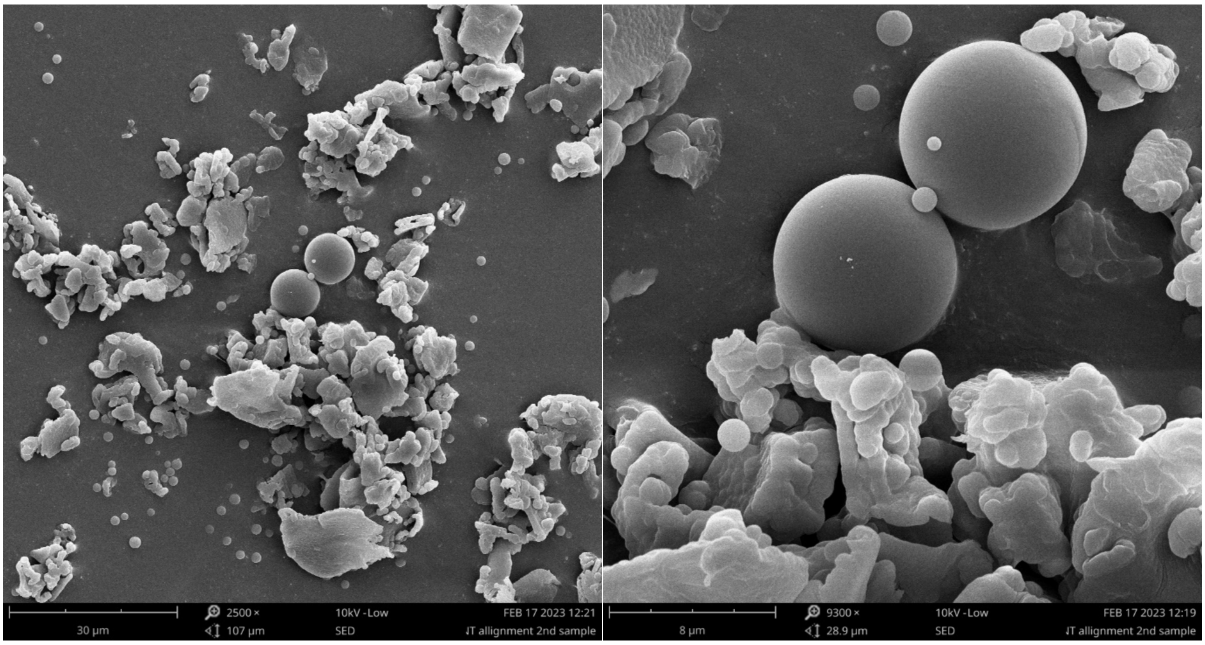

Figure S70. FE-SEM micrographs of *rac*-**[5]Heli-TFB** COF with 2.5k and 9.3k magnification at 25 °C.

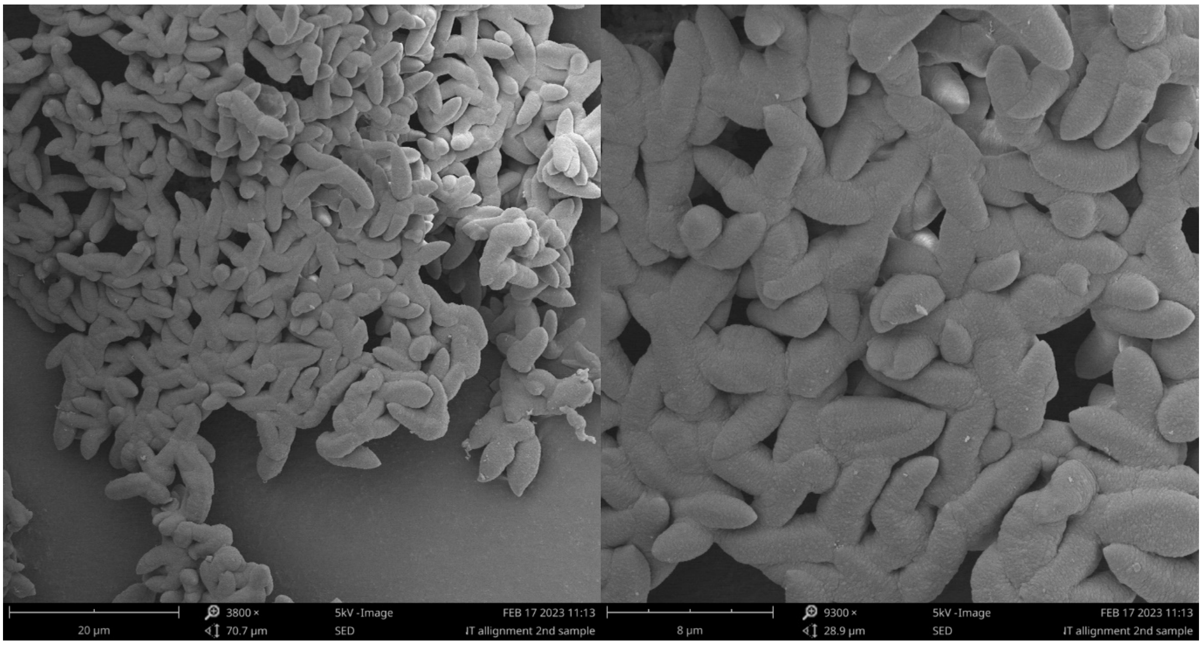

Figure S71. FE-SEM micrographs of *rac*-**[5]Heli-TFP** COF with 2.5k and 9.3k magnification at 25 °C.

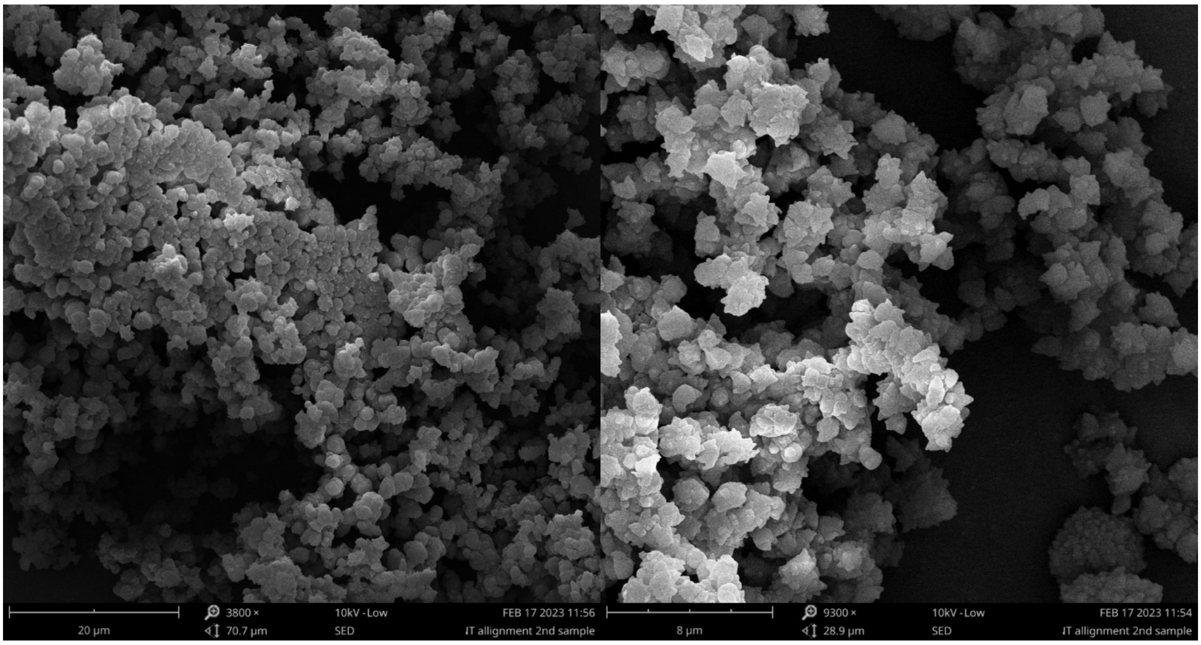

Figure S72. FE-SEM micrographs of (*P*)-**[5]Heli-TFP** COF with 2.5k and 9.3k magnification at 25 °C.

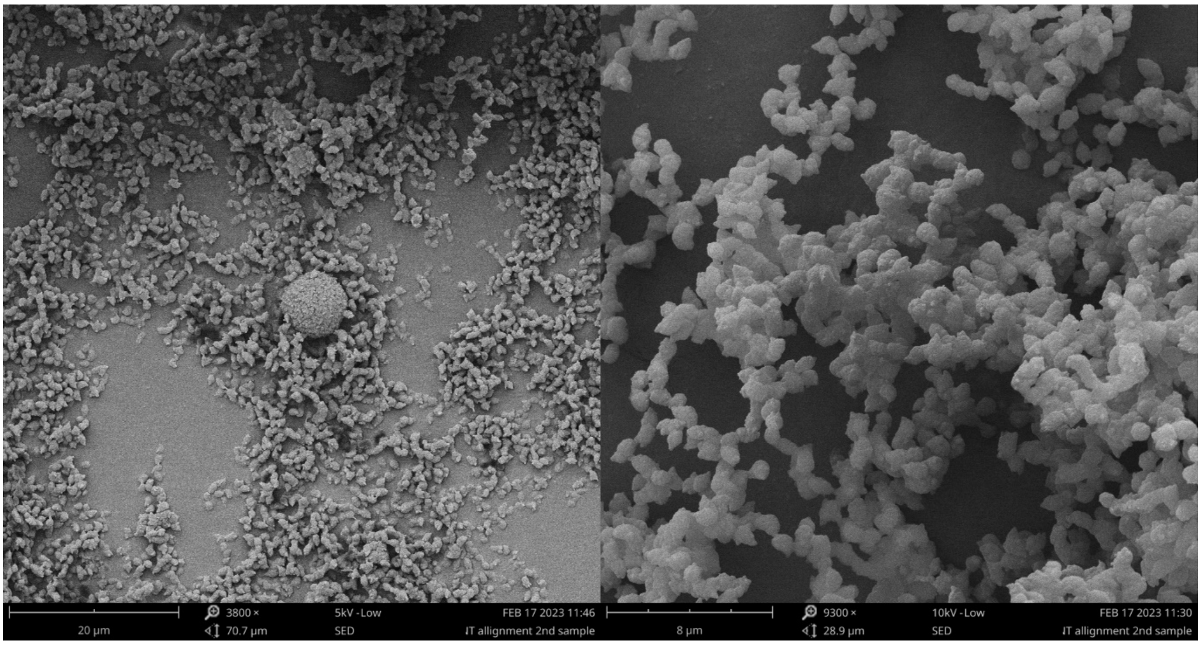

Figure S73. FE-SEM micrographs of (*M*)-**[5]Heli-TFP** COF with 2.5k and 9.3k magnification at 25 °C.

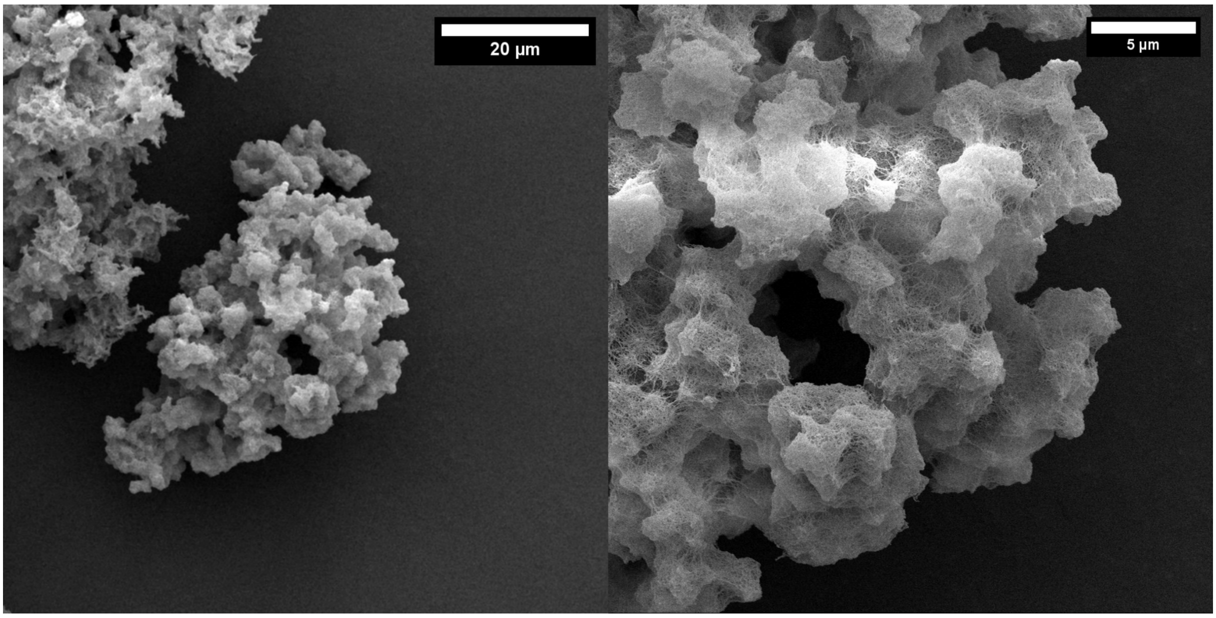

Figure S74. FE-SEM micrographs of *rac*-**[5]Heli-TFP** POP from (*R*,*R*)-(*P*/*M*)-**7** at 25 °C.

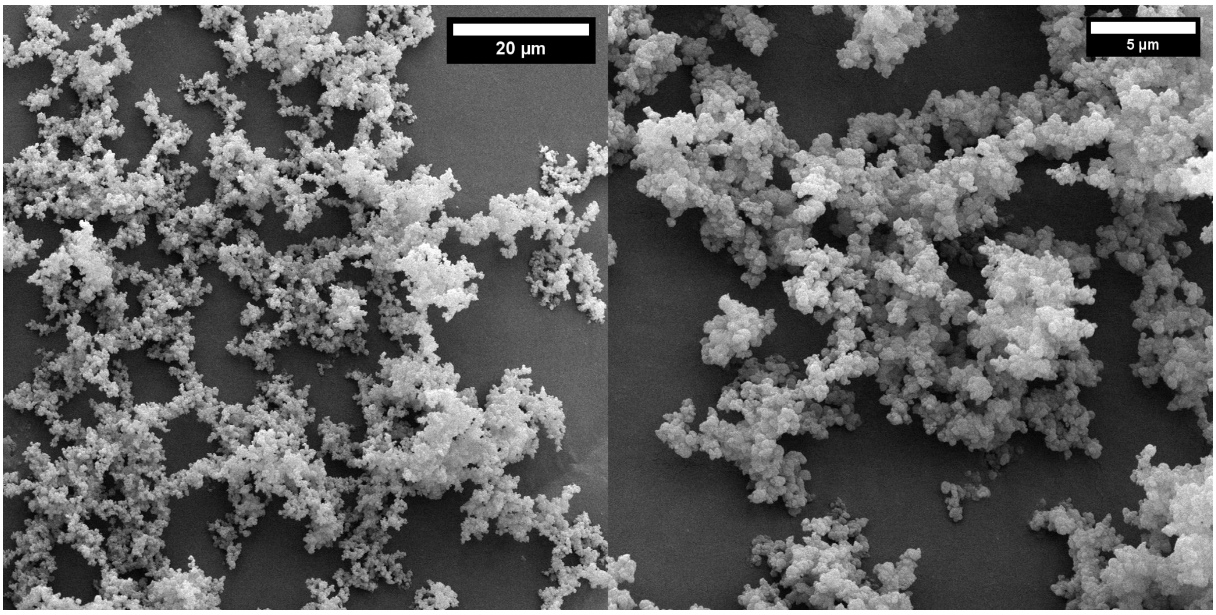

Figure S75. FE-SEM micrographs of (*P*)-**[5]Heli-TFP** POP from (*R*,*R*)-(*P*)-(+)-**7** at 25 °C.

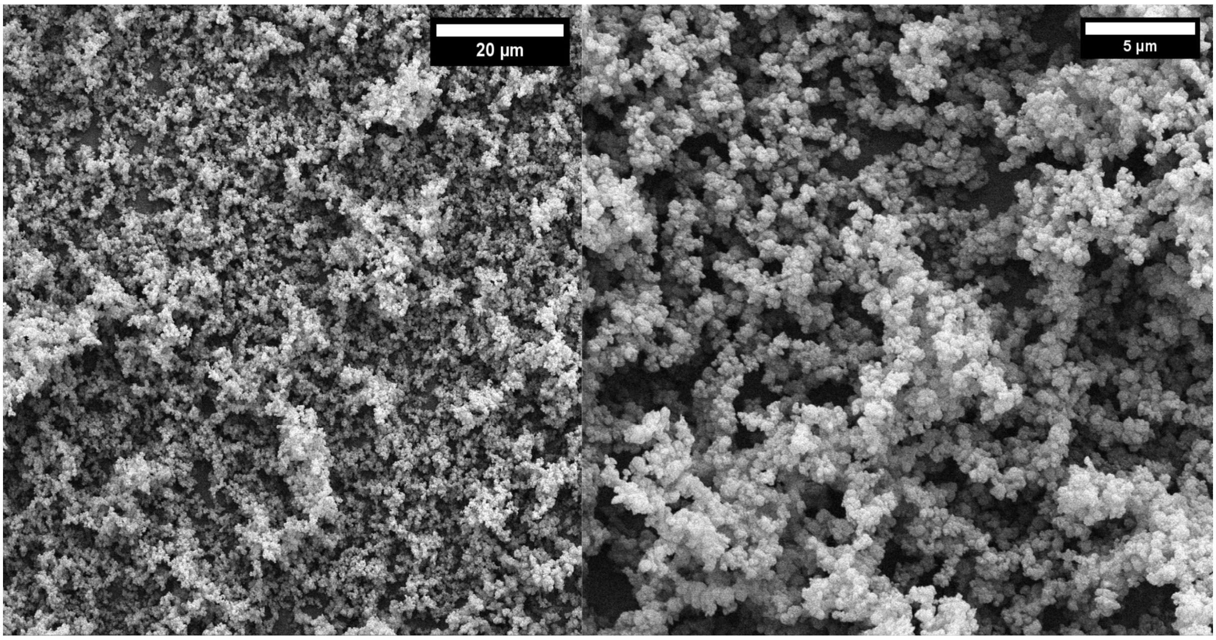

Figure S76. FE-SEM micrographs of (*M*)-**[5]Heli-TFP** POP from (*R*,*R*)-(*P*)-(+)-**7** at 25 °C.

S9. Atomic Force Microscopy

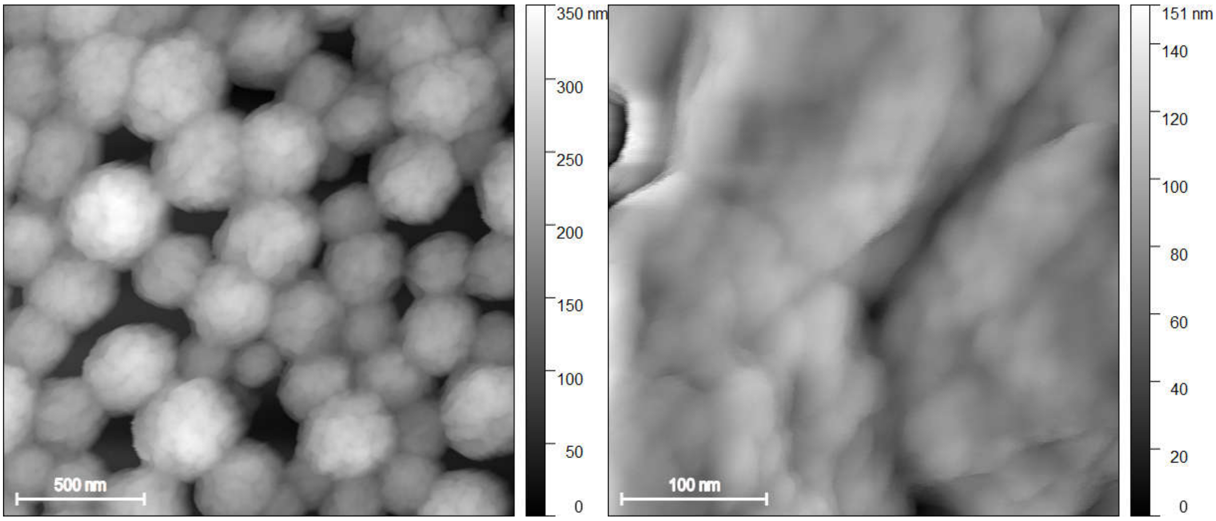

Figure S77. AFM analysis of *rac*-**[5]Heli-TFP** COF film with concentration of 1.2 mM of (±)-**3**.[13]

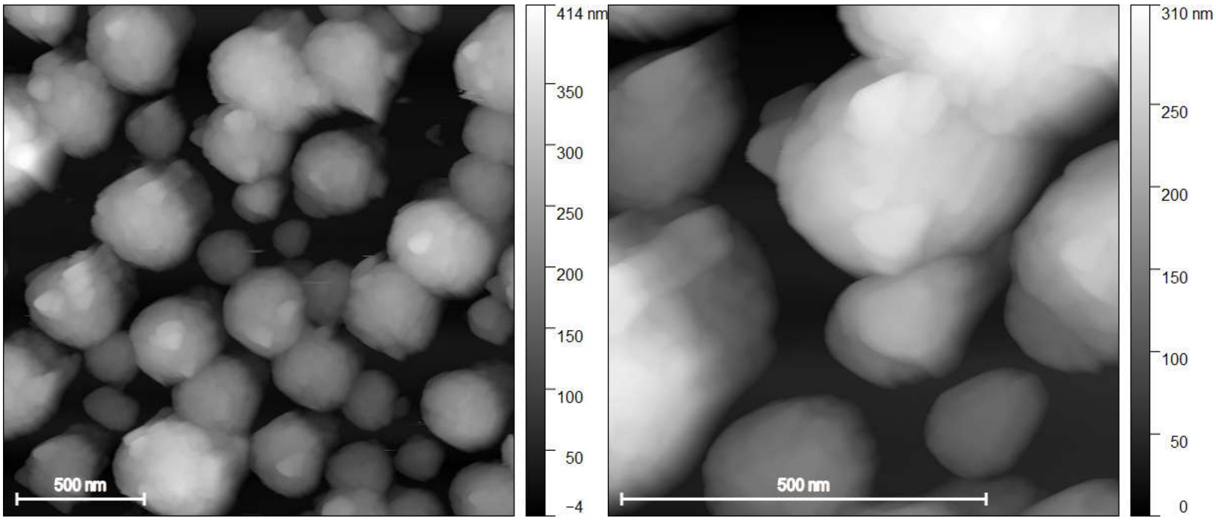

Figure S78. AFM analysis of (*P*)-**[5]Heli-TFP** COF film with concentration of 1.2 mM of (*P*)-(–)-**3**.[13]

S10. Elemental Analysis

All measurements were conducted in triplicate. Before analytical characterization, the samples were dried under vacuum (10^{-2} mbar) at 120 °C for 24 h and were immediately measured to diminish the amount of adsorbents inside the pores. Nevertheless, there is a significant difference between the calculated and the obtained elemental composition. This discrepancy has been documented in previous COF reports,[20] and it related to the high surface area of the porous materials.

Table S10. Calculated and experimental data of Elemental Analysis.

**Sample**	**Calculated (Cal)** **Experimental (Exp)**
rac-**[5]Heli-TFP** COF	Cal: C: 85.11%, H: 4.76%, N: 4.73%, O: 5.40% Exp: C: 82.79%, H: 4.68%, N: 4.88%
(*P*)-**[5]Heli-TFP** COF	Exp: C: 82.78%, H: 4.85%, N: 4.83%
(*M*)-**[5]Heli-TFP** COF	Exp: C: 82.80%, H: 4.85%, N: 4.83%
rac-**[5]Heli-TFB** COF	Cal: C: 89.97%, H: 5.03%, N: 5.00% Exp: C: 88.67%, H: 5.07%, N: 4.71%

S11. Thermal Gravimetric Analysis

Before analytical characterization, the samples were dried under vacuum (10^{-2} mbar) at 120 °C for 24 h and were immediately measured to diminish the amount of adsorbents inside the pores. The temperature was initially set at 30 ºC and held by 2 min. The heating ramp was established from 30 ºC to 800 ºC at 20 °C min^{-1}. Approximately 1 mg of material was used for each measurement. Onset temperatures for all materials are listed below.

Table S11. TGA onset temperature of COF powders.

Sample	Onset Temperature [°C]
rac-**[5]Heli-TFP** COF	396
(*P*)-**[5]Heli-TFP** COF	373
(*M*)-**[5]Heli-TFP** COF	372
rac-**[5]Heli-TFB** COF	393

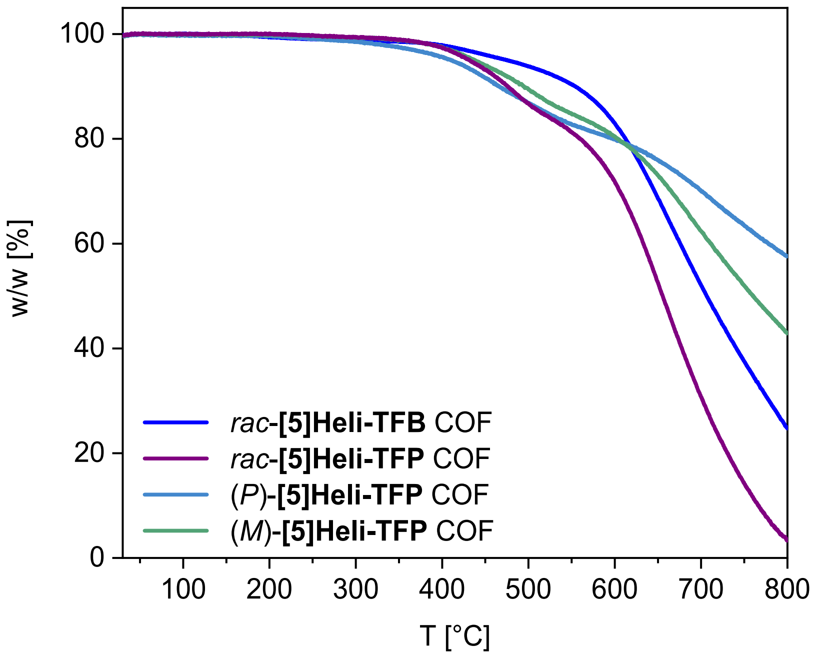

Figure S79. Thermogravimetric analysis of COFs. *rac*-**[5]Heli-TFB** COF (dark blue), *rac*-**[5]Heli-TFP** COF (purple), (*P*)-**[5]Heli-TFP** COF (light blue), and (*M*)-**[5]Heli-TFP** COF (green).

S12. UV-Vis Spectroscopy

For the diffuse reflectance UV-Vis spectra collection, the samples were mixed with $BaSO_4$ for dilution to avoid the detector saturation before each measurement.

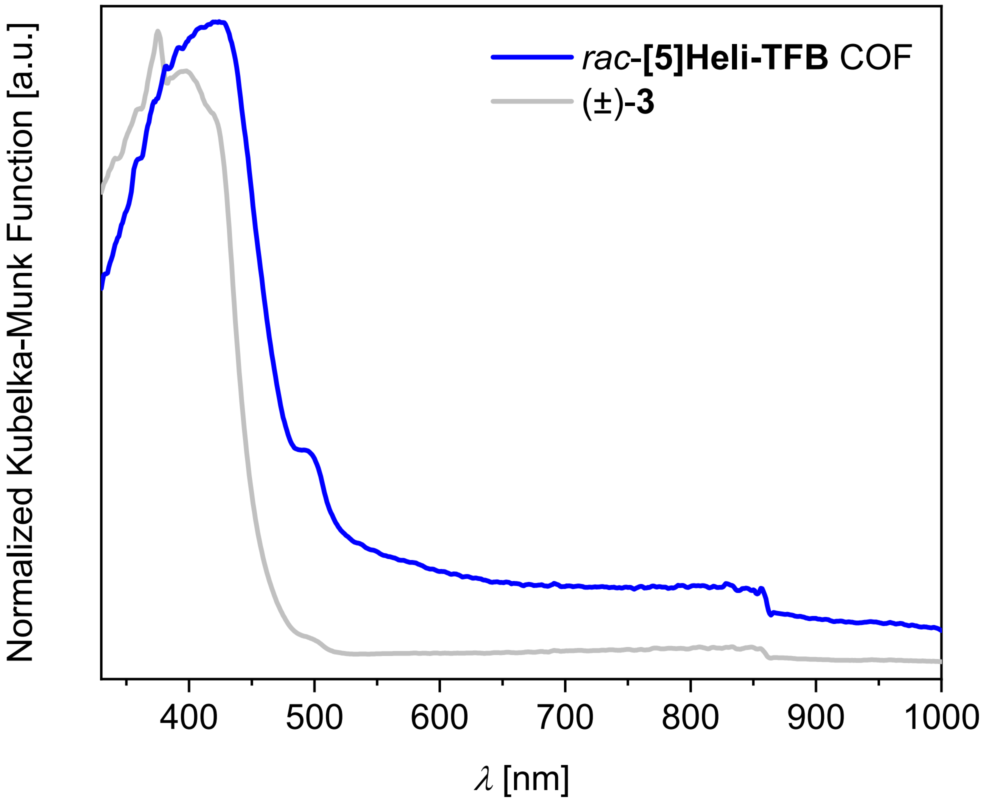

Figure S80. Normalized absorption spectra of solid *rac*-**[5]Heli-TFB** COF (dark blue), and building block (±)-**3** (grey), samples recorded by diffuse-reflectance UV-Vis spectroscopy, and converted using the Kubelka–Munk function.

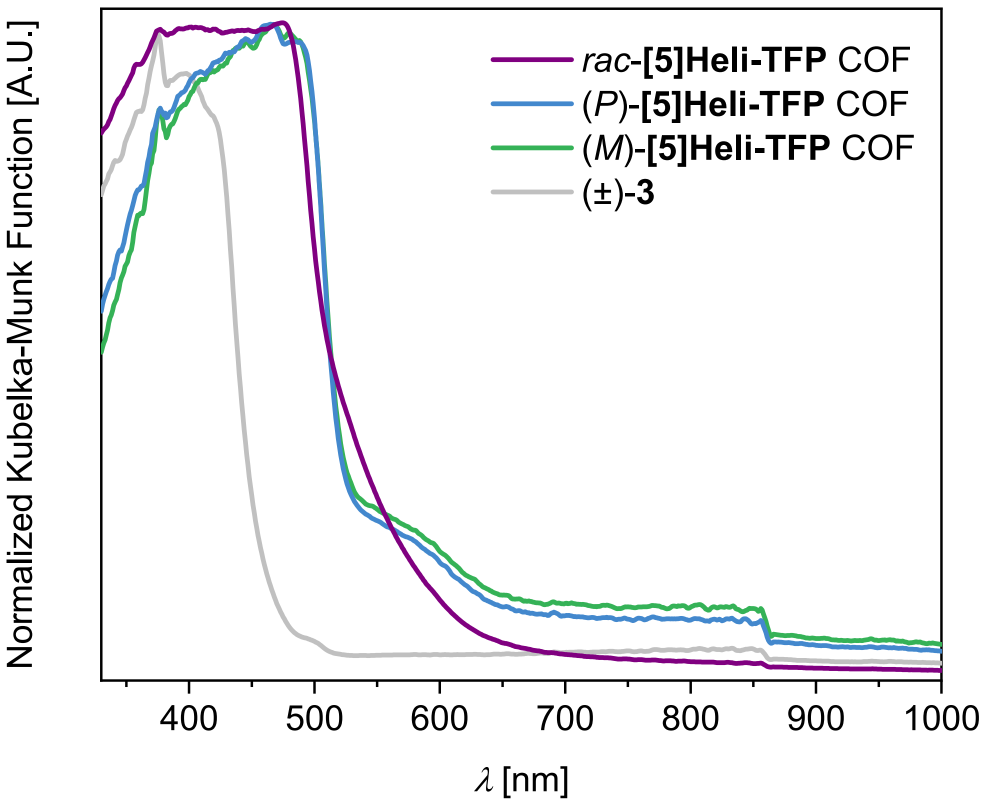

Figure S81. Normalized absorption spectra of building block (±)-**3** (grey), and *rac*-**[5]Heli-TFP** COF (purple), (*P*)-**[5]Heli-TFP** COF (light blue), (*M*)-**[5]Heli-TFP** COF (green) samples recorded by diffuse-reflectance UV-Vis spectroscopy, and converted using the Kubelka–Munk function.

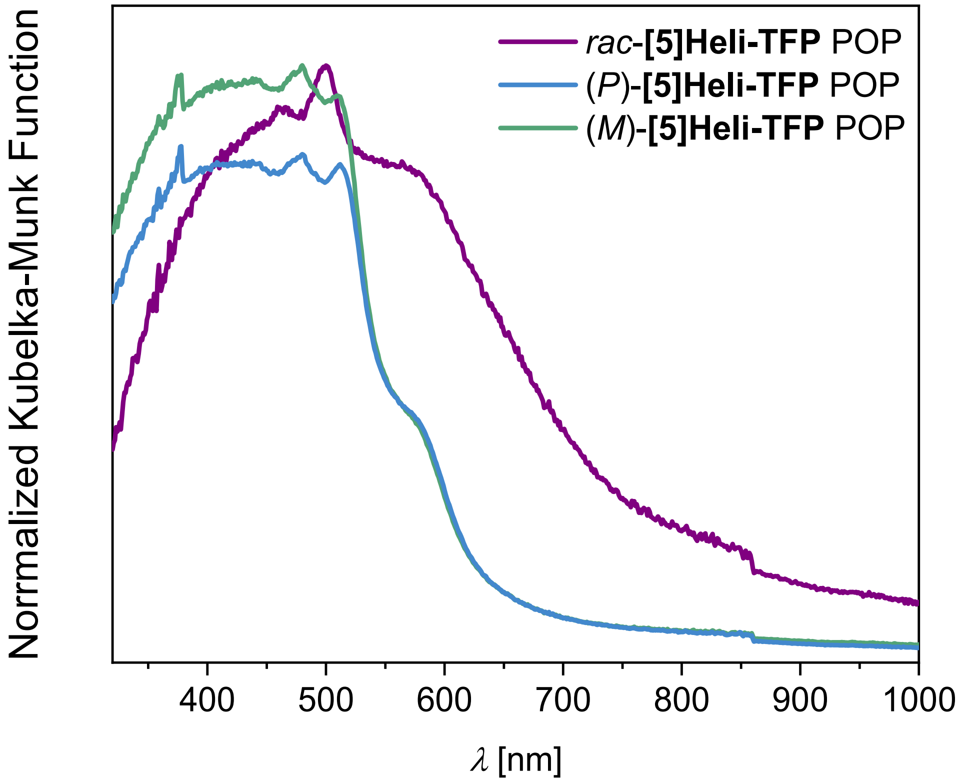

Figure S82. Normalized absorption spectra of *rac*-**[5]Heli-TFP** POP, (*P*)-**[5]Heli-TFP** POP (blue), (*M*)-**[5]Heli-TFP** POP (green) samples recorded by diffuse-reflectance UV-Vis spectroscopy and converted using the Kubelka–Munk function.

S13. FT-IR Spectroscopy

Before analytical characterization, the samples were dried under vacuum (10^{-2} mbar) at 120 °C for 24 h and were immediately measured to diminish the amount of humidity in the samples.

For the TFB-base sample, the characteristic C−H-stretch band TFB-aldehyde at 2870 cm^{-1} and the C=O-stretch band at 1700 cm^{-1} were not detected in the resulting material *rac*-**[5]Heli-TFB** COF. Instead, the characteristic C=N-stretch is visible around 1600 cm^{-1} (1614 cm^{-1} *rac*-**[5]Heli-TFB** COF) (Figure S83).

In the other hand, the TFP-based materials do not exhibit the characteristic C=O-stretch band TFP-aldehyde at 1695 cm^{-1}. Instead, the C=O-stretch of the keto-enamine is visible around 1600 cm^{-1} (1582 cm^{-1} *rac*-**[5]Heli-TFP** COF, 1600 cm^{-1} (*P*)-**[5]Heli-TFP**, 1600 cm^{-1} (*M*)-**[5]Heli-TFP** COF). Furthermore, the C−N-stretch of the keto-enamine around 1280 cm^{-1} (1285 cm^{-1} *rac*-**[5]Heli-TFP** COF, 1288 cm^{-1} (*P*)-**[5]Heli-TFP**, 1289 cm^{-1} (*M*)-**[5]Heli-TFP** COF) (Figure S84).

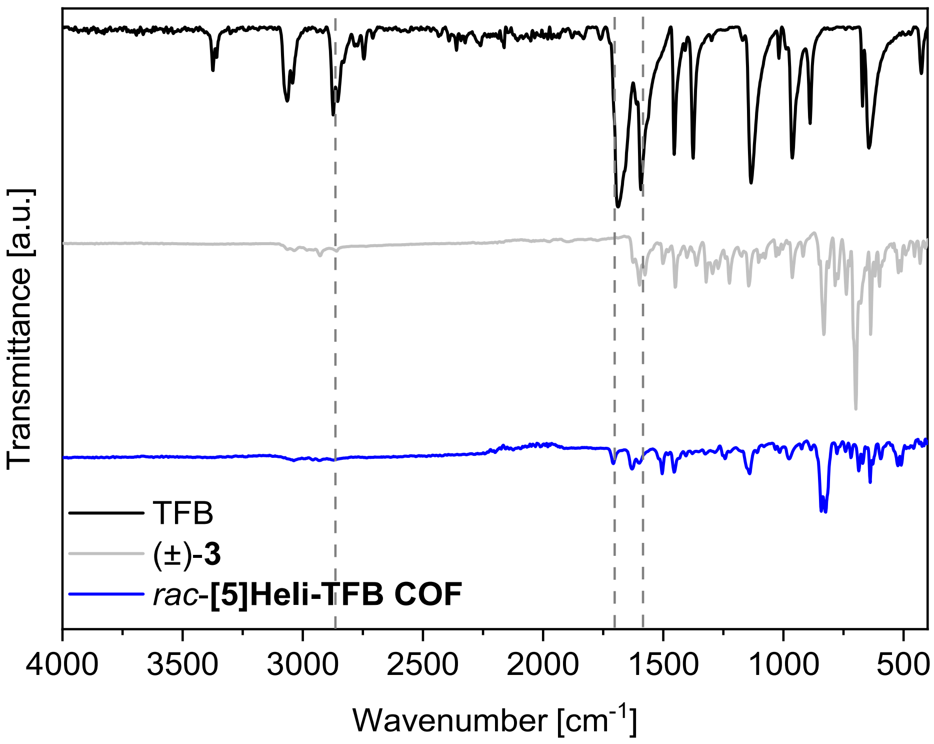

Figure S83. FT-IR spectra of building blocks TFB (black), (±)-**3** (grey), and *rac*-**[5]Heli-TFB** COF (dark blue).

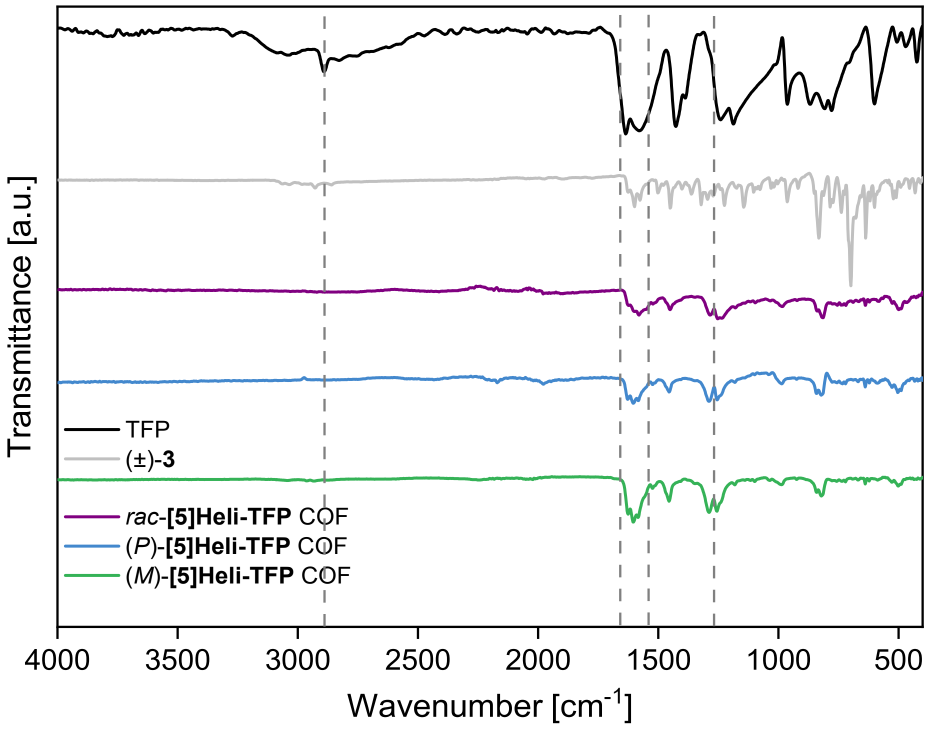

Figure S84. FT-IR spectra of building blocks TFP (black), (±)-**3** (grey), *rac*-**[5]Heli-TFP** COF (dark blue), *rac*-**[5]Heli-TFP** COF (purple), (*P*)-**[5]Heli-TFP** COF (light blue), and (*M*)-**[5]Heli-TFP** COF (green).

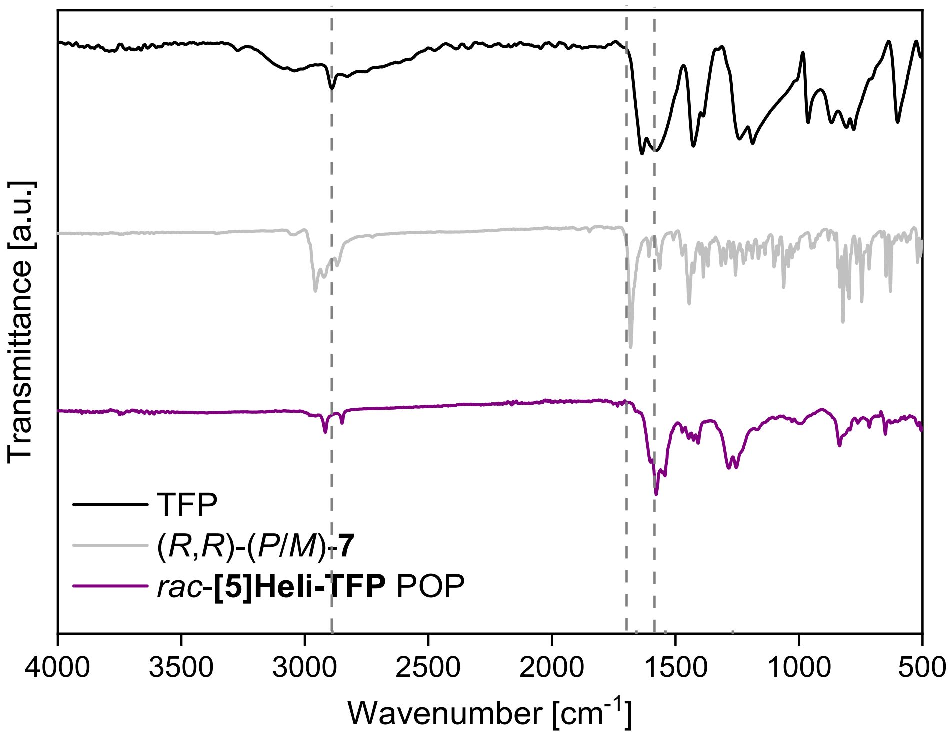

Figure S85. FT-IR spectra comparison of building blocks TFP (black), (*R*,*R*)-(*P*/*M*)-**7** (grey), and the amorphous polymer *rac*-**[5]Heli-TFP** POP (purple).

S14. CP-MAS NMR Analysis

Cross-Polarization Magic-Angle-Spinning solid-state NMR studies of the *rac*-**[5]Heli-TFB** COF and *rac*-**[5]Heli-TFP** COF show sharp signals indicating a degree of periodicity. Since each material contains multiple carbons in a similar chemical environment, the signals tend to overlap. The observed signals below 40 ppm are attributed to the methyl groups. Signals in the range of 120—140 ppm are assigned to aromatic carbons from the [5]helicene, while those around 150—160 ppm correspond to –C=N imines and aromatic carbons linked to nitrogen –C–N. Signals around 180—200 ppm are attributed to ketoenamine linkages C=O. Additionally, since –CHO aldehydes signals were not detected in the IR spectra, the significant downfield signals (*) could be associated with imine carbons with strong electronic effects inside the non-planar frameworks.[21]

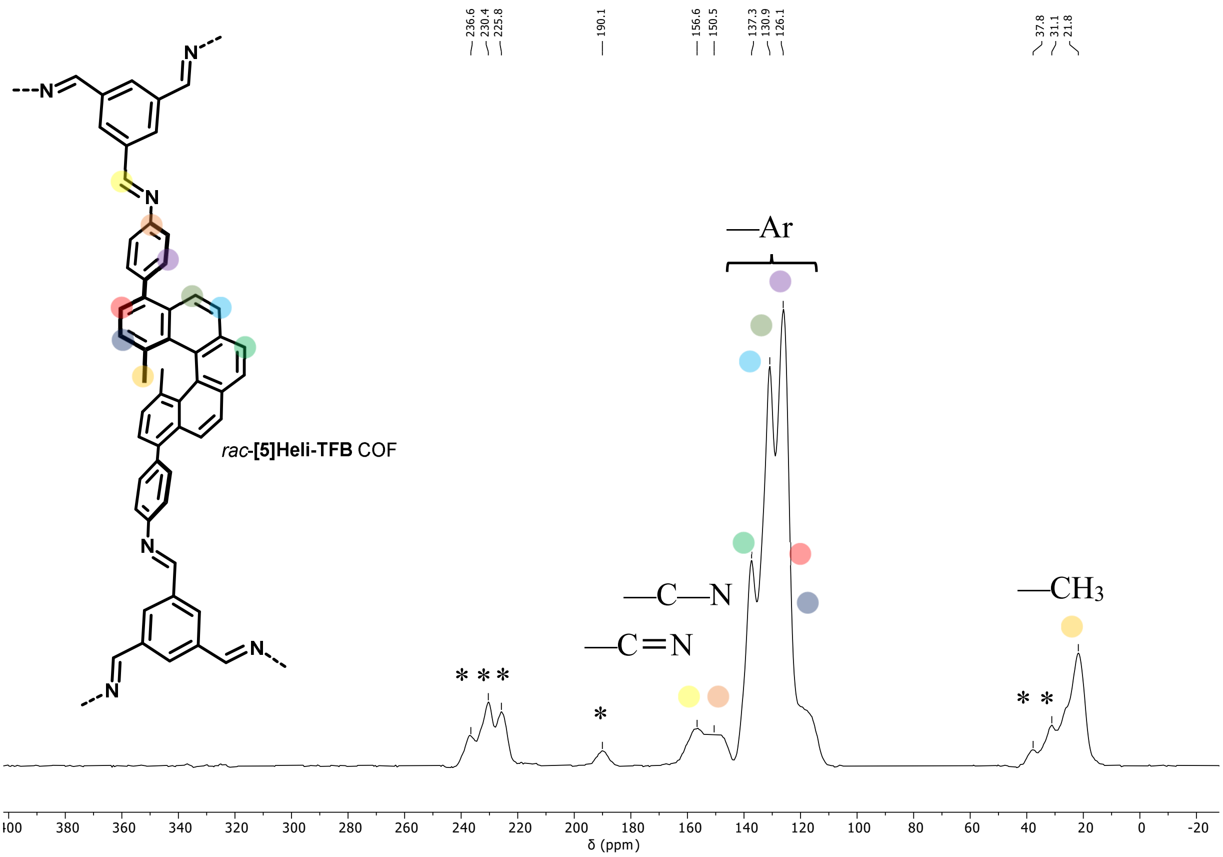

Figure S86. CP-MAS NMR of *rac*-**[5]Heli-TFB** COF; spinning side-bands are marked with asterisks.

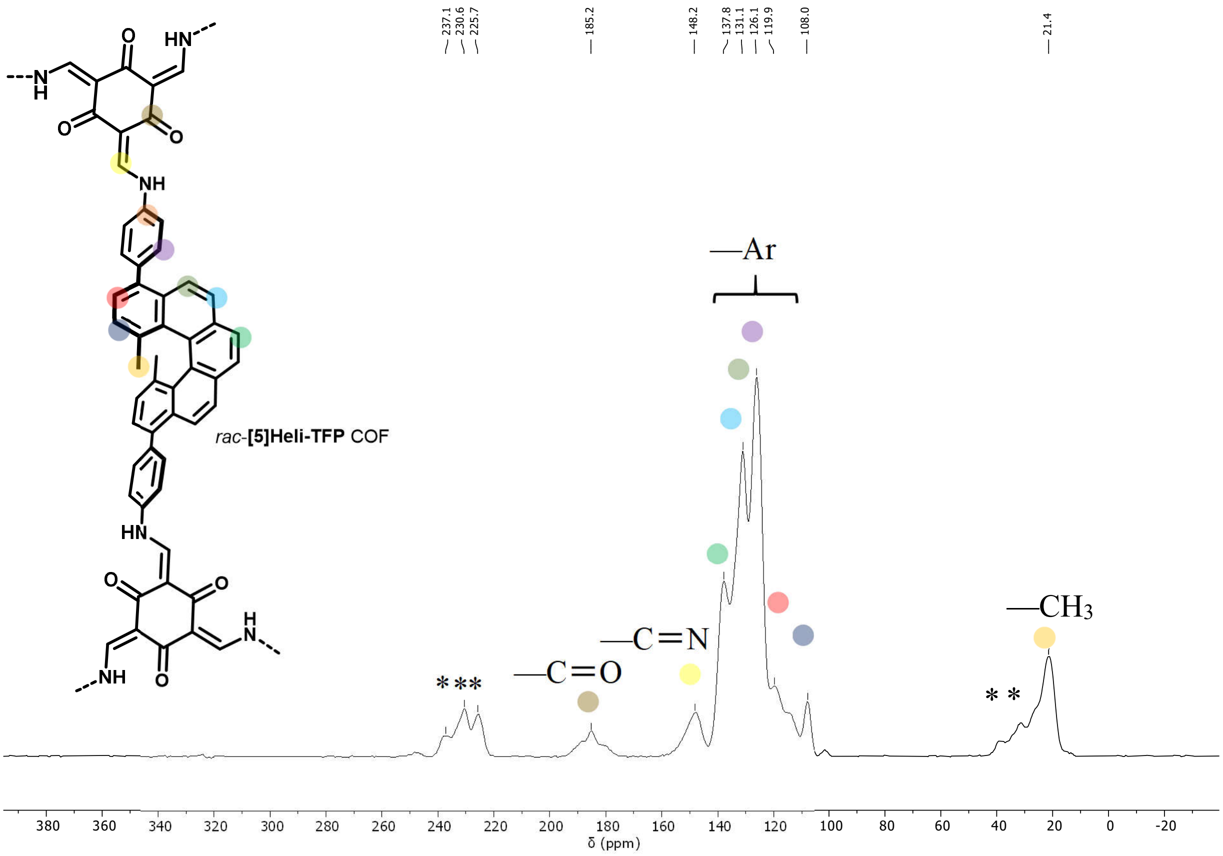

Figure S87. CP-MAS NMR of *rac*-**[5]Heli-TFP** COF; spinning side-bands are marked with asterisks.

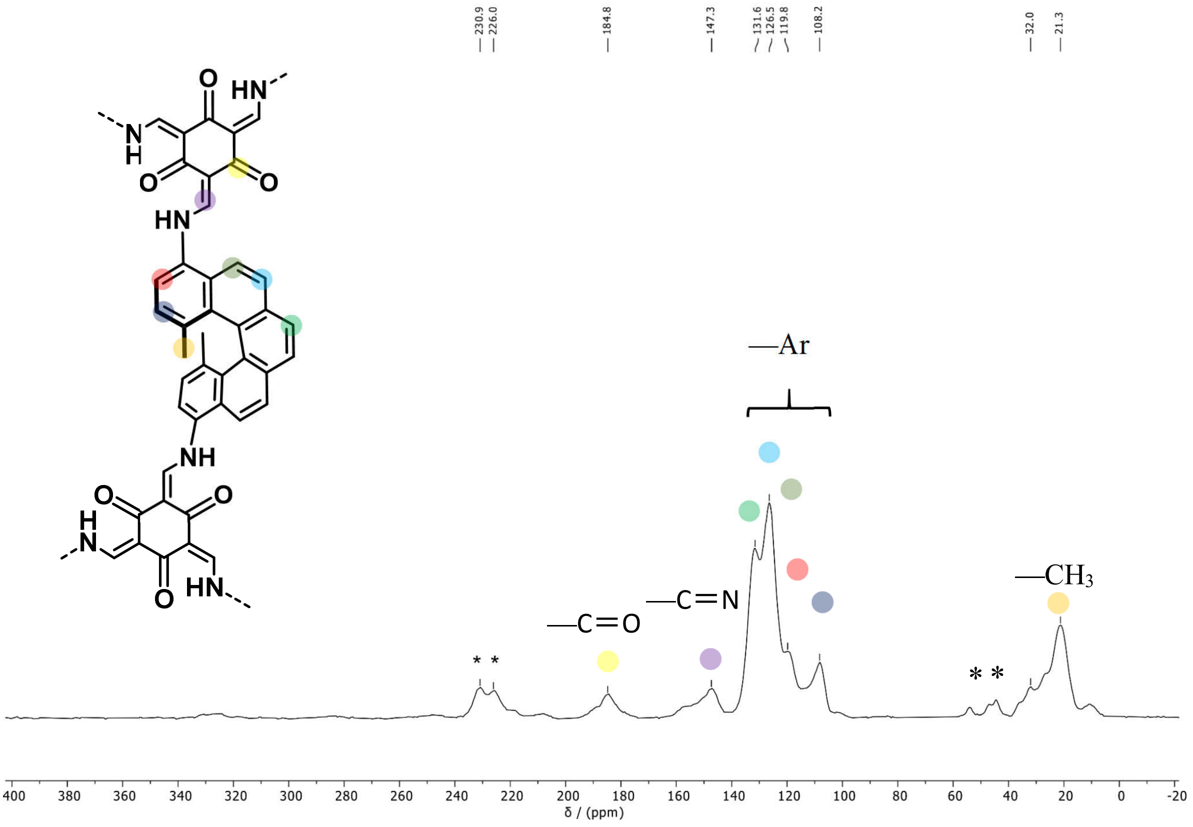

Figure S88. CP-MAS NMR of *rac*-**[5]Heli-TFB** POP from (*R*,*R*)-(*P*/*M*)-**5**.

S15. PXRD and Structure Modelling of COF materials

Structural models for the racemic and homochiral **HeliCOFs** have been proposed employing a combination of crystal building simulations with different stacking modes for hexagonal 2D lattices using Accelrys' Materials Studio program package.[11] Due to the non-planar structure of [5]helicene building blocks, and their respective (*P*)- and (*M*)- configurations, the crystal building models were carried out in the primitive symmetry *P*1. Furthermore, the structural models were optimized using the Forcite module with an 'ultra-fine' optimization with Universal Force Field and 'Smart' algorithm.[11] The experimental diffractogram patterns were fitted using Pawley refinement to computational COF models, and no constraints were placed during the optimizations.

S15.1 Racemic [5]Heli-TFB and [5]Heli-TFP COFs

We first aimed to model racemic hexagonal lattices with AA eclipsed stacking, however, the force field optimization did not lead to the target geometry due to the non-planar symmetry of the building blocks. Only optimizations towards AA-inclined and AA-serrated unit cells converged successfully. Such stacking modes have been described in the literature and various names have been used. In this work, we followed the report of Van Speybroeck and coworkers.[22] Furthermore, as an alternative stacking mode an AB-staggered unit cell was included. In all cases, the orientation of the [5]helicene *fjord* region in (*P*)- and (*M*)-configurations are alternating over the periodic layers. Based on the comparison of the experimental data and the simulated PXRD patterns from the different stacking modes, the most suitable stacking mode is AA-inclined for *rac*-**[5]Heli-TFB** COF (see figures S89 and S92). Similarly, the simulated PXRD patterns and Pawley refinement from AA-inclined stacking mode suggest the best fit for *rac*-**[5]Heli-TFP** COF, but show also the partial character of AA-serrated stacking mode (figures S93 and S96). In this case, both AA-inclined and AA-serrated modes could be present.

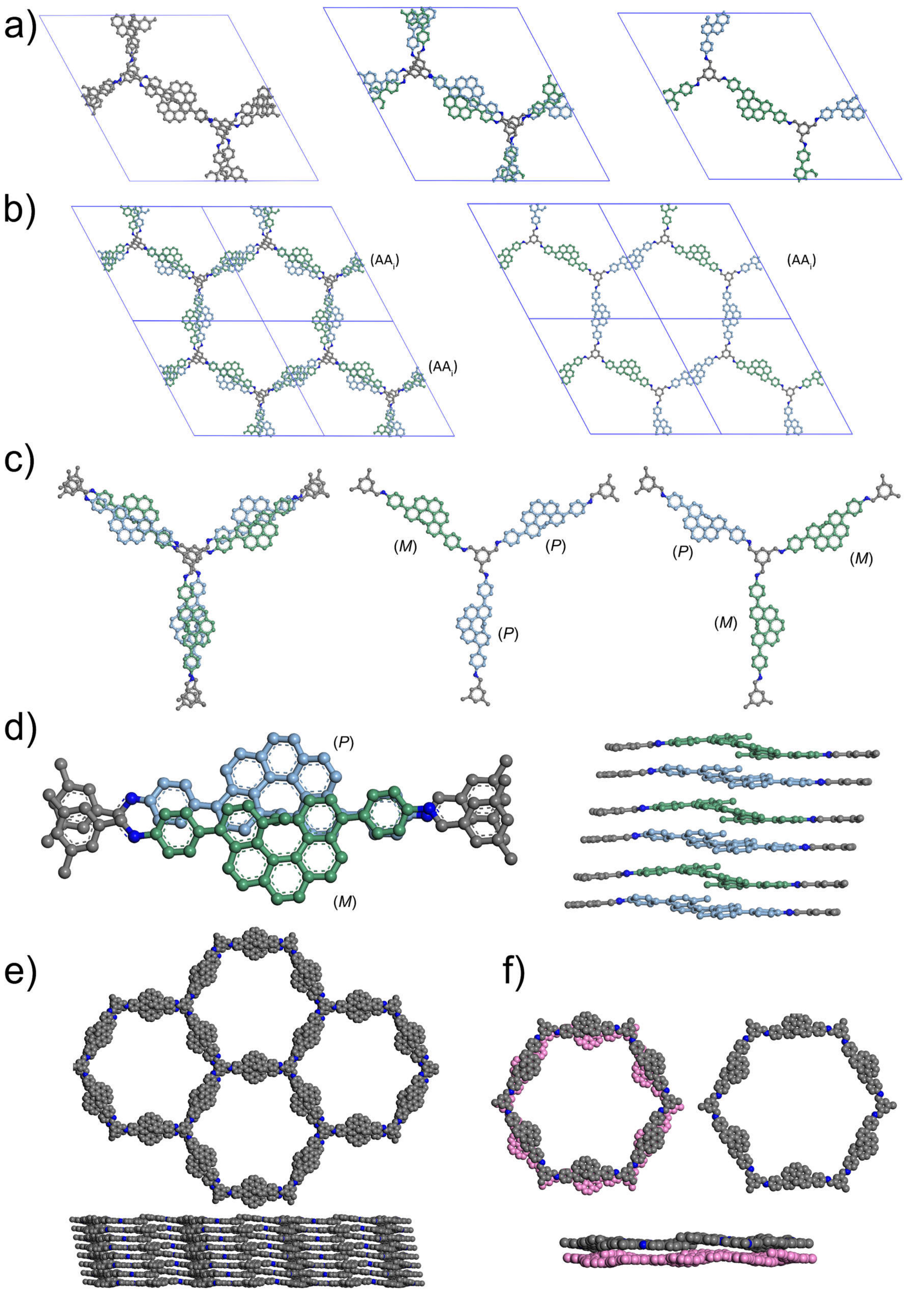

Figure S89. Pawley refinement structural model of *rac*-**[5]Heli-TFB** COF with AA-inclined mode ((*rac*)-[5]helicene linkers are alternating in (*P*)- or (*M*)- configuration over the lattices, two lattices are considered and remarked in different colors for better visualization, hydrogen atoms are omitted for clarity; a) unit cell of *rac*-**[5]Heli-TFB** COF and the remark of two different layers; b) hexagonal arrange from 4-unit cells and one single layer; c) basic node and linker connectivity extracted from the repetitive lattices; d) inclined stacking mode of the *rac*-[5]helicene linkers from the repetitive layers; e) top and side view of the corresponding refined structure; f) example of single hexagonal pore extracted from the refined model.

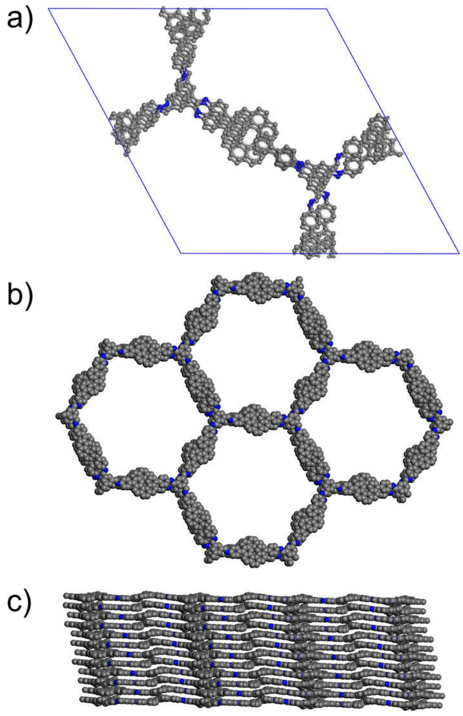

Figure S90. Pawley refinement structural model of *rac*-**[5]Heli-TFB** COF with AA-serrated mode *rac*-[5]helicene linkers are alternating in (*P*)- or (*M*)- configuration over the lattices, four lattices are considered, hydrogen atoms are omitted for clarity; a) complete unit cell of optimized in Material Studio; c) top view of the corresponding refined structure; d) side view of the corresponding refined structure.

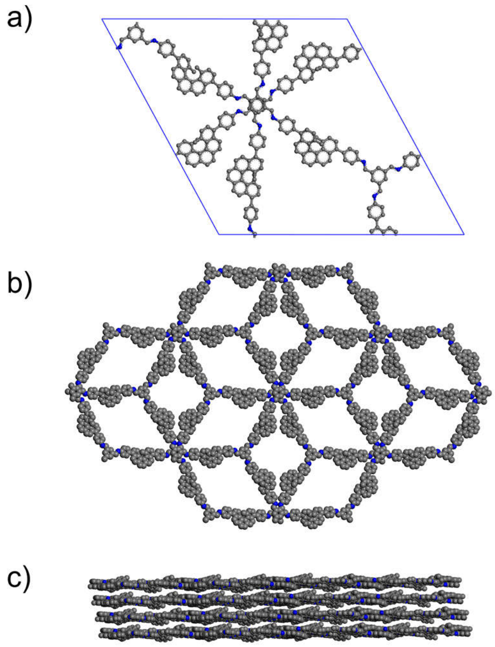

Figure S91. Pawley refinement structural model of *rac*-**[5]Heli-TFB** COF with AB-staggered mode *rac*-[5]helicene linkers are alternating in (*P*)- or (*M*)- configuration over the lattices, four lattices are considered, hydrogen atoms are omitted for clarity; a) complete unit cell of optimized in Material Studio; c) top view of the corresponding refined structure; d) side view of the corresponding refined structure.

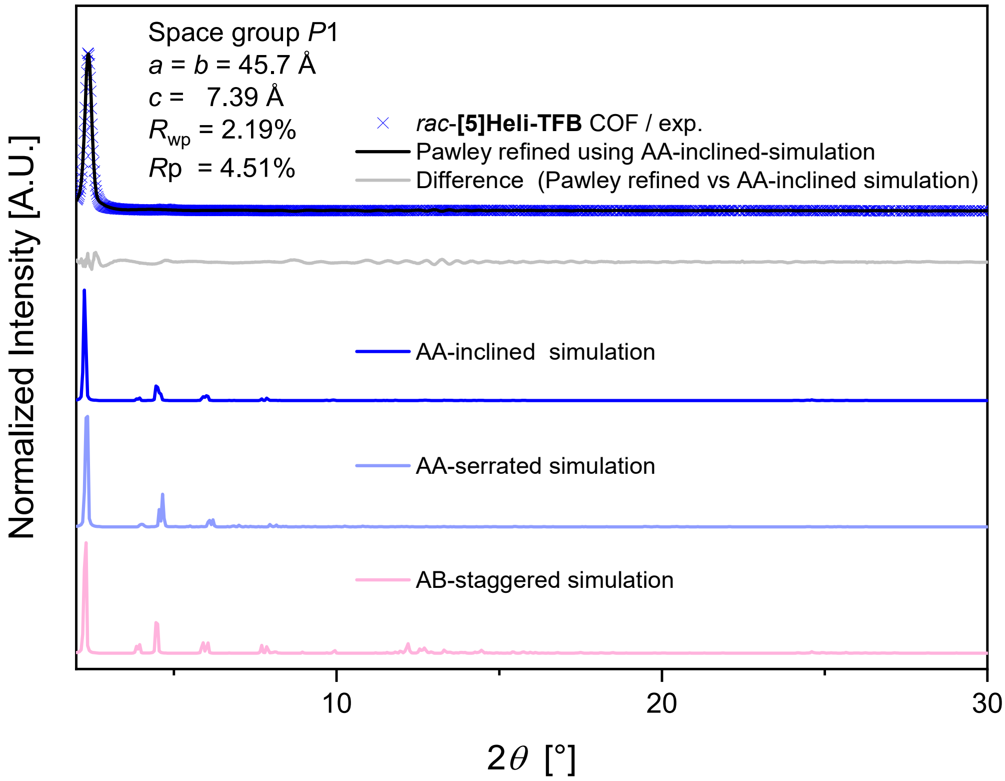

Figure S92. Experimental PXRD data and simulated PXRD patterns from the different structural models of *rac*-**[5]Heli-TFB** COF: experimental PXRD pattern (blue crosses), Pawley refinement (black line) using an optimized structural model AA-inclined (inset), difference between Pawley refinement and AA-inclined simulation (gray), simulated PXRD pattern from AA-inclined lattice (blue), simulated PXRD pattern from AA-serrated lattice (green), simulated PXRD pattern from AB stacking lattice (pink).

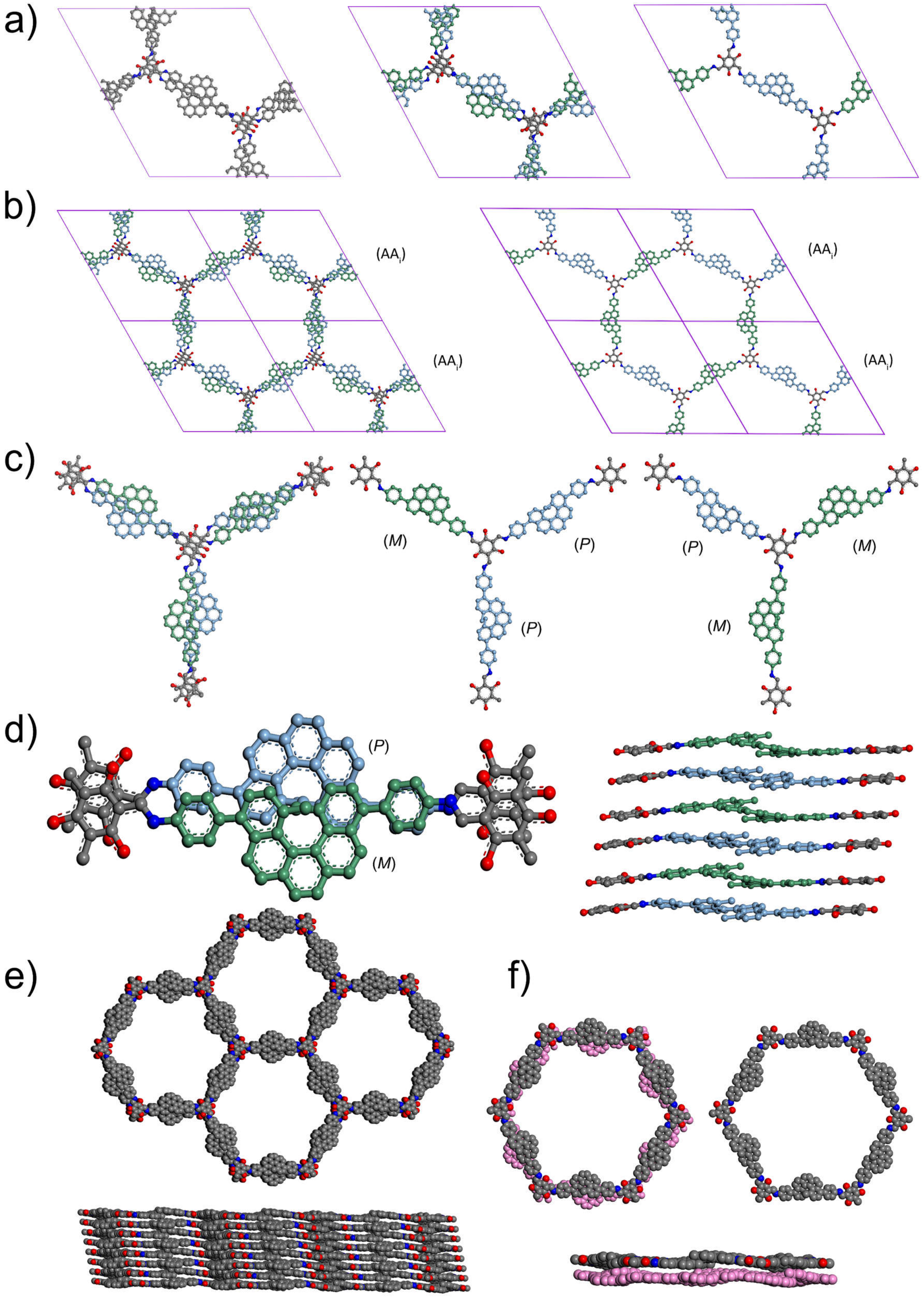

Figure S93. Pawley refinement structural model of *rac*-**[5]Heli-TFP** COF with AA-inclined mode *rac*-[5]helicene linkers are alternating in (*P*)- or (*M*)- configuration over the lattices, two lattices are considered and remarked in different colors for better visualization (hydrogen atoms are omitted for clarity); a) unit cell of *rac*-**[5]Heli-TFP** COF and remark of two different layers; b) hexagonal arrange from 4-unit cells and one single layer; c) basic node and linker connectivity extracted from the repetitive lattices; d) inclined stacking mode of the *rac*-[5]helicene linkers from the repetitive layers; e) top and side view of the corresponding refined structure; f) example of single hexagonal pore extracted from the refined model.

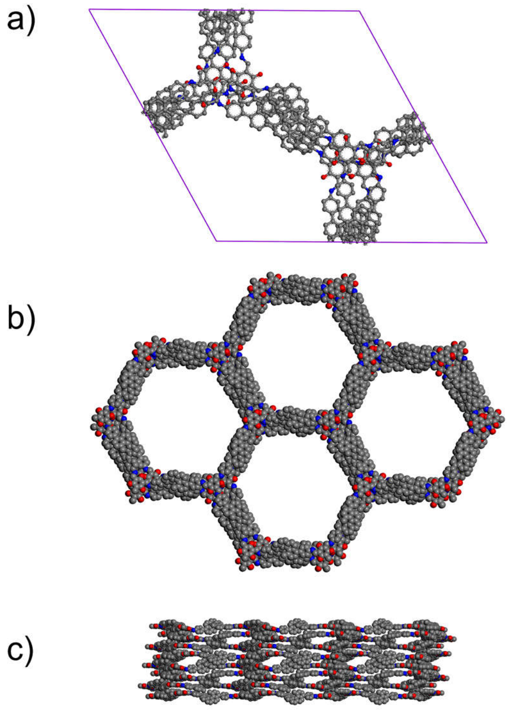

Figure S94. Pawley refinement structural model of *rac*-**[5]Heli-TFP** COF with AA-serrated mode *rac*-[5]helicene linkers are alternating in (*P*)- or (*M*)- configuration over the lattices, four lattices are considered, hydrogen atoms are omitted for clarity; a) complete unit cell of optimized in Material Studio; c) top view of the corresponding refined structure; d) side view of the corresponding refined structure.

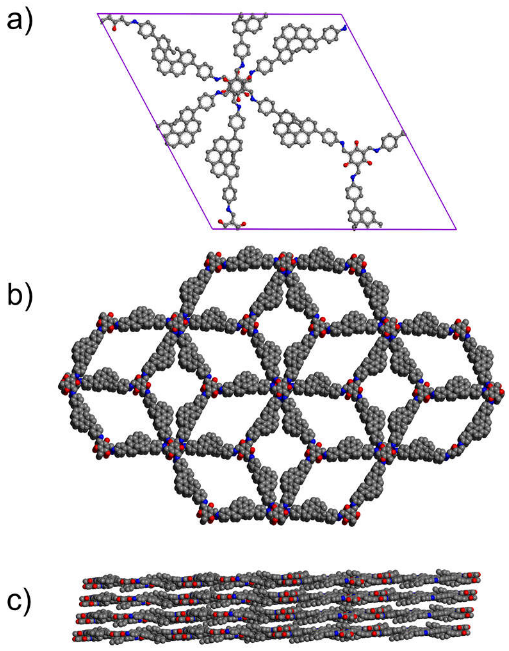

Figure S95. Pawley refinement structural model of *rac*-**[5]Heli-TFP** COF with AB-staggered mode *rac*-[5]helicene linkers are alternating in (*P*)- or (*M*)- configuration over the lattices, four lattices are considered, hydrogen atoms are omitted for clarity; a) complete unit cell of optimized in Material Studio; c) top view of the corresponding refined structure; d) side view of the corresponding refined structure.

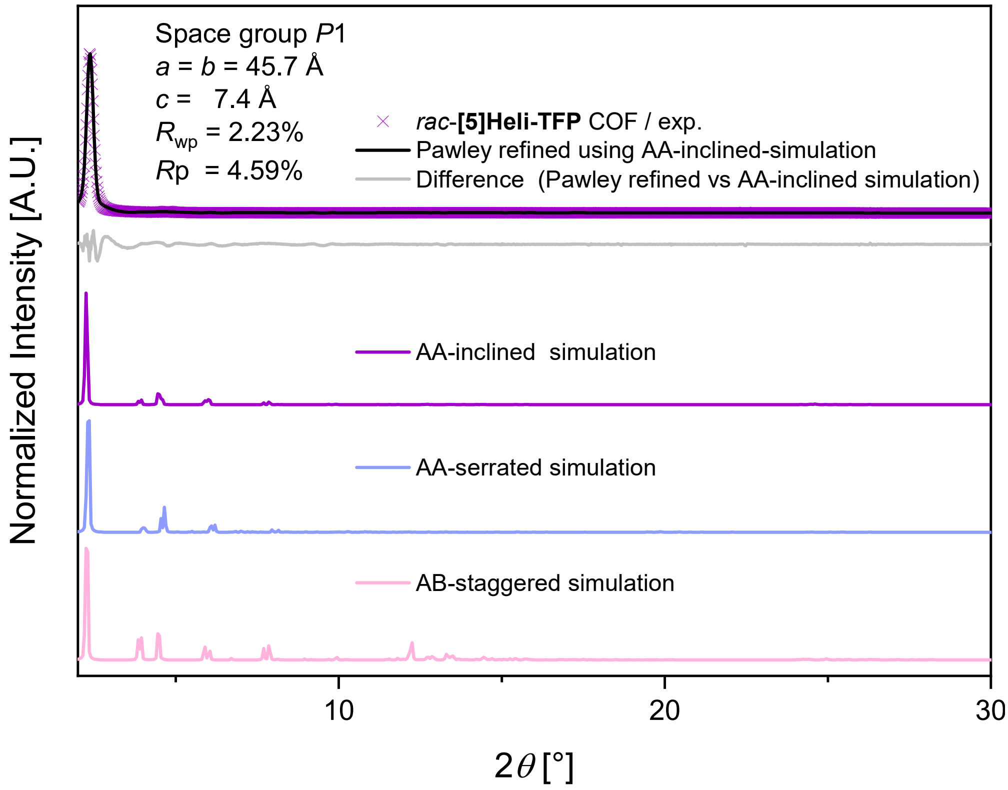

Figure S96. Experimental PXRD data and simulated PXRD patterns from the different structural models of *rac*-**[5]Heli-TFP** COF: experimental PXRD pattern (purple crosses), Pawley refinement (black line) using an optimized structural model AA-inclined (inset), difference between Pawley refinement and AA-inclined simulation (gray), simulated PXRD pattern from AA-inclined lattice (purple), simulated PXRD pattern from AA-serrated lattice (blue), simulated PXRD pattern from AB stacking lattice (pink).

S15.2 Homochiral HeliCOFs

As in the racemic analogs, only optimization towards hexagonal AA-inclined and AA-serrated unit cells converged successfully. However, the second reflex near $2\theta = 4.2°$ did not show a good agreement. The report of Heine and co-workers inspired us to consider a multilayer statistical representation of the asymmetric homochiral crystalline lattices of each (*P*)- and (*M*)-**[5]HeliTFP** COFs.[23] Hence, we propose that both homochiral **[5]Heli-TFP** COFs exhibit mostly a statistical combination of the AA-inclined and AB-staggered stacking mode, noted as $(AA_i:AB)^{stat}$, having both stacking modes present in the asymmetric homochiral crystals. Based on the simulated PXRD patterns, the $(AA_i:AB)^{stat}$ stacking mode is the most suitable for both homochiral COFs (see figures S98 and S101). Other hexagonal stacking modes were considered and exhibited a relative intense main reflex at $2\theta = 2.4°$, not matching the experimental

PXRD patterns (Tables S12 and S13). From these stacking models, the AA-serrated model showed a deviated simulated PXRD pattern with similar relative intensities to the experimental pattern, but the Pawley refinement agreement factors do not support this model ((*M*)-COF: R_{wp} = 12.17%, R_p = 4.85%, see Table S12 and (*P*)-COF: R_{wp} = 17.24%, R_p = 7.84%, see Table S13). However, the simulation of the non-planar geometries of homochiral lattices by force field empirical parameters could lead to deviations from experimental and calculated PXRD patterns. In our main proposed statistical $(AA_i:AB)^{stat}$ model, the hexagonal AA-inclined stacking mode is structurally alternating with the AB-staggered mode. Furthermore, the alternating layers in the homochiral AB lattices are not aligned perfectly above the pore center of the subsequent AA layers in order to decrease the proximity of enantiopure [5]helicene cores, reducing steric hindrance between the homochiral 2D layers. This is supported by the N_2 sorption isotherms from the homochiral samples where a lower N_2 uptake compared to the racemic case was observed—in agreement with the double statistical $(AA_i:AB)^{stat}$ model.

Furthermore, we noticed the difference in the relative intensity of the reflex at $2\theta = 4.2°$ for the two homochiral (*P*)- and (*M*)-**[5]Heli-TFP** COFs. Based on our diastereomeric excess analysis (see section S15), the parent building block (*R*,*R*)-(*P*)-(+)-**5** for the synthesis of the corresponding (*P*)-**[5]Heli-TFP** COF contained triple the amount of the opposite enantiomer as an impurity (< 0.7%) as compared to the parent building block (*R*,*R*)-(*M*)-(–)-**5** for the synthesis of (*M*)-**[5]Heli-TFP** COF (< 0.2%). Possibly the difference in diastereomeric excess can cause the relative intensity difference in the second reflex near $2\theta = 4.3°$ between both homochiral COFs. Based on the good agreement of the dataset of opposite chirality COFs from ECD spectroscopy, and the identical behavior in N_2 sorption, similar features in HR-TEM imaging, and identical chemical composition, we have high confidence that the relative intensity difference in the PXRD patterns is due to the above-mentioned reason and the high sensitivity of this method.

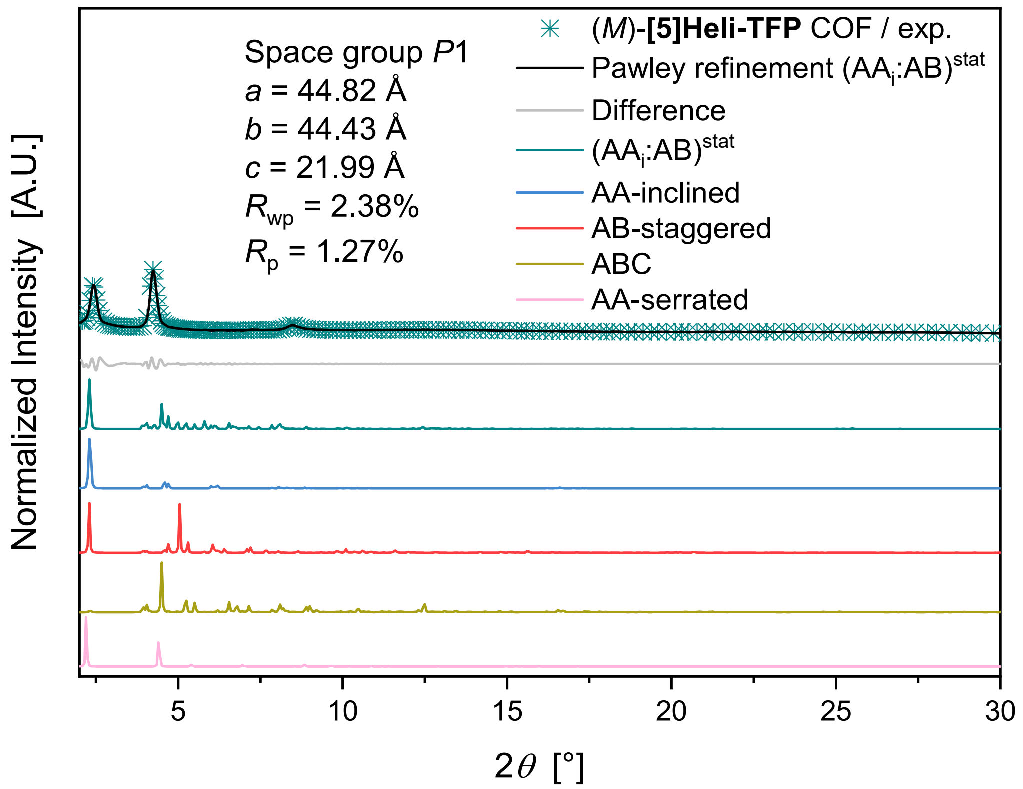

Figure S97. Experimental and simulated PXRD patterns from the different structural models of (*M*)-**[5]Heli-TFP** COF: experimental PXRD pattern (green asterisk), Pawley refinement (black line) using an optimized structural model $(AA_i{:}AB)^{stat}$, difference between Pawley refinement and $(AA_i{:}AB)^{stat}$ stacking simulation (gray), simulated PXRD pattern from $(AA_i{:}AB)^{stat}$ stacking model (green), simulated PXRD pattern from AA-inclined lattice (blue), simulated PXRD pattern from AB-staggered lattice (red), simulated PXRD pattern from ABC lattice (yellow), simulated PXRD pattern from AA-serrated lattice (pink).

Table S12. Structural models of (*M*)-**[5]Heli-TFP** COF: calculated lattice parameters and corresponding agreement factors from the structural models with different stacking mode.

**Stacking model**	**Lattice parameters [Å]**	**Agreement factors (Pawley refined)**
AA-inclined	a = 44.82, b = 44.43, c = 11.12	R_{wp} = 27.30% R_p = 17.65%
AB-staggered	a = 44.82, b = 44.43, c = 17.92	R_{wp} = 26.54% R_p = 25.03%
AA-serrated	a = 42.34, b = 44.15, c = 12.15	R_{wp} = 12.17% R_p = 4.85%
ABC	a = 42.82, b = 44.43, c = 21.93	R_{wp} = 2.69% R_p = 1.43%
$(AA_i{:}AB)^{stat}$	a = **44.82**, b = **44.43**, c = **21.99**	R_{wp} = **2.38%** R_p = **1.27%**

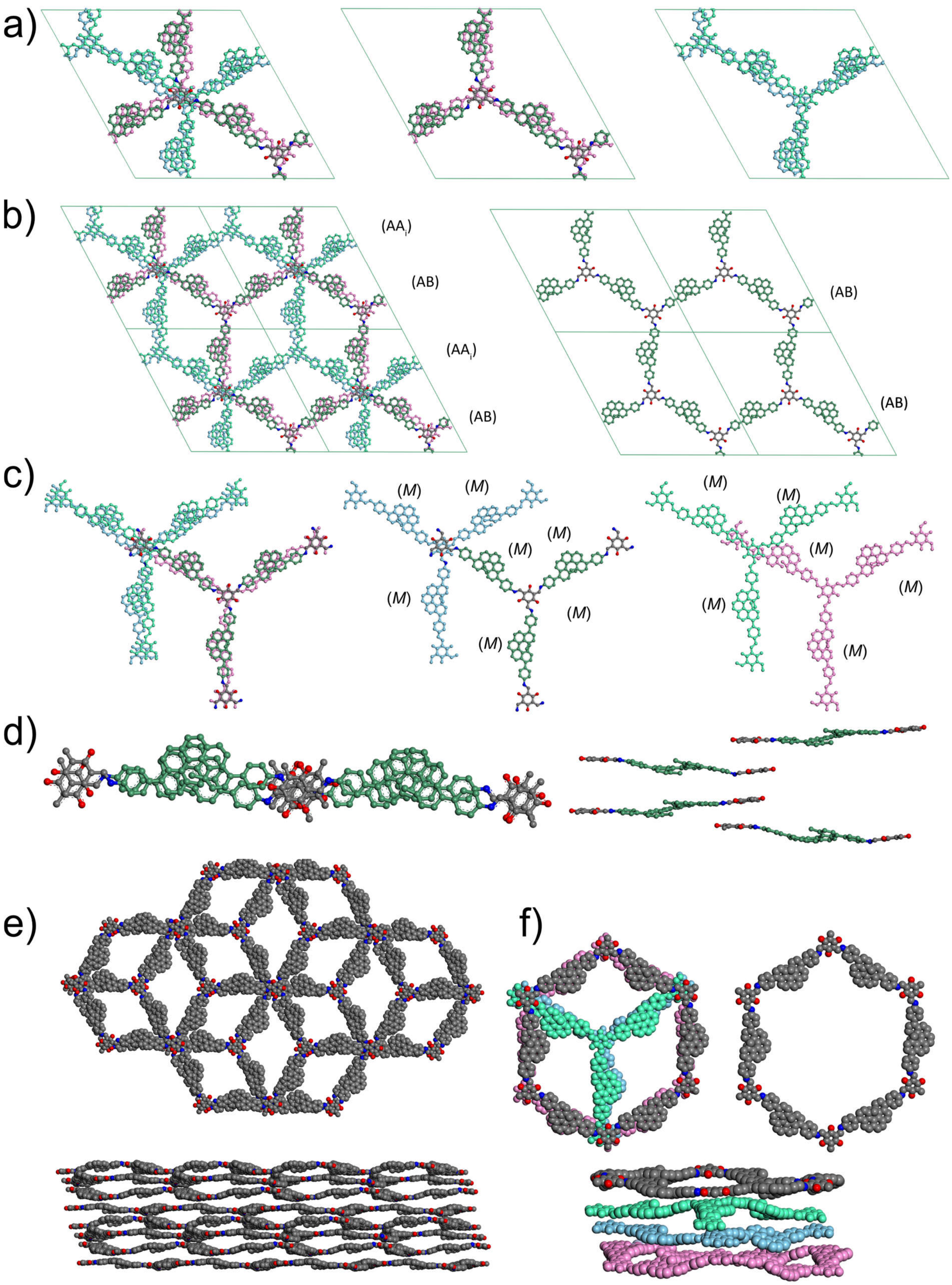

Figure S98. Pawley refinement structural model of (*M*)-**[5]Heli-TFP** COF with a combination of AA-inclined and AB-staggered stacking modes $(AA_i{:}AB)^{stat}$ (the unit cell is constructed with (*M*)-[5]helicene linkers, four lattices were contemplated and remarked in different colors for better visualization, hydrogen atoms are omitted for clarity); a) unit cell of (*M*)-**[5]Heli-TFP** COF optimized in Material Studio with four lattices; b) remark of the hexagonal topology with 4 layers and one single layer; c) shifted stacking mode of the (*M*)-[5]helicene linkers; e) top and side view of the corresponding hexagonal refined structure; f) single hexagonal pore extracted from the refined structure.

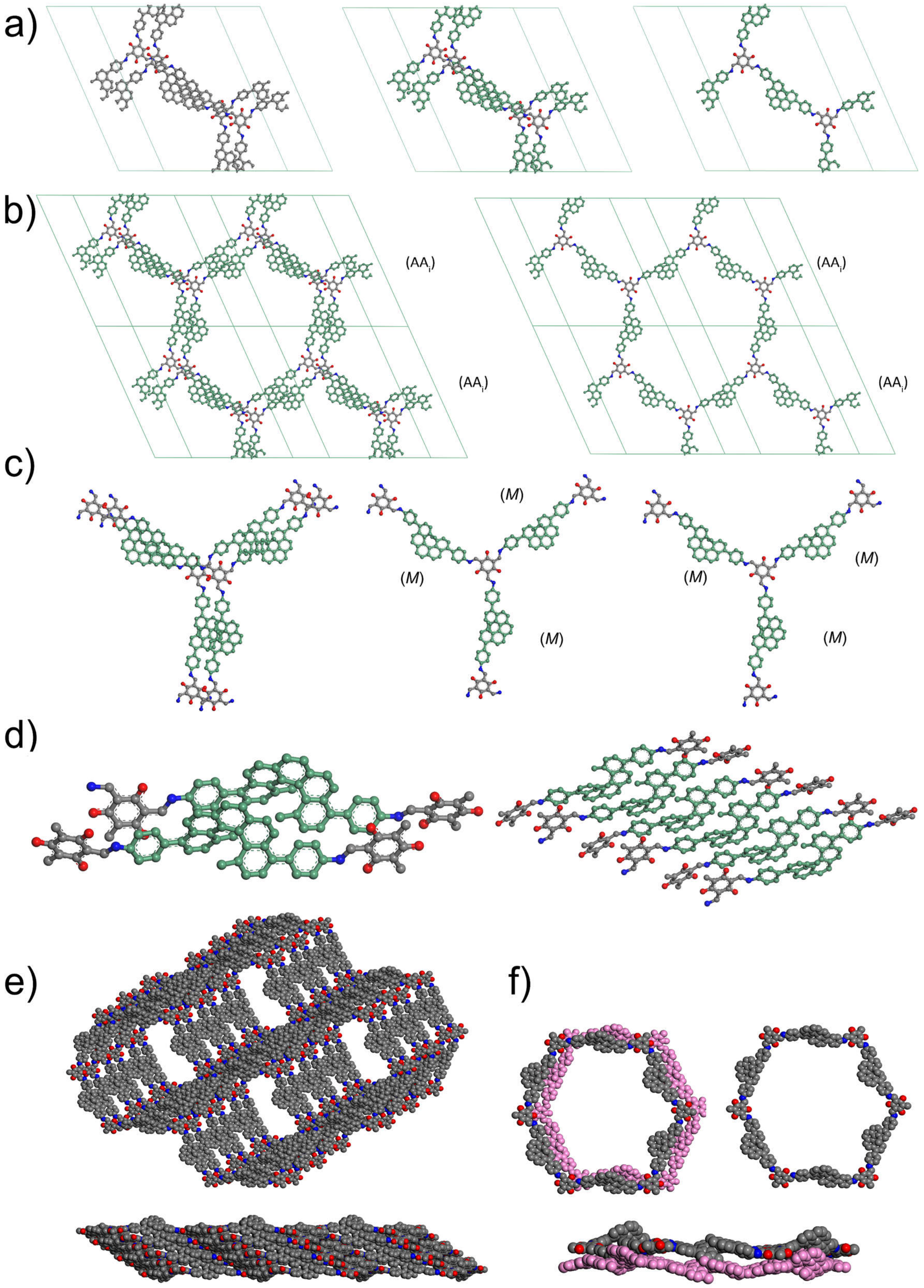

Figure S99. Pawley refinement structural model (*M*)-**[5]Heli-TFP** COF with AA-serrated stacking mode (the unit cell is constructed with (*M*)-[5]helicene linkers, two lattices were considered for the unit cell, hydrogen atoms are omitted for clarity); a) unit cell of (*M*)-**[5]Heli-TFP** COF optimized in Material Studio with four lattices; b) remark of the hexagonal topology with 4 layers and one single layer; c) shifted stacking mode of the (*M*)-[5]helicene linkers; e) top and side view of the corresponding hexagonal refined structure; f) Single hexagonal pore extracted from the refined structure.

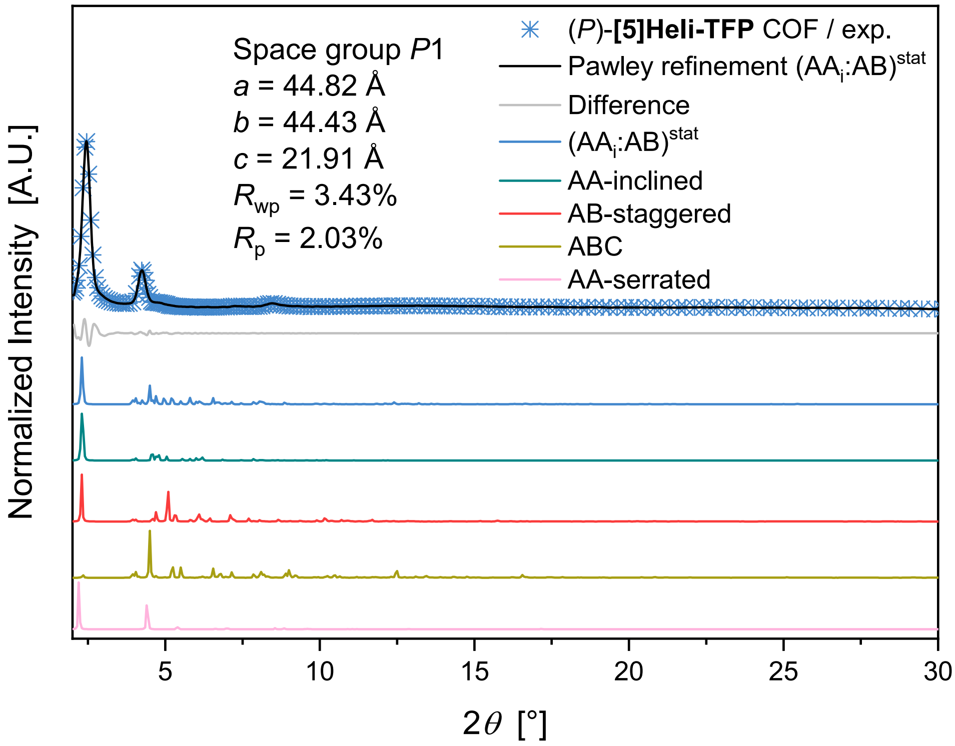

Figure S100. Experimental and simulated PXRD patterns from the different structural models of (*P*)-**[5]Heli-TFP** COF: experimental PXRD pattern (blue asterisk), Pawley refinement (black line) using an optimized structural model $(AA_i{:}AB)^{stat}$, difference between Pawley refinement and $(AA_i{:}AB)^{stat}$ stacking simulation (gray), simulated PXRD pattern from $(AA_i{:}AB)^{stat}$ stacking model (blue), simulated PXRD pattern from AA-inclined lattice (green), simulated PXRD pattern from AB-staggered lattice (red), simulated PXRD pattern from ABC lattice (yellow), simulated PXRD pattern from AA-serrated lattice (pink).

Table S13. Structural models of (*P*)-**[5]Heli-TFP** COF: calculated lattice parameters and corresponding agreement factors from the structural models with different stacking mode.

**Stacking model**	**Lattice parameters [Å]**	**Agreement factors (Pawley refined)**
AA-inclined	a = 44.82, b = 44.43, c = 11.01	R_{wp} = 4.97% R_p = 3.01%
AB-staggered	a = 44.82, b = 44.43, c = 17.87	R_{wp} = 5.07% R_p = 3.12%
AA-serrated	a = 42.34, b = 44.15, c = 12.15	R_{wp} = 17.24% R_p = 7.84%
ABC	a = 42.82, b = 44.43, c = 21.89	R_{wp} = 4.42% R_p = 2.20%
$(AA_i{:}AB)^{stat}$	a = **44.82**, b = **44.43**, c = **21.91**	R_{wp} = **3.43%** R_p = **2.03%**

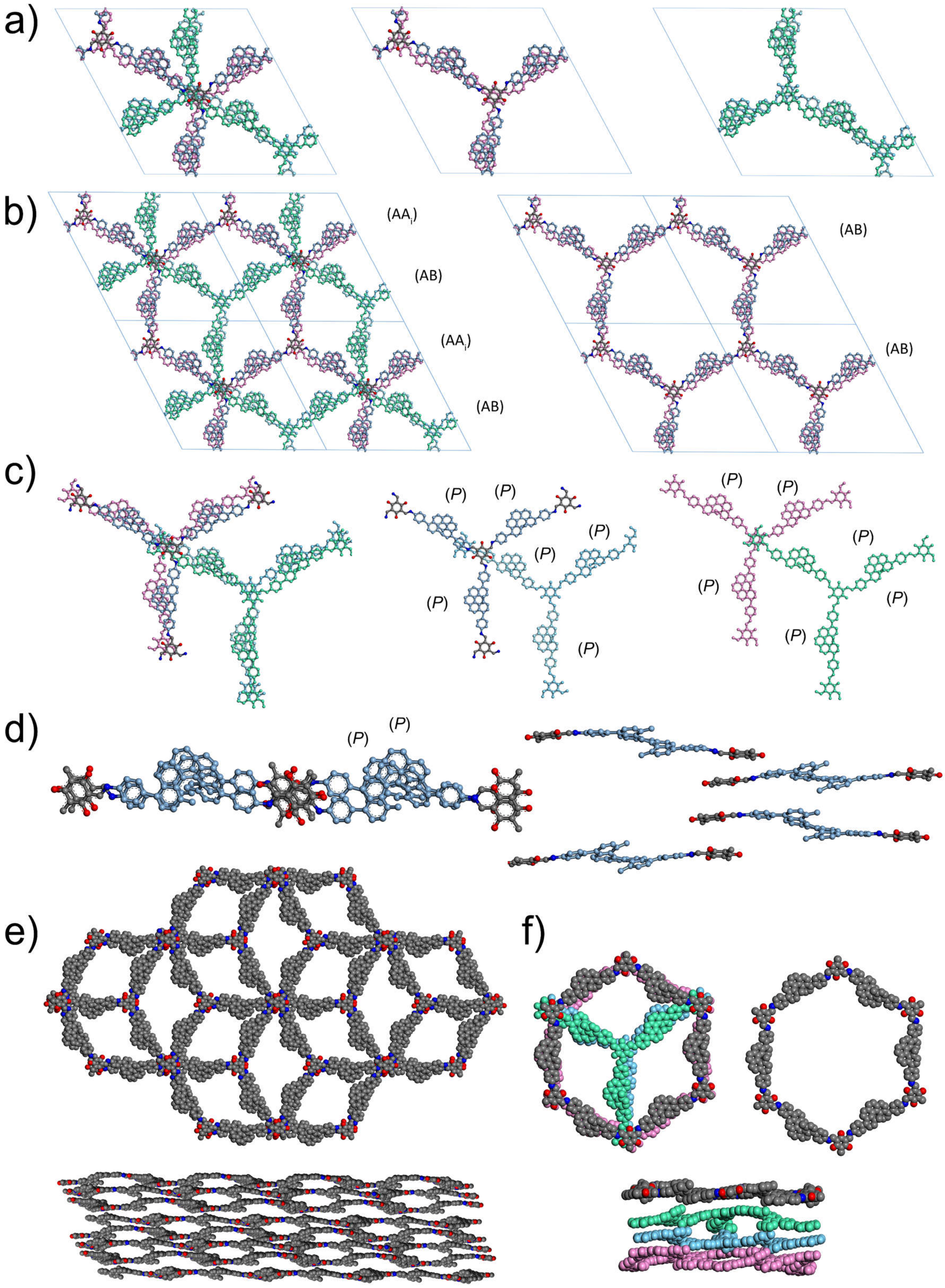

Figure S101. Pawley refinement structural model of (*P*)-**[5]Heli-TFP** COF with a combination of AA-inclined and AB-staggered stacking modes (AA_i:AB)^{stat} (the unit cell is constructed with (*P*)-[5]helicene linkers, four lattices were contemplated and remarked in different colors for better visualization, hydrogen atoms are omitted for clarity). a) Unit cell of (*P*)-**[5]Heli-TFP** COF optimized in Material Studio with four lattices. b) Remark of the hexagonal topology with 4 layers and one single layer. c) Shifted stacking mode of the (*P*)-[5]helicene linkers e) Top and side view of the corresponding hexagonal refined structure. f) Single hexagonal pore extracted from the refined structure.

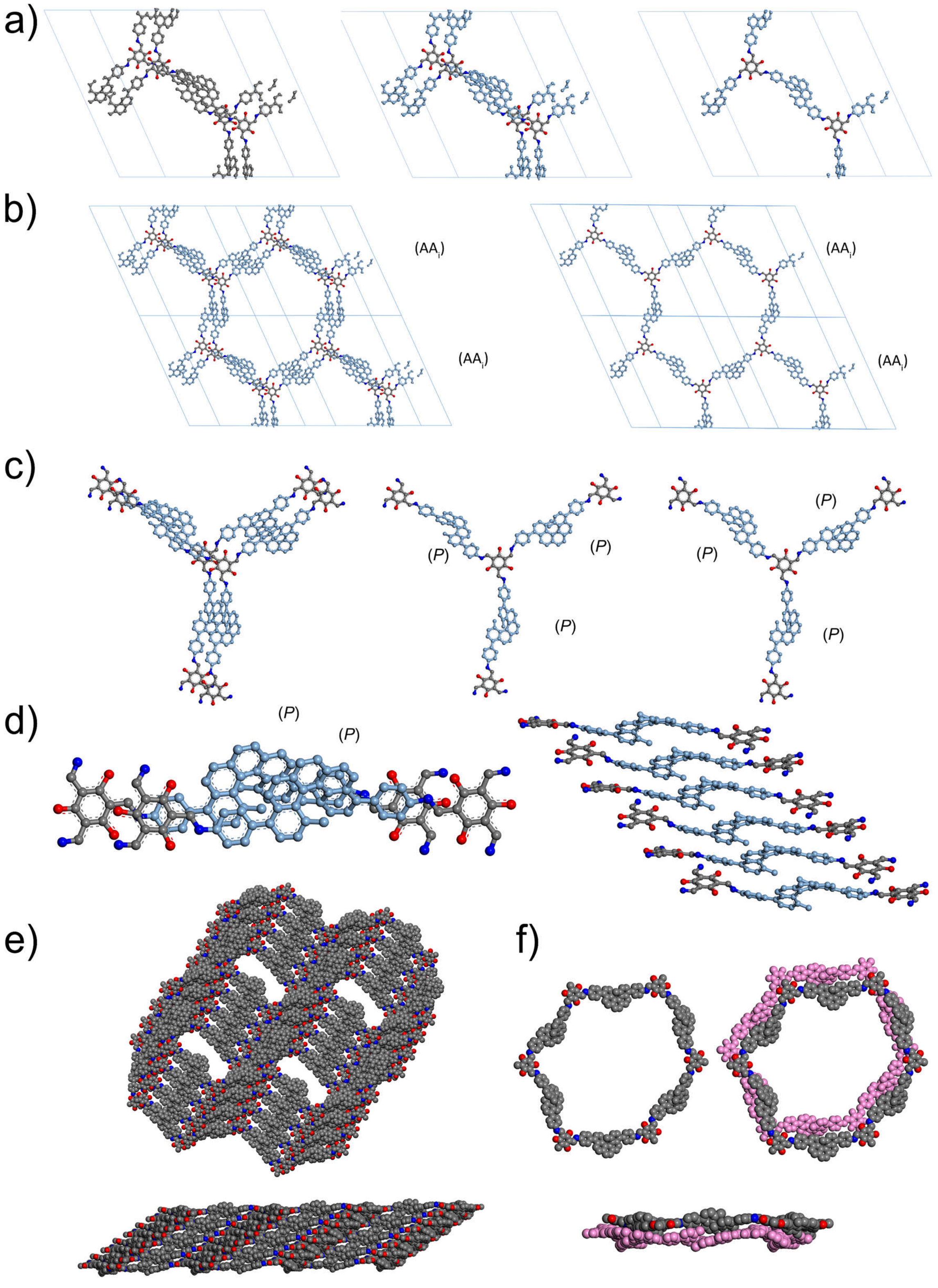

Figure S102. Pawley refinement structural model of (*P*)-**[5]Heli-TFP** COF with AA-serrated stacking mode (the unit cell is constructed with (*P*)-[5]helicene linkers, two lattices are contemplated, hydrogen atoms are omitted for clarity); a) unit cell of (*P*)-**[5]Heli-TFP** COF optimized in Material Studio with four lattices; b) remark of the hexagonal topology with 4 layers and one single layer; c) shifted stacking mode of the (*P*)-[5]helicene linkers; e) top and side view of the corresponding hexagonal refined structure; f) single hexagonal pore extracted from the refined structure.

S16. Diastereomer Separation

The separation of the diastereomers of (*R*,*R*)-(*P*/*M*)-**5** was achieved by preparative HPLC using chiral stationary phase ((*S*,*S*) Whelk-O®) with isocratic flow 15 mL min^{-1}, using *n*-hexane/*i*-PrOH 97/3 as mobile phase, 6 runs were done with stacked injections of 5 mL every 50 min of 2 mg mL^{-1} solution of (*R*,*R*)-(*P*/*M*)-**5** in *n*-hexane/*i*-PrOH 95/5, 35 mg of fraction A (FA), and 20 mg of fraction B (FB) were separated, and the successful diastereomer resolution was confirmed by reinjecting the collected fractions (Figure S103). The fraction A eluted at 32.0 min and corresponds to (*R*,*R*)-(*M*)-(–)-**5** (*ee* = 99.9%) (Figure S104), as confirmed by ECD and ORD spectroscopy, their theoretical simulation by TD-DFT calculations (Figure S109), and the absolute configuration derivate from the single-crystal X-ray structure of (*M*)-(–)-**1** (Figure S127). Hence, the fraction B eluted at 36.5 min and corresponds to (*R*,*R*)-(*P*)-(+)-**5** (*ee* = 99.6%, Figure S105). Repeated reinjections of fraction B in the HPLC system resulted in no further change on the *ee* of (*R*,*R*)-(*P*)-(+)-**5**.

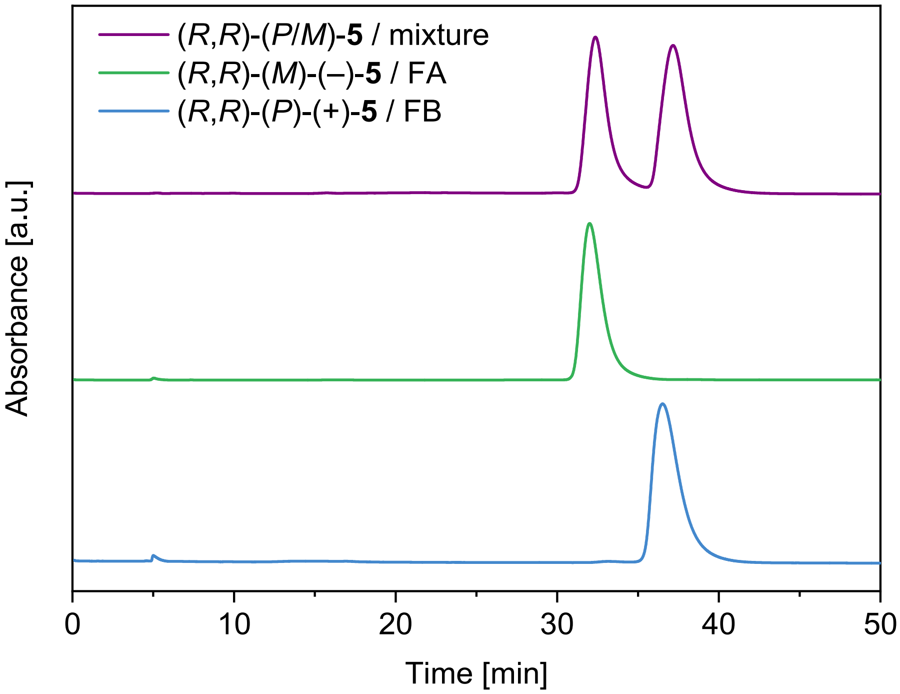

Figure S103. Chromatograms of diastereomers separation from (*R*,*R*)-(*P*/*M*)-**5** using *n*-hexane/*i*-PrOH 97/3, using ((*S*,*S*)-Whelk-O®) as a stationary phase with isocratic mode flow 15 mL min^{-1}.

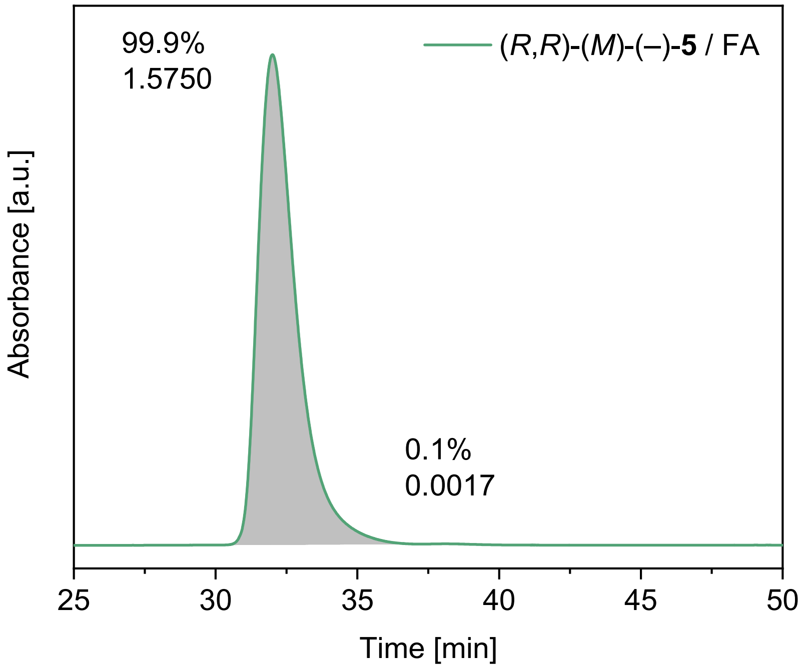

Figure S104. Chromatogram of (*R*,*R*)-(*M*)-(–)-**5** (FA, *ee* = 99.9%) using *n*-hexane/*i*-PrOH 97/3, using ((*S*,*S*)-Whelk-O®) as a stationary phase with isocratic mode flow 15 mL min^{-1}.

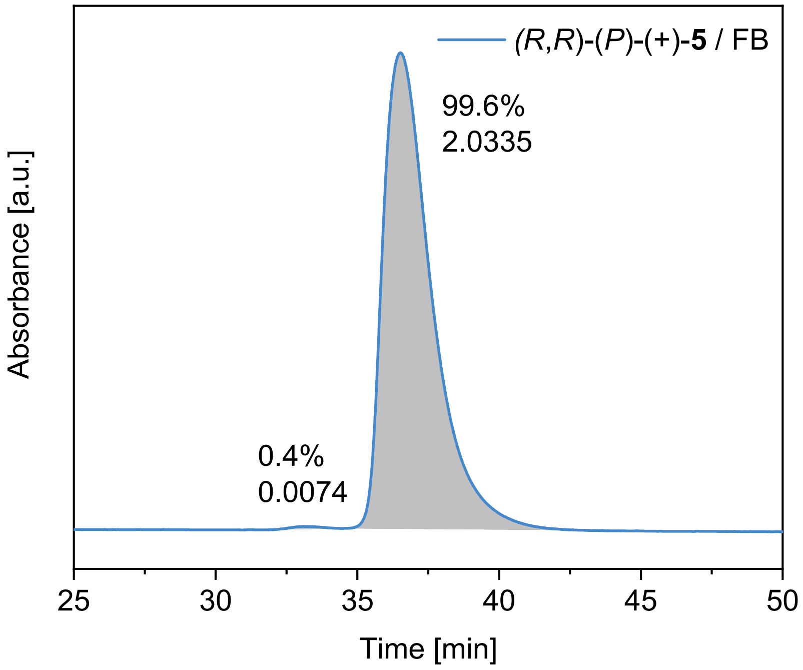

Figure S105. Chromatogram of (*R*,*R*)-(*P*)-(+)-**5** (FB, *ee* = 99.6%) using *n*-hexane/*i*-PrOH 97/3, using ((*S*,*S*)-Whelk-O®) as a stationary phase with isocratic mode flow 15 mL min^{-1}.

The separation of the diastereomers (*R*,*R*)-(*P*/*M*)-**7** was achieved by preparative high-performance chromatography using a chiral stationary phase (*S*,*S*) Whelk-O® 1 with isocratic flow of 15 mL min^{-1}, *n*-hexane/*i*-PrOH 97:3. We made 12 runs with stacked injections of 5 mL of 1 mg mL^{-1}. The successful diastereomer resolution was confirmed by reinjection the collected fractions, confirming the relative high purity of the first fraction to elute (fraction A, 33.6 mg) and the second fraction to elute (fraction B, 16.8 mg), no decomposition was observed during the separation of the diastereomers (Figure S106). The fraction A eluted around 18.0 min and correspond to the diastereomer (*R*,*R*)-(*M*)-(–)-**7** (*ee* = 99.8%, Figure S107). The fraction B eluted at 20.5 min and correspond to the diastereomer (*R*,*R*)-(*P*)-(+)-**7** (*ee* = 99.2%, Figure S108). As confirmed by ECD and ORD spectroscopy, and their theoretical simulation by TD-DFT calculations (Figure S112).

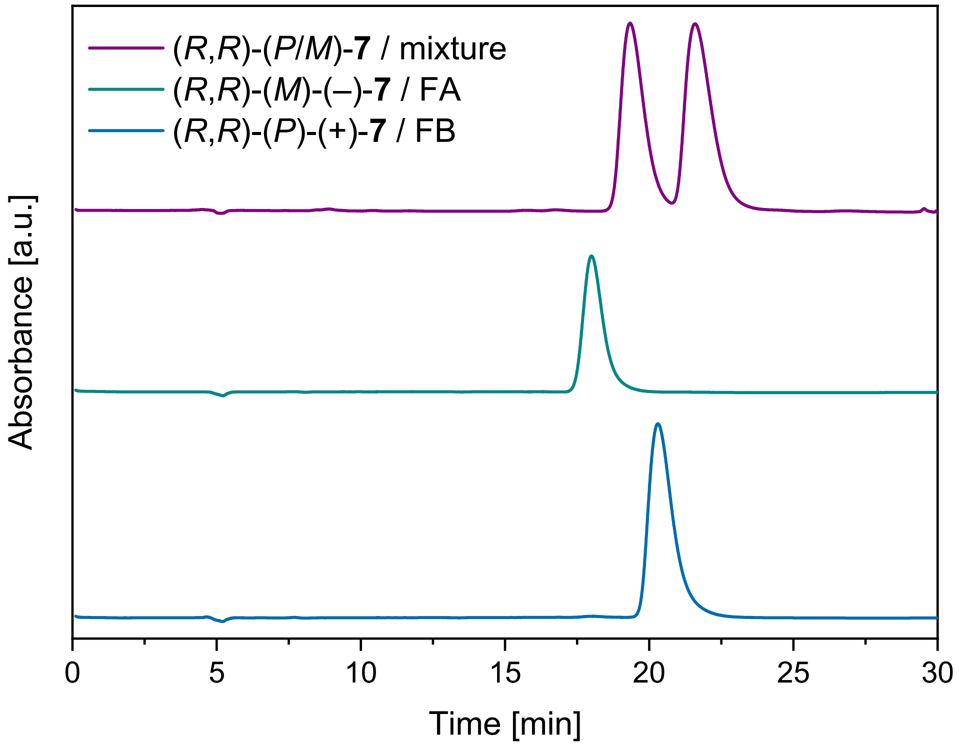

Figure S106. Chromatograms of the separation of diastereomers from (*R*,*R*)-(*P*/*M*)-**7** using *n*-hexane/*i*-PrOH 97/3 using (*S*,*S*) Whelk-O® as a stationary phase with isocratic mode flow 15 mL min $^{-1}$.

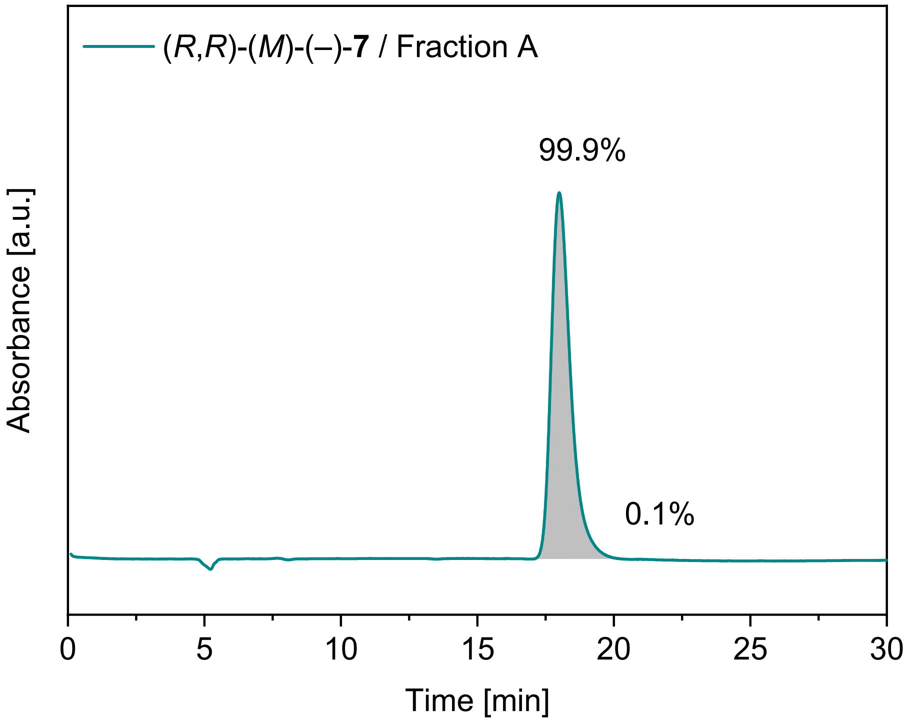

Figure S107. Chromatogram of (*R*,*R*)-(*M*)-(–)-**7** (FA, *ee* = 99.8%) with *n*-hexane/*i*-PrOH 97/3 as eluent with (*S*,*S*)-Whelk-O® as a stationary phase and isocratic mode flow 15 mL min $^{-1}$.

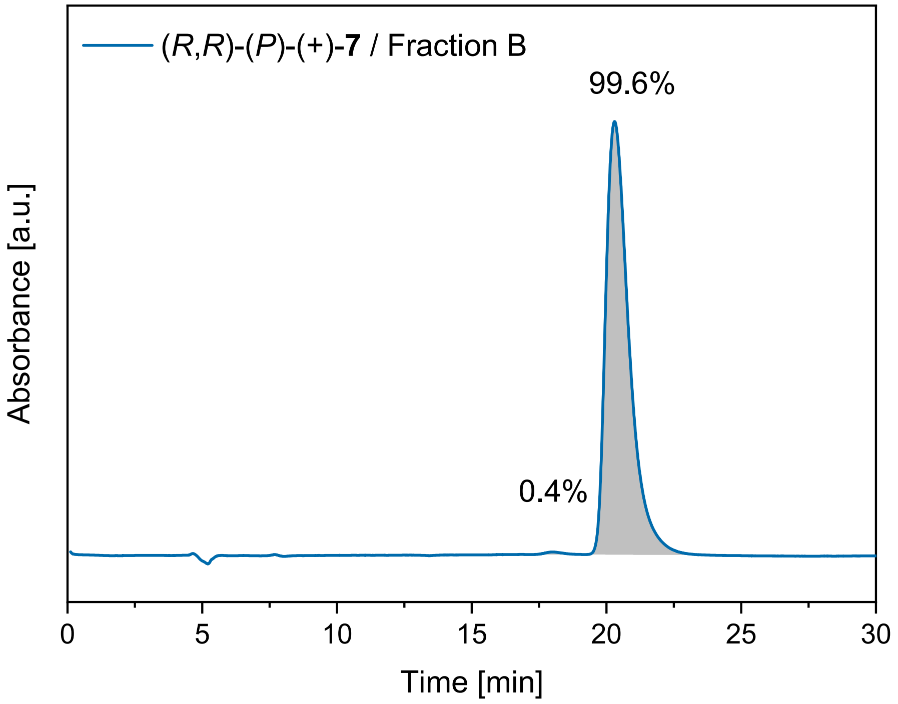

Figure S108. Chromatogram of (*R*,*R*)-(*P*)-(+)-**7** (FB, *ee* = 99.2%) using *n*-hexane/*i*-PrOH 97/3 as eluent with (*S*,*S*)-Whelk-O® as a stationary phase and isocratic mode flow 15 mL min $^{-1}$.

S17. Electronic Circular Dichroism spectroscopy

The absolute configuration analysis was derived from the single-crystal X-ray analysis of the enantiopure (*M*)-(–)-**1** (Figure S127), which was further validated by the comparison of the experimental electronic circular dichroism (ECD) spectra with the calculated ECD curves using time-dependent density functional theory (TD-DFT). Initially, the structure was optimized at DFT/CAM-B3LYP/6-31G(d) level of theory. Subsequently, the conformers (local and global minima) within a range of 3 kcal mol^{-1} above the global minimum were identified, and the ECD spectra were calculated for all the conformers at the level of theory CAM-B3LYP/def2-TZVP/CPCM(CH_2Cl_2). Then, the Boltzmann-weighted summation of spectra was simulated using the program Specdis,[24] and compared with the experimental ECD spectra. As the authors of *Specdis* indicate, that "*the UV shift is a pure empirical approach*",[24] for our calculated structures being ± 20 nm shift range within an energy range of 250–450 nm (band width 0.3) lead to identify the correct structure, supported by the single crystal X-ray structure of the pure enantiomer (*M*)-(–)-**1** (Figure S127). In our case, when applying the *Specdis* default analysis values of ± 30 nm for the UV shift within an energy range of 200–600 nm (band width 0.3), it was not possible to determine a specific configuration. We noticed that —despite many years of experience— in this case, assigning the absolute configuration using the TD-DFT approach for (*R*,*R*)-(*P*/*M*)-**5**, and especially for (*P*/*M*)-**3** could be flawed using this method. Hence, we rely on the single-crystal X-ray diffraction results.

Furthermore, we observed an uncommon inversion in the sign of the specific rotation and first Cotton effect in ECD for the enantiopure [5]helicenes (*P*)-(–)-**3** and (*M*)-(+)-**3** with regard of their helical chirality. Typically, (*M*)-carbohelicenes display a levorotatory optical rotation in conjunction with a first negative Cotton effect. In contrast, (*P*)-carbohelicenes usually display a dextrorotatory optical rotation with a first positive Cotton effect.[25] For example, their parent pure diastereomers (*R*,*R*)-(*M*)-(–)-**5** and (*R*,*R*)-(*P*)-(+)-**5** are in line with this empirical trend. However, the enantiopure carbo[5]helicene (*M*)-(+)-**3** displays a positive specific rotation with a first positive Cotton effect despite being (*M*)-configured. The opposite behaviour was observed in (*P*)-(–)-**3**. This uncommon inversion is most likely influenced by the presence of multiple conformers promoted by the benzophenone imine moiety (as seen in their corresponding ^{1}H NMR spectra, Figure S31).

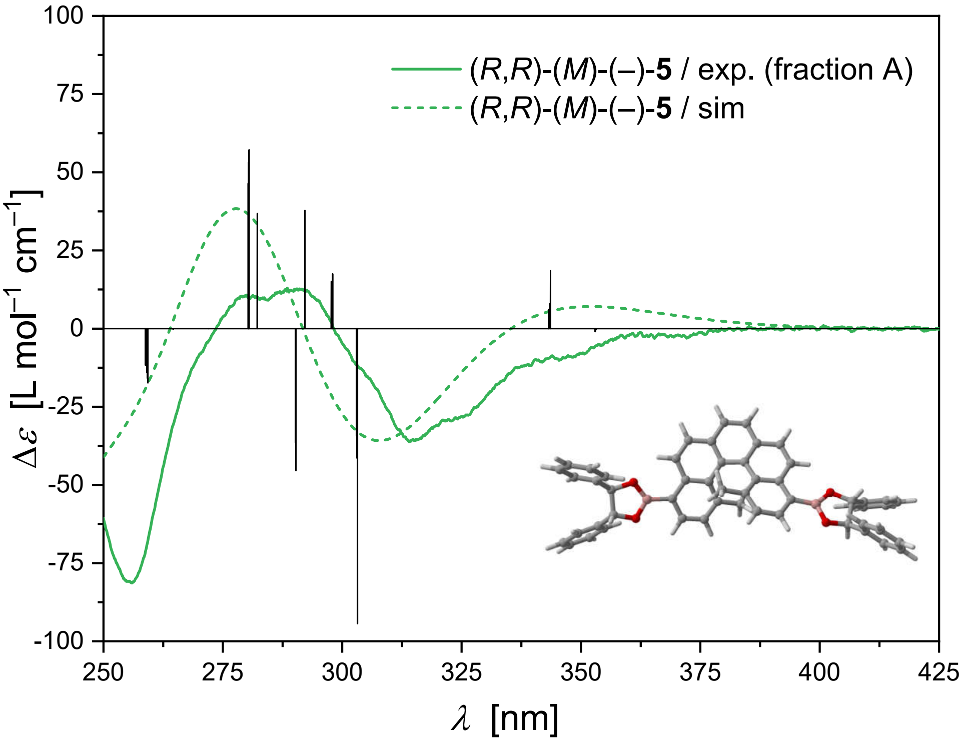

Figure S109. Comparison of the experimental ECD spectrum (*R*,*R*)-(*M*)-(–)-**5** (from fraction A) after the diastereomer separation (c = 1.3×10^{-5} M) CH_2Cl_2 at 298 K (solid blue line), the simulated spectra (dotted graphs), and discrete vertical transitions (black vertical) at the level of theory: TD-DFT-D3(BJ):CAM-B3LYP/def2-TZVP/CPCM(CH_2Cl_2) with a similarity factor = 0.96.

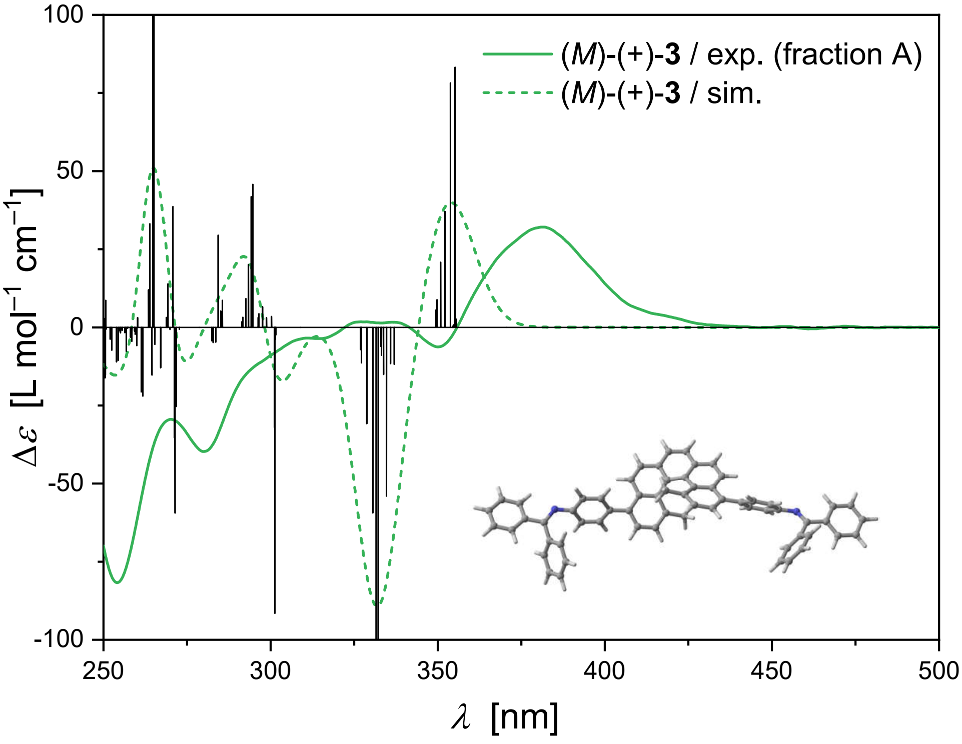

Figure S110. Comparison of the experimental ECD spectrum (*M*)-(+)-**3** (from fraction FA) after the diastereomer separation (c = 1.1x10^{-6} M) CH_2Cl_2 at 298 K (solid green line), the simulated spectra (dotted graphs), and discrete vertical transitions (black vertical) at the level of theory: TD-DFT-D3(BJ)/CAM-B3LYP/def2-TZVP/CPCM(CH_2Cl_2) with a similarity factor = 0.52.

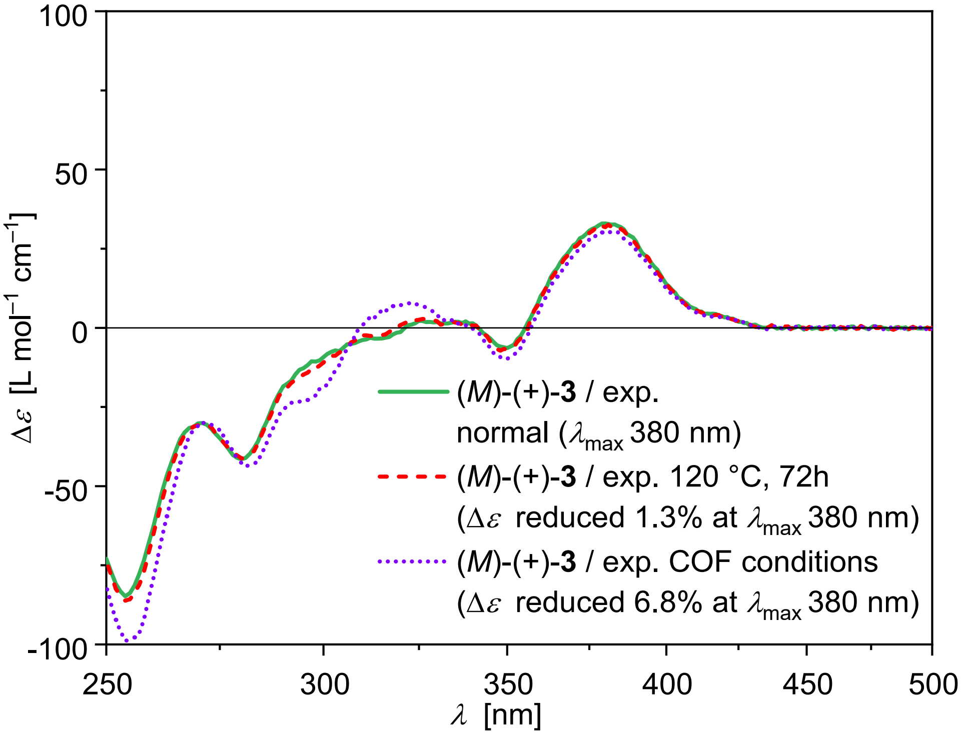

Figure S111. ECD spectra comparison of enantiopure building block (*M*)-(+)-**3** (c = 1.1x10^{-6} M) CH_2Cl_2 at 298 K (solid green line), following the intensity variation of Cotton effect at 380 nm after high-temperature treatment of (*M*)-(+)-**3** (120 °C, 72 h, red-dots), and solvothermal acid conditions used for COF synthesis treatment of (*M*)-(+)-**3** (40 equiv. of AcOH 6 M, 120 °C, 72 h, red-dots). Revealing that the Cotton effect intensity variation at 380 nm is less than 10%.

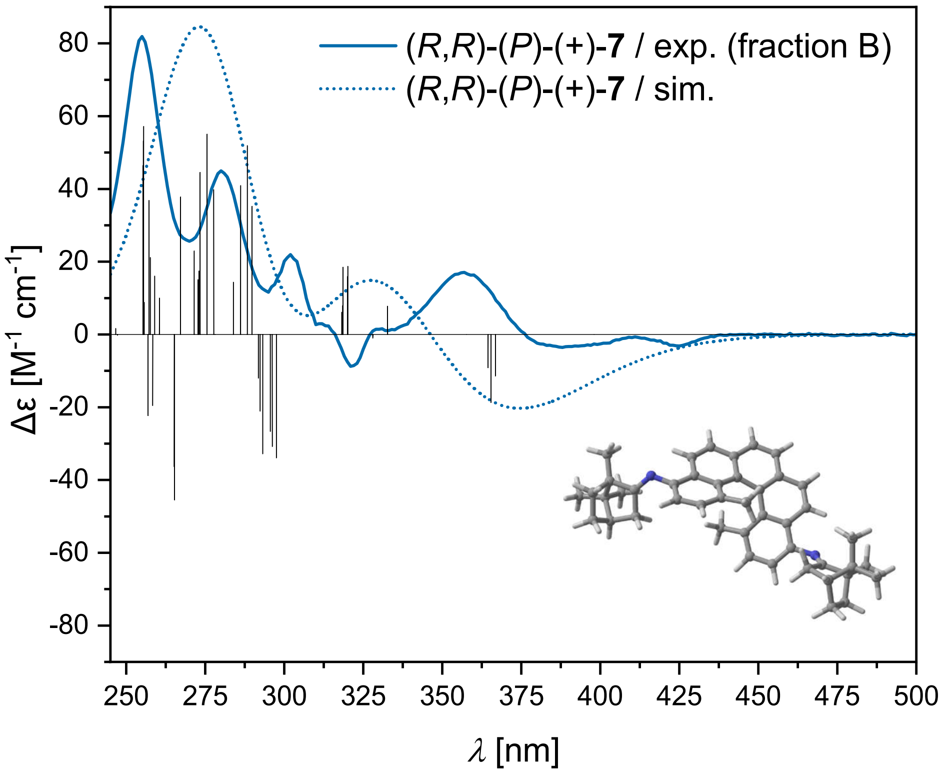

Figure S112. Comparison of the experimental and simulated ECD spectrum (*R*,*R*)-(*P*)-(+)-**7** (c = 6.0×10^{-5} M) CH_2Cl_2 at 298 K (solid blue line), the simulated spectra (dotted graphs), and discrete vertical transitions (black vertical) at the level of theory: TD-DFT-D3(BJ)/B3LYP/6-31G(d) /CPCM(CH_2Cl_2) with a similarity factor = 0.73.

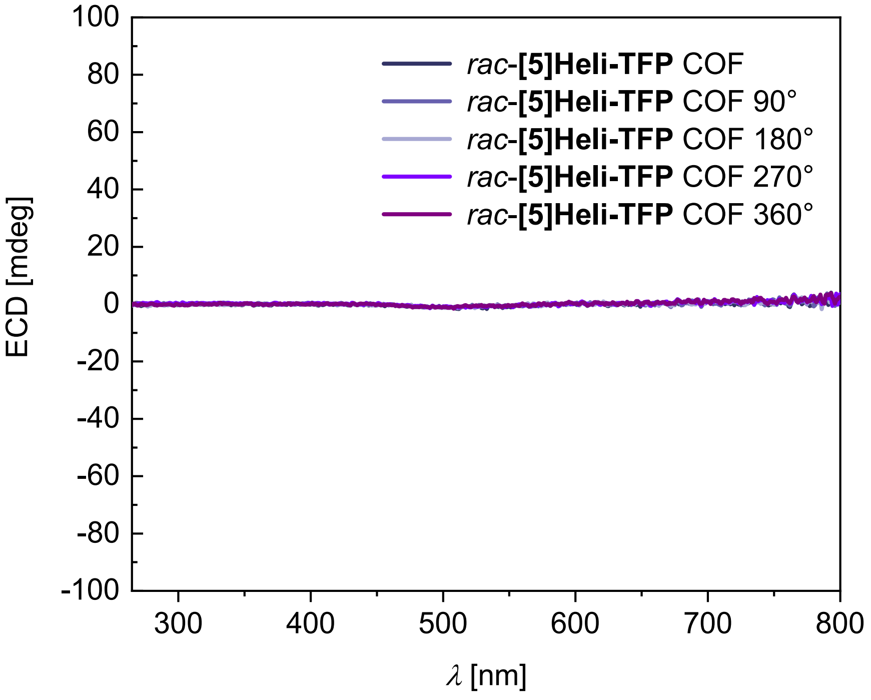

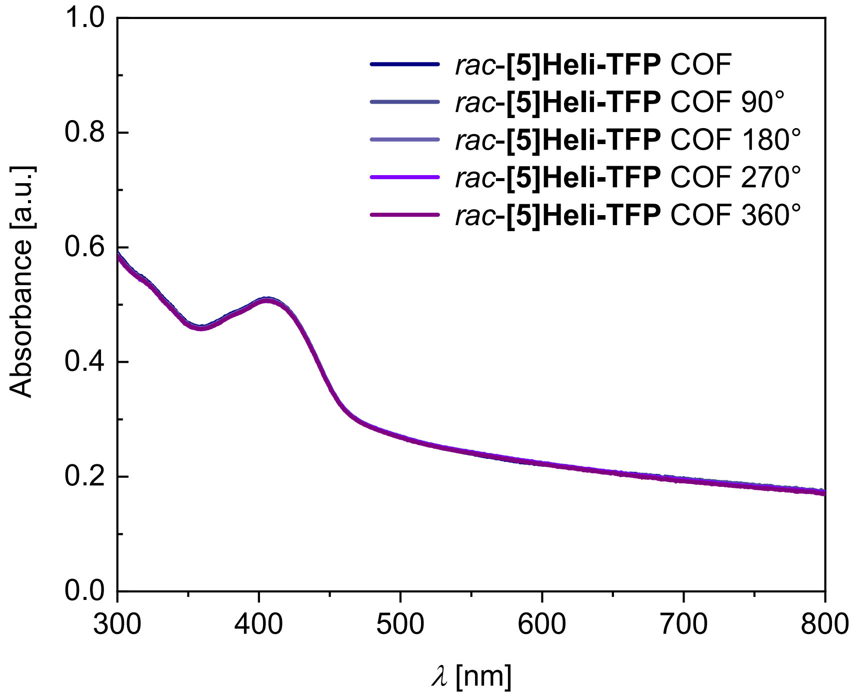

Figure S113. (top) ECD spectra of *rac*-**[5]Heli-TFP** COF ($c \approx 0.08$ mg mL^{-1}) in DMSO suspension at 298 K with different rotation angles using a 10 mm cuvette; (bottom) corresponding UV-VIS spectra of the same solutions (logarithmically scaled on the Y-axis).

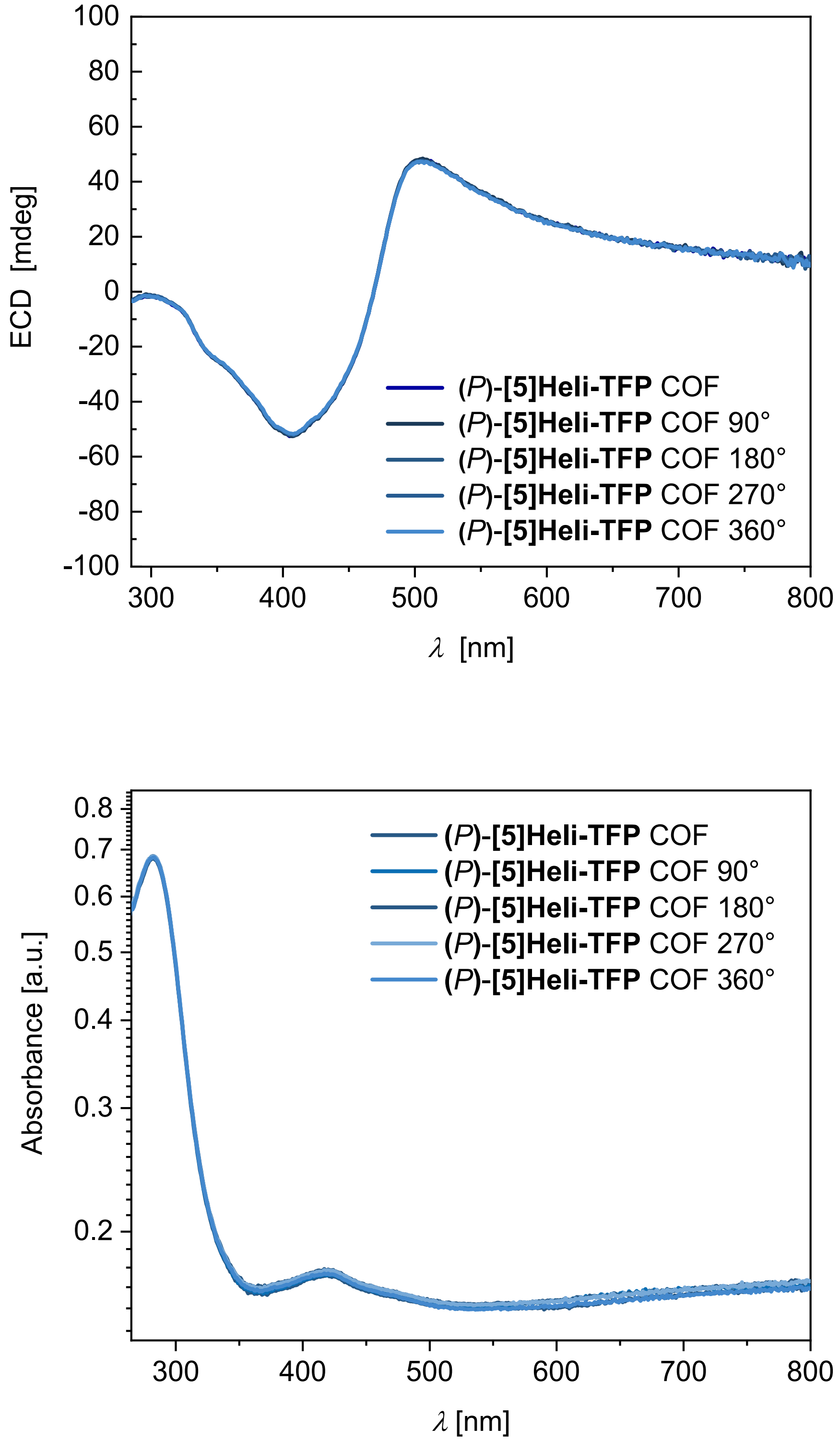

Figure S114. (top) ECD spectra of (*P*)-**[5]Heli-TFP** COF ($c \approx 0.08$ mg mL^{-1}) in DMSO suspension at 298 K with different rotation angles using a 10 mm cuvette; (bottom) corresponding UV-VIS spectra of the same solutions (logarithmically scaled on the Y-axis).

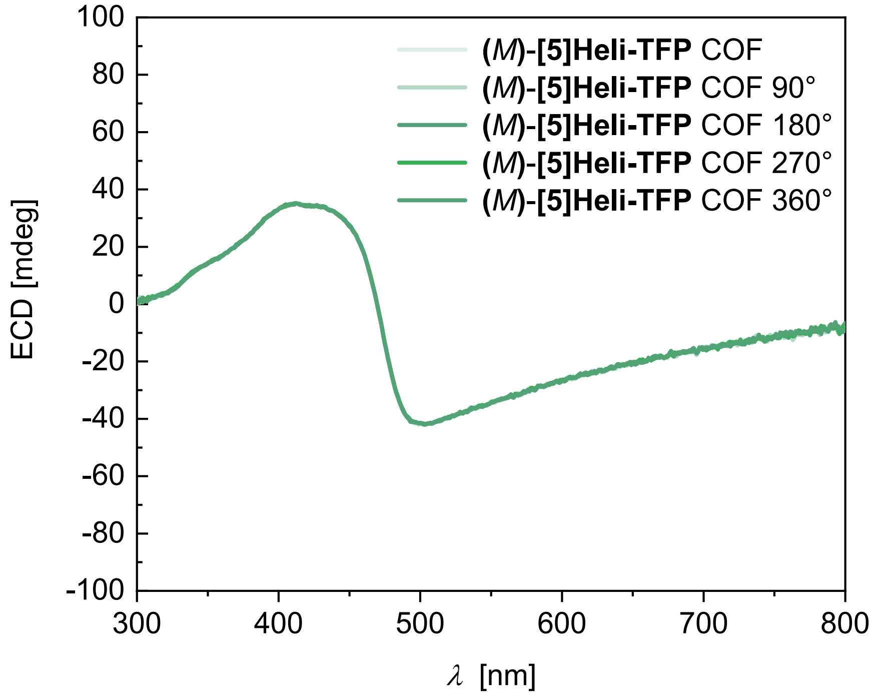

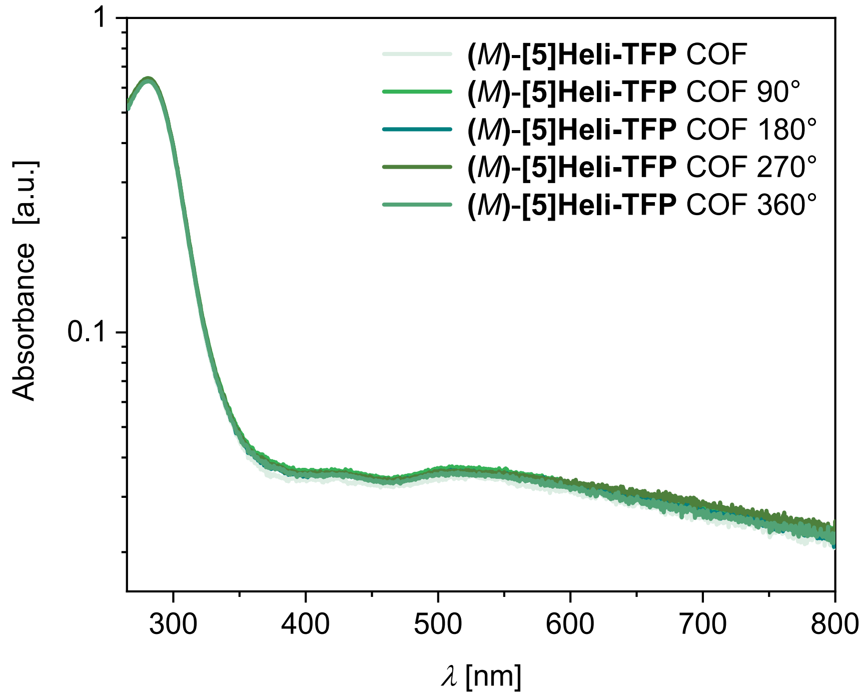

Figure S115. (top) ECD spectra of (*M*)-**[5]Heli-TFP** COF ($c \approx 0.08$ mg mL^{-1}) in DMSO suspension at 298 K with different rotation angles using a 10 mm cuvette; (bottom) corresponding UV-VIS spectra of the same solutions (logarithmically scaled on the Y-axis).

The corresponding dissymmetry factor (g_{abs} factor) was calculated using the equation (S1),[26] which has been applied for 2D chiral perovskites and 2D COFs.[26,27] From the corresponding **[5]Heli-TFP** COFs in DMSO dispersions (Figure S113–115), (*P*)-**[5]Heli-TFP** COF exhibited a $|g_{abs}|$ factor of 9.2×10^{-3} at λ = 507 nm, (Figure S116). In contrast, (*M*)-**[5]Heli-TFP** COF exhibited a $|g_{abs}|$ factor of $3.5\text{x}10^{-2}$ at λ = 504 nm, approximately three times higher than the opposite homochiral analogue. This difference can be attributed to the diastereomeric excess analysis observed for the parent building blocks (see section S16). Specifically, the pure diastereomer (*R*,*R*)-(*P*)-(+)-**5** used for the synthesis of the corresponding homochiral (*P*)-**[5]Heli-TFP** COF contained four times the amount of the opposite enantiomer as an impurity (0.4% based on ee% Figure S105), compared to the highly purified parent building block (*R*,*R*)-(*M*)-(–)-**5** used for the synthesis of (*M*)-**[5]Heli-TFP** COF (0.1% based on ee%, Figure S104). Furthermore, these measurements were conducted on sonicated exfoliated samples with analytical comparable quantities, the observed deviations between the homochiral COF pairs can also be attributed to variations in colloidal behavior, in combination with the difference in enantiomeric purity based on the ee% of the used building blocks.

$$g_{abs} = \frac{CD\ [mdeg]}{32980 \times Absorbance\ [a.u.]} \quad \text{eq. (S1)}$$

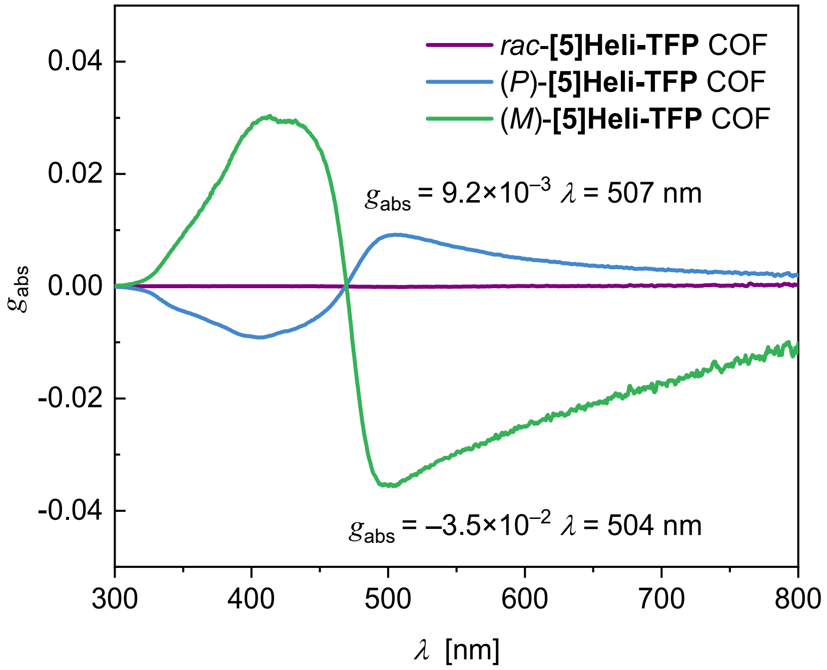

Figure S116. Calculated wavelength-dependent anisotropy factor (g_{abs}) of **[5]Heli-TFP** COFs based on the figures S70-72 and equation 1.

To the best of our knowledge, our homochiral **[5]Heli-TFP** COFs exhibit the highest g_{abs} for 2D CCOFs reported to date (see Table S14). This is the result of the appropriate incorporation of helical chirality into the backbone of two-dimensional extended crystalline structures

Table S14. Comparison of the reported dissymmetry factors of 2D CCOFs (g_{abs}).

2D CCOF	$\lvert g_{abs}\rvert$	λ [nm]	Aspect	Year	Reference
[a](*R*- and *S*-NEA)PbI_3	4.0×10^{-2}	395	film	2020	26
^{R}COF	2.0×10^{-2}	354	solid	2021	28
CityU-8	4.5×10^{-4}	550	solid	2023	27
TpPa(Λ)-COF	9.5×10^{-4}	550	film	2024	29
(*P*)-**[5]Heli-TFP** COF	9.2×10^{-3}	507	solid	***this work***	
(*M*)-**[5]Heli-TFP** COF	3.5×10^{-2}	504	solid	***this work***	

[a](*R*)- or ((*S*)-NEA)PbI_3 is a 2D chiral perovskite, used as reference for intense chiral 2D reticular material.[26]

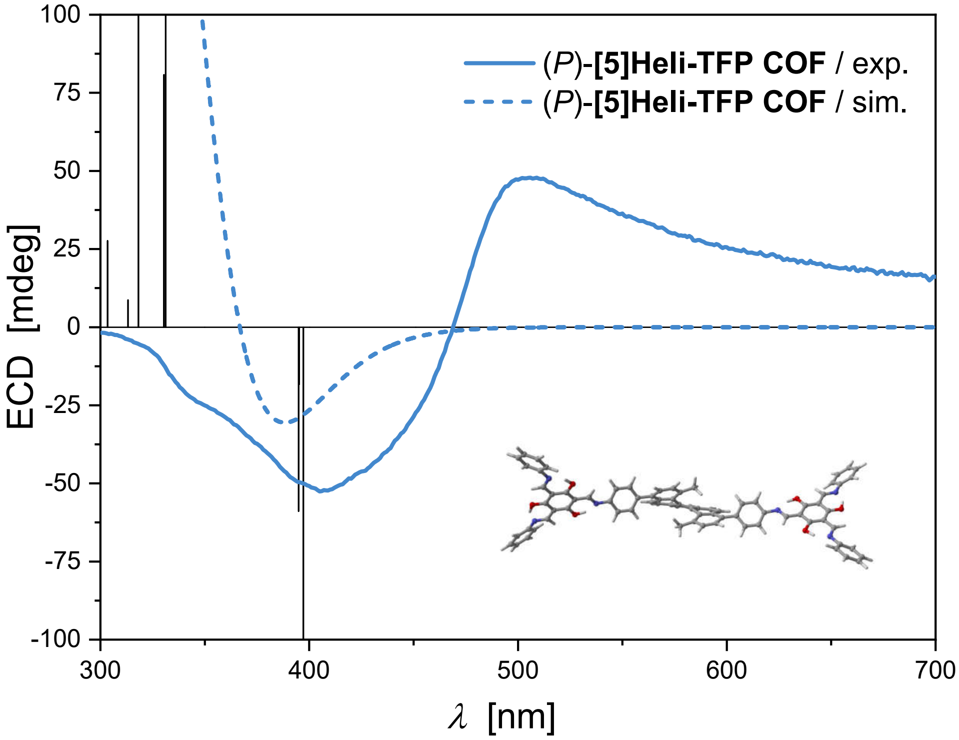

Figure S117. Comparison of the experimental ECD spectrum of a fragment from (*P*)-**[5]Heli-TFP** COF ($c \approx 0.08$ mg mL^{-1}) in DMSO (blue solid line), the simulated spectra (dotted line, UV shift 30 nm, band width 0.3), and discrete vertical transitions (black vertical) at the level of theory: TD-DFT-D3(BJ)/CAM-B3LYP/def2-TZVP/CPCM(DMSO) with a similarity factor = 0.53.

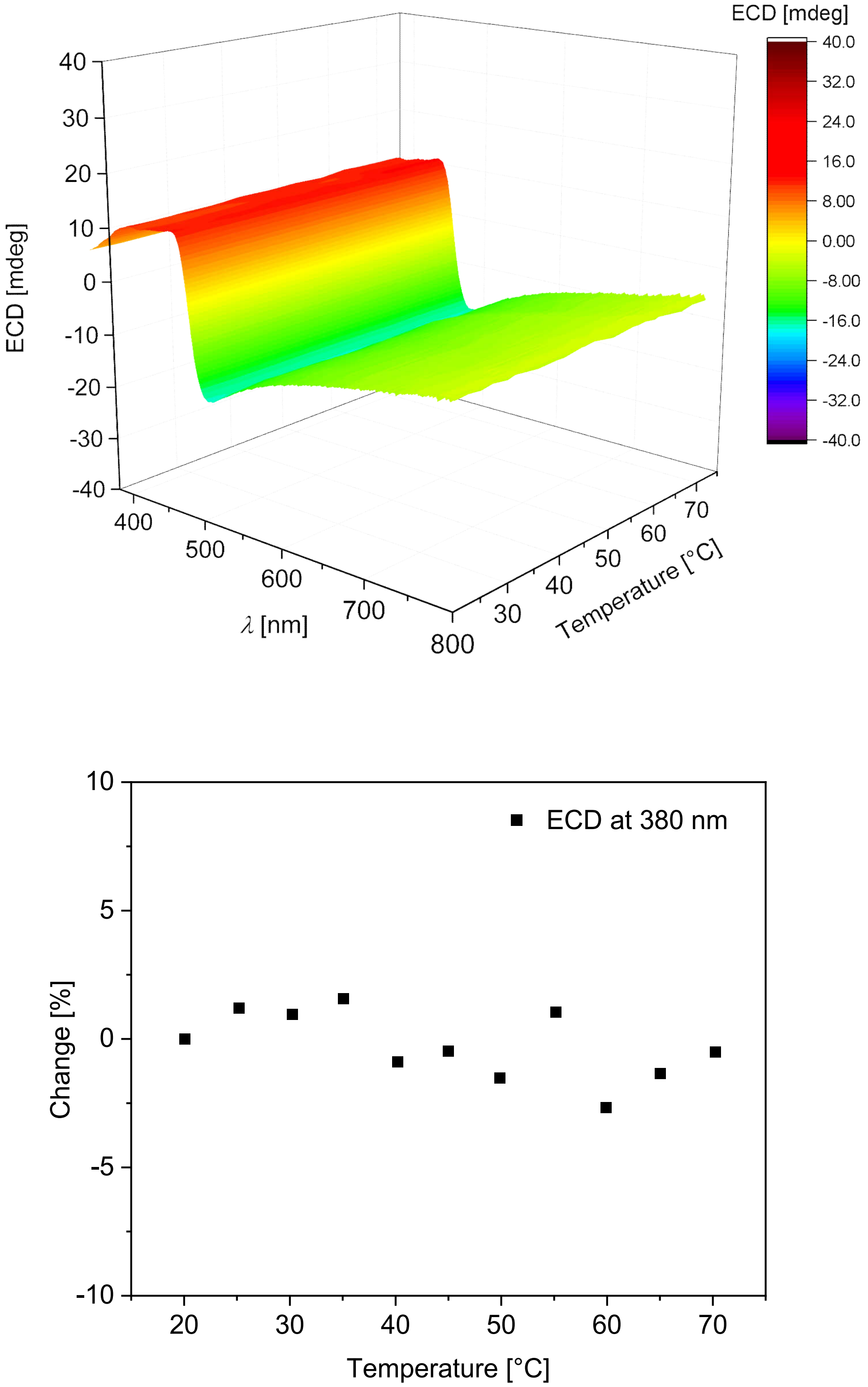

Figure S118. (Top) VT-ECD spectra of (*M*)-**[5]Heli-TFP** COF ($c \approx 0.05$ mg· mL^{-1}) in DMSO using 1 mm cuvette from 20 °C to 70 °C; (bottom) variation intensity of ECD at 380 nm at different temperatures, showing < 5% of intensity variation.

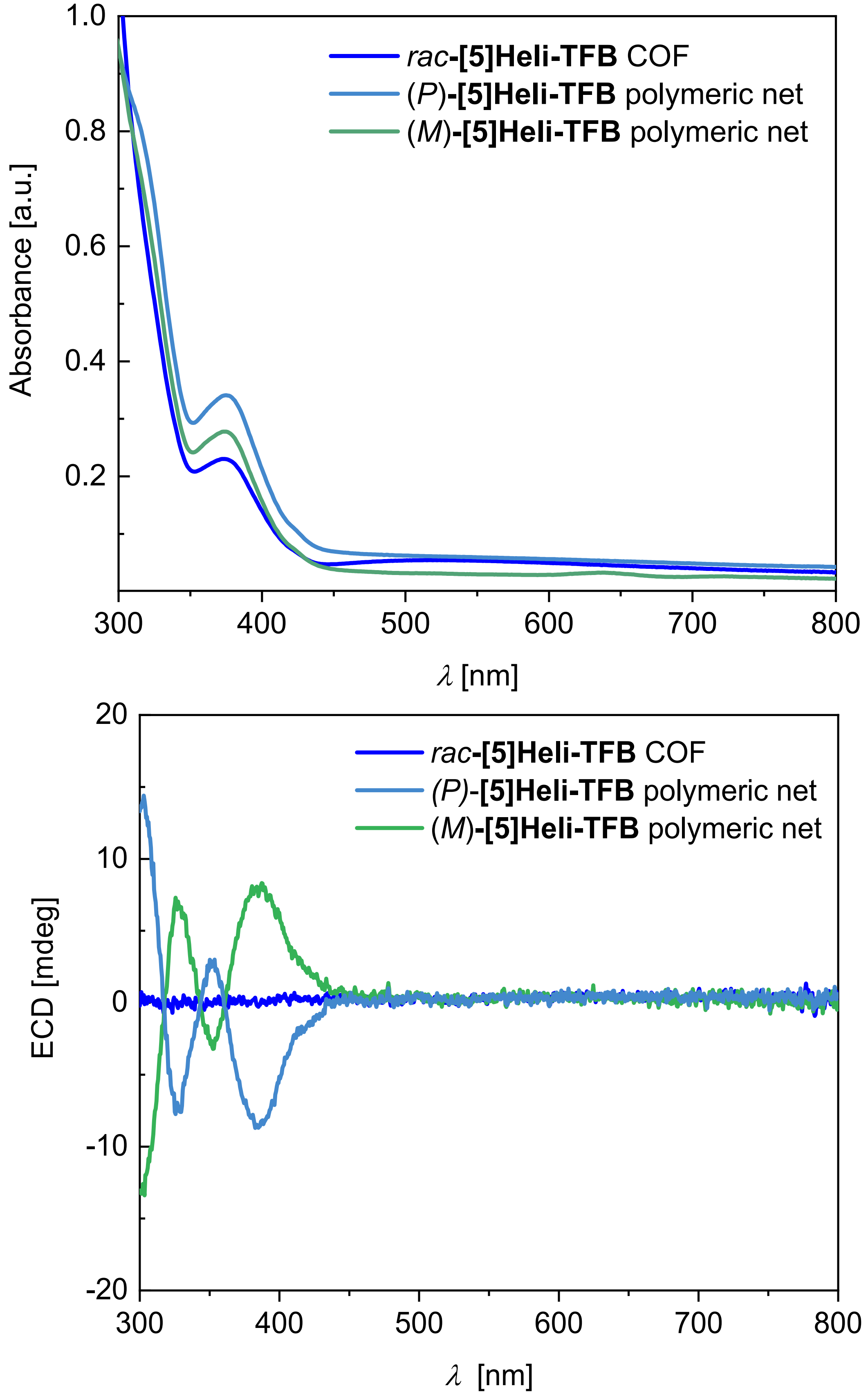

Figure S119. Comparison of optoelectronic properties of racemic and homochiral **[5]Heli-TFB** COF and polymeric nets; (a) average ECD spectra of *rac*-**[5]Heli-TFB** COF, (*P*)-**[5]Heli-TFB** polymeric net, and (*M*)-**[5]Heli-TFP** polymeric net ($c \approx 0.16$ g L^{-1}) in DMSO suspension using a 10 mm cuvette; (b) corresponding average UV-Vis spectra of the same solutions.

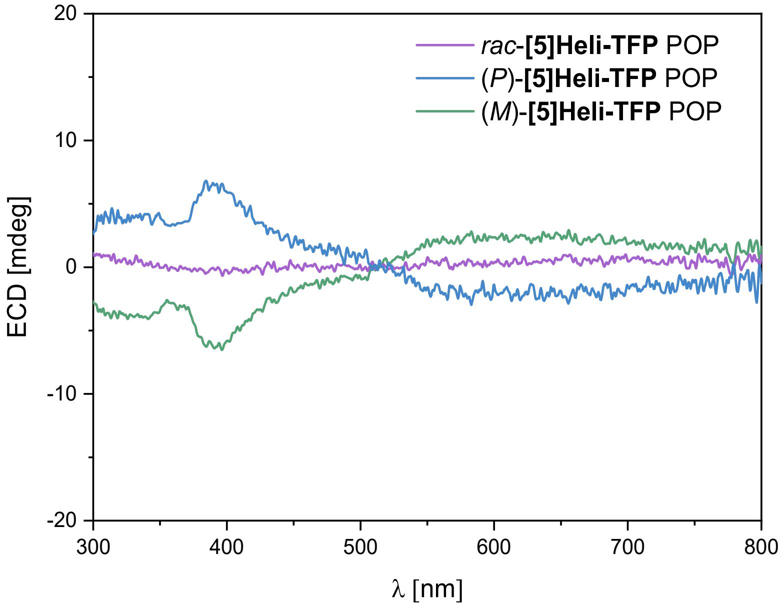

Figure S120. ECD spectra comparison of racemic and homochiral of **[5]Heli-TFP** POPs; (left) normalized absorption spectra of *rac*-**[5]Heli-TFP** POP (purple), (*P*)-**[5]Heli-TFP** POP (blue), (*M*)-**[5]Heli-TFP** POP (green) ($c \approx 0.08$ mg mL^{-1}) in DMSO at 298 K.

For the collection of the ECD spectra the **[5]Heli-TFP COF-2** films, the films were placed between rectangular quartz layers and positioned in front of the incited light as showed below. We observed a silent ECD signal when using clean quartz-substrates, as show in Figure S121.

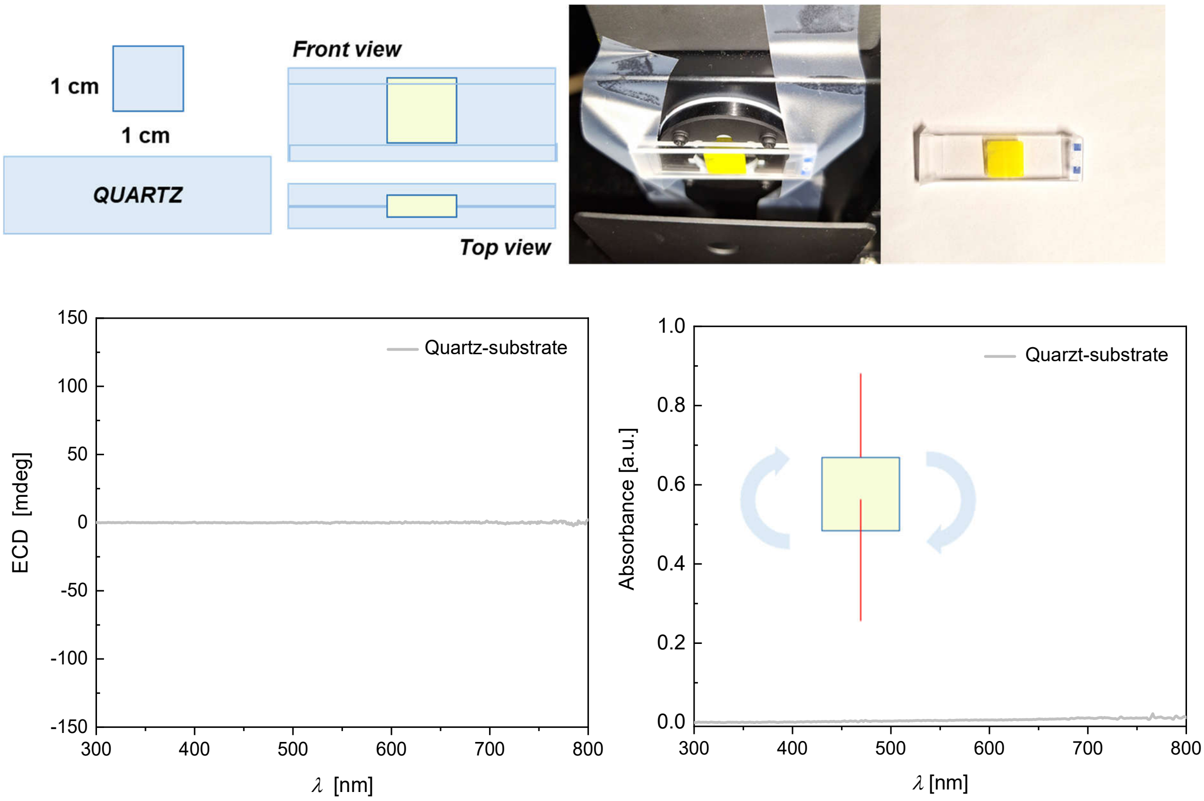

Figure S121. (top) Representation of the set-up used for ECD and UV-Vis measurements of the COF-films on quartz substrates, enabling the rotation and flipping of the films without directly touching its surface; (bottom) ECD spectrum and UV-Vis spectra at 298 K.

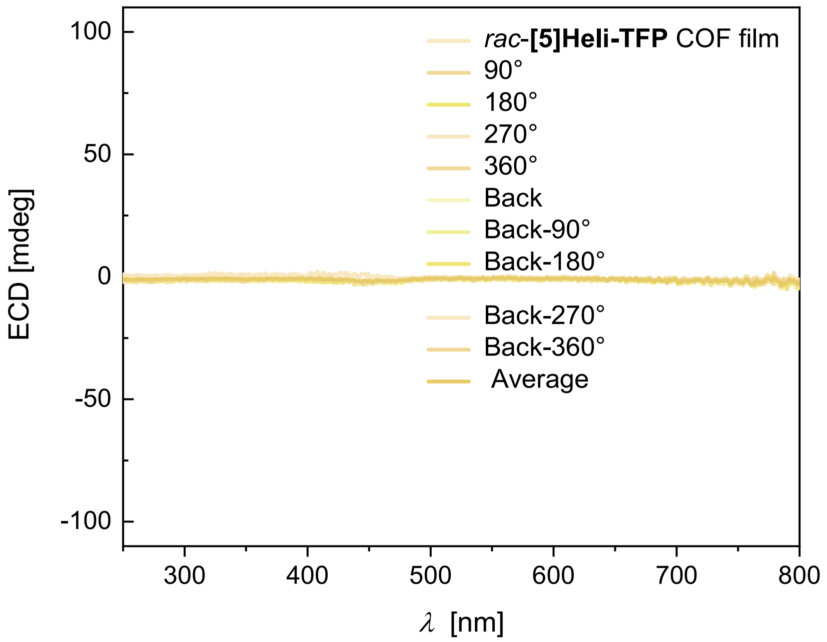

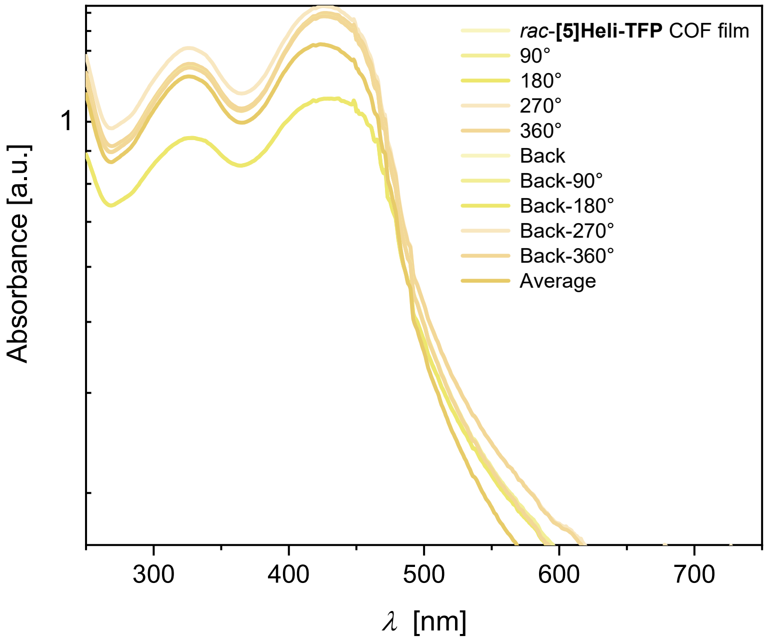

Figure S122. Characterization of *rac*-**[5]Heli-TFP** COF film in quartz-substrate with concentration of 1.2 mM of (±)-**3**; (top) ECD spectra with different rotation angles; (bottom) corresponding UV-Vis spectra with different rotation angles at 298 K (logarithmically scaled on the Y-axis).

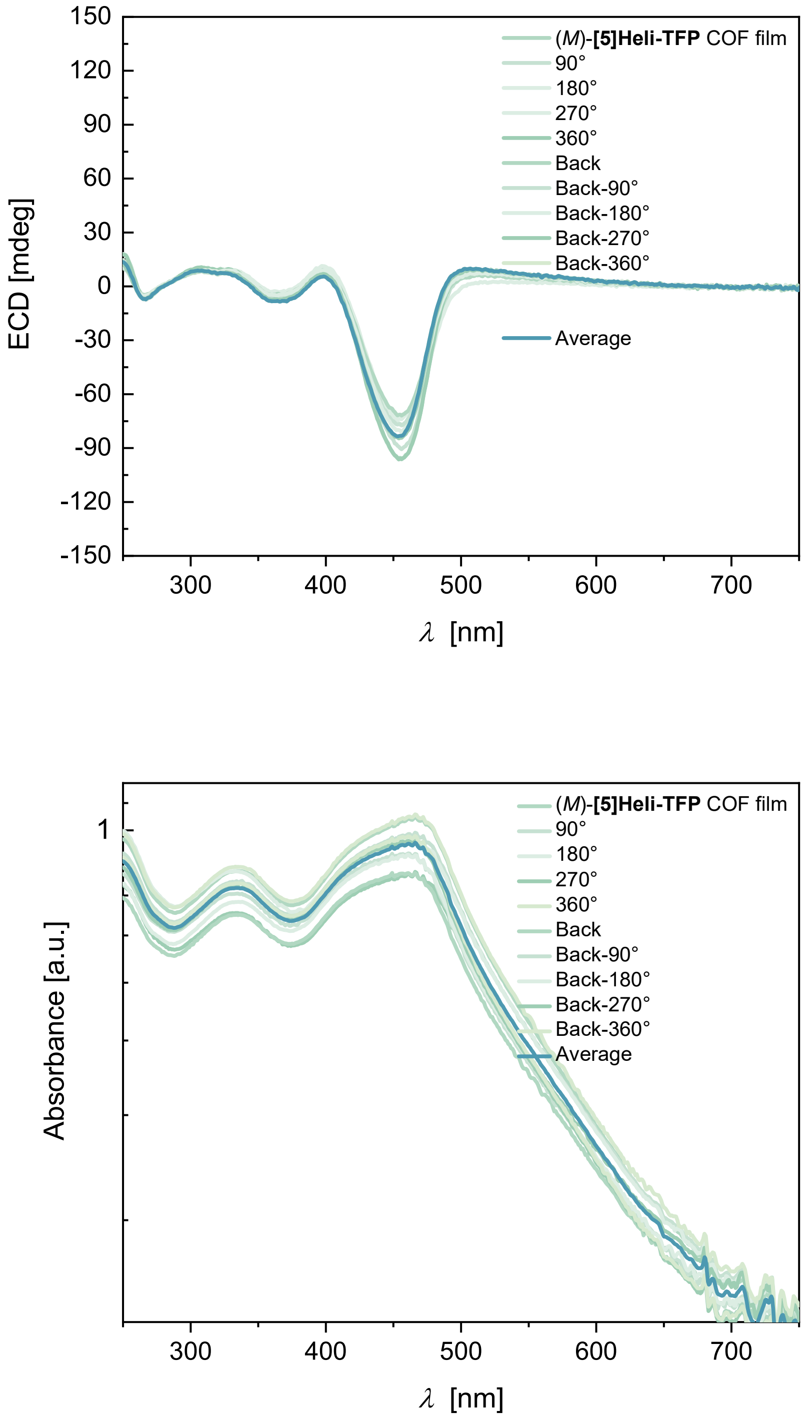

Figure S123. Characterization of (*M*)-**[5]Heli-TFP** COF film in quartz-substrate with concentration of 1.2 mM of (*M*)-(+)-**3**; (top) ECD spectra with different rotation angles; (bottom) corresponding UV-Vis spectra with different rotation angles at 298 K (logarithmically scaled on the Y-axis).

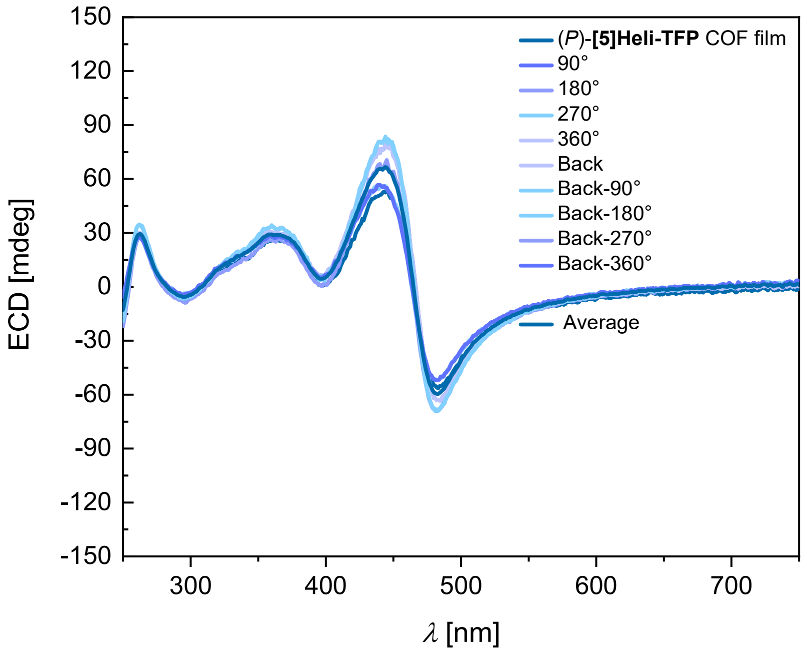

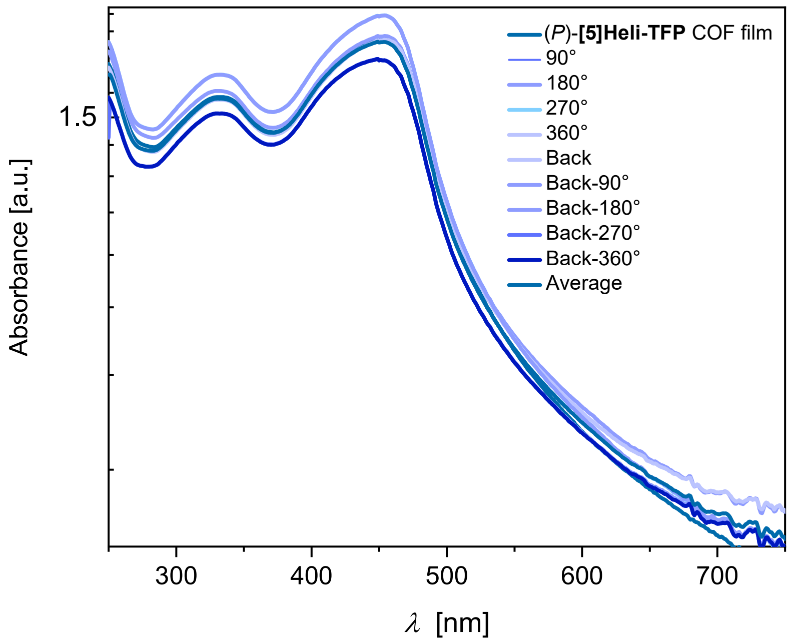

Figure S124. Characterization of (*P*)-**[5]Heli-TFP** COF film in quartz-substrate with concentration of 1.2 mM of (*P*)-(–)-**3**; (top) ECD spectra with different rotation angles; (bottom) corresponding UV-Vis spectra with different rotation angles at 298 K (logarithmically scaled on the Y-axis).

S18. Crystallographic Data

CCDC 2347463 ((±)-**3b**), CCDC 2401154 ((*M*)-**3**), CCDC 2400906 ((*M*)-(–)-**1**), CCDC-1951197 ((±)-**6**), CCDC-2127426 ((*R*,*R*)-(*P*/*M*)-**7**), CCDC-2372387 ((*R*,*R*)-(*P*)-(+)-**7**), contain the supplementary crystallographic data for this paper, including structure factors and refinement instructions. These data can be obtained free of charge from the joint Cambridge Crystallographic Data Centre (Cambridge Crystallographic Data Centre, 12 Union Road, Cambridge CB2 1EZ, UK (fax: +44(1223)-336-033; e-mail: deposit@ccdc.cam.ac.uk), or on graph via www.ccdc.cam.ac.uk/structures.

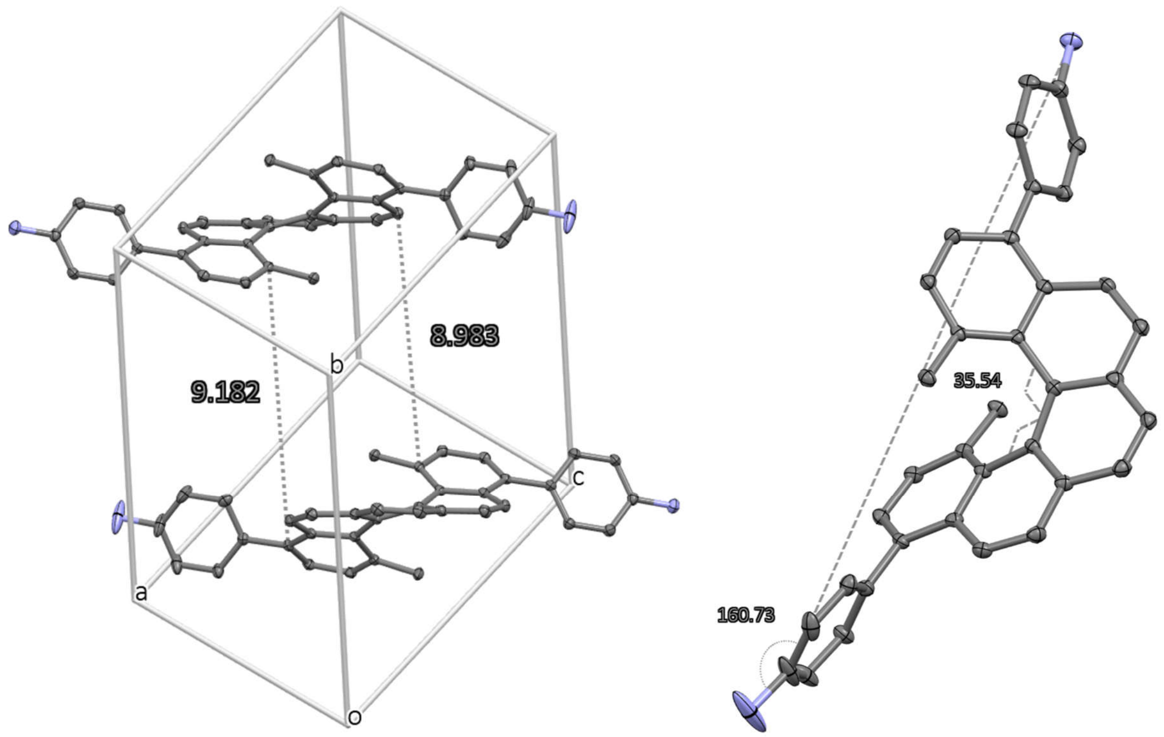

Figure S125. Single-crystal X-ray structure of (±)-**3b** at 100 K, hydrogen atoms are omitted for clarify, color code: carbon (grey), nitrogen (blue), yellow rods of (±)-**3b** were grown by slow evaporation from cyclohexane/CH_2Cl_2 at 25 °C. Distances in the unit cell in Å; angle ∠(N—C···N) in °; inner helical pitch dihedral ∠(C—C—C—C) in °.

Table S15. Crystal data and structure refinement for (±)-**3b**.

CCDC number	2347463
Empirical formula	$C_{36}H_{28}N_2$
Formula weight	488.63
Temperature/K	102.00
Crystal system	triclinic
Space group	P-1
a/Å	8.8916(11)
b/Å	12.0249(14)
c/Å	13.2232(16)
α/°	69.159(5)
β/°	85.116(6)
γ/°	69.729(6)
Volume/Å^3	1238.3(3)
Z	36
ρ_{calc}/g/cm^3	1.310
μ/mm^{-1}	0.076
F(000)	516.0
Crystal size/mm^3	0.837 × 0.338 × 0.292
Radiation	MoKα (λ = 0.71073)
2Θ range for data collection/°	4.106 to 67.03
Index ranges	$-13 \leq h \leq 13$, $-18 \leq k \leq 18$, $-20 \leq l \leq 20$
Reflections collected	56807
Independent reflections	9605 [R_{int} = 0.1774, R_{sigma} = 0.1351]
Data/restraints/parameters	9605/0/347
Goodness-of-fit on F^2	1.027
Final R indexes [$I \geq 2\sigma(I)$]	R_1 = 0.0899, wR_2 = 0.2013
Final R indexes [all data]	R_1 = 0.1371, wR_2 = 0.2331
Largest diff. peak/hole / e Å^{-3}	0.71/–0.76

Due to inherent limitations, the absolute configuration from the measured enantiopure single-crystal (*M*)-(+)-**3** cannot be directly determined with high accuracy. In this case, the calculated Friedif value of 8 for the corresponding chemical formula $C_{62}H_{44}N_2$ indicates *a priori* difficulties to achieve a Flack parameter less than 0.1.[30] This is primarily due to weak resonant scattering due to the absence of heavy heteroatoms. Based on the absolute configuration of (*M*)-(–)-**1** (Figure S127), which was obtained from the identical pure diastereomer (*R*,*R*)-(*M*)-(–)-**5** (Fraction A, Figure S104), it is possible to indirectly assign the absolute configuration to (*M*)-(+)-**3**, the molecular structure of the [5]helicene building block, along with *N*-benzophenone diimine motifs, can be visualized in the following crystal structure (Figure S126).

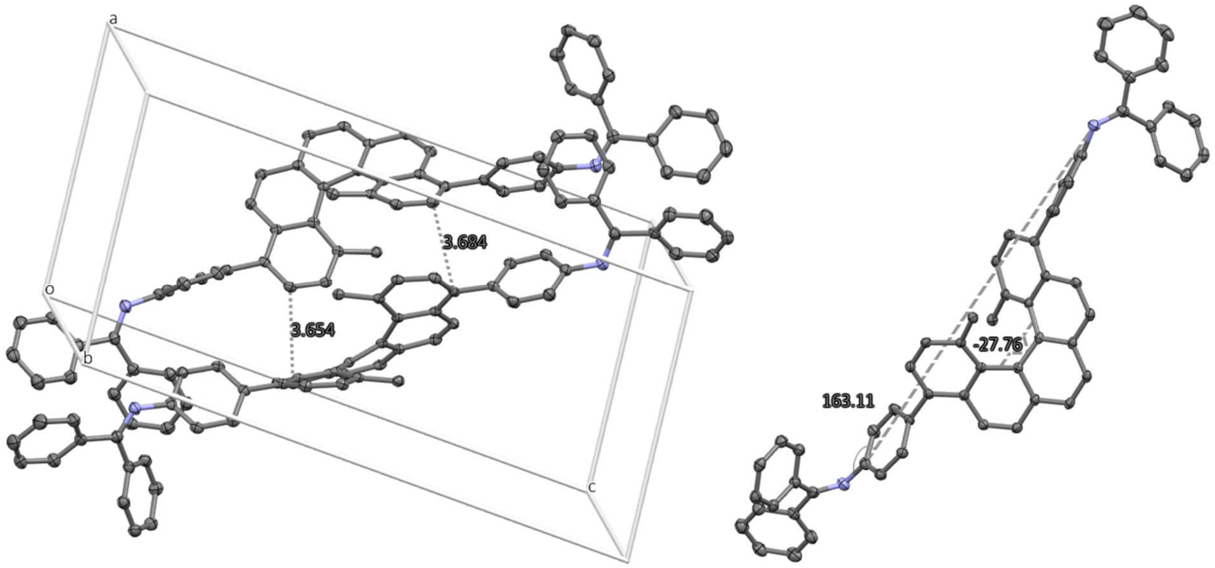

Figure S126. Single-crystal X-ray structure of (*M*)-(+)-**3** at 100 K, hydrogen atoms are omitted for clarify, color code: carbon (grey), nitrogen (blue); crystals of (*M*)-(+)-**3** were grown by slow evaporation from *n*-BuOH/chlorobenzene at 25 °C during two weeks. Later, based on the absolute configuration of (*M*)-(–)-**1**, we concluded that this crystal was configured as (*M*). Distances in the unit cell in Å; angle ∠(N—C···N) in °; inner helical pitch dihedral ∠(C—C—C—C) in °.

Table S16. Crystal data and structure refinement for (*M*)-(+)-**3**

CCDC number	2401154
Empirical formula	$C_{62}H_{44}N_2$
Formula weight	816.99
Temperature/K	100.00
Crystal system	monoclinic
Space group	$P2_1$
a/Å	10.7157(9)
b/Å	9.1712(10)
c/Å	22.425(3)
α/°	90
β/°	99.579(6)
γ/°	90
Volume/Å^3	2173.1(4)
Z	2
ρ_{calc}/g/cm^3	1.249
μ/mm^{-1}	0.547
F(000)	860.0
Crystal size/mm^3	0.166 × 0.087 × 0.037
Radiation	CuKα (λ = 1.54178)
2Θ range for data collection/°	7.996 to 150.04
Index ranges	$-13 \leq h \leq 13, -11 \leq k \leq 11, -28 \leq l \leq 28$
Reflections collected	107127
Independent reflections	8891 [R_{int} = 0.0239, R_{sigma} = 0.0141]
Data/restraints/parameters	8891/1/579
Goodness-of-fit on F^2	1.029
Final R indexes [$I \geq 2\sigma(I)$]	R_1 = 0.0470 wR_2 = 0.1103
Final R indexes [all data]	R_1 = 0.0586, wR_2 = 0.1175
Largest diff. peak/hole / e Å^{-3}	0.16/–0.21
Flack parameter	–0.3(8)

The absolute configuration of (*M*)-(–)-**1** was directly determined with high accuracy, yielding a Flack parameter of 0.020 in the Sohncke space group $P2_1$.[31] This enantiopure single crystal was obtained using the parent diastereomeric fraction (*R*,*R*)-(*M*)-(–)-**5** (Fraction A, Figure S104). Based on these measurements, we proposed that subsequent [5]helicene derivatives synthetized from (*R*,*R*)-(*M*)-(–)-**5** will hold an (*M*)-configuration. Further assignments were also validated by theoretical simulation of ECD spectra (Figure S109).

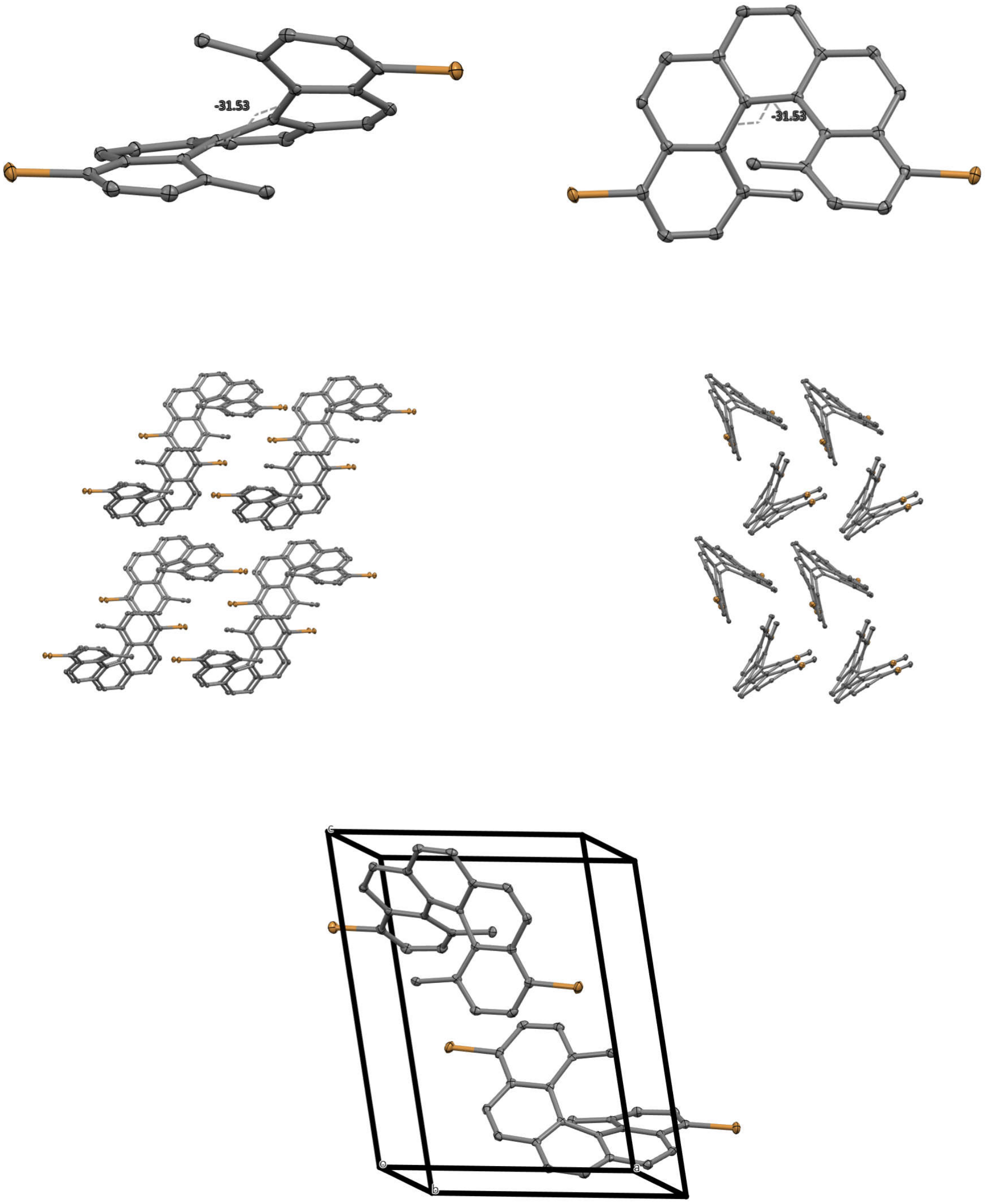

Figure S127. Single-crystal X-ray structure of (*M*)-(–)-**1** at 100 K, hydrogen atoms are omitted for clarify, color code: carbon (grey), nitrogen (blue); crystals were grown by slow evaporation of a saturated solution of (*M*)-(–)-**1** in $CHCl_3$ and vapors of CH_3CN at 10 °C during three days. Inner helical pitch dihedral in °.

Table S17. Crystal data and structure refinement for (*M*)-(–)-**1**.

CCDC number	2400906
Empirical formula	$C_{24}H_{16}Br_2$
Formula weight	464.19
Temperature/K	100.00
Crystal system	monoclinic
Space group	$P2_1$
a/Å	9.7447(4)
b/Å	7.2691(3)
c/Å	12.7578(5)
α/°	90
β/°	101.7770(10)
γ/°	90
Volume/Å^3	884.68(6)
Z	2
ρ_{calc}/g/cm^3	1.743
μ/mm^{-1}	5.825
F(000)	460.0
Crystal size/mm^3	0.147 × 0.133 × 0.068
Radiation	CuKα (λ = 1.54178)
2Θ range for data collection/°	9.27 to 127.564
Index ranges	$-11 \leq h \leq 11, -8 \leq k \leq 8, -14 \leq l \leq 14$
Reflections collected	28075
Independent reflections	2845 [R_{int} = 0.0239, R_{sigma} = 0.0141]
Data/restraints/parameters	2845/1/237
Goodness-of-fit on F^2	1.096
Final R indexes [$I \geq 2\sigma (I)$]	R_1 = 0.0126, wR_2 = 0.0332
Final R indexes [all data]	R_1 = 0.0126, wR_2 = 0.0332
Largest diff. peak/hole / e Å^{-3}	0.19/–0.23
Flack parameter	0.020(4)

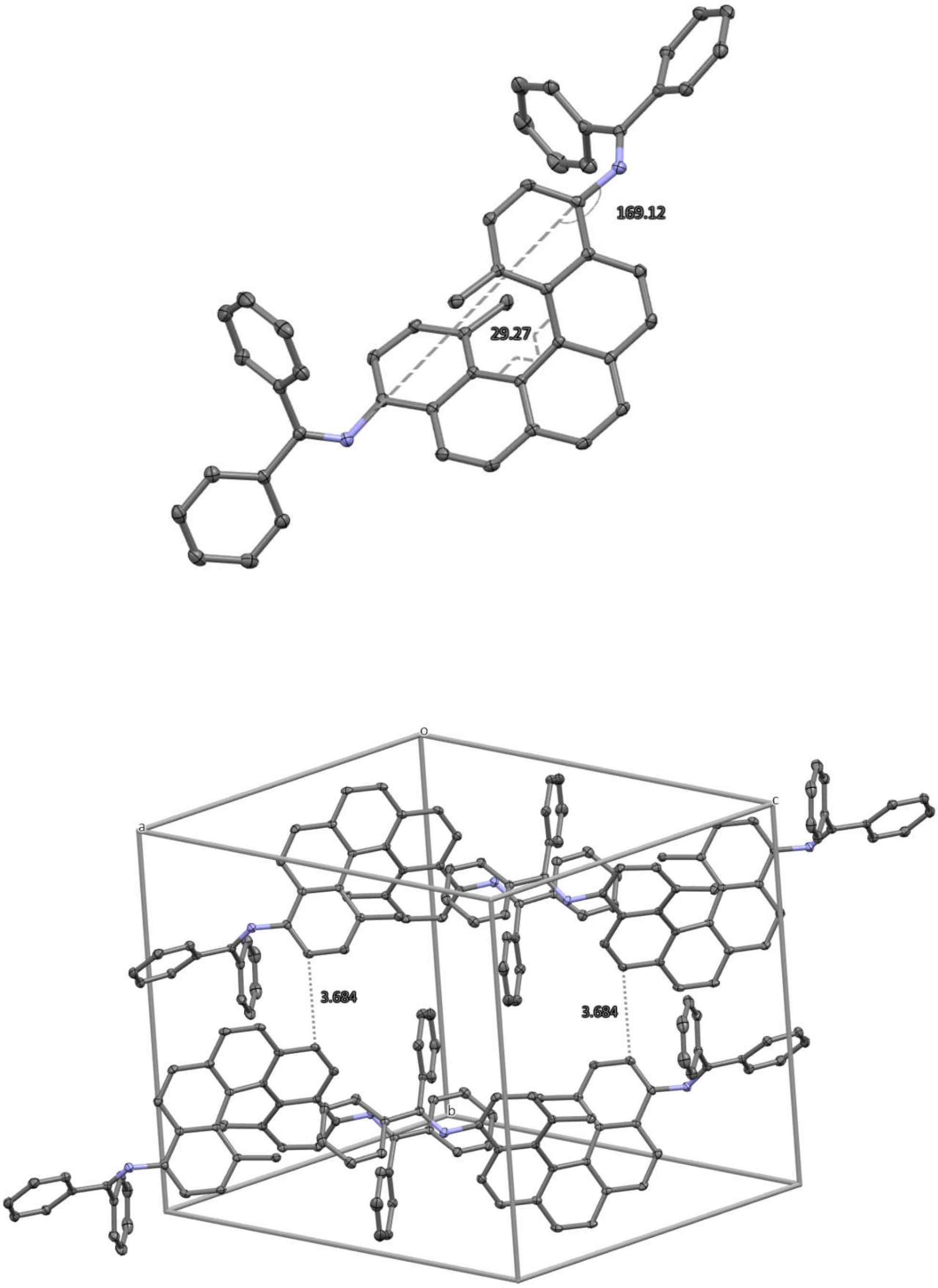

Figure S128. Single-crystal X-ray structure of (±)-**6** at 100 K. Solvent molecules and hydrogen atoms are omitted for clarity. The ellipsoids are displayed at 50% probability level. The crystals were grown by vapor phase diffusion of (7:3) CH_2Cl_2/MeOH into a saturated solution of (±)-**6** in (1:4) CH_2Cl_2/MeOH over 7 days at 25 °C. Color code: C, gray; N, blue. Distances in the unit cell in Å; angle ∠(N—C···N) in °; inner helical pitch dihedral ∠(C—C—C—C) in °.

Table S18. Crystal data and structure refinement for (±)-**6**.

CCDC number	1951197
Empirical formula	$C_{51}H_{39}Cl_2N_2$
Formula weight	750.74
Temperature/K	99.95
Crystal system	monoclinic
Space group	$P2_1$/n
a/Å	14.2334(10)
b/Å	15.5285(10)
c/Å	17.3466(13)
α/°	90
β/°	101.449 (3)
γ/°	90
Volume/Å^3	3757.7(5)
Z	4
ρ_{calc}/g/cm^3	1.327
μ/mm^{-1}	0.214
F(000)	1572.0
Crystal size/mm^3	0.564 × 0.378 × 0.316
Radiation	Mo$K\alpha$ (λ = 0.71073)
2θ range for data collection/°	4.892 to 55.86
Index ranges	$-18 \leq h \leq 18$, $-20 \leq k \leq 20$, $-22 \leq l \leq 22$
Reflections collected	111062
Independent reflections	8995 [R_{int} = 0.0593, R_{sigma} = 0.0267]
Data/restraints/parameters	995/0/499
Goodness-of-fit on F^2	1.055
Final R indexes [$I \geq 2\sigma$ (I)]	R_1 = 0.0491, wR_2 = 0.1225
Final R indexes [all data]	R_1 = 0.0647, wR_2 = 0.1322
Largest diff. peak/hole / e Å^{-3}	0.92/–0.82

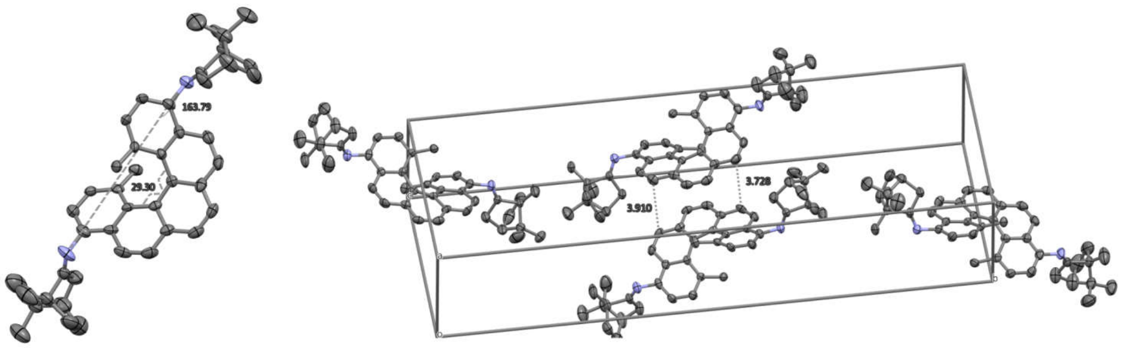

Figure S129. Single-crystal X-ray structure of (*R*,*R*)-(*P*/*M*)-**7** at 100 K. Solvent molecules and hydrogen atoms are omitted for clarity. The ellipsoids are displayed at 50% probability level. *R*1= 5.03%. The crystals were grown by layer diffusion of MeOH into a saturated solution of (*R*,*R*)-(*P*/*M*)-**7** in chlorobenzene over 7 days at 25 °C. Color code: C, gray; N, blue. Distances in the unit cell in Å; angle ∠(N—C···N) in °; inner helical pitch dihedral ∠(C—C—C—C) in °.

Table **S19**. Crystal data and structure refinement for (*R*,*R*)-(*P*/*M*)-**7**.

CCDC number	2127426
Empirical formula	$C_{44}H_{48}N_2$
Formula weight	604.84
Temperature/K	100.0
Crystal system	monoclinic
Space group	$P2_1$
a/Å	7.277(4)
b/Å	37.10(3)
c/Å	13.332(10)
α/°	90
β/°	91.10(2)
γ/°	90
Volume/Å^3	3598(4)
Z	4
ρ_{calc}/g/cm^3	1.116
μ/mm^{-1}	0.064
F(000)	1304.0
Crystal size/mm^3	0.705 × 0.453 × 0.134
Radiation	Mo$K\alpha$ (λ = 0.71073)
2θ range for data collection/°	3.762 to 57.198
Index ranges	$-8 \le h \le 9$, $-49 \le k \le 49$, $-17 \le l \le 17$
Reflections collected	84108
Independent reflections	17935 [R_{int} = 0.0816, R_{sigma} = 0.0771]
Data/restraints/parameters	17935/127/845
Goodness-of-fit on F^2	1.123
Final R indexes [$I \ge 2\sigma(I)$]	R_1 = 0.0970, wR_2 = 0.1983
Final R indexes [all data]	R_1 = 0.1467, wR_2 = 0.2219
Largest diff. peak/hole / e Å^{-3}	0.46/−0.27
Flack parameter	0.4(10)

Due to inherent limitations based on only light atoms (C, N, H), the absolute configuration of the measured enantiopure single-crystal (*R*,*R*)-(*P*)-(+)-**7** cannot be directly determined with high accuracy. In this case, the calculated Friedif value of 10 for the corresponding chemical formula $C_{44}H_{48}N_2$ indicates *a priori* difficulties to achieve a Flack parameter less than 0.1.[30] This is primarily due to weak resonant scattering due to the absence of heavy heteroatoms. The absolute configuration of (*R*,*R*)-(*P*)-(+)-**7** was validated using a combination of time-dependent density functional theory (TD-DFT) calculations, electronic circular dichroism spectroscopy (ECD), and optical rotation. Satisfyingly, the experimental and simulated ECD spectra show good agreement with the (*P*)-configuration (Figure S112).

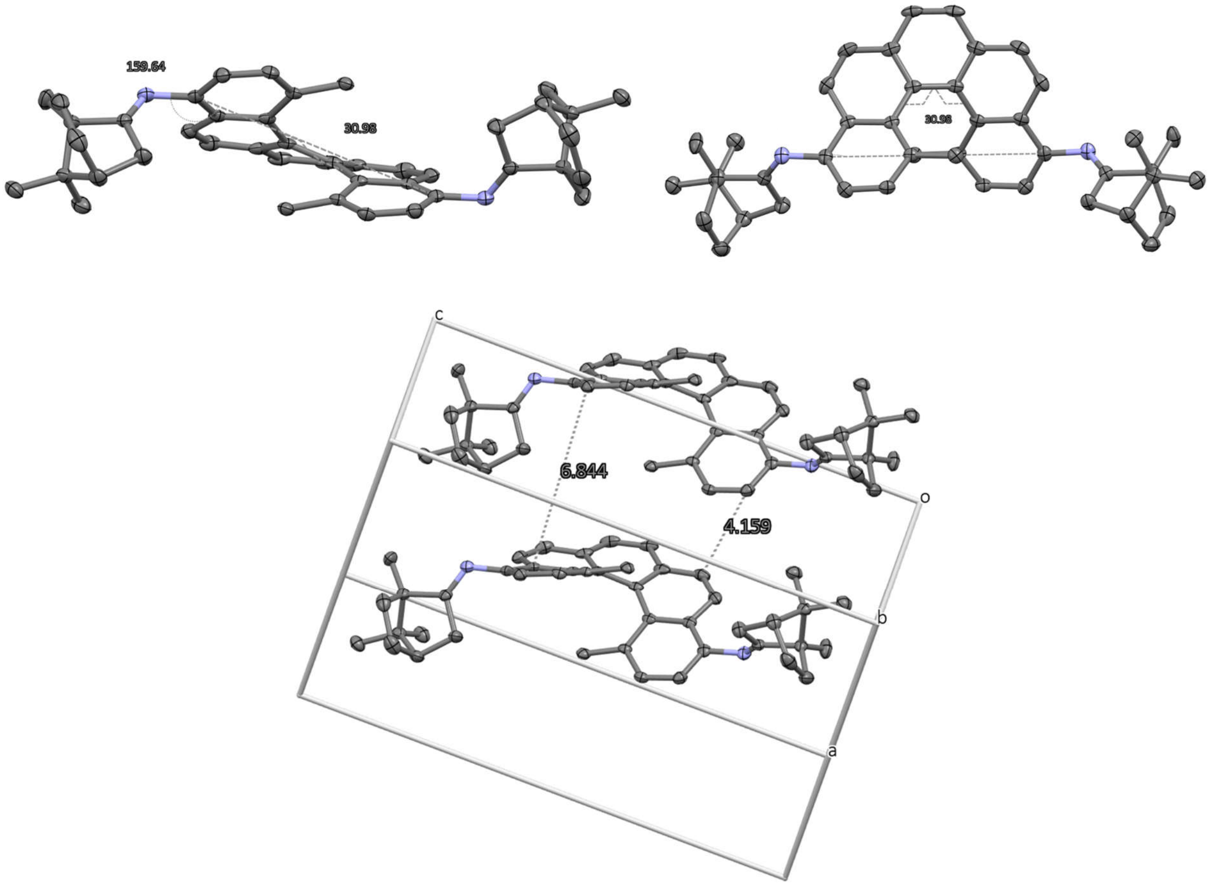

Figure S130. Single-crystal X-ray structure of (*R*,*R*)-(*P*)-(+)-**7** at 100 K. Solvent molecules and hydrogen atoms are omitted for clarity. The ellipsoids are displayed at 50% probability level. *R*1= 5.03%. The crystals were grown by layer diffusion of MeOH into a saturated solution of (*R*,*R*)-(*P*)-(+)-**7** in chlorobenzene over 7 days at 25 °C. Color code: C, gray; N, blue. Distances in the unit cell in Å; angle ∠(N—C···N) in °; inner helical pitch dihedral ∠(C—C—C—C) in °.

Table S20. Crystal data and structure refinement for (*R*,*R*)-(*P*)-(+)-**7**.

CCDC number	2372387
Empirical formula	$C_{44}H_{48}N_2$
Formula weight	604.84
Temperature [K]	100
Crystal system	monoclinic
Space group (number)	$C2$ (5)
a/Å	11.1912(8)
b/Å	8.5276(7)
c/Å	18.1987(15)
α/°	90
β/°	90.952(5)
γ/°	90
Volume/Å^3	1736.5(2)
Z	2
ρ_{calc}/g/cm^3	1.157
μ/mm^{-1}	0.499
F(000)	652
Crystal size/mm^3	0.632×0.542×0.054
Radiation	yellow
2θ range for data collection/°	plate
Index ranges	Cu$K\alpha$ (λ=1.54178 Å)
Crystal size/mm^3	4.86 to 149.08 (0.80 Å)
Radiation	$-13 \le h \le 13$, $-10 \le k \le 10$, $-22 \le l \le 22$
Reflections collected	28877
Independent reflections	3550 [Rint = 0.0507 Rsigma = 0.0278]
Data / Restraints / Parameters	3550/1/212
Goodness-of-fit on F^2	1.112
Final R indexes [$I \ge 2\sigma(I)$]	$R1$ = 0.0503 w$R2$ = 0.1390
Final R indexes [all data]	$R1$ = 0.0517 w$R2$ = 0.1400
Largest diff. peak/hole / e Å^{-3}	0.28/−0.23
Flack X parameter	−0.2(5)

S19. DFT calculations

The formation of homochiral COFs is strengthened by the β-ketoenamine-linked linkages, which in comparison with imine-bonds are more robust.[32] We used the transimination-approach,[33] and aniline-based modulation strategy which facilitates the self-correction of the lattices allowing the incorporation of enantiopure helicenes,[34] and promoting the formation of the homochiral 2D COFs with helical chirality. We must remark that the phenyl-spacer in the central annulated [5]helicene core enables the formation of more "planar" conformers during COF linkage construction, playing an essential role for achieving crystalline [5]HeliCOFs. To investigate these steric effects, a theoretical analysis of non-planar conformers was conducted. Where the steric restrictions between the imine-bond and the Hα from the ortho-fused aromatic rings (marked in red in Figure S131), representing the behavior of (4,11)-diimine-[5]helicene without the phenyl-extension. The results indicate that achieving a planar configuration requires overcoming an energetic barrier of 9.4 kcal mol^{-1}. In contrast, the non-planar conformers with the lowest energy correspond to the torsion angles (–139.4 and 139.5)°. This suggest that without a phenyl-spacer the presence of non-planar conformers is promoted, and the formation of homochiral lattices would be limited.

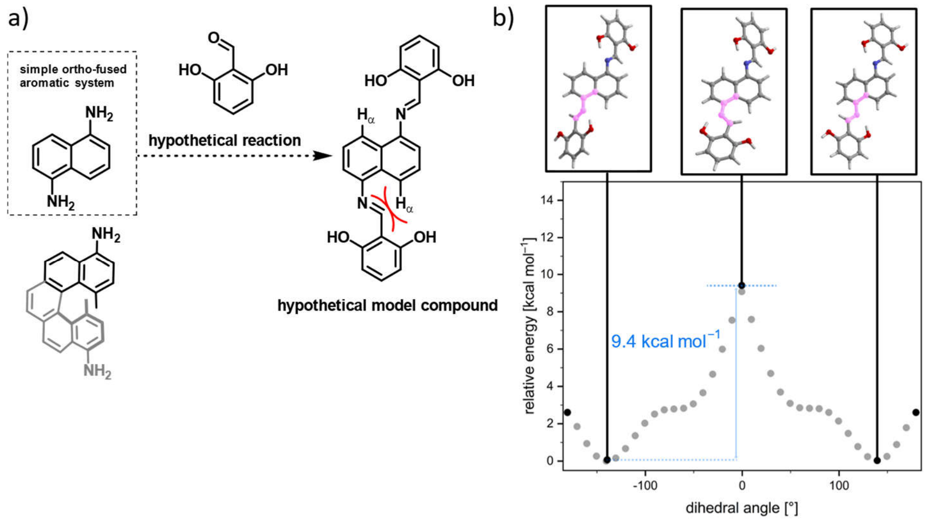

Figure S131. Representation of non-planar conformers caused between the imine linkages and α-hydrogens from [5]helicene building blocks without a phenyl-extension; (a) hypothetical model compound from 1,5-diaminonapthalene used for the DFT calculations with the possible interferences marked in red; (b) scan around the dihedral angle (pink) of the model compound and the resulting relative energy of the conformations, theoretical analysis was conducted DFT/B3LYP/6-31G(d) level of theory; the structures are confirmed ground-state minima according to the analysis of their analytical frequencies computed at the same level, which show no imaginary frequencies, and color code: carbon, grey; hydrogen, light gray; nitrogen, blue.

S20. Stability of [5]Heli-TPF COFs

We investigated the stability of the [5]HeliCOFs after exposure to basic, acid, humid, and organic solvents, and PXRD analysis revealed no significant changes in the crystalline reflexes (Figure S132). Furthermore, the homochiral and racemic **[5]Heli-TFP** COFs were analyzed under moisture and no significant difference changes were observed in PXRD (Figure S133).

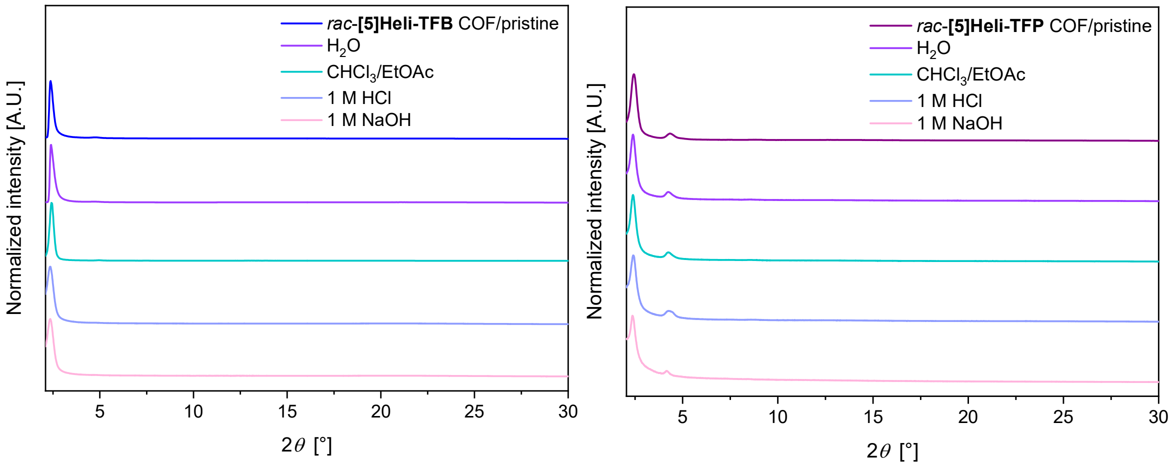

Figure S132. PXRD of [5]Heli-TFB and TFP COFs after treatment with various conditions; 3 mg of the COF was suspended in 3 mL of solvent (H_2O, $CHCl_3$/EtOAc, 1 M HCl, and 1 M NaOH) for 72 h. The materials were washed with acetone and methanol, dried with *sc*CO_2.

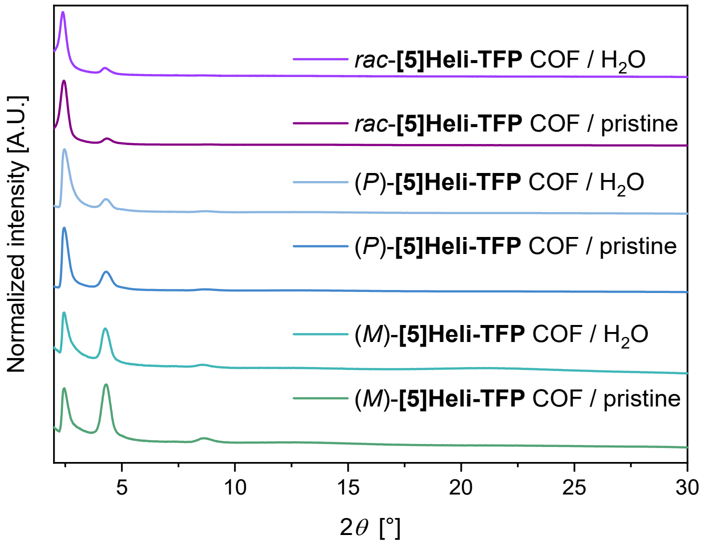

Figure S133. PXRD of homochiral and racemic **[5]Heli-TFP** COFs under moisture 3 mg of the COF was suspended in 3 mL of H_2O. The materials were washed with acetone and methanol, dried with *sc*CO_2.

S21. Photoluminescence spectroscopy

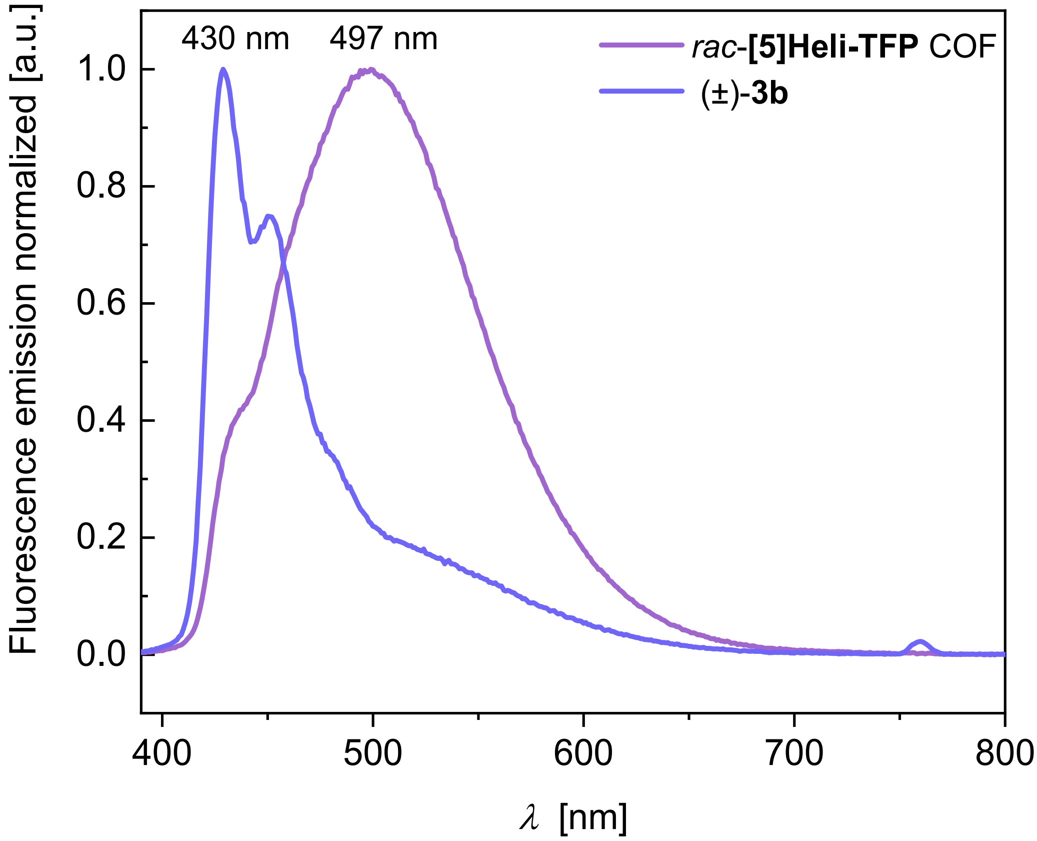

Figure S134. Emission spectra of *rac*-**[5]Heli-TFP** COF in suspension ($c \approx 0.05$ mg mL^{-1}) in DMSO and (±)-**3b** in CH_2Cl_2 at 298 K (λ_{ex} = 380 nm).

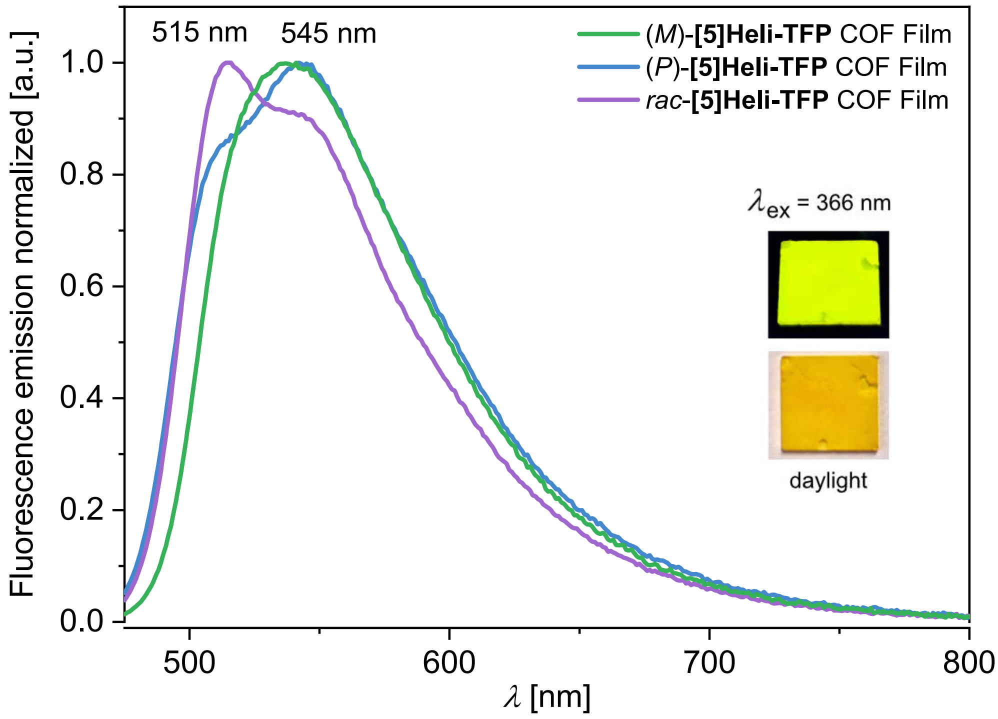

Figure S135. Fluorescence emission spectra (λ_{ex} = 460 nm) of enantiopure and racemic films at 298 K.

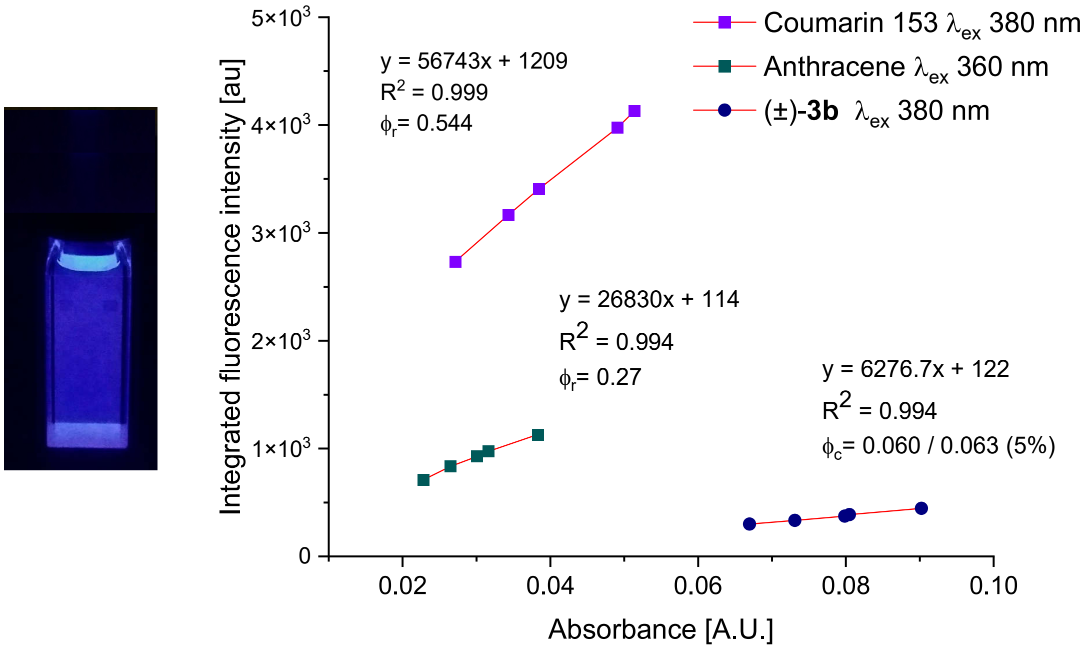

Figure S136. Relative fluorescence quantum yield determination of (±)-**3b** in CH_2Cl_2 at 298 K.[35]

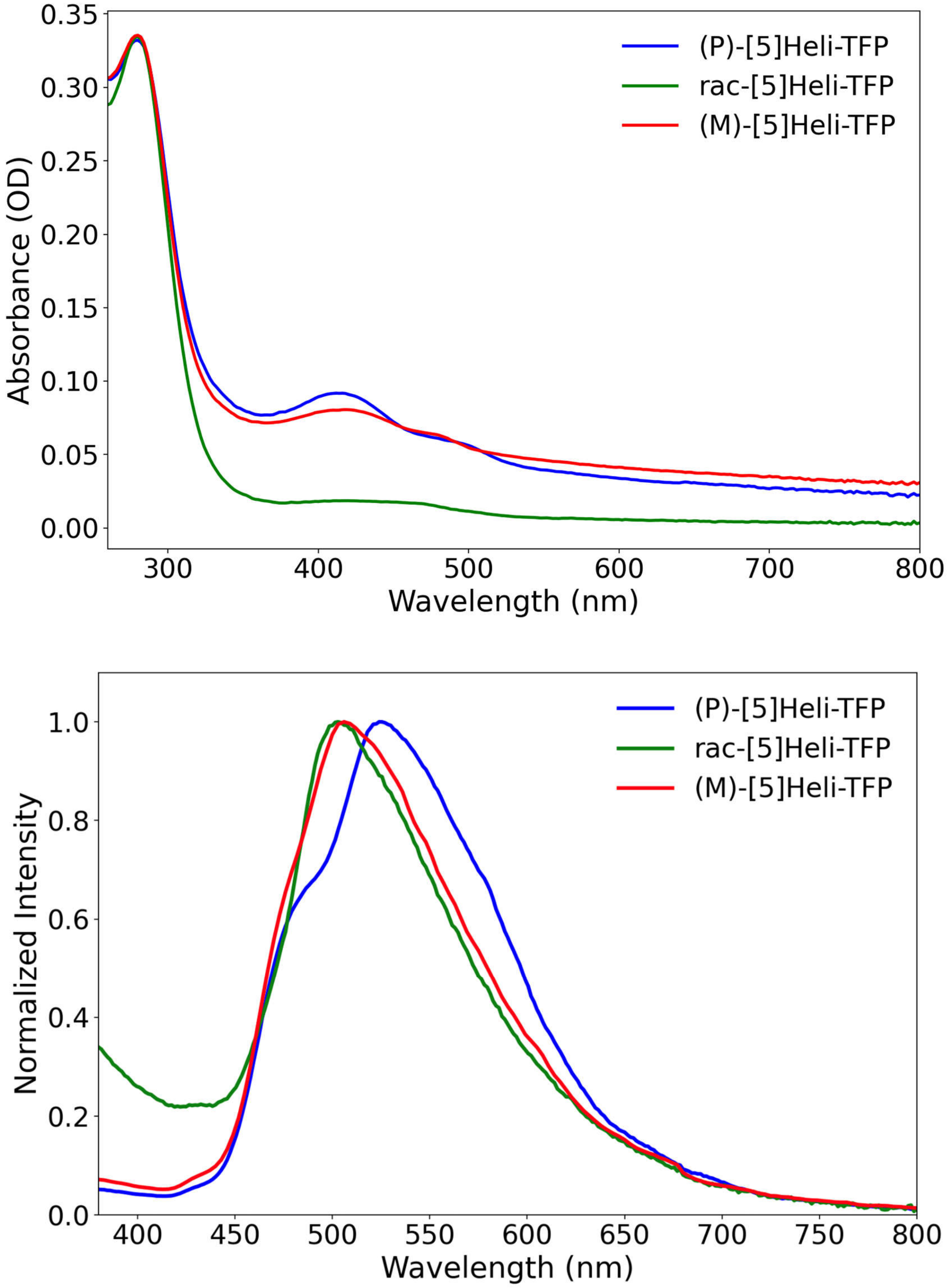

Figure S137. Comparative analysis of absorbance and PL of homochiral and racemic **[5]Heli-TFP COF** as powder dispersion 0.03 mg mL^{-1} in DMSO at 298 K; (top) absorbance spectra (solid-lines); (bottom) PL emission scan, (λ_{ex} = 300 nm).

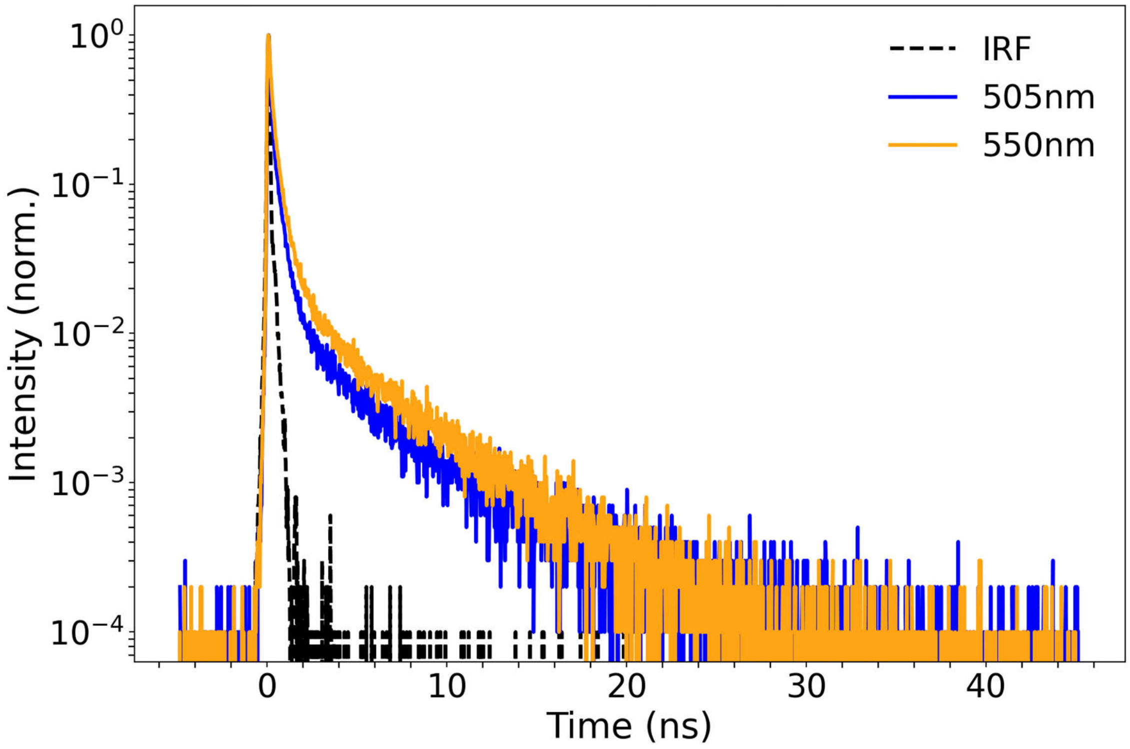

Figure S138. Time-correlated single-photon counting (TCSPC) measurement of *rac*-**[5]Heli-TFP** COF as powder dispersion 0.03 mg mL^{-1} in DMSO at 298 K. Using the following conditions: fluence: ~ 0.18 μJ cm^{-2}, power = 150 μW, rep rate= 50 kHz, spot size = ~ 3.25 mm diameter, λ = 375 nm, FWHM of IRF: 130 ps.

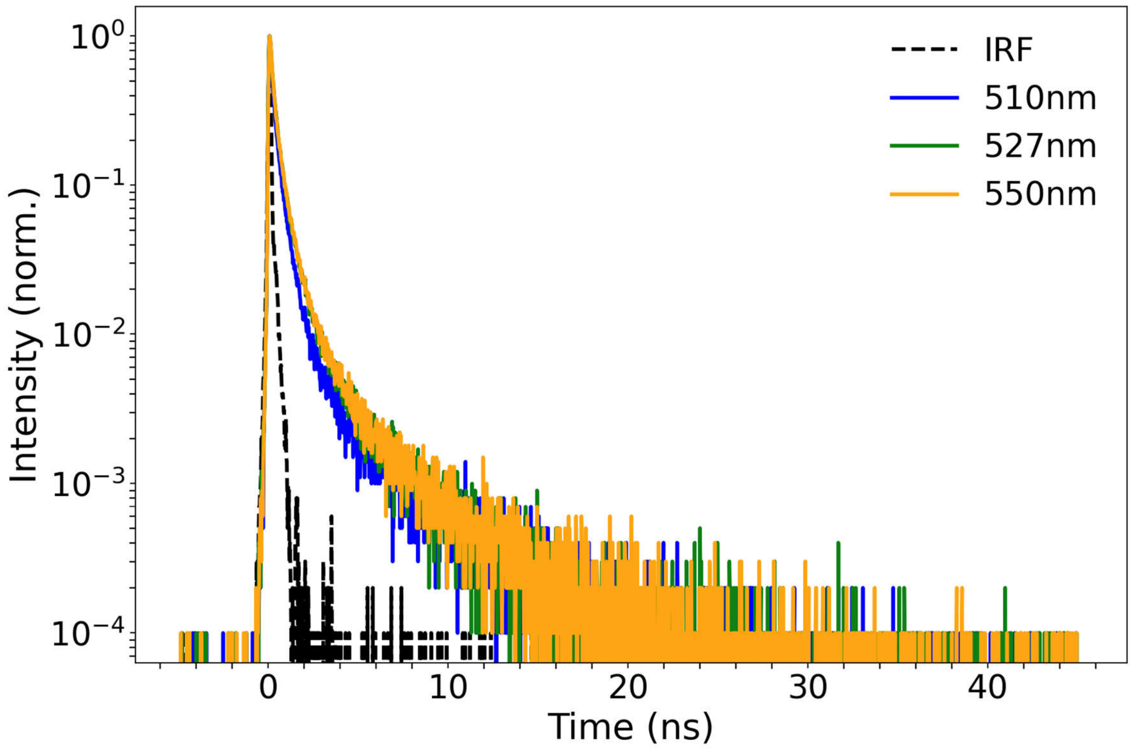

Figure S139. Time-correlated single-photon counting (TCSPC) measurement of (*P*)-**[5]Heli-TFP** COF as powder dispersion 0.03 mg mL^{-1} in DMSO at 298 K. Using the following conditions: fluence: ~ 0.18 μJ cm^{-2}, power = 150 μW, rep rate= 50 kHz, spot size = ~ 3.25 mm diameter, λ = 375 nm, FWHM of IRF: 130 ps.

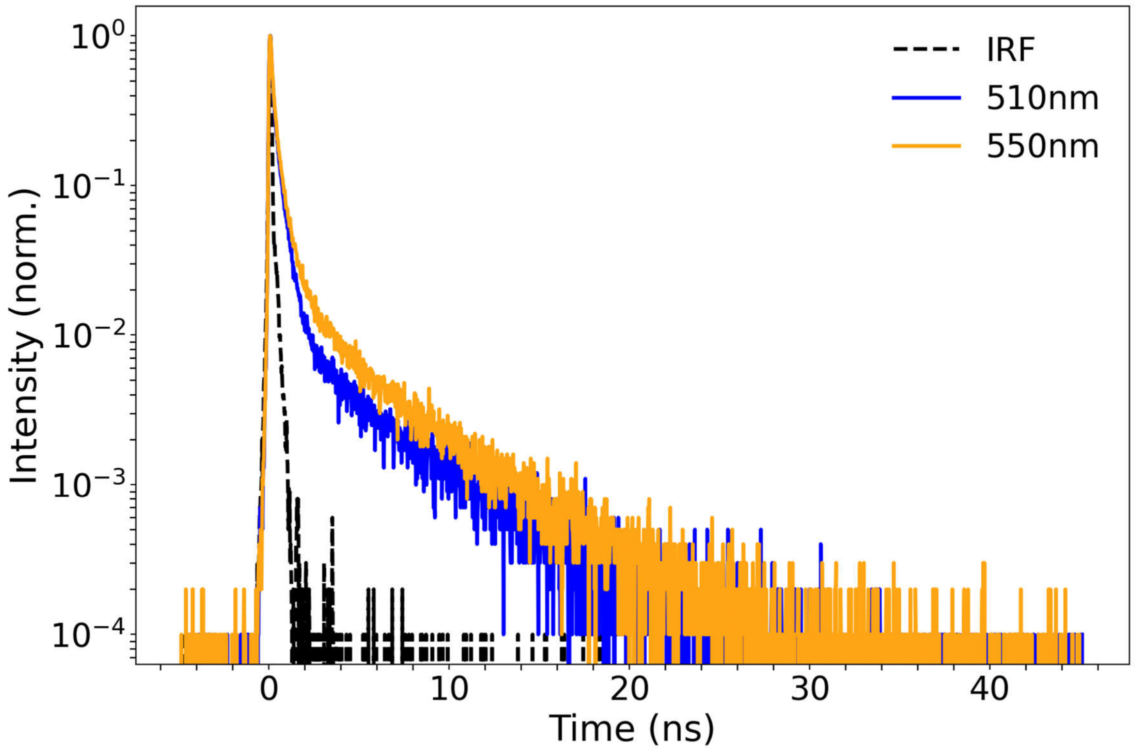

Figure S140. Time-correlated single-photon counting (TCSPC) measurement of (*M*)-**[5]Heli-TFP** COF as powder dispersion 0.03 mg mL^{-1} in DMSO at 298 K. Using the following conditions: fluence: ~ 0.18 μJ cm^{-2}, power = 150 μW, rep rate= 50 kHz, spot size = ~ 3.25 mm diameter, λ = 375 nm, FWHM of IRF: 130 ps.

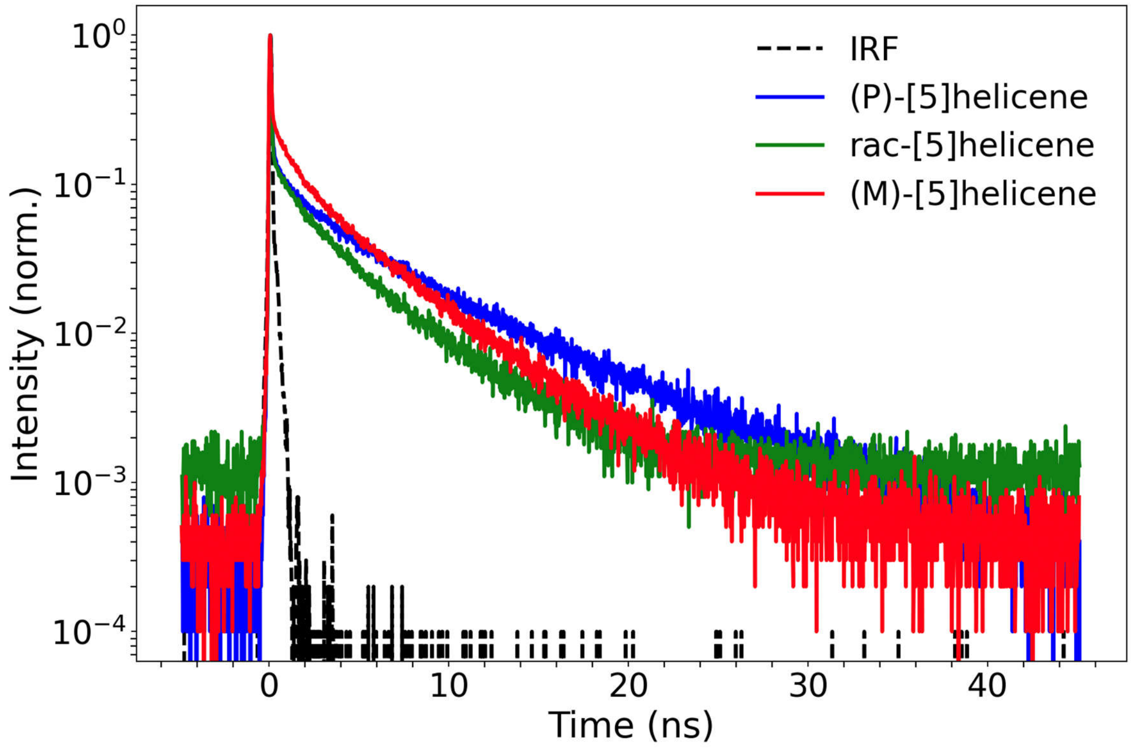

Figure S141. Time-correlated single-photon counting (TCSPC) measurement of building blocks: (±)-**3**, (*M*)-(+)-**3**, and (*P*)-(–)-**3** at 5.4 μM in CH_2Cl_2 at 298 K. Using the following conditions: fluence: ~ 0.18 μJ cm^{-2}, power = 150 μW, rep rate= 50 kHz, spot size = ~ 3.25 mm diameter, λ = 375 nm, FWHM of IRF: 130 ps.

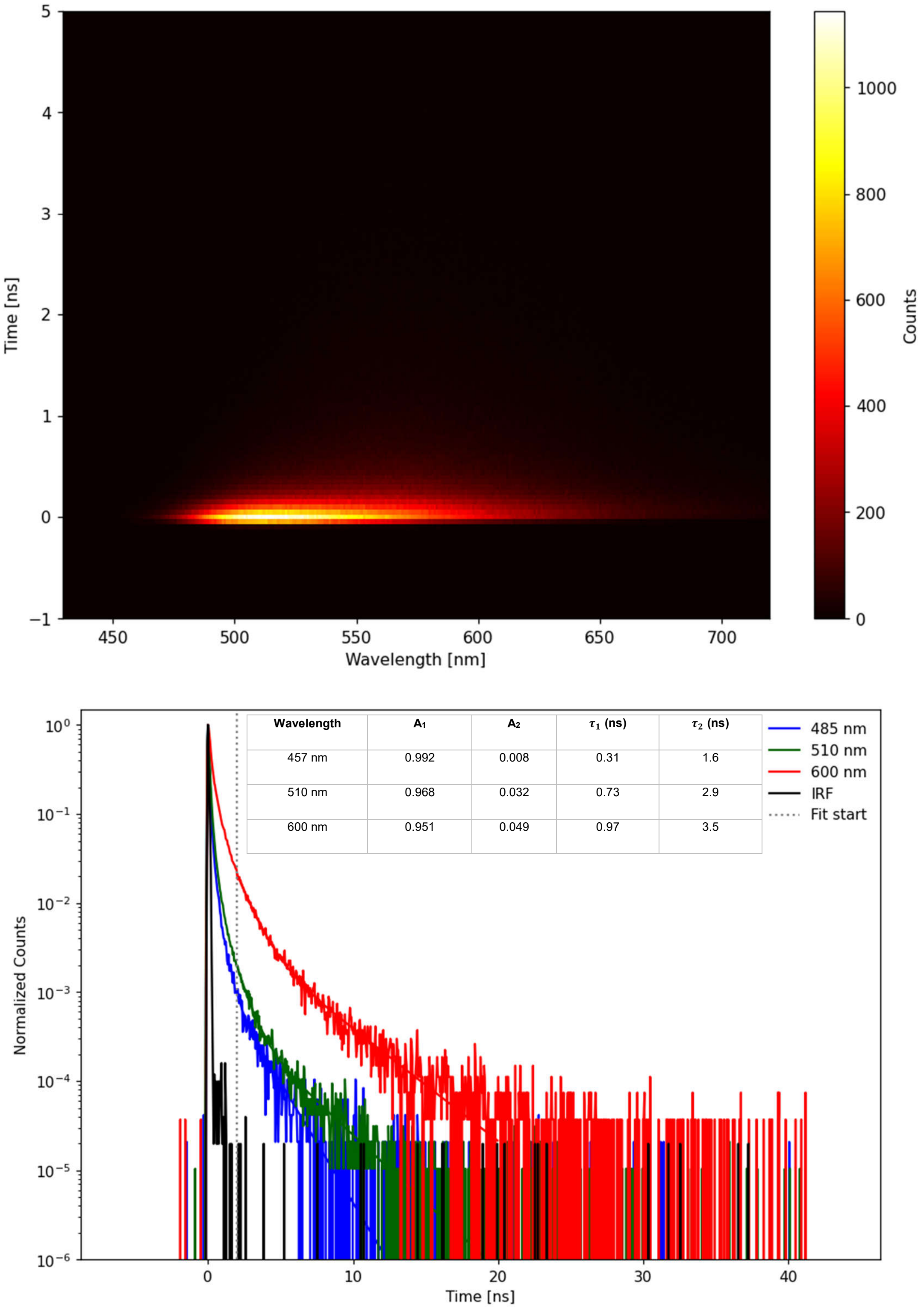

Wavelength	A_1	A_2	τ_1 (ns)	τ_2 (ns)
457 nm	0.992	0.008	0.31	1.6
510 nm	0.968	0.032	0.73	2.9
600 nm	0.951	0.049	0.97	3.5

Figure S142. Time-correlated single-photon counting (TCSPC) measurement of (*M*)-**[5]Heli-TFP** COF film at 298 K. Using the following conditions: fluence: ~ 1.2 μJ cm^{-2}, power= 160 μW, rep rate= 50kHz, spot size = ~ 850 μm diameter, excitation wavelength = 343 nm. Reasonable biexponential fit from 2 ns onwards.

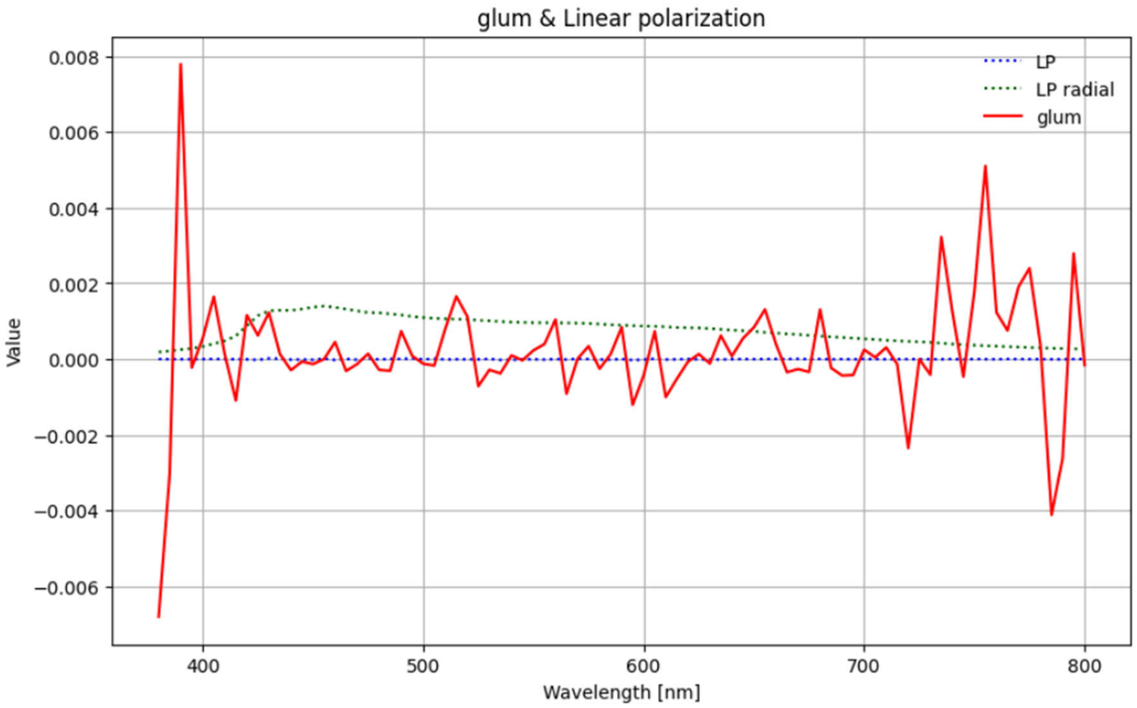

Figure S143. CPL measurements of (*M*)-(+)-**3** in CH_2Cl_2 at 1.3×10^{-7} M 298 K.[36]

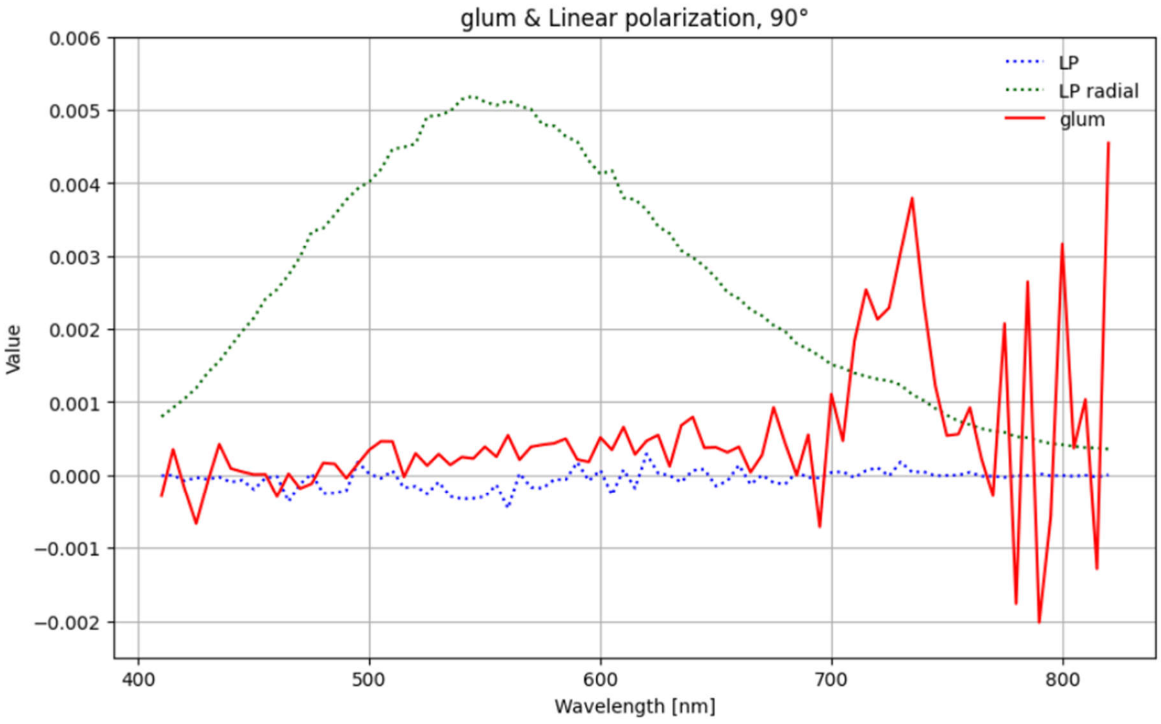

Figure S144. CPL measurements of (*P*)-**[5]Heli-TFP** COF film at 298 K.[36]

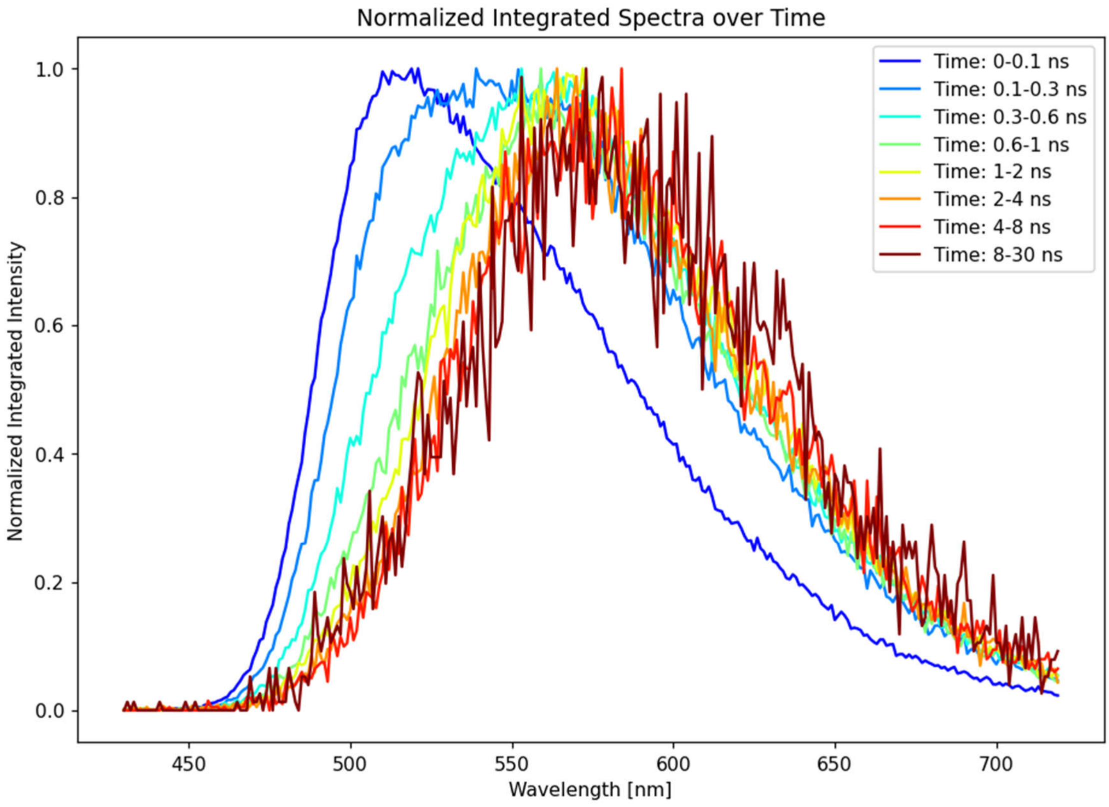

Figure S145. Spectral evolution extracted from TCSPC measurements presented in Figure S142 (top) for (*M*)-**[5]Heli-TFP** COF film at 298 K.

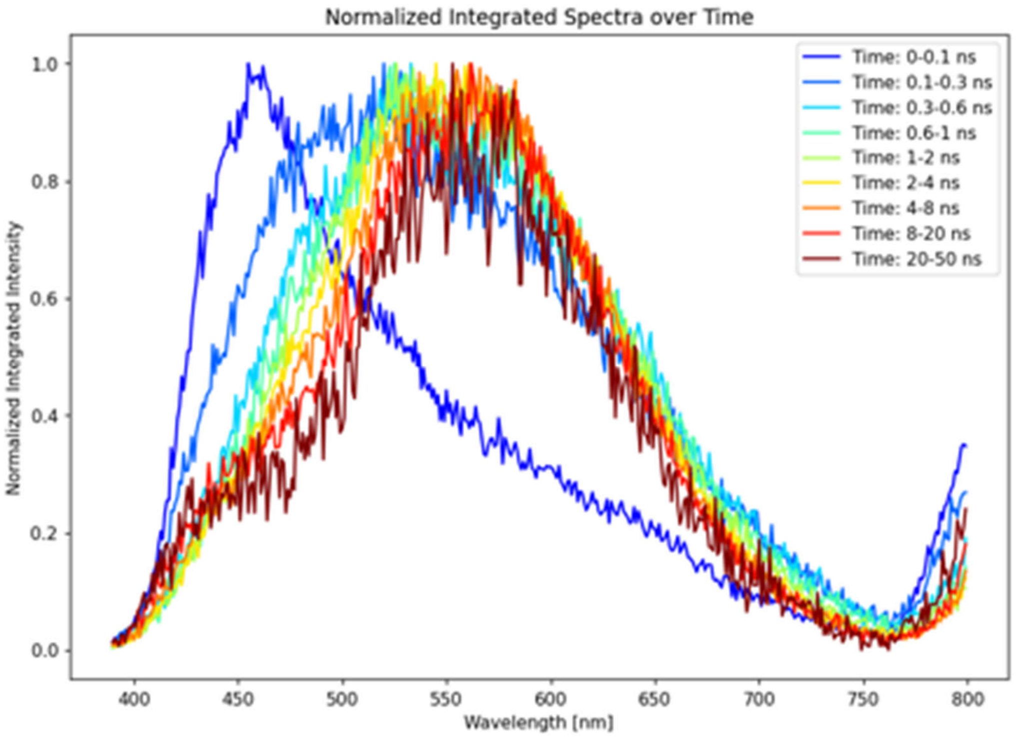

Figure S146. Transient PL measurements of (*M*)-(+)-**3** at 5.4 μM in CH_2Cl_2 at 298 K over time. Extracted from time-correlated single-photon counting (TCSPC) measurement of (*M*)-(+)-**3**, using the following conditions: fluence = ~1.2 μJ cm^{-2}, power= 160 μW, rep rate= 50kHz, spot size = ~ 850 μm diameter, excitation wavelength = 343 nm.

S22. Cartesian coordinates

***rac*-[5]Heli-TFP** COF AA-inclined

a = *b* = 45.7 Å, *c* = 7.4 Å

C	0.312394	24.891266	1.788912
C	1.630959	25.348425	1.865844
N	0.153069	29.985617	1.580695
C	5.107977	23.922255	2.340249
C	4.962553	22.529953	2.288574
C	6.080475	21.703593	2.435491
C	7.366407	22.236754	2.643563
C	7.483049	23.641281	2.696106
C	6.372311	24.471162	2.545932
C	9.753649	22.079738	2.991894
C	8.530532	21.423623	2.778032
C	8.552680	19.991457	2.701172
C	9.798152	19.281238	2.669328
C	10.942316	20.001203	3.008974
C	10.937517	21.379846	3.141416
C	1.891130	26.725020	1.828862
C	0.842132	27.639796	1.717697
N	-2.439986	24.039928	1.523521
C	0.380332	31.396234	1.541325
C	1.670982	31.941023	1.578380
C	1.848498	33.328509	1.551167
C	0.747470	34.209616	1.484367
C	-0.533462	33.629639	1.438121
C	-0.718318	32.248547	1.464971
C	-0.100202	36.608119	1.534015
C	0.948858	35.624914	1.532712
C	2.266511	36.081778	1.688024
C	2.551029	37.417351	1.912670
C	1.519155	38.340004	1.987286
C	0.186277	37.999438	1.757502
C	-0.478350	27.175284	1.654009
C	-0.746206	25.804470	1.685129
N	3.993494	24.803210	2.184213
C	-3.781839	23.547318	1.507888
C	-4.876196	24.381420	1.774727
C	-6.172262	23.857739	1.786307
C	-6.414577	22.492978	1.521856
C	-5.296822	21.682210	1.252267
C	-4.000888	22.196946	1.246405
C	-8.119308	20.630762	1.238734
C	-7.739778	21.966524	1.605157
C	-8.725236	22.792394	2.165613
C	-9.997632	22.321655	2.436939
C	-10.326416	21.009209	2.135940
C	-9.447957	20.141898	1.485959
C	23.240360	14.502764	1.539950
C	21.999322	13.861116	1.520988
N	24.127433	9.476305	1.465261
C	18.353616	14.878124	1.378413
C	18.387868	16.274835	1.282375
C	17.200166	17.004126	1.238661
C	15.942213	16.368025	1.295402
C	15.934578	14.963794	1.387388
C	17.121020	14.227243	1.424587
C	13.419895	16.644283	1.475538
C	14.749139	17.150488	1.274993
C	14.892370	18.530519	1.065545
C	13.798899	19.373772	1.011121
C	12.529043	18.869241	1.233523
C	12.304661	17.540836	1.586775
C	21.941508	12.461946	1.545956
C	23.117578	11.709356	1.580768
N	25.841499	15.759091	1.584965
C	24.073330	8.047543	1.473149
C	22.895697	7.363402	1.797814
C	22.867267	5.968797	1.775596
C	24.002096	5.212726	1.427916
C	25.176810	5.924092	1.105061
C	25.214639	7.320426	1.133504
C	25.073179	3.165053	0.720834
C	23.978682	3.787356	1.339332
C	22.896421	2.950778	1.776102
C	22.930843	1.528112	1.574906
C	24.018404	1.019364	0.866448
C	25.090878	1.804348	0.474476
C	24.359199	12.359154	1.594012
C	24.423791	13.755211	1.579755
N	19.556094	14.106001	1.440292
C	27.099971	16.438730	1.609138
C	28.307617	15.736218	1.685347
C	29.519794	16.426257	1.693742
C	29.572244	17.831347	1.627593
C	28.341730	18.517662	1.554279
C	27.123779	17.832583	1.547838
C	30.728271	19.935242	1.335351
C	30.804485	18.553907	1.568537
C	32.111889	17.964041	1.646555
C	33.294350	18.745848	1.405698
C	33.107090	20.091040	1.089284
C	31.859813	20.694840	1.097896
C	13.180499	15.274935	1.575846
C	7.370711	19.253052	2.706889
C	9.834387	17.867076	2.375928
C	8.579525	17.188366	2.475152
C	7.384294	17.875853	2.632490
C	8.488002	15.808019	2.415589
C	9.614020	15.041849	2.220616
C	10.847496	15.640452	2.021736
C	11.004673	17.062402	2.006827
C	11.915953	14.780925	1.815328
C	-9.803265	18.772541	1.174539
C	-8.723597	17.936653	0.743342
C	-7.476906	18.446419	0.433369
C	-7.190240	19.770647	0.660113
C	34.599285	18.121453	1.370018
C	-8.826025	16.556908	0.719603
C	35.714186	15.940134	1.117726
C	34.602685	16.692700	1.453392
C	32.255577	16.602542	1.902682
C	33.471590	15.973020	1.791083
C	21.776267	3.510616	2.384106
C	21.828585	0.691438	2.001254
C	20.676358	1.388138	2.490350
C	20.680784	2.751601	2.718035
C	-0.885704	38.965751	1.874095
C	19.455624	0.760781	2.664189
C	-3.295204	39.164500	2.345459
C	-2.205890	38.417167	1.933585
C	-2.464433	37.093893	1.629764
C	-1.430563	36.216954	1.407580
C	20.782876	14.671145	1.461353
C	25.731898	14.412365	1.606171
C	23.027056	10.249414	1.590466
C	2.721809	24.382823	2.010735
C	-2.136681	25.350926	1.630488
C	1.142037	29.072754	1.677458
C	-1.887819	24.527768	-1.982003
C	-0.583961	24.041122	-1.857883
N	-0.292545	29.136383	-1.916071
C	3.424309	22.567535	-1.287730
C	3.561753	21.194070	-1.091600
C	4.828433	20.628386	-0.937518
C	5.997723	21.416068	-0.977323
C	5.835050	22.800481	-1.175552
C	4.569412	23.373279	-1.325509
C	8.537209	21.478799	-0.916457
C	7.277078	20.797229	-0.842927
C	7.303130	19.409105	-0.635868
C	8.493541	18.718151	-0.491991
C	9.698508	19.388832	-0.624433
C	9.766803	20.741108	-0.955322
C	0.497905	24.927643	-1.764595
C	0.267453	26.305703	-1.804183
N	-4.281098	24.013180	-2.026657
C	-0.492844	30.551660	-1.958192
C	0.619132	31.388378	-1.905143
C	0.457776	32.773050	-1.939592
C	-0.812651	33.370881	-2.029118
C	-1.926439	32.505424	-2.088463
C	-1.772371	31.115695	-2.051264
C	-2.292841	35.261093	-2.337136
C	-0.993457	34.787775	-2.094181
C	0.059105	35.760753	-1.991846
C	-0.211957	37.165323	-2.132394
C	-1.519904	37.528003	-2.456696
C	-2.553964	36.607361	-2.527767
C	-1.038094	26.801312	-1.926638
C	-2.110811	25.910563	-2.009939

N	2.113406	23.113188	-1.451582
C	-5.414152	23.146360	-2.110827
C	-6.680124	23.677964	-1.880391
C	-7.807137	22.861113	-1.948567
C	-7.714454	21.490643	-2.253184
C	-6.423690	20.971971	-2.493245
C	-5.286468	21.784500	-2.417977
C	-8.675164	19.354942	-2.867485
C	-8.866801	20.650387	-2.363263
C	-10.213283	21.055428	-2.066534
C	-11.322876	20.172120	-2.299937
C	-11.038401	18.937270	-2.885433
C	-9.741397	18.512505	- 3.134396
C	23.007929	13.021450	-2.062988
C	21.757936	13.643938	-2.111188
N	20.947177	8.610950	-1.711980
C	17.855116	15.501970	-2.249203
C	17.800219	16.895346	-2.222860
C	16.567890	17.552529	-2.257164
C	15.355397	16.842286	-2.326570
C	15.434533	15.433716	-2.363335
C	16.662640	14.771035	-2.322083
C	12.955094	16.672339	-2.542892
C	14.081181	17.485867	-2.344880
C	13.857606	18.891995	-2.152658
C	12.524493	19.414192	-2.036789
C	11.483598	18.554403	-2.376724
C	11.678149	17.200443	-2.592757
C	20.585327	12.877553	-2.084989
C	20.670643	11.484342	-2.007116
N	25.445414	13.295964	-1.960725
C	21.020358	7.182684	-1.671017
C	19.879813	6.449072	-1.340635
C	19.925075	5.051907	-1.308284
C	21.106819	4.346814	-1.619343
C	22.244360	5.110640	-1.942343
C	22.208168	6.504407	-1.968831
C	22.226203	2.096061	-1.993438
C	21.127880	2.918581	-1.571142
C	20.009817	2.276244	-1.016839
C	19.987271	0.908156	-0.807609
C	21.083653	0.137214	-1.161222
C	22.200587	0.672012	-1.803676
C	21.920875	10.853463	-1.962245
C	23.085685	11.624369	-1.993655
N	19.128657	14.852254	-2.191450
C	26.646740	14.072308	-1.960725
C	27.872529	13.432686	-1.772266
C	29.057595	14.173079	-1.755331
C	29.054839	15.574068	-1.929822
C	27.805785	16.192921	-2.121031
C	26.618527	15.460719	-2.136519
C	30.404333	17.727817	-2.057633
C	30.281308	16.307500	-1.876918
C	31.448535	15.588233	-1.577149
C	32.670731	16.216545	-1.416264
C	32.765511	17.589828	-1.572879
C	31.680840	18.380197	-1.951823
C	14.926364	19.780814	-2.062337
C	11.017281	21.404503	-1.248938
C	10.990783	22.831567	-1.146241
C	9.806611	23.534446	-0.980363
C	8.600670	22.870810	-0.903575
C	12.295251	20.783079	-1.622672
C	13.453092	21.624615	-1.615434
C	14.728252	21.123612	-1.827053
C	13.365726	22.992207	-1.415685
C	12.145242	23.592175	-1.211956
C	-10.476060	22.342421	-1.602842
C	-12.680229	20.613882	-2.061468
C	-12.837614	22.009132	-1.788478
C	-11.759331	22.822844	-1.493993
C	-14.071932	22.632330	-1.839645
C	30.493831	21.903800	-2.101635
C	30.560869	20.527253	-2.227564
C	31.796842	19.812614	-2.119149
C	29.346713	19.879997	-2.359427
C	29.277026	18.508853	-2.294943
C	23.344675	-0.139699	-2.154974
C	24.516740	0.578030	-2.553459
C	24.490502	1.934443	-2.821673
C	23.358670	2.671482	-2.565039
C	0.859880	38.135213	-2.068706
C	2.184608	37.595196	-2.041566
C	2.423193	36.247416	-1.851659
C	1.378866	35.355428	-1.806064
C	25.761286	-0.025392	-2.610995
C	3.298579	38.371567	-2.310069
C	-3.002758	23.581287	-2.068054
C	-1.301872	28.241283	-1.969338
C	1.867041	24.432378	-1.603855
C	19.265368	13.509344	-2.142309
C	24.216739	13.846306	-2.074713
C	22.033428	9.395213	-1.889293
O	1.329961	27.153728	-1.548851
O	-3.631976	26.333449	-2.075581
O	-0.465472	22.600876	-1.721895
O	0.141637	23.515156	1.779721
O	-1.480289	28.185271	1.642429
O	3.252317	27.376879	1.875398
O	24.177304	10.857057	-1.973752
O	21.945706	15.141303	-2.159389
O	23.520866	15.986533	1.560576
O	19.365351	10.787931	-1.902031
O	25.553944	11.504472	1.613553
O	20.587189	11.795704	1.492618
C	10.858719	18.638639	0.056306
C	10.277300	19.474847	-2.564460
C	12.375817	19.435751	2.888835
C	11.107114	19.365172	0.983330
C	35.897935	18.772541	1.174539
C	-11.101915	18.121453	1.370018
C	36.875175	16.556908	0.719603
C	-9.987014	15.940134	1.117726
C	-0.772861	40.422425	2.001254
C	21.715742	-0.765236	1.874095
C	-3.145822	40.491768	2.664189
C	19.306242	-0.566487	2.345459
C	33.020971	20.613882	-2.061468
C	31.629268	22.632330	-1.839645
C	-15.207369	21.903800	-2.101635
C	-13.904358	19.812614	-2.119149
C	0.743229	39.591288	-2.154974
C	23.461326	-1.595774	-2.068706
C	3.159840	39.705595	-2.610995
C	25.900025	-1.359420	-2.310069

(***P***)-[5]Heli-TFP COF AA-serrated

a = 42.34 Å, b = 44.15 Å, c = 12.15 Å

C	3.716474	27.588687	1.658672
C	5.010416	28.088753	1.439470
N	3.443467	32.759981	1.098437
C	8.542572	26.655953	1.181060
C	8.410436	25.273425	1.368752
C	9.548702	24.468899	1.485845
C	10.837678	25.026886	1.415720
C	10.949858	26.411724	1.218077
C	9.816821	27.216901	1.097963
C	5.201618	29.479443	1.303010
C	4.108571	30.359782	1.356255
N	0.943950	26.658195	2.062960
C	3.783136	34.148681	0.986378
C	5.110647	34.585291	1.118574
C	5.422661	35.941514	1.014274
C	4.420856	36.892127	0.764511
C	3.091273	36.449812	0.646530
C	2.775434	35.090171	0.754620
C	2.809753	29.848380	1.557037
C	2.607599	28.464957	1.718729
N	7.409809	27.527820	1.160745
C	-0.375714	26.114740	2.159324
C	-1.520318	26.924367	2.160745
C	-2.793919	26.339507	2.158021
C	-2.951643	24.941157	2.148722
C	-1.788879	24.151690	2.157784
C	-0.520723	24.729421	2.162226
C	25.281008	15.646177	1.402157
C	24.088107	14.921092	1.343995
N	26.419349	10.613824	1.847194
C	20.536315	16.227571	1.021441
C	20.833261	17.534873	0.610815
C	19.821960	18.461957	0.411692
C	18.480140	18.127857	0.615731

C	18.179300	16.809669	1.016999
C	19.199219	15.867264	1.222341
C	24.109475	13.524626	1.477139
C	25.338102	12.851059	1.632612
N	27.645610	17.110862	2.147893
C	26.364008	9.192149	2.013327
C	25.155794	8.538386	2.300878
C	25.119151	7.148167	2.430705
C	26.284773	6.387343	2.266526
C	27.495928	7.043995	1.996744
C	27.534528	8.438205	1.874616
C	26.542918	13.594659	1.684317
C	26.509678	14.998941	1.627637
N	21.592585	15.281094	1.241590
C	28.711091	17.991734	2.493486
C	30.019809	17.537119	2.683785
C	31.021177	18.439236	3.053603
C	30.733740	19.805793	3.237268
C	29.416164	20.241677	3.041046
C	28.415181	19.343783	2.675552
C	22.867049	15.713954	1.145582
C	27.736407	15.782859	1.909501
C	25.307571	11.380374	1.780326
C	6.139347	27.130004	1.389067
C	1.223160	27.971229	1.912285
C	4.392234	31.804253	1.213280
O	1.733419	30.696533	1.607795
O	3.544980	26.238373	1.817224
O	6.460457	29.992883	1.118989
O	22.938695	12.812393	1.435620
O	27.747530	12.959669	1.833808
O	25.232999	17.006234	1.238037
C	13.350489	24.481355	1.172649
C	12.041385	24.163690	1.634270
C	11.868372	23.000750	2.392622
C	12.946926	22.196188	2.734306
C	14.226082	22.481882	2.266467
C	14.431201	23.563564	1.365258
C	16.104385	18.645365	0.159323
C	17.391798	19.133565	0.393923
C	17.593682	20.546666	0.369462
C	16.482908	21.435633	0.225776
C	15.240514	20.875775	-0.176202
C	15.056737	19.498021	-0.158197
C	18.883373	21.088366	0.431000
C	15.675823	23.818192	0.692076
C	15.931021	25.153595	0.282398
C	14.895958	26.071964	0.181888
C	13.613410	25.715636	0.567283
C	16.697396	22.844271	0.417496
C	18.026238	23.323167	0.272151
C	19.101713	22.450916	0.324982
C	18.277810	24.672768	0.042940
C	17.224494	25.574425	-0.014925
C	15.340758	21.708475	2.887706
C	14.145354	21.677050	-0.793118
C	31.825009	22.136754	3.359573
C	31.821886	20.743003	3.643807
C	32.927782	20.200304	4.310592
C	34.000108	20.997402	4.688110
C	34.054379	22.337035	4.315094
C	33.025422	22.887502	3.514572
C	31.890658	26.240679	2.467071
C	31.892169	24.867350	2.694624
C	33.111353	24.174257	2.900618
C	30.680593	24.181672	2.719559
C	30.660373	22.814264	2.977495
C	34.471114	22.051169	1.190477
C	35.134242	23.174722	4.912109
C	38.047143	22.895609	1.778253
C	38.043847	24.266456	2.064145
C	36.782873	24.920113	2.200961
C	35.563982	24.165334	2.166194
C	35.639299	22.815906	1.714879
C	36.872074	22.193591	1.571666
C	36.708229	26.313023	2.310236
C	34.331352	24.826872	2.509537
C	34.305294	26.243707	2.431297
C	35.487601	26.970127	2.371418
C	33.093777	26.934475	2.395879
C	3.765686	39.262222	1.069060
C	4.745682	38.346127	0.676084
C	5.984091	38.843223	0.188048
C	6.281556	40.242106	0.240228
C	5.209222	41.130193	0.537551
C	3.983173	40.629495	0.969380
C	7.606722	40.674785	-0.116915
C	8.377263	39.798364	-0.928394
C	8.034085	38.458830	-1.052299
C	6.878369	37.979932	-0.456349
C	9.469304	40.271361	-1.650854
C	7.373364	40.742089	2.916313
C	5.237255	42.588540	0.222578
C	8.193512	44.125768	1.354656
C	7.688938	44.986201	2.360106
C	7.032983	44.430657	3.461030
C	6.908418	43.052119	3.592042
C	7.351573	42.196009	2.584875
C	7.890189	42.729615	1.381427
C	8.197031	41.942368	0.219972
C	9.193318	42.447711	-0.657724
C	9.559428	43.788226	-0.618752
C	9.015436	44.630324	0.340441
C	9.835040	41.607867	-1.563611
C	39.384557	24.941157	2.148722
C	7.765473	46.464391	2.266526
C	-4.292353	24.266456	2.064145
C	26.208237	4.909153	2.360106
C	8.159824	26.138387	4.597610
C	9.337380	25.380706	4.762085
N	8.682301	30.479875	3.952679
C	14.235867	24.980124	4.886582
C	14.317789	23.925715	5.804315
C	15.538449	23.280591	6.015876
C	16.698781	23.661074	5.310771
C	16.585918	24.730972	4.406304
C	15.377089	25.390167	4.203686
C	19.057903	23.065351	4.639306
C	17.913715	22.940476	5.487565
C	17.986625	22.018883	6.545313
C	19.133623	21.272934	6.784415
C	20.212362	21.334736	5.908970
C	20.120260	22.116355	4.728385
C	10.586475	26.017561	4.794897
C	10.656758	27.415521	4.651507
N	6.692211	24.214177	4.088429
C	8.995168	31.861701	3.742836
C	10.277810	32.349818	4.024049
C	10.608877	33.667452	3.740407
C	9.684892	34.546098	3.156126
C	8.382416	34.055784	2.911279
C	8.041959	32.725788	3.196164
C	9.009213	36.766659	2.461918
C	10.035128	35.889512	2.842930
C	11.371002	36.404516	2.883442
C	11.610414	37.805618	2.746803
C	10.551322	38.614655	2.253555
C	9.268946	38.090704	2.138002
C	9.488849	28.175060	4.508235
C	8.236092	27.538869	4.504859
N	13.015525	25.674878	4.649552
C	5.406642	23.614652	3.928041
C	4.245369	24.292542	4.310000
C	3.001211	23.698208	4.133146
C	2.868433	22.417333	3.569595
C	4.047562	21.756570	3.169393
C	5.303579	22.344580	3.356079
C	1.496963	20.758976	2.459727
C	1.598082	21.799822	3.394103
C	0.411246	22.184122	4.093404
C	-0.802947	21.442662	3.931772
C	-0.882074	20.548648	2.831440
C	0.272933	20.180814	2.148900
C	31.476555	12.786660	4.250417
C	30.349879	13.617902	4.428337
N	30.720837	8.497706	4.075221
C	25.593612	14.545545	4.980754
C	25.852479	15.906263	4.789685
C	24.808161	16.796579	4.576525
C	23.473179	16.368731	4.539804
C	23.216700	15.005777	4.798629
C	24.267663	14.100691	5.004919
C	21.103848	16.884612	4.557690
C	22.415652	17.280138	4.267297
C	22.610486	18.578041	3.700132
C	21.541141	19.523845	3.659621
C	20.223114	19.032499	3.876868
C	20.027045	17.731809	4.331737
C	29.088929	13.064599	4.705167
C	28.940168	11.667407	4.767416
N	33.047024	14.658418	3.851281
C	30.535242	7.082865	4.121064
C	29.441720	6.505731	4.780446
C	29.318697	5.116832	4.848261
C	30.282185	4.266269	4.266290
C	31.353605	4.874089	3.587363

C	31.483740	6.260086	3.517593
C	31.127315	1.900587	3.929640
C	30.162217	2.851585	4.395762
C	29.035652	2.357441	5.071846
C	28.874070	1.006666	5.333810
C	29.793729	0.079166	4.864727
C	30.871111	0.496799	4.036487
C	30.044506	10.829630	4.546792
C	31.317571	11.393337	4.309882
N	26.667473	13.599213	5.018719
C	34.330991	15.284762	3.809822
C	35.523081	14.561217	3.967664
C	36.742185	15.239242	4.110936
C	36.792419	16.649483	4.125624
C	35.591030	17.342763	3.891201
C	34.379026	16.674460	3.741947
C	38.076569	18.714138	4.791225
C	38.009308	17.337367	4.410509
C	39.210093	16.613883	4.384509
C	40.422922	17.209966	4.709136
C	40.498923	18.569944	5.002135
C	39.334867	19.381439	4.928397
C	23.812054	18.903509	3.057216
C	21.057240	22.038043	3.646827
C	21.223708	23.204405	2.852584
C	20.288704	24.227595	2.893273
C	19.190152	24.123813	3.730516
C	21.849036	20.886365	3.311599
C	23.029769	21.111069	2.552596
C	24.002271	20.129916	2.446637
C	23.248844	22.322481	1.906481
C	22.321163	23.346036	2.010840
C	0.400701	23.308103	4.926679
C	-1.891914	21.696963	4.837304
C	-1.889762	22.950435	5.510486
C	-0.744075	23.721818	5.572853
C	-3.032639	23.440896	6.128468
C	38.146496	22.686396	6.139544
C	38.152384	21.397782	5.621775

C	39.338592	20.811968	5.100986
C	36.965070	20.693252	5.646414
C	36.920816	19.393893	5.186511
C	31.759429	-0.416657	3.374617
C	33.064881	0.044485	3.065922
C	33.349249	1.400468	3.061421
C	32.374863	2.311753	3.438287
C	12.925709	38.305761	3.028076
C	14.007819	37.396006	2.915306
C	13.779412	36.028365	2.911042
C	12.479963	35.545692	2.948355
C	34.090164	-0.854086	2.783406
C	15.313833	37.857790	2.772390
C	6.839854	25.503604	4.461564
C	9.657205	29.623600	4.326288
C	11.800407	25.206571	4.999470
C	27.949312	13.991452	4.839377
C	32.837873	13.340515	4.057098
C	29.822563	9.372462	4.571609
C	21.475752	20.673369	6.349625
C	18.992502	19.771881	3.473705
C	29.680694	-1.294229	5.436985
C	10.764717	39.969183	1.664595
C	-2.174777	20.100181	2.235846
C	41.792713	19.047644	5.573090
O	30.469982	14.978598	4.325340
O	32.416035	10.586850	4.154231
O	27.710041	11.111223	5.010545
O	11.870793	28.055154	4.659621
O	7.086567	28.275511	4.376631
O	9.274830	24.016469	4.890550
C	40.444286	21.696963	4.837304
C	39.303561	23.440896	6.128468
C	-4.189704	22.686396	6.139544
C	-2.997608	20.811968	5.100986
C	13.240129	39.660391	3.374617
C	31.445009	-1.771287	3.028076
C	15.570865	39.222962	2.783406
C	33.833132	-2.219258	2.772390

(*P*)-[5]Heli-TFP COF (AA$_i$:AB)^{stat}

a = 44.82 Å, *b* = 44.43 Å, *c* = 21.91 Å

C	-11.151786	30.555341	-2.079965
C	-9.890242	31.135215	-1.828229
N	-11.835321	35.667244	-2.034563
C	-9.782963	32.544423	-1.704812
C	-10.920082	33.364067	-1.842723
N	-13.907150	29.468397	-2.564892
C	-12.183325	32.763434	-2.049697
C	-12.303938	31.366370	-2.171195
N	-7.472421	30.747088	-1.364191
C	11.367240	20.418368	-4.444278
C	10.117174	19.768252	-4.513979
N	12.286520	15.367530	-5.113797
C	10.073875	18.360364	-4.664893
C	11.262232	17.614265	-4.766354
N	14.088223	21.647879	-4.236452
C	12.507221	18.276928	-4.698145
C	12.566005	19.672504	-4.524850
N	7.638679	19.981708	-4.491598
C	8.949980	20.546732	-4.428078
C	13.836138	20.254429	-4.424454
C	11.158369	16.226370	-4.939223
C	-8.780370	30.283031	-1.706091
C	-13.590091	30.849905	-2.380513
C	-10.752157	34.760836	-1.813521
C	-11.311814	29.307675	1.228837
C	-9.984294	28.671946	1.367601
N	-9.427561	33.804607	2.074209
C	-6.029297	26.928003	1.959532
C	-5.923627	25.538553	2.028594
C	-4.689281	24.938811	2.278198
C	-3.533476	25.716442	2.462791
C	-3.644504	27.113884	2.365379
C	-4.882982	27.717593	2.124727
C	-1.004438	25.784003	2.741384
C	-2.126695	24.911674	2.749484
C	-1.932902	23.580204	3.119307
C	-0.680581	23.106288	3.468668
C	0.436593	23.929276	3.407066
C	0.320250	25.275454	2.945586
C	-8.849620	29.466898	1.641079
C	-8.996079	30.865231	1.764922

N	-13.747015	29.006682	0.867966
C	-10.263855	31.480890	1.629142
C	-11.407718	30.706587	1.357583
N	-7.314405	27.509945	1.742754
C	13.619331	18.015967	2.593668
C	12.389834	18.699322	2.630757
N	11.375321	13.612581	2.480482
C	8.557997	20.764789	2.660811
C	8.551437	22.137850	2.916597
C	7.378669	22.878916	2.759928
C	6.193675	22.256239	2.343850
C	6.203223	20.877539	2.080178
C	7.377916	20.133549	2.241962
C	4.134768	22.018690	1.155725
C	5.001627	22.819222	1.904964
C	4.595716	24.130370	2.256030
C	3.266173	24.570199	1.979142
C	2.490941	23.794501	1.078776
C	2.913684	22.517204	0.712790
C	11.167792	17.986276	2.593028
C	11.191463	16.578656	2.515013
N	16.104697	18.214884	2.771652
C	12.423776	15.883268	2.468546
C	13.631372	16.607524	2.496469
N	9.787958	20.050003	2.784867
C	5.503720	25.023390	2.834532
C	1.435241	26.175348	2.766536
C	1.160004	27.570050	2.842632
C	-0.138332	28.048176	2.742023
C	-1.198914	27.165132	2.644612
C	2.811042	25.794432	2.571499
C	3.810590	26.727590	2.957736
C	5.131188	26.328075	3.119307
C	3.493031	28.064707	3.167693
C	2.180300	28.492719	3.052803
C	-12.425799	28.485510	1.015469
C	-10.454695	32.860558	1.770251
C	-7.571172	28.913665	1.807979
C	9.921304	18.635064	2.628199
C	14.793353	18.775883	2.686177
C	12.504492	14.484178	2.409928

O	-7.965706	31.576949	2.003442
O	-12.536864	31.286613	1.235445
O	-9.886952	27.409054	1.236085
O	-11.247154	29.290886	-2.208071
O	-13.232755	33.483539	-2.130482
O	-8.651241	33.087798	-1.484410
O	14.738563	15.977091	2.443393
O	12.391507	19.972013	2.705574
O	11.408246	21.684471	-4.307219
O	10.090916	15.937020	2.484745
O	13.592998	17.615215	-4.787457
O	8.955900	17.750083	-4.718395
C	1.666542	23.350057	4.012639
C	1.301548	24.342264	0.369824
C	-6.245337	29.841967	-1.134410
C	6.427544	20.871736	-4.325763
C	-6.465294	28.485133	-1.373783
C	6.779676	22.114802	-3.794582
C	-5.387335	27.605888	-1.477163
C	5.806944	23.092703	-3.583985
C	-4.066296	28.065660	-1.344154
C	4.464613	22.842337	-3.902225
C	-3.853663	29.438125	-1.129081
C	4.112939	21.595354	-4.442785
C	-4.933358	30.319689	-1.015896
C	5.088152	20.612385	-4.649759
C	-1.578966	27.550799	-1.511481
C	2.274738	23.320102	-4.728626
C	-2.865646	26.948024	-1.484623
C	3.395011	23.723692	-3.996439
C	-2.948804	25.555498	-1.438582
C	3.359521	24.965658	-3.315410
C	-1.811396	24.768471	-1.422808
C	2.148877	25.718892	-3.254661
C	-0.547054	25.337866	-1.510628
C	1.103830	25.356642	-4.143516
C	-0.397921	26.750956	-1.655360
C	1.161146	24.147200	-4.834777
C	4.519751	25.489720	-2.735629
C	0.866686	27.422620	-1.839739
C	0.942385	28.783588	-1.429416
C	-0.206566	29.524208	-1.196651
C	-1.450556	28.924559	-1.284471
C	2.076346	26.829720	-2.350671
C	3.309728	27.419897	-1.963795
C	4.507151	26.734669	-2.125580
C	3.348269	28.702354	-1.428990
C	2.170811	29.402584	-1.217540
C	0.576360	24.393892	-1.266139
C	-0.001614	26.288464	-4.500977
C	17.238844	19.055680	3.102042
C	29.932304	28.256860	0.762028
C	18.488001	18.511842	3.401951
C	28.740340	28.984857	0.741992
C	19.625619	19.319182	3.397474
C	27.510888	28.326362	0.788886
C	19.538156	20.687572	3.091384
C	27.454763	26.927037	0.856456
C	18.279586	21.220980	2.765257
C	28.653025	26.196418	0.869245
C	17.136197	20.415699	2.778473
C	29.886572	26.856703	0.826827
C	20.756740	22.871142	2.616475
C	26.639569	24.733609	0.375366
C	20.938946	21.551521	3.111634
C	26.374264	26.072542	0.677832
C	22.177062	21.209768	3.657523
C	25.030984	26.479284	0.873508
C	23.213075	22.124647	3.717846
C	23.983958	25.510434	0.909958
C	23.071982	23.399087	3.183041
C	24.277548	24.206252	0.434623
C	21.862088	23.779104	2.526098
C	25.600212	23.823362	0.213794
C	24.710455	27.836403	0.986267
C	21.655782	25.046981	1.867023
C	20.313242	25.505248	1.751280
C	19.245137	24.640525	1.936938
C	19.469920	23.329031	2.317632
C	22.686014	25.919629	1.362699
C	22.380788	27.303633	1.261237
C	23.391706	28.244917	1.111389
C	21.063644	27.746647	1.294063
C	20.030987	26.840869	1.480360
C	24.181785	24.334228	3.510020
C	23.216859	23.253135	0.004689
C	-29.534764	22.132325	-4.001555
C	-15.328064	29.043099	-2.711542
C	-28.443634	21.306824	-3.729143
C	-16.372772	29.968954	-2.662303
C	-27.201473	21.862183	-3.420495
C	-17.686367	29.563128	-2.901676
C	-27.024920	23.255533	-3.378717
C	-17.975345	28.223086	-3.194764
C	-28.123202	24.078639	-3.679904
C	-16.923573	27.294946	-3.250824
C	-29.371286	23.523867	-3.980453
C	-15.606668	27.700940	-3.006335
C	-25.417179	25.217327	-3.168333
C	-19.056600	26.395254	-4.289314
C	-25.520067	23.809152	-3.006335
C	-19.143057	27.653381	-3.686086
C	-24.441740	23.122159	-2.447230
C	-20.417730	28.251703	-3.524941
C	-23.290906	23.784839	-2.058862
C	-21.601379	27.517529	-3.835081
C	-23.149180	25.151219	-2.266262
C	-21.457328	26.326803	-4.593060
C	-24.178319	25.894999	-2.919794
C	-20.196079	25.761418	-4.774454
C	-20.528778	29.581433	-3.105452
C	-24.084492	27.296198	-3.254874
C	-25.306238	28.015052	-3.380635
C	-26.515098	27.349514	-3.522596
C	-26.558915	25.967973	-3.464831
C	-22.867246	28.049609	-3.421135
C	-22.929207	29.451858	-3.199667
C	-21.769323	30.192856	-3.009319
C	-24.149451	30.116833	-3.183680
C	-25.329768	29.405661	-3.332036
C	-21.960618	25.757676	-1.608466
C	-22.586452	25.717691	-5.349333
C	-11.654416	36.999697	-2.118546
C	-9.863618	52.467873	-5.082889
C	-10.394646	37.554073	-1.889830
C	-11.207913	52.090949	-5.033864
C	-10.212777	38.935607	-1.950793
C	-11.559004	50.744696	-4.924728
C	-11.287234	39.792447	-2.243028
C	-10.569591	49.753155	-4.863127
C	-12.547153	39.223313	-2.497322
C	-9.219419	50.132895	-4.919613
C	-12.733465	37.839174	-2.427407
C	-8.866488	51.483282	-5.024485
C	-12.078302	42.153764	-2.769520
C	-9.536864	47.646534	-5.313310
C	-10.967324	41.406168	-2.292906
C	-10.685605	48.374715	-4.988462
C	-9.852961	42.098691	-1.816931
C	-11.887796	47.675855	-4.717968
C	-9.816554	43.481518	-1.807126
C	-11.888888	46.251427	-4.631001
C	-10.867868	44.227458	-2.325306
C	-10.753494	45.561602	-5.129783
C	-11.995677	43.577603	-2.913613
C	-9.583396	46.260580	-5.424150
C	-13.094930	48.369269	-4.579631
C	-13.091718	44.266681	-3.552438
C	-14.344532	43.592235	-3.592937
C	-14.429221	42.228435	-3.355057
C	-13.300303	41.513624	-2.995464
C	-13.051602	45.596328	-4.106214
C	-14.276750	46.312038	-4.182523
C	-14.285658	47.687024	-4.380971
C	-15.495091	45.651097	-4.075733
C	-15.525746	44.285590	-3.839771
C	-10.785104	45.687469	-2.052894
C	-10.794344	44.121804	-5.507707
C	-9.498413	35.037614	2.209989
C	-10.382383	50.863399	2.350458
C	-8.334626	35.740970	2.522474
C	-11.643962	50.263431	2.347047
C	-8.382096	37.119225	2.734350
C	-11.767270	48.881898	2.192297
C	-9.594280	37.822716	2.637151
C	-10.628523	48.078239	2.038186
C	-10.756213	37.109931	2.296530
C	-9.361800	48.683267	2.034776
C	-10.713120	35.727213	2.092967
C	-9.237602	50.068447	2.194642
C	-10.781743	40.073788	2.604752
C	-9.292924	46.302882	1.160202
C	-9.542249	39.446211	2.903381
C	-10.526191	46.750223	1.643423
C	-8.531735	40.211286	3.488278
C	-11.584647	45.820116	1.790287

C	-8.718592	41.552001	3.772414
C	-11.350729	44.426305	1.592266
C	-9.901765	42.196217	3.431153
C	-10.145719	44.047272	0.946620
C	-10.937694	41.492189	2.745221
C	-9.120535	44.976623	0.776736
C	-12.879300	46.260390	2.084867
C	-12.161231	42.096739	2.275214
C	-13.289249	41.241773	2.123448
C	-13.140531	39.863307	2.086572
C	-11.894352	39.292971	2.277133
C	-12.364512	43.495920	1.996408
C	-13.691356	43.996065	2.085507
C	-13.932508	45.362194	2.164587
C	-14.780094	43.131561	2.079751
C	-14.577844	41.760768	2.040105
C	-10.042295	43.568959	3.987060
C	-9.972125	42.721173	0.290530
N	-9.614210	54.026388	-5.113797
N	-30.732377	21.647879	-4.236452
N	31.073585	29.006682	0.867966
N	-10.525409	52.271439	2.480482
C	-14.888296	28.256860	0.762028
C	15.285836	22.132325	-4.001555
C	12.037113	13.809015	-5.082889
C	11.518347	12.204541	2.350458
C	56.800082	17.030162	9.388616
C	58.061627	17.610036	9.640352
N	56.116767	22.141678	9.434018
C	58.169573	19.018858	9.763769
C	57.031786	19.838888	9.625858
N	54.044938	15.942832	8.903689
C	55.768763	19.237869	9.418884
C	55.647930	17.841191	9.297386
N	60.479492	17.221884	10.104603
C	34.498727	6.892803	7.024303
C	33.248443	6.243073	6.954602
N	35.417788	1.842351	6.354784
C	33.205363	4.834798	6.803688
C	34.393719	4.088699	6.702226
N	37.219492	8.122700	7.232129
C	35.638489	4.751749	6.770436
C	35.697273	6.147325	6.943731
N	30.769992	6.456504	6.977196
C	32.081511	7.021141	7.040716
C	36.967625	6.728864	7.044127
C	34.289856	2.700804	6.529358
C	59.171498	16.757852	9.762490
C	54.361997	17.324339	9.088068
C	57.199711	21.235657	9.655060
O	56.704758	15.765681	9.260723
O	54.719333	19.957974	9.338099
O	59.300628	19.562619	9.984171
O	34.539514	8.159292	7.161362
O	36.724485	4.089649	6.681124
O	32.087212	4.224879	6.750399
C	38.531741	8.540897	7.412671
C	52.749951	15.443048	8.618701
C	39.649151	7.705991	7.431216
C	51.708175	16.320621	8.929054
C	40.910879	8.226608	7.719827
C	50.380832	15.932974	8.741905
C	41.081563	9.593871	7.995223
C	50.074376	14.660114	8.240351
C	39.953882	10.431306	7.948756
C	51.122568	13.781928	7.922111
C	38.687020	9.910042	7.668883
C	52.453833	14.168609	8.114164
C	42.688709	11.524133	8.406825
C	48.906845	13.032300	6.938615
C	42.610842	10.107294	8.321776
C	48.869147	14.160214	7.763524
C	43.741552	9.357110	8.647051
C	47.611821	14.689890	8.145497
C	44.919153	9.966895	9.041387
C	46.408779	13.995233	7.818305
C	45.031874	11.351243	9.071016
C	46.488777	12.962554	6.848450
C	43.940707	12.175501	8.658774
C	47.731354	12.468410	6.453688
C	47.531979	15.920384	8.805851
C	43.993321	13.615526	8.573939
C	42.760280	14.316386	8.691813
C	41.547773	13.658925	8.543671
C	41.518566	12.288259	8.355881
C	45.184146	14.415193	8.434962
C	45.134291	15.753445	8.909871
C	46.301687	16.475845	9.123452
C	43.916169	16.376012	9.156917
C	42.731987	15.675363	8.991509
C	46.273026	11.858388	9.715383
C	45.297506	12.471789	6.101556
C	17.106860	16.416250	10.160023
C	29.550081	7.156960	6.892574
C	16.871239	15.046609	10.038738
C	29.935912	8.434585	7.304815
C	17.939767	14.155951	9.937064
C	28.978473	9.431387	7.499425
C	19.267169	14.616782	9.953263
C	27.619210	9.165027	7.283713
C	19.494230	16.000172	10.049822
C	27.233914	7.882663	6.862519
C	18.424426	16.893923	10.161302
C	28.193212	6.881155	6.671745
C	21.738856	14.076524	9.673817
C	25.383073	9.591334	6.558986
C	20.455238	13.484901	9.825370
C	26.546428	10.043029	7.188432
C	20.374051	12.100502	9.982465
C	26.554328	11.333453	7.773542
C	21.509818	11.309995	9.988434
C	25.350589	12.095957	7.850704
C	22.766375	11.864229	9.780395
C	24.252359	11.673264	7.057769
C	22.907884	13.261293	9.519493
C	24.265876	10.414297	6.458377
C	27.748669	11.893401	8.239499
C	24.159191	13.909812	9.207221
C	24.261791	15.297346	9.507343
C	23.129653	16.060446	9.752685
C	21.882063	15.462562	9.787216
C	25.334972	13.272874	8.669858
C	26.590223	13.883684	8.934596
C	27.774860	13.181160	8.752349
C	26.663134	15.202674	9.366448
C	25.501515	15.923619	9.595590
C	23.901584	10.934719	10.026802
C	23.127825	12.581344	6.698390
C	33.321469	-15.006207	9.308257
C	35.267636	0.442841	6.342208
C	34.614009	-14.491792	9.413982
C	33.924421	0.104459	6.522963
C	34.829843	-13.114881	9.349609
C	33.545938	-1.232642	6.652774
C	33.757466	-12.223475	9.176954
C	34.506037	-2.252824	6.603536
C	32.462666	-12.752983	9.045224
C	35.854549	-1.911550	6.414680
C	32.242489	-14.131777	9.118975
C	36.235494	-0.570676	6.288919
C	32.989038	-9.833550	8.758317
C	35.424439	-4.382221	6.028870
C	34.119359	-10.619561	9.111941
C	34.337214	-3.625600	6.476069
C	35.296109	-9.965697	9.480060
C	33.147514	-4.293340	6.859321
C	35.375138	-8.584954	9.500949
C	33.113141	-5.718246	6.930728
C	34.299585	-7.802187	9.099578
C	34.170830	-6.433359	6.312366
C	33.099422	-8.410980	8.621685
C	35.325534	-5.764334	5.908438
C	31.981996	-3.567217	7.126404
C	31.965590	-7.682151	8.104359
C	30.695440	-8.319350	8.183652
C	30.594045	-9.683092	8.414499
C	31.731614	-10.435057	8.650248
C	31.989850	-6.346672	7.563372
C	30.785112	-5.594791	7.619218
C	30.797405	-4.217518	7.437397
C	29.564680	-6.221871	7.841752
C	29.516591	-7.588983	8.065138
C	34.453159	-6.348656	9.377745
C	34.048822	-7.866661	5.925277
N	33.196897	-16.517180	9.434018
N	15.658892	17.221884	10.104603
C	61.927460	16.416250	10.160023
C	56.241339	23.652651	9.308257
C	11.819454	15.782496	12.697418
C	13.146974	15.146767	12.836182
N	13.703707	20.279428	13.542790
C	14.281649	15.941719	13.109660
C	14.135452	17.339640	13.233716
N	9.384253	15.481503	12.336547
C	12.867414	17.955711	13.097723
C	11.723770	17.181022	12.826164

N	15.816908	13.984741	13.211548
C	-8.070001	4.490788	14.062248
C	-9.299453	5.174117	1 4.099550
N	-10.314010	0.087402	13.949063
C	-10.521496	4.461072	14.061822
C	-10.497649	3.053091	13.983594
N	-5.584634	4.689705	14.240233
C	-9.265336	2.357702	13.937126
C	-8.057740	3.081959	13.965050
N	-11.901155	6.524438	14.253448
C	10.705688	14.959945	12.484050
C	12.676793	19.334992	13.238831
C	15.560097	15.388486	13.276560
C	-11.768027	5.109885	14.096779
C	-6.895759	5.250317	14.154757
C	-9.184795	0.958974	13.878722
O	15.165781	18.051383	13.472023
O	10.594624	17.761048	12.704026
O	13.244317	13.883875	12.704665
O	-6.950769	2.451912	13.911974
O	-9.297825	6.446834	14.174155
O	-11.598416	2.411841	13.953326
C	-4.489349	5.487262	14.377291
C	8.256975	14.686870	12.341450
C	-3.239734	4.869258	14.425038
C	7.102884	15.431280	12.594464
C	-2.075591	5.637628	14.439532
C	5.858599	14.802704	12.659690
C	-2.136812	7.041113	14.405428
C	5.749647	13.417733	12.471900
C	-3.399671	7.653764	14.331037
C	6.909783	12.671626	12.210359
C	-4.567865	6.885419	14.326134
C	8.158600	13.301219	12.149610
C	-0.884663	9.248013	14.207194
C	4.793395	11.373327	11.688556
C	-0.706515	7.854983	14.424185
C	4.620589	12.635993	12.263222
C	0.567821	7.388790	14.750738
C	3.325692	13.027270	12.684416
C	1.642751	8.253413	14.860087
C	2.247530	12.092205	12.660756
C	1.499293	9.608860	14.591512
C	2.431321	10.895882	11.920682
C	0.242710	10.132637	14.160513
C	3.704035	10.533010	11.481583
C	3.077820	14.345092	13.083655
C	0.020147	11.506724	13.776834
C	-1.305816	12.007457	13.903448
C	-2.383747	11.146541	14.047114
C	-2.175209	9.782203	14.149002
C	1.025525	12.436193	13.328143
C	0.771232	13.820508	13.521475
C	1.799428	14.751363	13.433655
C	-0.514138	14.277690	13.787492
C	-1.558389	13.375947	13.920074
C	2.677496	10.440546	14.956433
C	1.289939	10.064759	11.446413
C	13.538751	21.542345	13.713314
C	12.690498	37.370433	13.621871
C	14.709075	22.247836	13.994678
C	11.428006	36.773381	13.649367
C	14.668095	23.629310	14.185878
C	11.299121	35.390184	13.518490
C	13.455958	24.334347	14.098485
C	12.432974	34.581558	13.357345
C	12.286625	23.618629	13.789197
C	13.700785	35.183309	13.322814
C	12.323721	22.232667	13.606523
C	13.830342	36.570540	13.458807
C	12.273070	26.587158	14.051377
C	13.749697	32.789359	12.484477
C	13.516239	25.961330	14.338497
C	12.525734	33.247433	12.981553
C	14.538187	26.732977	14.893978
C	11.467909	32.321948	13.161243
C	14.358641	28.078515	15.160209
C	11.695387	30.924884	12.980914
C	13.171738	28.719975	14.829179
C	12.888415	30.532514	12.320560
C	12.122474	28.007880	14.172236
C	13.912698	31.457142	12.118703
C	10.179472	32.770071	13.471170
C	10.891735	28.608311	13.714167
C	9.759989	27.753740	13.595013
C	9.905078	26.374552	13.577321
C	11.153115	25.804157	13.755305
C	10.687039	30.003089	13.417029
C	9.362916	30.507654	13.521048
C	9.126074	31.875413	13.583076
C	8.272633	29.645915	13.547479
C	8.471209	28.274164	13.525738
C	13.043280	30.101533	15.366116
C	13.048177	29.196540	11.682588
C	17.029518	13.443895	13.669191
C	31.690105	7.433081	14.108290
C	17.151188	12.054445	13.689227
C	31.668582	8.796192	14.412248
C	18.393816	11.460771	13.913679
C	30.486635	9.528340	14.285208
C	19.541149	12.244907	14.121719
C	29.307338	8.907008	13.851225
C	19.412886	13.643845	14.073759
C	29.332297	7.538258	13.539380
C	18.167012	14.240712	13.858259
C	30.516300	6.802849	13.671109
C	22.069972	12.332838	14.394770
C	27.247090	8.686595	12.662034
C	20.958264	11.447862	14.375799
C	28.107502	9.470647	13.436213
C	21.169128	10.106164	14.698089
C	27.686929	10.763878	13.834173
C	22.427489	9.635467	15.026774
C	26.352200	11.197134	13.576895
C	23.535575	10.472851	14.990537
C	25.582557	10.444388	12.652442
C	23.401184	11.833458	14.577017
C	26.018849	9.185656	12.240627
C	28.587173	11.646635	14.440598
C	24.504988	12.752085	14.425890
C	24.213345	14.139753	14.551652
C	22.909568	14.605744	14.471932
C	21.859050	13.713968	14.347023
C	25.884356	12.394525	14.213162
C	26.874094	13.325363	14.628814
C	28.200239	12.936110	14.772267
C	26.541902	14.650260	14.886518
C	25.223606	15.066725	14.790811
C	24.773742	9.888052	15.571384
C	24.384295	11.002522	11.966937
N	12.605859	38.746260	13.949063
N	32.919445	6.524438	14.253448
C	-10.229372	-1.288425	13.621871
C	-13.130495	7.433081	14.108290

S23. References

(1) Ravat, P.; Hinkelmann, R.; Steinebrunner, D.; Prescimone, A.; Bodoky, I.; Juríček, M. Configurational Stability of [5]Helicenes. *Org. Lett.* **2017**, *19*, 3707–3710. DOI: 10.1021/acs.orglett.7b01461.
(2) Wolfe, J. P.; Åhman, J.; Sadighi, J. P.; Singer, R. A.; Buchwald, S. L. An Ammonia Equivalent for the Palladium-Catalyzed Amination of Aryl Halides and Triflates. *Tetrahedron Lett.* **1997**, *38*, 6367–6370. DOI: 10.1016/S0040-4039(97)01465-2.
(3) Fulmer, G. R.; Miller, A. J. M.; Sherden, N. H.; Gottlieb, H. E.; Nudelman, A.; Stoltz, B. M.; Bercaw, J. E.; Goldberg, K. I. NMR Chemical Shifts of Trace Impurities: Common Laboratory Solvents, Organics, and Gases in Deuterated Solvents Relevant to the Organometallic Chemist. *Organometallics* **2010**, *29*, 2176–2179. DOI: 10.1021/om100106e.
(4) Sheldrick, G.M. SADABS. **1996**, University of Göttingen, Germany. DOI: 10.1107/S010827019700752X.
(5) Sheldrick, G. M. *Acta Crystallogr. A.* **2015**, *71*, 3–8. DOI: 10.1107/S0108767307043930.
(6) Sheldrick, G. M. *Acta Crystallogr. C.* **2015**, *71*, 3–8. DOI: 10.1107/S2053229614024218.
(7) Hübschle, C. B.; Sheldrick, G. M.; Dittrich, B. ShelXle: a Qt graphical user interface for SHELXL. *J. Appl. Crystallogr.* **2011**, *44*, 1281–1284. DOI: 10.1107/S0021889811043202.
(8) Spek, A. L. Structure validation in chemical crystallography. *Acta Crystallogr. D.* **2009**, *65*, 148–155. DOI: 10.1107/S090744490804362X.
(9) Smales, G. J.; Pauw, B. R. The MOUSE project: a meticulous approach for obtaining traceable, wide-range X-ray scattering information. *J. INSTRUM.* **2021**, *16*, P06034. DOI: 10.1088/1748-0221/16/06/P06034.
(10) a) Filik, J.; Ashton, A. W.; Chang, P. C. Y.; Chater, P. A.; Day, S. J.; Drakopoulos, M.; Gerring, M. W.; Hart, M. L.; Magdysyuk, O. V.; Michalik, S.; Tang, C. C.; Terrill, N. J.; Wharmb, M. T.; Wilhelm, H. Processing two-dimensional X-ray diffraction and small-angle scattering data in DAWN 2. *J. Appl. Crystallogr.* **2017**, *50*, 959–966. DOI: doi:10.1107/S1600576717004708. b) Pauw, B. R.; Smith, A. J.; Snow, T.; Terrill, N. J.; Thunemann, A. F. The modular small-angle X-ray scattering data correction sequence. *J. Appl. Crystallogr.* **2017**, *50*, 1800–1811. DOI: 10.1107/S1600576717015096.
(11) BIOVIA, Dassault Systèmes. Materials Studio: San Diego **2022**.
(12) Osterrieth, J. W. M.; Rampersad, J.; Madden, D.; Rampal, N.; Skoric, L.; Connolly, B.; Allendorf, M. D.; Stavila, V.; Snider, J. L.; Ameloot, R.; Marreiros, J.; Ania, C.; Azevedo, D.; Vilarrasa-Garcia, E.; Santos, B. F.; Bu, X. H.; Chang, Z.; Bunzen, H.; Champness, N. R.; Griffin, S. L.; Chen, B.; Lin, R. B.; Coasne, B.; Cohen, S.; Moreton, J. C.; Colón, Y. J.; Chen, L.; Clowes, R.; Coudert, F. X.; Cui, Y.; Hou, B.; D'Alessandro, D. M.; Doheny, P. W.; Dincă, M.; Sun, C.; Doonan, C.; Huxley, M. T.; Evans, J. D.; Falcaro, P.; Ricco, R.; Farha, O.; Idrees, K. B.; Islamoglu, T.; Feng, P.; Yang, H.; Forgan, R. S.; Bara, D.; Furukawa, S.; Sanchez, E.; Gascon, J.; Telalović, S.; Ghosh, S. K.; Mukherjee, S.; Hill, M. R.; Sadiq, M. M.; Horcajada, P.; Salcedo-Abraira, P.; Kaneko, K.; Kukobat, R.; Kenvin, J.; Keskin, S.; Kitagawa, S.; Otake, K.; Lively, R. P.; DeWitt, S. J. A.; Llewellyn, P.; Lotsch, B. V.; Emmerling, S. T.; Pütz, A. M.; Martí-Gastaldo, C.; Padial, N. M.; García-Martínez, J.; Linares, N.; Maspoch, D.; Suárez del Pino, J. A.; Moghadam, P.; Oktavian, R.; Morris, R. E.; Wheatley, P. S.; Navarro, J.; Petit, C.; Danaci, D.; Rosseinsky, M. J.; Katsoulidis, A. P.; Schröder, M.; Han, X.; Yang, S.; Serre, C.; Mouchaham, G.; Sholl, D. S.; Thyagarajan, R.; Siderius, D.; Snurr, R. Q.; Goncalves, R. B.; Telfer, S.; Lee, S. J.; Ting, V. P.; Rowlandson, J. L.; Uemura, T.; Iiyuka, T.; van der Veen, M. A.; Rega, D.; Van Speybroeck, V.; Rogge, S. M. J.; Lamaire, A.; Walton, K. S.; Bingel, L. W.; Wuttke, S.; Andreo, J.; Yaghi, O.; Zhang, B.; Yavuz, C. T.; Nguyen, T. S.; Zamora, F.; Montoro, C.; Zhou, H. C.; Kirchon, A.; Fairen-Jimenez; D. How Reproducible are Surface Areas Calculated from the BET Equation? *Adv. Mater.* **2022**, *34*, 2201502–2201514. DOI: 10.1002/adma.202201502.
(13) Nečas, D.; Klapetek, P. Gwyddion: an open-source software for SPM data analysis. *Cent. Eur. J. Phys.* **2012**, *10*, 181–188. DOI: 10.2478/s11534-011-0096-2.
(14) Schindelin, J.; Arganda-Carreras, I.; Frise, E.; Kaynig, V.; Longair, M.; Pietzsch, T.; Preibisch, S.; Rueden, C.; Saalfeld, S.; Schmid, B.; Tinevez, J. Y.; White, D. J.; Hartenstein, W.; Eliceiri, K.; Tomancak, P.; Cardona, A. Fiji: an open-source platform for biological-image analysis. *Nature Methods* **2012**, *9*, 676–682. DOI: 10.1038/nmeth.2019.
(15) de Mello, J. C.; Wittmann, H. F.; Friend, R. H. An improved experimental determination of external photoluminescence quantum efficiency. *Adv. Mater.* **1997**, *9*, 230–232. DOI: 10.1002/adma.19970090308.
(16) Chen, D.; Xu, G.; Zhou, Q.; Chung, L. W.; Tang, W. Practical and Asymmetric Reductive Coupling of Isoquinolines Templated by Chiral Diborons. *J. Am. Chem. Soc.* **2017**, *139*, 9767–9770. DOI: 10.1021/jacs.7b04256.
(17) Hitosugi, S.; Tanimoto, D.; Nakanishi, W.; Isobe, H. A Facile Chromatographic Method for Purification of Pinacol Boronic Esters. *Chem. Lett.* **2012**, *41*, 972–973. DOI: 10.1246/cl.2012.972.
(18) Jousselin-Oba, T.; Mamada, M.; Wright, K.; Marrot, J.; Adachi, C.; Yassar, A.; Frigoli, M. Synthesis, Aromaticity, and Application of peri-Pentacenopentacene: Localized Representation of Benzenoid Aromatic Compounds. *Angew. Chem. Int. Ed.* **2022**, *61*, e202112794. DOI: 10.1002/anie.202112794.
(19) Kondo, Y.; Kadota, T.; Hirazawa, Y.; Morisaki, K.; Morimoto, H.; Ohshima, T. Scandium(III) Triflate Catalyzed Direct Synthesis of N-Unprotected Ketimines. *Org. Lett.* **2020**, *22*, 120–125. DOI: 10.1021/acs.orglett.9b04038.
(20) Nguyen, H. L.; Gropp, C.; Ma, Y.; Zhu, C.; Yaghi, O. M. 3D Covalent Organic Frameworks Selectively Crystallized through Conformational Design. *J. Am. Chem. Soc.* **2020**, *142*, 20335–20339. DOI: 10.1021/jacs.0c11064.
(21) Zhou, Z.; Zhang, L.; Yang, Y.; Vitorica-Yrezabal, I. J.; Wang, H.; Tan, F.; Gong, L.; Li, Y.; Chen, P.; Dong, X.; Liang, Z.; Yang, J.; Wang, C.; Hong, Y.; Qiu, Y.; Gölzhäuser, A.; Chen, X.; Qi, H.; Yang, S.; Liu, W.; Sun, J.; Zheng,

Z. Growth of single-crystal imine-linked covalent organic frameworks using amphiphilic amino-acid derivatives in water. *Nature Chem.* **2023**, *15*, 841–847. DOI: 10.1038/s41557-023-01181-6.
(22) Rawat, K. S.; Borgmans, S.; Braeckevelt, T.; Stevens, C. V.; Van Der Voort, P.; Van Speybroeck, V. How the Layer Alignment in Two-Dimensional Nanoporous Covalent Organic Frameworks Impacts Its Electronic Properties. *ACS Appl. Nano Mater.* **2022**, *5*, 14377–14387. DOI: 10.1021/acsanm.2c02647.
(23) Zhang, Y.; Položij, M.; Heine, T. Statistical Representation of Stacking Disorder in Layered Covalent Organic Frameworks. *Chem. Mater.* **2022**, *34*, 2376–2381. DOI: 10.1021/acs.chemmater.1c04365.
(24) Bruhn, T.; Schaumlöffel, A.; Hemberger, Y.; Bringmann, G. SpecDis: Quantifying the Comparison of Calculated and Experimental Electronic Circular Dichroism Spectra. *Chirality* **2013**, *25*, 243–249. DOI: 10.1002/chir.22138.
(25) Dhbaibi, K.; Favereau, L.; Crassous, J. Enantioenriched Helicenes and Helicenoids Containing Main-Group Elements (B, Si, N, P). *Chem. Rev.* **2019**, *119*, 8846–8953. DOI: 10.1021/acs.chemrev.9b00033.
(26) Ishii, A.; Miyasaka, T. Direct detection of circular polarized light in helical 1D perovskite-based photodiode. *Sci. Adv.* **2020**, *6*, eabd3274. DOI: 10.1126/sciadv.abd3274.
(27) Gu, Q.; Zha, J.; Chen, C.; Wang, X.; Yao, W.; Liu, J.; Kang, F.; Yang, J.; Li, Y. Y.; Lei, D.; Tang, Z.; Han, Y.; Tan, C.; Zhang, Q. Constructing Chiral Covalent-Organic Frameworks for Circularly Polarized Light Detection. *Adv. Mater.* **2024**, *36*, 2306414. DOI: 10.1002/adma.202306414.
(28) Du, C.; Zhu, X.; Yang, C.; Liu, M. Stacked Reticular Frame Boosted Circularly Polarized Luminescence of Chiral Covalent Organic Frameworks. *Angew. Chem. Int. Ed.* **2022**, *61*, e202113979.
DOI: 10.1002/anie.202113979.
(29) Weng, W.; Guo, J. Chiral Covalent Organic Framework Films with Enhanced Photoelectrical Performances. *J. Am. Chem. Soc.* **2024**, *146*, 13201–13209. DOI: 10.1021/jacs.4c01097.
(30) Flack, H. D.; Shmueli, U. The mean-square Friedel intensity difference in P1 with a centrosymmetric substructure. *Acta Cryst.* **2007**, *63*, 257–265. DOI: doi:10.1107/S0108767307002802.
(31) Flack, H. D.; Bernardinelli, G. Reporting and evaluating absolute-structure and absolute-configuration determinations. *Journal of Applied Crystallography* **2000**, *33*, 1143–1148. DOI: doi:10.1107/S0021889800007184.
(32) Kandambeth, S.; Mallick, A.; Lukose, B.; Mane, M. V.; Heine, T.; Banerjee, R. Construction of Crystalline 2D Covalent Organic Frameworks with Remarkable Chemical (Acid/Base) Stability via a Combined Reversible and Irreversible Route. *J. Am. Chem. Soc.* **2012**, *134*, 19524–19527. DOI: 10.1021/ja308278w.
(33) Vitaku, E.; Dichtel, W. R. Synthesis of 2D Imine-Linked Covalent Organic Frameworks through Formal Transimination Reactions. *J. Am. Chem. Soc.* **2017**, *139*, 12911–12914. DOI: 10.1021/jacs.7b06913.
(34) Ma, T.; Kapustin, E. A.; Yin, S. X.; Liang, L.; Zhou, Z.; Niu, J.; Li, L. H.; Wang, Y.; Su, J.; Li, J.; Wang, X.; Wang, W. D.; Wang, W.; Sung, J.; Yaghi, O. M. Single-Crystal X-Ray Diffraction Structures of Covalent Organic Frameworks. *Science.* **2018**, *361*, 48–52. DOI: 10.1126/science.aat7679.
(35) Würth, C.; Grabolle, M.; Pauli, J.; Spieles, M.; Resch-Genger, U. Relative and absolute determination of fluorescence quantum yields of transparent samples. *Nature Protocols* **2013**, *8*, 1535–1550.
DOI: 10.1038/nprot.2013.087.
(36) Kitzmann, W. R.; Freudenthal, J.; Reponen, A. P. M.; VanOrman, Z. A.; Feldmann, S. Fundamentals, Advances, and Artifacts in Circularly Polarized Luminescence (CPL) Spectroscopy. *Adv. Mater.* **2023**, *35*, 2302279. DOI: 10.1002/adma.202302279.
